# Comprehensive network pharmacology and experimental study to investigate the effect and mechanism of solasonine on breast carcinoma treatment

**DOI:** 10.1186/s12935-025-03665-6

**Published:** 2025-02-17

**Authors:** Wenkai Ge, Min Gao, Yingqi Dai, Gang Zheng, Li Yang, Wenshu Zuo, Xingsong Tian

**Affiliations:** 1 Shandong Provincial Hospital, Shandong University, Jinan, China; 2 Shandong Cancer Hospital and Institute, Shandong First Medical University and Shandong Academy of Medical Sciences, Jinan, China; 3 Shandong Provincial Third Hospital, Shandong University, Jinan, China; 4 Shandong Provincial Hospital Affiliated to Shandong First Medical University, Jinan, China

**Keywords:** Breast carcinoma, Ferroptosis, Solasonine, Network pharmacology, Molecular docking, ERK2/MAPK pathway

## Abstract

**Background:**

Ferroptosis is a therapeutic strategy for breast carcinoma (BC). Solasonine (SS) was linked to ferroptosis as a tumor suppressor. However, whether SS could treat BC by activating ferroptosis and its underlying mechanisms has not been reported.

**Methods:**

We obtained the intersection of genes targeting SS and BC disease through network pharmacology. Bioinformatics analysis revealed that the intersection genes were primarily enriched in the extracellular signal-regulated kinase 2/mitogen-activated protein kinase (ERK2/MAPK) signaling pathway. The interaction modes of SS with ERK2 and epidermal growth factor receptor (EGFR) were simulated by molecular docking. We further detected the expressions of ERK2 and p-ERK2 in BC patients and the correlation between ERK2/p-ERK2 and ferroptosis. The effects and mechanism of SS on ferroptosis in BC were validated by mutation plasmids construction, immunohistology, wound healing, transwell assay, and western blotting using in *vitro* and in vivo models.

**Results:**

ERK2 and p-ERK2 were up-regulated in BC patients, and the ERK2/p-ERK2 ratio was negatively correlated with ferroptosis. Molecular docking indicated that SS could bind to ERK2 and EGFR to inhibit the activity of the ERK2/MAPK pathway. In vitro and in vivo experiments confirmed that SS induced ferroptosis by inhibiting the ERK2/MAPK pathway, inhibiting proliferation, migration, and invasion of BC cells.

**Conclusion:**

SS could inactivate the ERK2/MAPK pathway, thereby inducing ferroptosis and further inhibiting BC cell proliferation, migration, and invasion. This study clarified the potential mechanism of SS in BC and provided a theoretical basis for its clinical application.

**Supplementary Information:**

The online version contains supplementary material available at 10.1186/s12935-025-03665-6.

## Introduction

Breast carcinoma (BC) has been reported to be a common cancer in women and a leading cause of cancer-related deaths [52]. The conventional treatment for BC primarily consists of surgery combined with radiotherapy, chemotherapy, and targeted therapy [24, 54, 60]. Current BC treatments often lead to relapse and are associated with drug resistance and toxic side effects [4, 20, 59, 66]. Targeted therapy, while effective, comes with side effects [24]. Therefore, new strategies for treating BC are needed.

Ferroptosis has emerged as an important player in the pathogenic mechanism of various diseases, including tumors, and targeting ferroptosis is becoming an effective new way to treat cancers [51, 64]. Unlike apoptosis and classical necrosis, ferroptosis is characterized by altered glutathione (GSH) metabolism and lipid peroxidation [10, 15]. It has been reported that Sanguinarine regulates STUB1/glutathione peroxidase 4 (GPX4)-dependent ferroptosis and inhibits the progression of non-small cell lung carcinoma (NSCLC) [63]. Anomanolide C is shown to induce ferroptosis by ubiquitinating GPX4, which suppresses tumor progression in triple-negative breast cancer (TNBC) [13]. Also, Escin inhibits BC tumor growth by modulating the glucose-6-phosphate dehydrogenase (G6PD)/GPX4-mediated ferroptosis pathway [30]. Moreover, BC cells exhibit ferroptosis sensitivity [44]. Thus, targeting ferroptosis becomes a promising strategy for BC treatment.

Traditional Chinese medicine (TCM) is beneficial to treat BC [13, 30, 34, 65]. Furthermore, Solasonine (SS), a natural sugar alkaloid found in *Solanum nigrum* L. plants, plays a potential anti-tumor role against a variety of carcinomas, including BC [1, 16, 32, 68]. SS is also verified to inhibit stemness and migratory properties of osteosarcoma cells [56]. SS can activate ferroptosis and inhibit pancreatic cancer and lung adenocarcinoma [32, 68]. However, it has not been reported whether SS can inhibit the development of BC through the ferroptosis pathway.

We have uncovered the converging genetic pathways of SS and BC disease targets through rigorous network pharmacology analysis. Subsequent bioinformatics analyses, including Gene Ontology (GO) and Kyoto Encyclopedia of Genes and Genomes (KEGG), predicted that the intersection genes were primarily enriched in extracellular signal-regulated kinase 2 (ERK2)/mitogen-activated protein kinase (MAPK) signaling pathway. This crucial signaling pathway is modulated during the BC treatment. MAPK1 (ERK2) is known as part of the classical MAPK signaling pathway and is associated with cell differentiation and proliferation [39]. ERK2 dysregulation is related to BC [8, 17]. What’s more, the inhibition of ERK induces ferroptosis in hepatocellular carcinoma (HCC) [35, 57]. Additionally, Dong et al. have demonstrated that SS exerts inhibitory effects on the ERK/MAPK pathway in bladder cancer, further cementing its potential to treat cancer [16].

Building on this robust foundation, we established in vitro and in vivo models of BC to investigate how SS prevents cancer progression by inducing ferroptosis as well as inhibiting the ERK2/MAPK pathway using immunohistology, wound healing, transwell assays, and western blot analyses.

## Materials and methods

### Potential SS target screening

First, a canonical SMILES of SS was downloaded from the PubChem database (https://pubchem.ncbi.nlm.nih.gov/). Then, we predicted the possible targets of SS using the SwissTargetPrediction database (probability > 0.05) (http://swisstargetprediction.ch/) [28], PharmMapper database (Norm fit > 0.6) (https://www.lilab-ecust.cn/pharmmapper/) [61], and similarity ensemble approach database (Max Tc > 0.4) (https://sea.bkslab.org/) [23, 27]. Combinations of these databases have been taken as the potential targets for SS.

### Disease targets of BC and intersection genes of SS

The search term "breast carcinoma" was applied in the GeneCards database (relevance score > 10) (https://www.genecards.org/) [18, 70], DisGeNET database (score_gda ≥ 0.3) (https://www.disgenet.org/) [48] and Therapeutic Target database (https://db.idrblab.net/ttd/) [74] to obtain BC-related target genes. The union set of the output data of the three databases was taken as the total number of targets for BC. Then, we used Venn diagrams to determine the intersection genes of SS-related and BC-related targets (https://jvenn.toulouse.inrae.fr/app/index.html), which served as potential targets for SS in treating BC.

### Protein–protein interaction (PPI) network

The STRING (confidence = 0.4) (https://cn.string-db.org/) [11, 72] was used to construct the PPI network of intersection genes of SS-related and BC-related targets. Then, we used Cytoscape 3.10.1 software to analyze the Degree value of the intersection genes to further narrow down the screened targets. The color and size of nodes were related to the Degree value.

### Bioinformatics analysis of intersection genes

GO functional annotation and KEGG pathway enrichment of the intersection genes were analyzed using Metascape **(**https://metascape.org/gp/index.html#/main/step1). Then, the network diagram of KEGG results was established by Cytoscape 3.10.1 software.

### Molecular docking

The mol2 format of the SS structure file was downloaded from Traditional Chinese Medicine Systems Pharmacology (TCMSP) database (https://old.tcmsp-e.com/molecule.php?qn=371). Next, the SDF file of the SS structure was retrieved from PubChem (https://pubchem.ncbi.nlm.nih.gov/) and converted into a mol2 file using OpenBabel-2.4.1. AutoDockTools1.5.6 was then used to balance the charge and check for rotatable bonds within the small molecule. Additionally, we obtained the PDB files of ERK2 (1PME, Residues: 1-360) and EGFR protein [1IVO, extracellular domain, Residues: 25-646; 1M14, intracellular domain (containing the kinase domain), Residues: 695-1022] from Protein Data Bank (PDB) (https://www.rcsb.org/). Pymol and AutoDockTools1.5.6 were used to analyze ligand removal and hydrogenation, respectively. Then, the molecular docking was performed using AutoDockTools1.5.6, and the docking results were visualized using Pymol. Each molecular docking calculation was performed three times, and the estimated inhibition constants (Ki) were obtained from the docking log files.

### Protein–protein docking

We obtained the PDB files of ERK2 (1PME, Residues: 1-360) and mitogen-activated extracellular signal-regulated kinase (MEK) proteins (2DYL, Residues: 101-405) from the UniProt (https://www.uniprot.org/), and then used the Pymol software to remove water, ions, and ligands. After processing, the PDB files of the two proteins were docked using HDOCK (http://hdock.phys.hust.edu.cn/). Next, the binding sites were visualized with Pymol. MEK and ERK2 were ligands and receptors in order.

### Clinical BC tumor tissue collection

The clinical experiments were approved by the Human Research Ethics Committee of Shandong Cancer Hospital and Institute, Shandong First Medical University, and Shandong Academy of Medical Sciences (approval number: SDTHEC2024007036; approval date: July 30, 2024). Tumor tissues and corresponding normal breast tissues (distance from tumor ≥ 5 cm) from BC patients from our hospital were selected. As described previously [45, 55]. The inclusion criteria for selected patients included: (1) a confirmed diagnosis with BC, according to the diagnostic criteria,and (2) completed surgical treatment, in addition to normal liver and kidney function tests. The exclusion criteria were: (1) patients with other malignant tumors; and (2) patients who had received pre-surgery radiotherapy or chemotherapy doses). All patients had complete clinical data and signed the written informed consent forms.

### Cell culture

MCF-7 and MDA-MB-231 cell lines (ATCC) were cultured in Dulbecco’s modified Eagle’s medium (DMEM) (Hyclone, USA) supplemented with 10% fetal bovine serum (Gibco, USA) and 1% penicillin and streptomycin (Gibco, USA) [71]. Normal human mammary epithelial cell line (HMEC, Lonza) was cultured in the epithelial cell basal medium (Lonza, USA) [25]. All cell lines were grown in a humidified chamber at 37 ℃ with 5% CO_2_.

### CCK-8 assay

We used Cell Counting Kit-8 (CCK-8, Dojindo, Japan) to screen the optimal SS concentration for treating BC cells. Briefly, cells were inoculated on 96-well plates (5 × 10^3^ cells/well) for 24 h, then treated with either vehicle [Dimethyl sulfoxide (DMSO)] or SS (19121-58-5, C_45_H_73_NO_16_, purity ≥ 98%, Desite, China) at differing concentrations (0.5 μg/mL, 1 μg/mL, 2 μg/mL, 4 μg/mL and 8 μg/mL) for 24 h [26, 42]. Then, we added CCK-8 buffer and measured the absorbance at 450 nm in a microplate reader (Thermo Scientific, NY, USA). Dose–response curves were plotted to calculate IC_50_ values using GraphPad Prism Software (version 8.0.1), and the IC_50_ values of MCF-7 cells and MDA-MB-231 cells were 2.92 μg/mL and 3.87 μg/mL respectively. We chose 3 μg/mL as the optimal SS concentration for subsequent experiments based on the cell viability result.

### Lactate dehydrogenase release detection

A lactate dehydrogenase (LDH) cytotoxicity detection kit (C0016, Beyotime, China) was used to detect cytotoxicity based on LDH release. Cells (5 × 10^3^) were inoculated into 96-well plates and divided into the following groups: no cell culture medium (blank control), untreated cells (sample control), untreated cells for subsequent lysis (sample maximum enzyme activity control), and cells treated with different concentrations of TPA (0 nM, 10 nM, 20 nM, 40 nM, and 80 nM). All groups were treated for 24 h, with the sample maximum enzyme activity control group being added 10 μL of LDH release reagent and mixed 1 h in advance and then incubated as usual. Next, the supernatant from each well was transferred and mixed with 60 μL of LDH detection working solution, followed by incubation in the dark at room temperature for 30 min. Finally, the absorbance was measured at 490 nm using a microplate reader (Thermo Scientific, NY, USA). Cell Mortality (%) = (Absorbance of treated sample—Absorbance of sample control)/(Absorbance of maximum cell enzyme activity—Absorbance of sample control) × 100.

### Colony formation assay

The colony formation capacity of BC cells was evaluated using a colony formation assay. Ferristatin-1 (Fer-1, C_15_H_22_N_2_O_2_, HY-100579, purity: 99.86%, MCE, China) served as a ferroptosis inhibitor [6] and Vincristine (57-22-7, C_46_H_56_N_4_O_10_, purity ≥ 98%, Macklin, China) acted as a positive control for SS [2]. Briefly, the cells in the 6-well plates (1000 cells/well) containing 5 mL of culture medium under various conditions and cultured for 14 d. The control group received a complete culture medium. The concentration of Vincristine was 3 μg/mL. Then, colonies were stained and observed using a fluorescence microscope (WSF-1600, Guangzhou, China).

### Wound-healing assay

The effect of SS on the migratory capacity of BC cells was investigated by wound-healing assay. The cells were seeded into 6-well plates at 3 × 10^5^ cells/well density. Vertical scratches (200 µl sterile plastic pipette tip) were created, and then the debris was removed using phosphate buffer solution after the cells formed a fused monolayer. Then, different treatment conditions were applied, and cells were co-cultured in a serum-free medium for 24 h. Next, an inverted microscope was used to capture the images (Nikon Eclipse TE300, USA) at 0 and 24 h time points. ImageJ was used to calculate the wound area using formulas: the width of the scratch = Scratch area/Scratch length; the percentage of wound closure = (Scratch width at 0 h—Scratch width at 24 h)/(Scratch width at 0 h) × 100% [22].

### Transwell assay

The invasion capabilities of BC cells were evaluated using a Transwell chamber coated with an 8.0 μm Matrigel membrane (Corning, USA). Before seeding cells, each Transwell insert was incubated at 37 °C for 2 h for gel solidification. Treated cells were suspended in a serum-free medium and seeded onto the upper chamber at 1 × 10^4^ cells/insert density. A complete medium was added to the lower chamber. After 24 h, non-migrating cells on the upper side of the membrane were removed using cotton swabs. The cells on the underside of the membrane were fixed with 4% paraformaldehyde (PFA; Beyotime, China) and subsequently stained with 0.1% crystal violet solution (Beyotime, China). Next, we counted the stained cells using a microscope (WSF-1600, Guangzhou, China).

### Co-immunoprecipitation (Co-IP) assay

The Co-IP assay was used to determine whether SS affected the combination between MEK and ERK2. Treated cells (1 × 10^7^) were lysed, during which the natural structure of the proteins should be maintained to avoid denaturation. Then, magnetic beads coated with anti-p-ERK2 (Abcam, UK) or IgG (Abcam, UK) were incubated with the cell lysate at 4 °C overnight and then washed to precipitate the proteins interacting with p-ERK2. Next, we used SDS-PAGE and western blotting (WB) to detect the p-ERK2 binding proteins. The input group served as the positive control.

### Cell transfection

To verify the effects of SS on ERK2 and EGFR phosphorylation, we constructed the T185 phosphorylation site-mutant plasmid of ERK2 and Y1069 phosphorylation site-mutant plasmid of EGFR using pTracer-CMV2 (Thermo Scientific, NY, USA) as well as their corresponding overexpression plasmids. The primer sequences used for plasmids construction were synthesized by BGI Genomics (Shenzhen, China) and are as follows:

ERK2 overexpression primers

Forward: CGGGGTACCATGGCGGCGGCGGCGGCGGCGGGCGC;

Reverse: GCTCTAGATTAAGATCTGTATCCTGGCTGGAA.

ERK2 (T185I) mutation primers

Forward: ATATTCAATCAGGAACCCTGTGTGATC;

Reverse: GTTCCTGATTGAATATGTGGCCAC.

EGFR overexpression primers

Forward: CGGGGTACCATGCGACCCTCCGGGACGGCCGG;

Reverse: AAGGAAAAAAGCGGCCGCTCATGCTCCAATAAATTCACTGC.

EGFR (Y1069C) mutation primers

Forward: TTGCAGCGATGTAGCTCAGACCCCAC;

Reverse: TCTGAGCTACATCGCTGCAAGAAGCT.

Cells were transfected with plasmids at about 70%-80% confluency using Lipofectamine®2000 (Thermo Scientific, NY, USA) and harvested after 48 h.

### Tumor xenotransplantation model

All animal experiments were approved by the Ethics Committee of Shandong Cancer Hospital and Institute, Shandong First Medical University, and Shandong Academy of Medical Sciences (approval number: SDTHEC2024003176; approval date: March 1, 2024). The care, euthanasia and other procedures involving the animals followed the National Institutes of Health guidelines for the care and use of laboratory animals. 12-O-tetradecanoyl phorbol-13-acetate (TPA, 16,561–29-8, C_36_H_56_O_8_, purity: 99.80%), sourced from Merck (Milan, Italy), and Erastin (Era, HY-15763, C_30_H_31_ClN_4_O_4_, purity: 99.62%), purchased from MCE (New Jersey, USA) were used as ERK2/MAPK and ferroptosis activators, respectively. BALB/c nude mice (5–7 weeks old) (Harlan, Gannat, France) were housed in cages with filter tops and sterilized food [46]. 2 × 10^6^ MCF-7 cells were injected into the mice mammary fat pad. Once the tumors reached sizes between 50 and 100 mm^3^, we divided the mice into four groups: control, low-dose SS, high-dose SS, and Vincristine groups. The control group received DMSO only, while the low-dose SS (20 mg/kg) group and high-dose SS (40 mg/kg) group were given oral administration of SS twice during each treatment cycle [32]. Each treatment cycle lasted one week, and the experiment was continued for five weeks. The Vincristine group received five doses of Vincristine intraperitoneally (i.p.) every 4 d at each dose of 1 mg/kg [38]. The high-dose SS group was selected for subsequent experiments based on the tumor size inhibition. Then, we randomly divided the mice into six groups: control, SS, SS (Vehicle), SS + TPA, SS + Era and SS + TPA + Era. The control and SS groups were treated as previously described. The SS (Vehicle) group received DMSO, the SS + TPA group was treated with TPA (100 ng/g/day) [73], the SS + Era group received Era (15 mg/kg, i.p. injection, every other day) [6], and the SS + TPA + Era group was treated with both TPA and Era. Finally, the mice were euthanized using CO_2_, then the tumors were excised for subsequent analysis.

### Immunohistochemical (IHC) staining

After deparaffinization, hydration, and antigen retrieval, we incubated tumor sections with 5% bovine serum albumin (BSA; Solarbio, China) for 1 h. Then we treated sections with the primary antibodies p-ERK (ab201015, 1:100, Abcam) and Ki67 (ab197547, 1:200, Abcam) at 4 °C overnight [75]. Then, we incubated the sections with the secondary antibodies. Next, color development was achieved using DAB horseradish peroxidase (Beyotime, China), followed by hematoxylin counterstaining. The sections were sealed with neutral gum after dehydration. Finally, the positivity rate of the cells was analyzed using ImageJ 1.51 K software after observation under a microscope (WSF-1600, Guangzhou, China).

### Detection of glutathione peroxidase (GPX) activity

The glutathione peroxidase assay kit (S0056, Beyotime, China) was used to detect the activity of GPX. Cells (1 × 10^6^) or tissue (20 mg) samples were washed with PBS and then homogenized in sample homogenate on ice. Subsequently, centrifugation was performed at 4℃ and 12,000 g for 10 min to obtain the supernatant. According to the kit instructions, the detection buffer, sample, and GPX detection working solution were added sequentially and mixed thoroughly, followed by the addition of 40 µL of GPX detection working solution. The mixture was incubated at room temperature for 15 min to eliminate the interference of GSSG in subsequent detection. Then, 10 µL of 30 mM peroxide reagent solution was added to each well, mixed thoroughly, and immediately measured at A_340_ using a microplate reader (Thermo Scientific, NY, USA). This initial reading was recorded as 0 min. And the A_340_ value was recorded every 10 min for a total of five consecutive readings. [Glutathione Peroxidase Activity in the Detection System] = (ΔA_340_/min)/(ε^μM^ × L(cm)) = [(ΔA_340_ (sample)-ΔA_340_ (blank))/min]/ (ε^μM^ × L(cm)). [Glutathione Peroxidase Activity in the Sample] = [Glutathione Peroxidase Activity in the Detection System] × [dil × (V(mL)/V_sample_(mL))]/[Protein Concentration in the Sample].

### NADPH/NADP^+^ ratio detection

The NADPH/NADP^+^ ratio was measured using a NADP^+^/NADPH detection kit (S0179, Beyotime, China). Treated cells (1 × 10^6^) or tissues (10 mg) were fully lysed with 200 μl of NADP^+^/NADPH extraction buffer and then centrifuged at 12,000*g* and 4 °C for 10 min to obtain the supernatant. Then, 50 μL of supernatant from each group was incubated with 100 μL of G6PDH working solution in the dark at 37 °C for 10 min. Subsequently, the absorbance at 450 nm was measured using a microplate reader (Thermo Scientific, NY, USA) to evaluate the total concentration of NADP^+^ and NADPH (NADP_total_). In addition, the supernatant was heated (water bath) at 60℃ before being incubated with G6PDH working solution in the dark at 37℃ to detect the concentration of NADPH in the samples. [NADP^+^] = [NADP_total_]—[NADPH], [NADPH]/[NADP^+^] = [NADPH]/([NADP_total_]—[NADPH]).

### Measurement of GSH/GSSG ratio

A GSH and GSSG assay kit (Beyotime, China) was used to measure the GSH and glutathione disulfide (GSSG) levels in cells and tumor tissues. We treated cells (1 × 10^7^) or tumor tissues (0.1 g) with protein remover M solution and thoroughly homogenized. Then we centrifuged to obtain the supernatant solution. Next, 0.5 mg/mL NADPH solution was added after incubating 150 μL of total GSH assay solution and the supernatant at 25 °C for 5 min. The absorbance was immediately measured at 412 nm using a microplate reader (BioTek, Winooski, VT, USA). The concentration of total glutathione or GSSG was determined using a standard curve. The concentration of GSH was calculated using the formula: GSH level = total glutathione – (GSSG × 2).

### Iron assay

The iron assay kit (Abcam, UK) was used to detect iron levels. Briefly, iron assay buffer was added to the treated cells (1 × 10^6^) or tissues (10 mg), and the mixture was thoroughly homogenized. Then the supernatant was collected. Next, 50 μL of the supernatant was added to a 96-well plate and supplemented with iron assay buffer to 100 μL. In addition, we added 5 μL of assay buffer and 100 μL of the iron probe and then kept them in the dark. Finally, we used a microplate reader (Thermo Scientific, NY, USA) to measure the absorbance at 593 nm, and the iron concentration was quantified using a standard curve.

### Lipid reactive oxygen species (ROS) detection

C11-BODIPY 581/591 kit (Invitrogen, USA) was used to measure the lipid ROS levels. Treated cells (6-well plates at 3 × 10^5^ cells/well) or tumor tissue sections were incubated with 5 μM and 10 μM of the BODIPY probe at 37 °C for 30 min. The reduced BODIPY appeared red and the oxidized BODIPY was green. A microscope (Zeiss, Germany) was used to observe the fluorescence with excitation/emission wavelengths of 500/510 nm for green and 581/591 nm for red.

### TUNEL assay

Apoptotic cells were labeled with a TUNEL apoptosis detection kit (HY-K1078, MCE, China). 4% PFA (Beyotime, China) was used to treat cells (6-well plates at 3 × 10^5^ cells/well) for 30 min, while tissue samples were fixed for 60 min. The samples were then sequentially treated with 0.3% Triton X-100 at room temperature for 5 min, 50 µL TUNEL working solution (5 µL TdT enzyme and 45 µL FITC-12-dUTP labeling mix) at 37℃ in the dark. Finally, the samples were mounted with an anti-fluorescence quenching mounting medium, and TUNEL-positive cells were imaged using a fluorescence microscope (Zeiss, Germany).

### Flow cytometry

Treated cells (6-well plates at 3 × 10^5^ cells/well) were stained with Annexin V and PI (Vazyme, China) and analyzed using a flow cytometer (BeamCyte, China). Data acquisition was followed by analysis using CytoSYS 1.1 software.

### Western blotting

Total proteins from tumor tissues or cells were extracted using RIPA buffer supplemented with 100 mM PMSF (Beyotime, China). A BCA protein detection kit (Beyotime, China) was used to quantify the protein concentration. Subsequent steps included sample preparation, SDS-PAGE (10%), electrotransfer onto 0.45 mm PVDF membranes (Thermo Fisher, USA), then membrane blocking with BSA (Solarbio, China). The membranes were incubated with primary antibodies followed by horseradish peroxidase (HRP)-labeled secondary antibodies (1:2000; ZSGB-BIO, Beijing, China). Protein expression was quantified and imaged, and data were analyzed. GAPDH and β-actin served as internal controls. Primary antibodies were ERK2 (ab32081, 1:250, Abcam), p-ERK2 (pT185) (ab201015, 1:1000, Abcam), p-EGFR (pY1069) (SAB4504170, 1:500, Sigma) [53], SLC7A11 (ab307601, 1:1000, Abcam), Vimentin (ab92547, 1:5000, Abcam), GPX4 (ab219592, 1:1000, Abcam), MMP-2 (ab92536, 1:1000, Abcam), Ferritin (ab75973, 1:1000, Abcam), MMP-9 (ab58803, 1:500, Abcam), E-cadherin (ab231303, 1:1000, Abcam), p-MEK (sc-81503, 1:500, Santa Cruz), EGFR (ab52894, 1:1000, Abcam), MEK (sc-81504, 1:500, Santa Cruz), RAS (ab108602, 1:1000, Abcam), Ets-1 (ab307672, 1:500, Abcam), RAF (ab181115, 1:1000, Abcam), GAPDH (ab8245, 1:1000, Abcam), β-actin (ab8226, 1:500, Abcam).

### Statistical analysis

Data in this study were expressed as mean ± standard deviation (SD). GraphPad prism (Version 8.0; La Jolla, CA, USA) was used for graph plotting. Statistical differences between two independent groups were evaluated using Student’s t-test, while one-way ANOVA followed by Bonferroni post-hoc tests was used for comparisons among multiple groups. All experiments were performed at least three times. A p-value of less than 0.05 was considered statistically significant.

## Results

### Acquisition and pathway prediction of disease-drug intersection genes

**Fig. 1:**
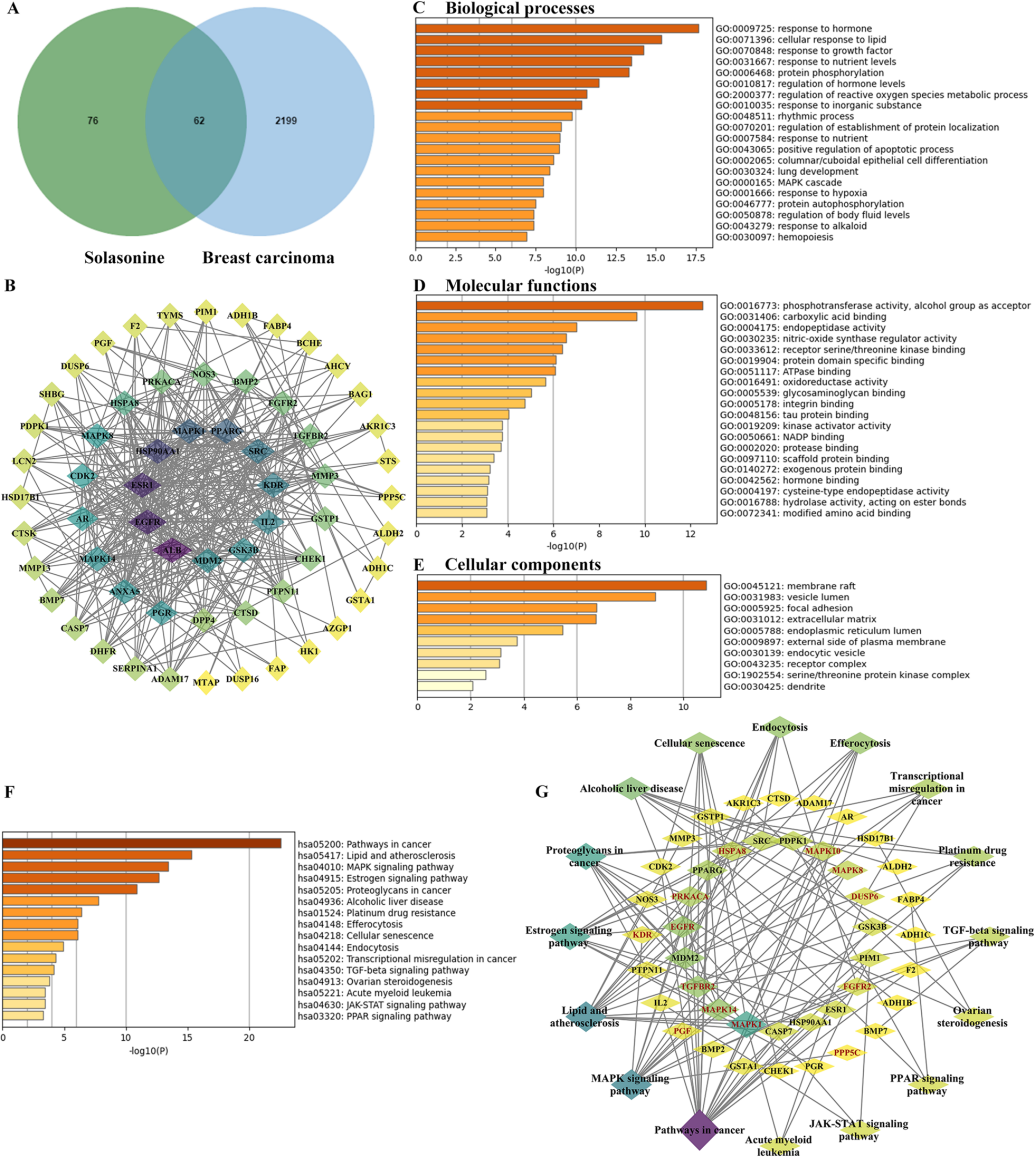
Network pharmacology and bioinformatics analysis for screening potential signaling pathways involved and the target genes for Solasonine (SS) treatment of breast carcinoma (BC). **A** SS and BC disease targets’ intersection genes were visualized through a Venn diagram and Cytoscape 3.10.1 (**B**). Gene Ontology (GO) functional enrichment analysis was performed for the intersection targets, including biological processes (**C**), molecular functions (**D**), and cellular components (**E**). **F** Kyoto Encyclopedia of Genes and Genomes (KEGG) enrichment pathways at the intersection of targets, along with their relationships, were visualized using Cytoscape 3.10.1 (**G**)

First, we utilized three databases—SwissTargetPrediction, PharmMapper, and the Similarity Ensemble approach—to predict the potential targets of SS. After deduplication, we identified a total of 138 potential targets for further analysis (Fig. 1A). Additionally, by searching databases including GeneCards, DisGeNET and the Therapeutic Target database, we obtained 2,261 genes associated with BC, excluding duplicates (Fig. 1A). Integrating the potential targets of SS with the BC-related genes revealed 62 overlapping genes, which might serve as the candidate targets for SS in treating BC (Fig. 1A). We then used STRING (confidence = 0.4) to filter out free nodes and employed Cytoscape software to construct a network map, resulting in a total of 61 target genes and 393 edges (Fig. 1B). Next, Metascape’s analysis found that among the intersection genes, the main biological processes predicted by GO analysis were response to hormones, cellular response to lipids, response to growth factors, response to nutrient levels, MAPK cascade etc. (Fig. 1C), the main molecular functions were phosphotransferase activity, alcohol group as acceptor, carboxylic acid binding, endopeptidase activity, etc. (Fig. 1D). Coincidentally, the MAPK pathway is reported to be a phosphorylation cascade [5, 62]. Besides, the main cellular components were membrane raft, vesicle lumen, focal adhesion, etc. (Fig. 1E). In addition, KEGG predicted that the mechanism of SS treatment of BC may be mainly related to MAPK signaling pathway (Fig. 1F, G). The most important gene enriched in the MAPK pathway (marked in red), as well as among the enriched pathways, was MAPK1 (ERK2) (Fig. 1G). Notably, MAPK1 was identified as a key gene among the overlapping genes associated with SS and BC (Fig. 1B). Thus, we speculated that the SS treatment of BC may be related to the ERK2/MAPK pathway.

### ERK2 expression in clinical BC patients

**Fig. 2:**
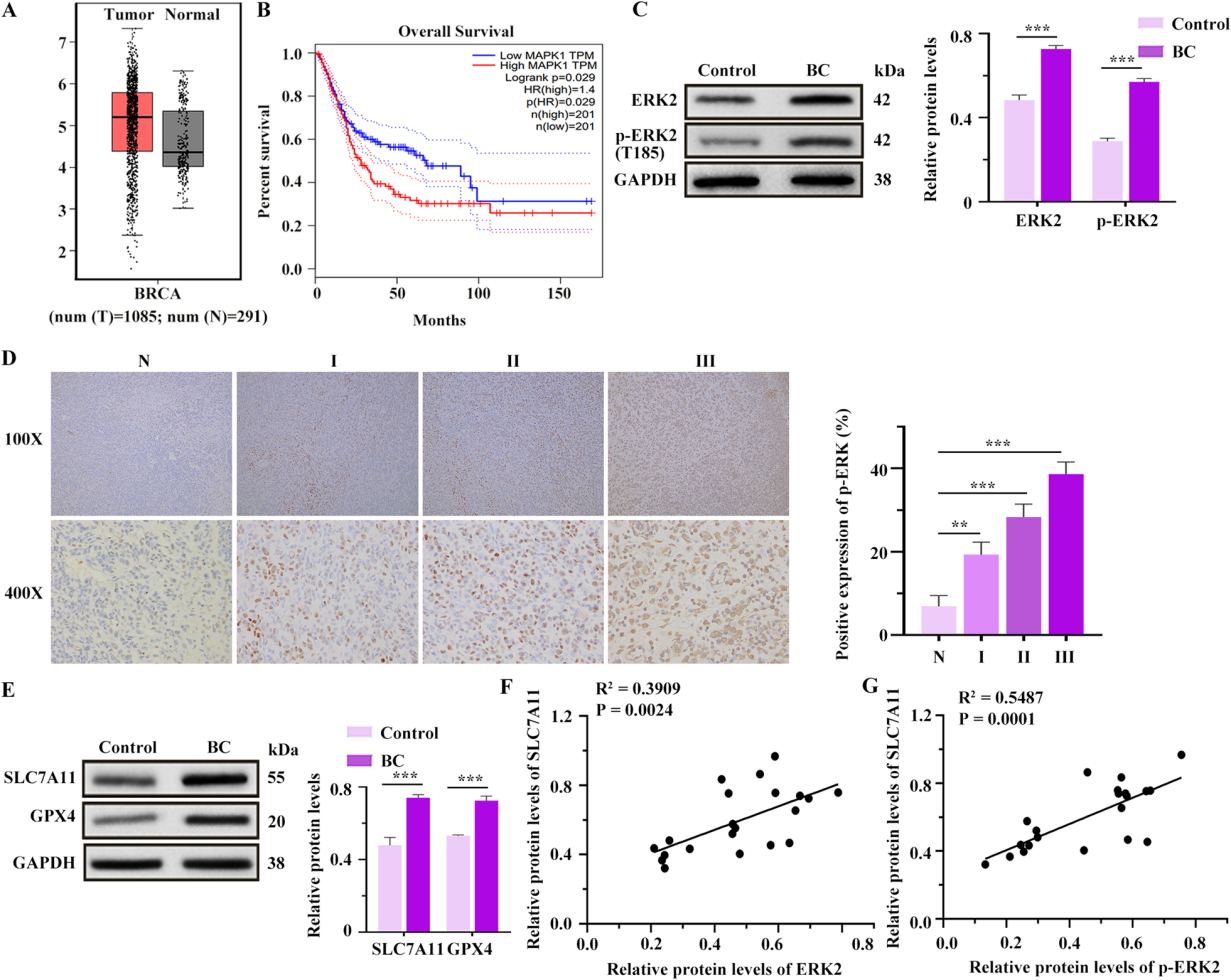
Expressions of extracellular signal-regulated kinase 2 (ERK2) and its phosphorylated form p-ERK2 in clinical BC patients and the relationship between p-ERK2 and ferroptosis. **A** The expression of ERK2 in normal and BC patients. **B** The survival time of BC patients with a high or low level of ERK2 expression. **C** The expressions of ERK2 and p-ERK2 in BC tissues (n = 7) were detected by western blotting (WB). **D** P-ERK immunohistochemical (IHC) staining in tumor tissues of BC patients and its quantitation. **E** The expressions of solute carrier family 7 member 11 (SLC7A11) and glutathione peroxidase 4 (GPX4) in BC tissues (n = 7) were detected by WB. **F** The correlation analysis of ERK2 and SLC7A11, n = 7. **G** The correlation analysis of p-ERK2 and SLC7A11, n = 7. **p < 0.01, ***p < 0.001

According to the Gene Expression Profiling Interactive Analysis 2 (GEPIA) database (http://gepia2.cancer-pku.cn/#index), ERK2 expression in BC patients was significantly up-regulated compared with normal, and elevated ERK2 levels were associated with poorer overall survival in BC patients (Fig. 2A, B). In this study, WB analysis confirmed that compared with the control group, ERK2 and p-ERK2 levels were significantly higher in the BC group (Fig. 2C). IHC staining further revealed that p-ERK2 expression was up-regulated in BC tissues compared with normal breast tissues, with positive expression rates of p-ERK2 being higher in stage II and III patients than that in stage I patients (Fig. 2D). Additionally, WB results showed that compared with the control group, SLC7A11 and GPX4 levels in the BC group were also elevated (Fig. 2E). Importantly, SLC7A11 expression was positively correlated with both ERK2 and p-ERK2 levels (Fig. 2F, G), suggesting that ERK2 may be a negative regulator of ferroptosis in BC.

### SS inhibits the proliferation, migration, and invasion of BC cells through ferroptosis

**Fig. 3:**
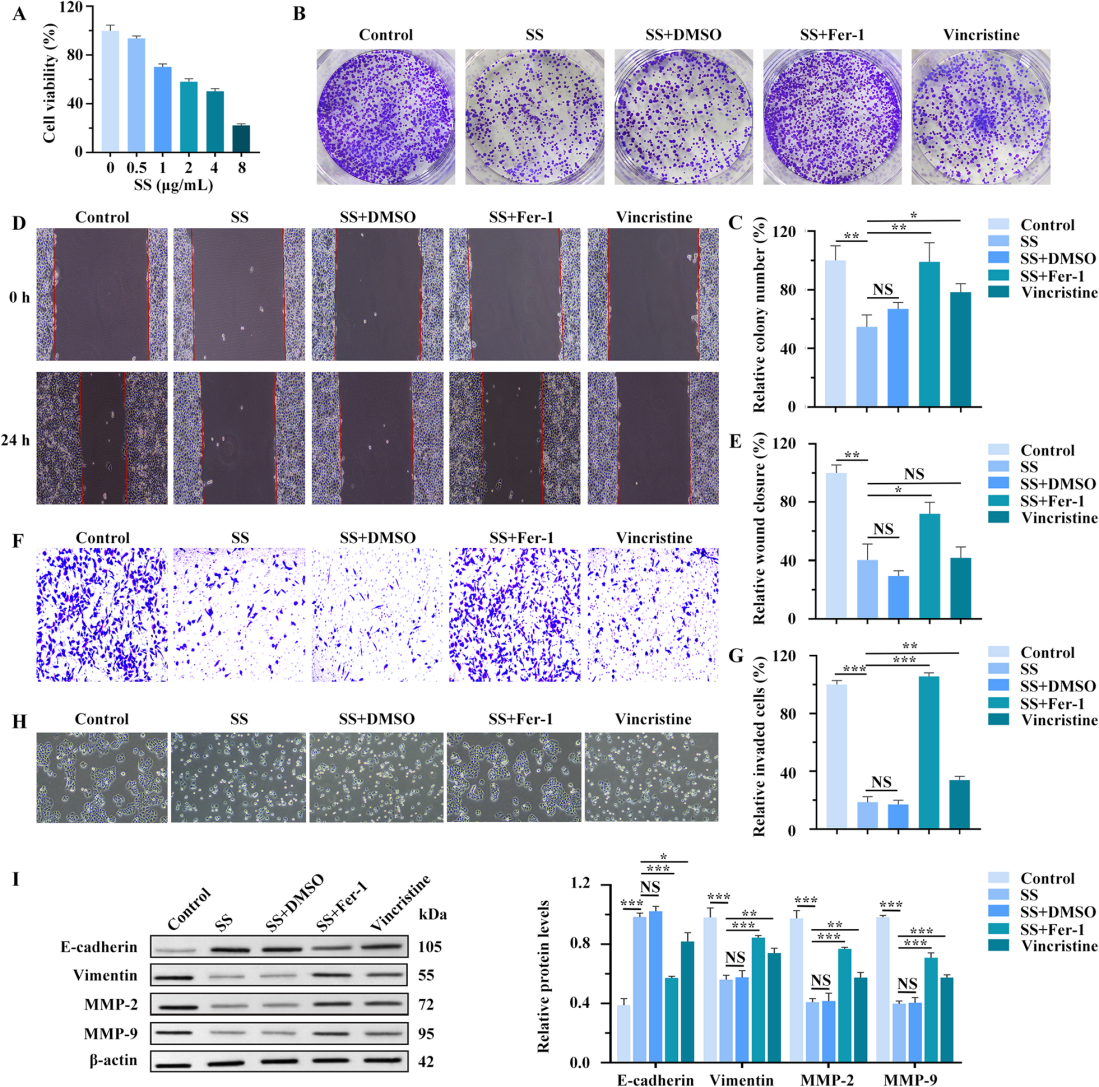
SS inhibits the proliferation, migration, and invasion of MCF-7 cells through ferroptosis. **A** CCK8 assay. **B** Clone formation assay and its quantitation (**C**). **D** Wound-healing assay and its quantitation (**E**). Transwell matrigel invasion assay (**F**) and its quantitation (**G**). **H** The morphological changes in MCF-7 cells of each group were observed by microscope. **I** The expression levels of epithelial-mesenchymal transition (EMT)-related factors detected by WB. *p < 0.05, **p < 0.01, ***p < 0.001

To investigate the effects of SS on BC cells, we used various concentrations of SS to treat MCF-7 and MDA-MB-231 cells. The CCK-8 assay demonstrated that SS treatment was cytotoxic to both cell lines (Fig. 3A and Fig. S_1_A). IC_50_ values of MCF-7 and MDA-MB-231 cells were 2.92 µg/mL and 3.87 µg/mL respectively. Based on the IC_50_ value, we selected 3 µg/mL as the experimental concentration of SS for subsequent experiments. To explore whether ferroptosis is involved in the effect of BC by SS, we added Fer-1 and assessed its impact on the proliferation, migration, and invasion of cells. The cell cloning assays revealed that the number of colonies of MCF-7 and MDA-MB-231 cells decreased significantly after SS treatment compared with the control group, suggesting that SS inhibits BC cell proliferation. Notably, the addition of Fer-1 salvaged the SS’s inhibition of cell proliferation (Fig. 3B, C and Fig. S_1_B, C). What’s more, wound-healing and Transwell assays yielded similar results, confirming the findings from the cell cloning assays (Fig. 3D-G and Fig. S_1_D-G). As a key regulatory event in tumor cell invasion, we observed the morphological changes of cells in each group by microscopy and examined markers of epithelial-mesenchymal transition (EMT) through WB analysis. Morphological analysis showed that MCF-7 and MDA-MB-231 cells mostly exhibited the spindle-shaped or slender fusiform morphology characteristic of mesenchymal cells. Cells treated with SS were more likely to be spherical or cobblestone-like, while Fer-1 weakened the effect of SS (Fig. 3H and Fig. S_1_H). The WB results showed that E-cadherin was up-regulated while Vimentin, MMP-9, and MMP-2 were downregulated after SS treatment, suggesting that SS suppresses the EMT process in BC cells (Fig. 3I and Fig. S_1_I). When treated with Fer-1, the expression of EMT-associated genes was reversed compared to the SS treatment alone. Moreover, SS demonstrated superior efficacy compared to the positive control drug in inhibiting BC cell progression. It indicates that the ferroptosis process may regulate SS on proliferation, migration, and invasion of BC cells.

To further verify whether SS exerts inhibitory effects on BC cells by regulating ferroptosis, the levels of ferroptosis in BC cells treated with SS were assessed. We found that ferroptosis was induced in SS‐treated BC cells, as evidenced by decreased protein levels of SLC7A11, ferritin and GPX4, GPX activity, NADPH/NADP^+^, as well as GSH/GSSG ratio, accompanied by increased Fe^2+^ and lipid ROS levels. In addition, the promoting effect of SS on ferroptosis was counteracted by using Fer-1 (Fig. S_2_A-G). Furthermore, TUNEL assays and flow cytometry revealed that compared with the control group, the apoptotic cells in the SS-treated group were significantly increased, and this pro-apoptotic effect was also rescued by Fer-1 (Fig. S_3_A, B). Thus, we speculate that SS promotes apoptosis of BC cells by activating the ferroptosis process.

### SS modulates the activity of the ERK2/MAPK pathway by binding directly to ERK2 or epidermal growth factor receptor (EGFR)

**Fig. 4:**
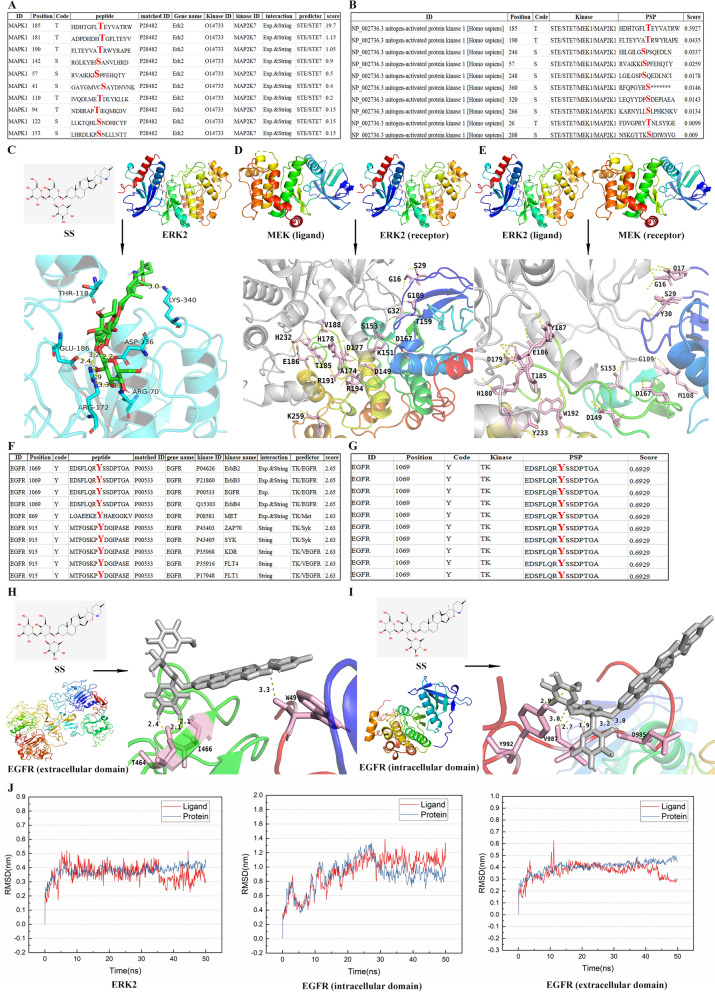
SS regulates the activity of the ERK2/mitogen-activated protein kinase (MAPK) pathway by binding to ERK2 or epidermal growth factor receptor (EGFR). **A** Prediction of ERK2 phosphorylation sites by iGPS1.0 and GPS6.0 (**B**). **C** Molecular docking results of SS with ERK2. **D** Docking results between mitogen-activated extracellular signal-regulated kinase (MEK) (ligand) and ERK2 (receptor) proteins. Gray, MEK; Iridescent, ERK2. **E** Docking results between ERK2 (ligand) and MEK (receptor) proteins. Gray, MEK; Iridescent, ERK2. **F** Prediction of EGFR phosphorylation sites by iGPS1.0 and GPS6.0 (**G**). **H** The results of SS docking with the extracellular domain and intracellular domain (**I**) of EGFR. **J** The kinetic assay of SS with ERK2 and EGFR

The phosphorylation of ERK facilitates its dissociation from MEK, a crucial step for its entry into the nucleus [7]. Reports indicate that human ERK2 can be phosphorylated at several sites, including Thr185, Tyr187, Ser246, and Ser244 [3, 41, 43]. The ERK2 phosphorylation sites were predicted using iGPS1.0 (http://igps.biocuckoo.org/index.php) and GPS6.0 (http://gps.biocuckoo.cn/index.php), identifying Thr185 as a highly predominant phosphorylation site by MEK (Fig. 4A, B). We conducted molecular docking studies to determine whether SS affects the phosphorylation of ERK2 at Thr185 residue. The SS structure was obtained from TCMSP, while ERK2 was sourced from PDB, as detailed in the materials and methods section. It indicated that the interaction between SS and ERK2 protein is primarily facilitated by hydrogen bonding (Arg70, Arg172, Glu186, Asp336, Lys340) and hydrophobic interaction (Thr118) to promote the formation of stable complexes (affinity =  − 11.5 ± 0.46 kcal/mol, Ki = 8.53 ± 1.07 μM) (Fig. 4C). Therefore, we speculated that the binding of SS to ERK2 (residues 70-340, including T185) may interfere with the phosphorylation of Thr185 due to covering the Thr185 site, thereby affecting the phosphorylation of ERK2.

To further assess whether SS influences the phosphorylation activity of ERK2, we performed molecular docking between MEK and ERK2. The findings suggested that the binding of SS to ERK2 may impede the interaction between MEK and ERK2, thereby affecting the phosphorylation of ERK2 by MEK (Fig. 4D, E). This is likely due to the overlap between the binding sites of SS and ERK2 (residues 70-340, including T185) and the region where MEK interacts with ERK2 (residues 10–250, including T185).

EGFR has been reported as a target in treating BC [37]. Dong et al. have reported that SS can inhibit EGFR expression by binding to NRP1, subsequently affecting the ERK/MAPK pathway [16]. Upon activation, EGFR undergoes autophosphorylation at the carboxyl-terminal, including sites such as Y1069, Y1092, and Y1197 in humans [9, 21, 69]. Notably, phosphorylation at Y1069 occurs during EGFR dimerization and is a critical site for EGFR signaling [49]. In this study, we found that EGFR is one of the key genes in the MAPK enrichment pathway and predicted the phosphorylation site at Y1069 of EGFR using GPS6.0 and iGPS1.0 (Fig. 4F, G). Then, we also conducted molecular docking of SS with EGFR, revealing that SS forms hydrogen bonds with multiple residues in both the extracellular domain (W49, T464, I466) (affinity =  − 8.7 ± 0.57 kcal/mol, Ki = 12.7 ± 0.50 μM) and intracellular domain (D985, V987, Y992) (affinity =  − 10.7 ± 0.25 kcal/mol, Ki = 9.57 ± 1.21 μM) (Fig. 4H, I). This suggests that the binding of SS may influence its phosphorylation and subsequent intracellular activation. The results of the kinetic assay further confirmed the molecular docking results that SS regulates the ERK2/MAPK signaling pathway mainly by affecting the phosphorylation of ERK2 and EGFR (Fig. 4J). In summary, SS has the potential to bind to ERK2 or EGFR which affects the phosphorylation activity of ERK2 T185 and EGFR Y1069, thereby modulating the activity of the ERK2/MAPK pathway.

### SS inhibits the ERK2/MAPK signaling pathway to activate ferroptosis and thereby prevent the proliferation, migration, and invasion of BC cells

**Fig. 5:**
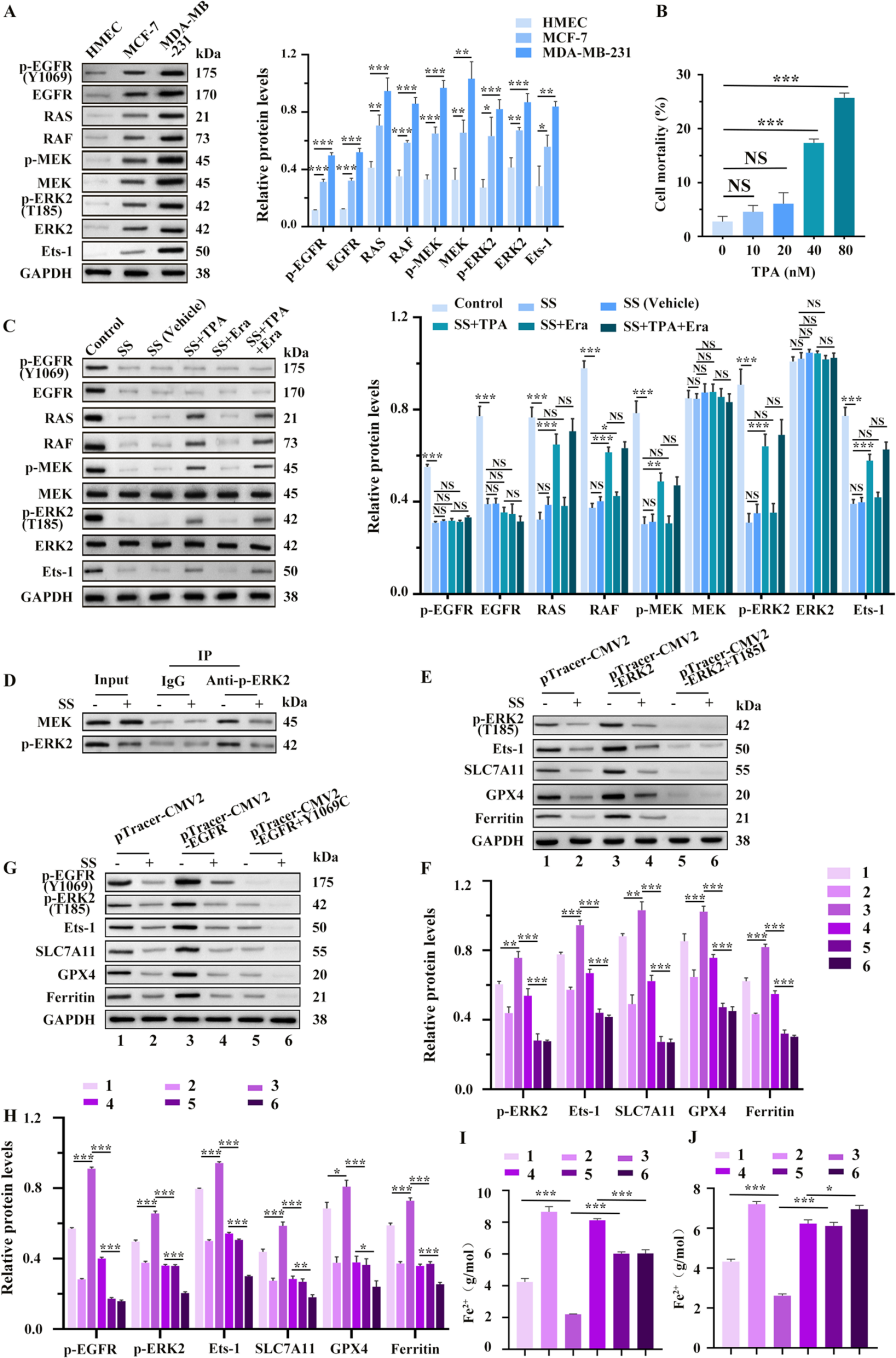
SS inhibits the ERK2/MAPK signaling pathway in BC cells. **A** WB analysis of the ERK2/MAPK pathway factors in MCF-7, MDA-MB-231, and HMEC cell lines. **B** Extracellular lactate dehydrogenase (LDH) release was detected by the LDH cytotoxicity detection kit. **C** The expressions of ERK2/MAPK signaling pathway-related factors in MCF-7 cells treated with SS were analyzed by WB and its quantitation. **D** Co-IP assay verified that SS inhibited the binding between MEK and ERK2 in MDA-MB-231 cells. (**E**) WB analysis of ERK2/MAPK signaling pathway and ferroptosis-related factors expression in MDA-MB-231 cells with ERK2 T185I phosphorylation site mutation treated with SS and its quantitation (**F**). Group: 1, pTracer-CMV2 (SS−); 2, pTracer-CMV2 (SS +); 3, pTracer-CMV2-ERK2 (SS-); 4, pTracer-CMV2-ERK2 (SS +), 5, pTracer-CMV2-ERK2 + T185I (SS−); 6, pTracer-CMV2-ERK2 + T185I (SS +). (**G**) WB analysis of ERK2/MAPK signaling pathway and ferroptosis-related factors expression in MDA-MB-231 cells with EGFR Y1069C phosphorylation site mutation treated with SS and its quantitation (**H**). Group: 1, pTracer-CMV2 (SS-); 2, pTracer-CMV2 (SS +); 3, pTracer-CMV2-EGFR (SS-); 4, pTracer-CMV2-EGFR (SS +), 5, pTracer-CMV2-EGFR + Y1069C (SS-); 6, pTracer-CMV2- EGFR + Y1069C (SS +). (**I**) Iron levels were detected using the iron assay kit. Group: 1, pTracer-CMV2 (SS-); 2, pTracer-CMV2 (SS +); 3, pTracer-CMV2-ERK2 (SS-); 4, pTracer-CMV2-ERK2 (SS +), 5, pTracer-CMV2-ERK2 + T185I (SS−); 6, pTracer-CMV2-ERK2 + T185I (SS +). **J** Iron levels were detected using the iron assay kit. Group: 1, pTracer-CMV2 (SS−); 2, pTracer-CMV2 (SS +); 3, pTracer-CMV2-EGFR (SS-); 4, pTracer-CMV2-EGFR (SS +), 5, pTracer-CMV2-EGFR + Y1069C (SS−); 6, pTracer-CMV2- EGFR + Y1069C (SS +). *p < 0.05, **p < 0.01, ***p < 0.001

**Fig. 6:**
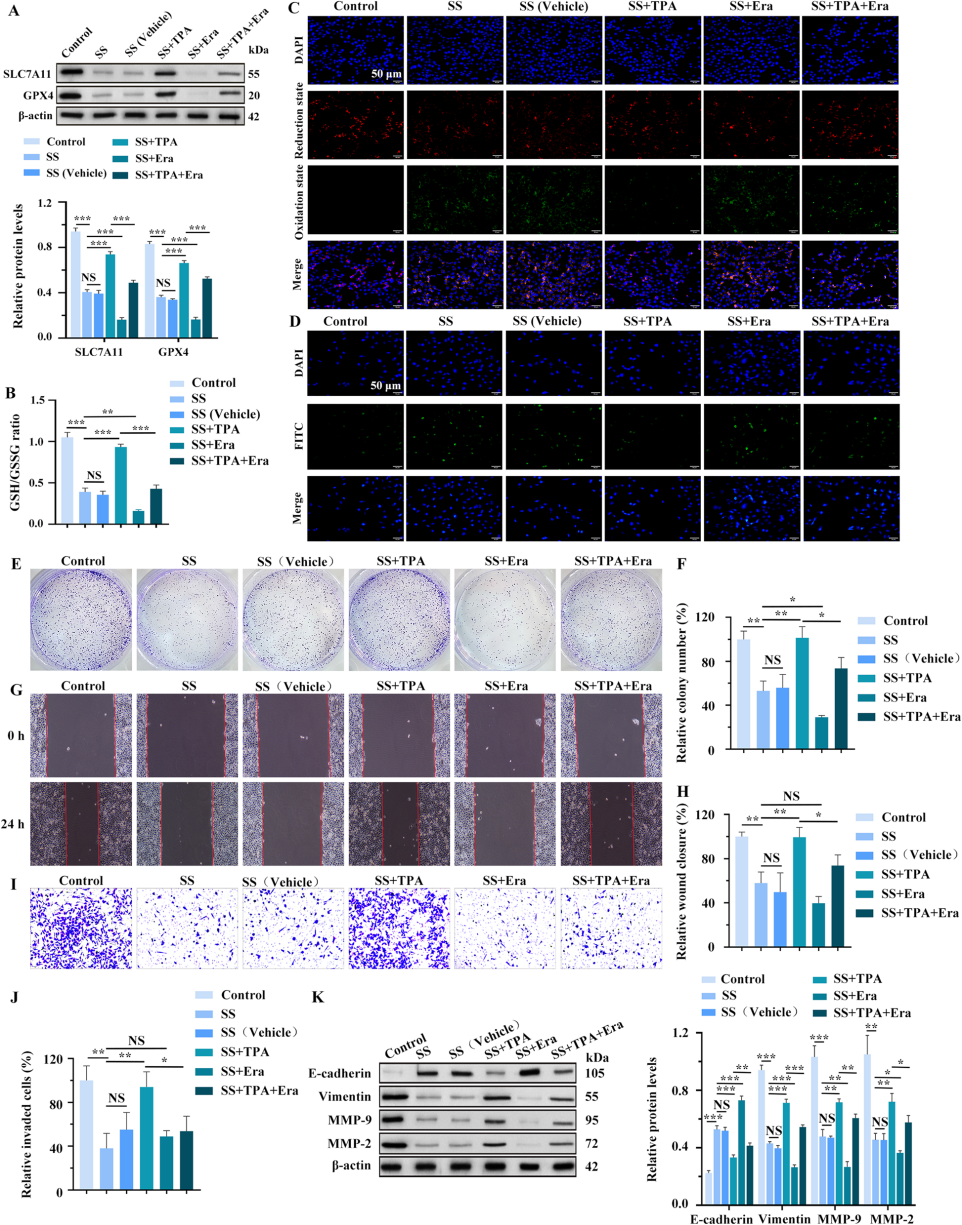
SS activates ferroptosis by suppressing the ERK2/MAPK signaling pathway to inhibit the proliferation, migration, and invasion of MCF-7 cells. **A** The expressions of SLC7A11 and GPX4 were detected by WB. **B** Measure the GSH/GSSG ratio using the GSH and GSSG assay kit. **C** Detection of lipid ROS levels using C11-BODIPY 581/591 kit. **D** TUNEL assay. **E** Clone formation assay and its quantitation (**F**). **G** Wound-healing assay and its quantitation (**H**). **I** Transwell matrigel invasion assay and its quantitation graph (**J**). **K** The expression levels of EMT-related factors were detected by WB. *p < 0.05, **p < 0.01, ***p < 0.001

To investigate whether SS regulates ferroptosis through the ERK/MAPK signaling pathway and subsequently affects apoptosis in BC cells, we first assessed the expression of key factors associated with this pathway. WB analysis revealed that components of the ERK2/MAPK signaling pathway—specifically RAS, RAF, p-MEK, MEK, p-ERK2 and ERK2—along with the upstream factor EGFR and the downstream substrate E-twenty-six-1 (Ets-1), were significantly up-regulated in BC cells than in HMEC cells. This indicates the ERK2/MAPK signaling pathway was activated in BC cells (Fig. 5A). Then, we used TPA as the activator of the MAPK signaling pathway. The results of the LDH release detection showed that 20 nM of TPA did not exhibit significant cytotoxicity towards BC cells (Fig. 5B and Fig. S_5_A). We chose 20 nM as the final concentration of TPA for the subsequent experiments. Following SS treatment, the expression levels of these factors (EGFR, p-EGFR, RAS, RAF, p-MEK, p-ERK2, Ets-1) were significantly reduced in BC cells compared to untreated controls (Fig. 5C and Fig. S_5_B). It indicates that the inhibitory effect of SS on ERK2/MAPK pathway factors (RAS, RAF, p-MEK, p-ERK2) and Ets-1 was partially reversed by TPA (Fig. 5C and Fig. S_5_B). Co-IP results showed that the expression level of p-ERK2 (T185) was significantly down-regulated after SS treatment, suggesting that SS might affect ERK2/MAPK signaling pathway by affecting the binding of MEK to ERK2 and thus the phosphorylation of ERK2 (Fig. 5D). Then, in order to further verify whether SS regulates the ERK2/MAPK pathway and ferroptosis by affecting the T185 phosphorylation site of ERK2 or the Y1069 phosphorylation site of EGFR, we constructed mutations at the T185 phosphorylation site of ERK2 (pTracer-CMV2-ERK2 + T185I) and the Y1069 phosphorylation site of EGFR (pTracer-CMV2-EGFR Y1069C) as well as their corresponding overexpressed plasmids. Results showed that a significant decrease of p-EGFR (Y1069), p-ERK2 (T185), Ets-1, SLC7A11, GPX4, and Ferritin in MDA-MB-231 cells treated with SS, suggesting that SS may affect ferroptosis by influencing the phosphorylation of ERK2 through p-EGFR (Y1069) or p-ERK2 (T185), which were opposite to the results of their overexpressing plasmids (Fig. 5E-J). The results in MCF-7 cells were similar to those of MDA-MB-231 cells, and we did not present them here. Thus, the above results preliminarily indicated that SS can influence ferroptosis by affecting the activity of the ERK2/MAPK pathway, which was further confirmed by using the ERK2/MAPK activator TPA and the ferroptosis activator Era. We found that TPA mitigated the activation of ferroptosis induced by SS. In contrast, the combination of SS and Era enhanced the ferroptotic effect in BC cells (SS + Era group) and partially counteracted TPA’s effects (SS + TPA + Era group) (Fig. 6A-C, Fig. S_4_A-D and Fig. S_5_C-I). Moreover, TUNEL assay and flow cytometry results showed that compared with the control group, there was a significant increase in apoptosis rates of BC cells following SS treatment, a response that was partially counteracted by TPA. The addition of Era further amplified the apoptotic effects in the SS + Era group, which partially countered TPA’s inhibitory effect on apoptosis (Fig. 6D, Fig. S_4_E and Fig. S_5_J, K). Thus, the results suggest that SS can effectively activate ferroptosis and promote BC cell death by inhibiting the ERK2/MAPK pathway.

Cell colony formation, wound-healing, and transwell assays were first performed to explore whether SS could regulate ferroptosis and subsequently affect progression in BC cells. Our findings demonstrated that SS treatment significantly reduced colony formation, wound closure, and invasion of BC cells compared with the control group. Notably, these effects could be partially reversed by TPA, while Era also partially counteracted TPA’s impact (Fig. 6E-J and Fig. S_6_A-F). WB analysis revealed that in the SS-treated group, the mesenchymal markers MMP-9, Vimentin, and MMP-2 were downregulated, while the epithelial marker E-cadherin was up-regulated across two BC cell lines. However, these expression patterns were reversed when BC cells were treated with both SS and TPA. Furthermore, Era was shown to partially neutralize the expression of these factors in the SS + TPA group (Fig. 6K and Fig. S_6_G). It suggests that SS inhibits the ERK2/MAPK signaling pathway, activating ferroptosis, thereby suppressing the proliferation, migration, and invasion of BC cells.

### SS inactivates the ERK2/MAPK pathway to induce ferroptosis and inhibits the growth and proliferation of BC in vivo

**Fig. 7:**
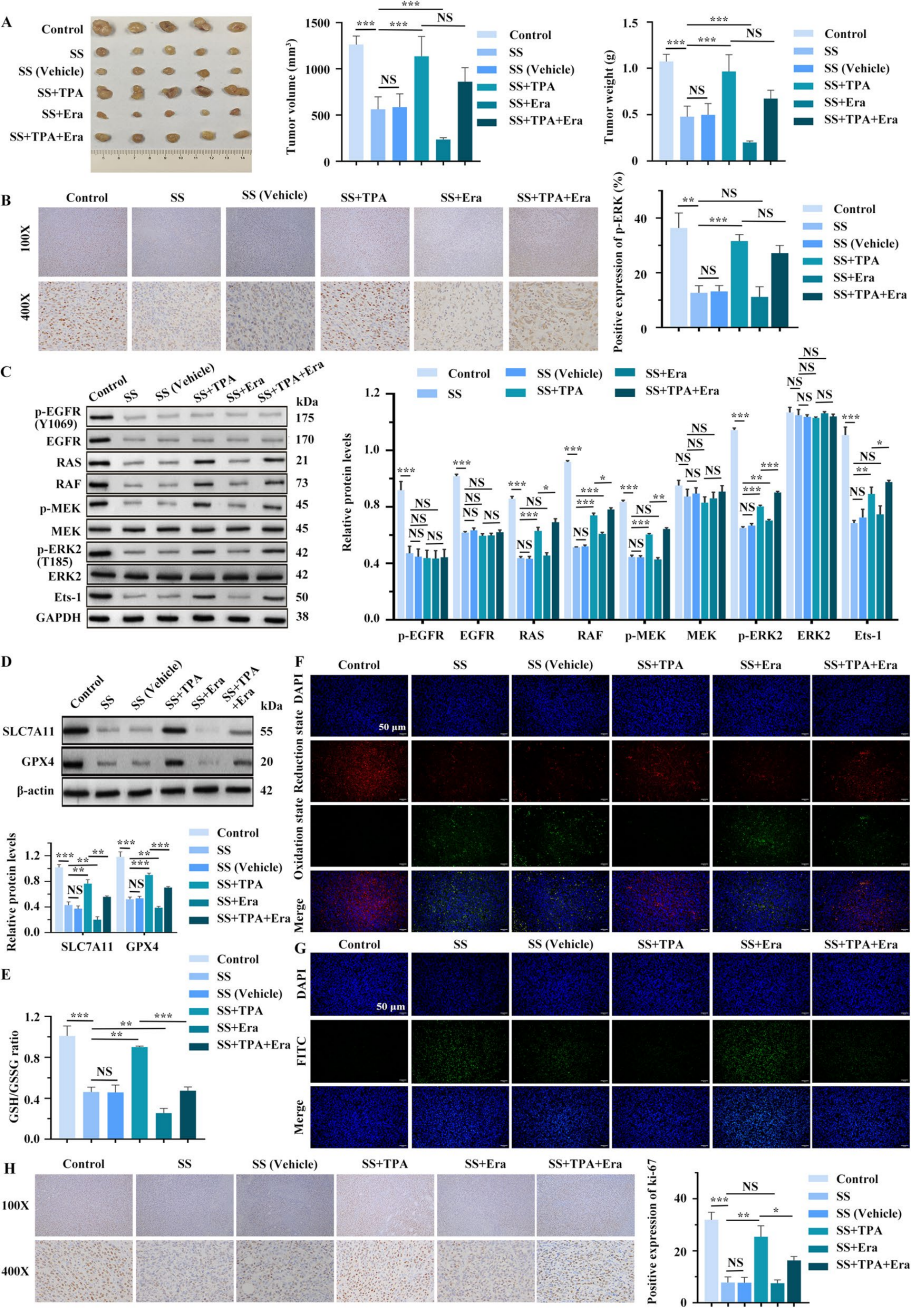
SS induces ferroptosis by inhibiting the ERK2/MAPK signaling pathway to regulate the development of BC. **A** Inhibitory effect of high-dose SS on tumor size, volume, and weight (n = 5). **B** IHC analysis of p-ERK expression in tumor tissues. **C** The expressions of ERK2/MAPK signaling pathway-related factors were detected by WB (n = 5). **D** The expression levels of SLC7A11 and GPX4 were detected by WB (n = 5). **E** Measurement of GSH/GSSG ratio by GSH and GSSG assay kit (n = 5). **F** Detection of lipid ROS using C11-BODIPY 581/591 kit. **G** TUNEL assay. **H** IHC analysis of Ki-67 expression. *p < 0.05, **p < 0.01, ***p < 0.001

To further confirm the anti-cancer effect of SS, we utilized an MCF-7 cell xenografted nude mice model. Our results indicated that the high-dose SS group exhibited a more pronounced inhibitory effect on tumor size, volume, and weight than the low-dose group and the positive control drug vincristine (Fig. S_7_A). Therefore, we selected the high-dose SS for the subsequent experiments. In comparison to the control group, the mean tumor volume and weight in the SS group were decreased; however, no significant changes were noted in the SS + TPA group. The most significant diminution in tumor volume occurred in the SS + Era group, where Era was shown to counteract TPA’s effects (SS + TPA + Era vs. SS + TPA) partially (Fig. 7A). Immunohistochemical staining revealed that SS treatment significantly reduced p-ERK positive expression compared to the control group, while TPA reversed this trend (Fig. 7B). Additionally, WB analysis revealed decreased expression levels of markers associated with ERK2/MAPK signaling pathway in the SS group but increased with TPA treatment (Fig. 7C). These findings suggest that SS effectively inactivates the ERK2/MAPK signaling pathway in vivo*.*

Next, to investigate whether SS influences BC ferroptosis through the ERK2/MAPK pathway, we conducted rescue experiments using both the ERK/MAPK pathway activator (TPA) and the ferroptosis activator (Era). Results showed that the protein levels of SLC7A11, GPX4 and ferritin, GPX activity, NADPH/NADP^+^, and GSH/GSSG ratio were downregulated in the SS group, while Fe^2^⁺ and lipid ROS levels increased, indicating that SS activated ferroptosis. When SS and TPA were administered together, the expression trends of ferroptosis-related indicators were reversed compared with SS treatment. Furthermore, adding Era alongside TPA partially salvaged TPA’s ferroptosis-inhibiting effects (Fig. 7D-F and Fig. S_7_B-E). In addition, TUNEL assays revealed a significant increase in apoptotic cells following SS treatment compared to the control group, with TPA partially reversing this effect. Notably, the use of Era in the SS + Era group enhanced apoptosis compared to the SS group, while Era also partially neutralized TPA’s inhibitory effect on apoptosis in the SS + TPA group, suggesting that SS promotes ferroptosis in BC by inhibiting the ERK2/MAPK signaling pathway (Fig. 7G).

IHC staining showed a significant reduction in Ki-67 expression in tumor tissues following SS treatment, which TPA could partially restore. Additionally, Era enhanced the proliferation inhibition of SS and partially counteracted TPA’s effects (Fig. 7H). Collectively, these data indicate that SS inhibits the ERK2/MAPK signaling pathway, induces ferroptosis, and consequently suppresses BC.

## Discussion

**Fig. 8:**
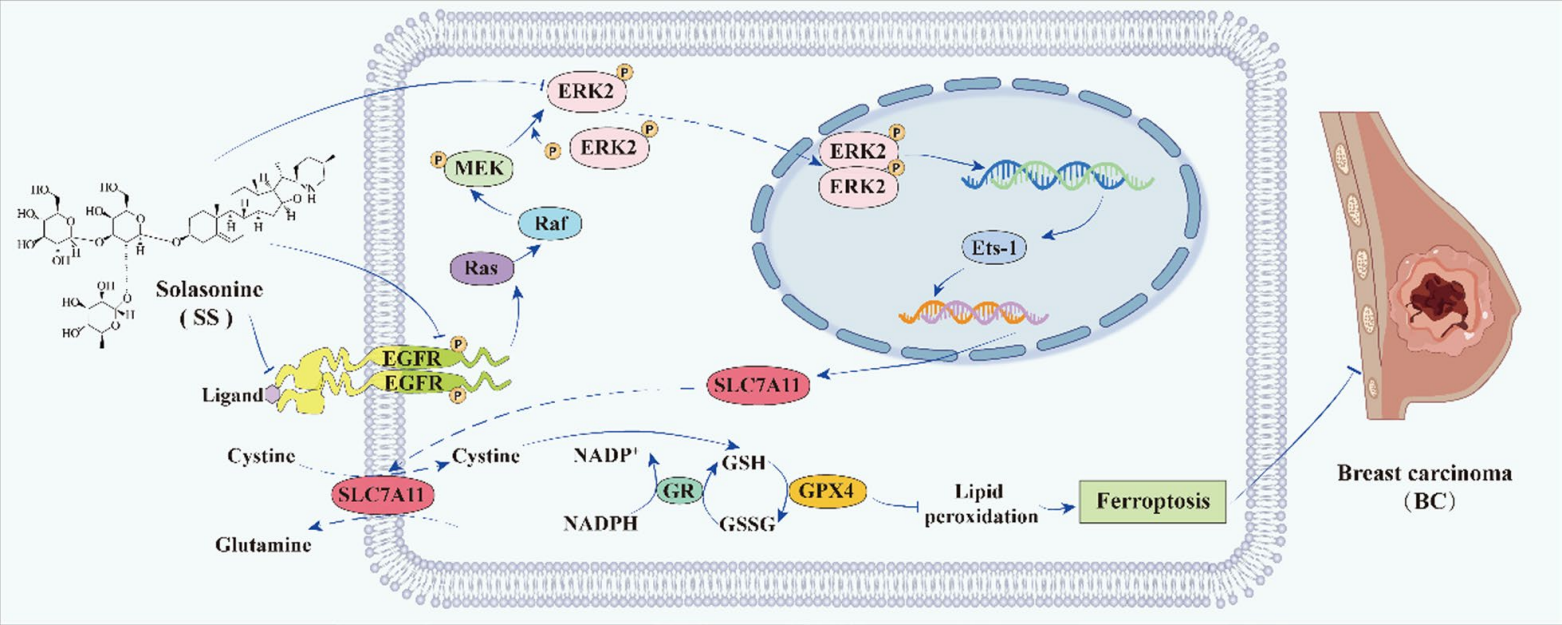
Graphical abstract illustrating the mechanism of SS in treating BC. The SS directly binds to EGFR and ERK2, which may affect the extracellular binding of ligands to EGFR, intracellular phosphorylation of EGFR, phosphorylation of ERK2 by MEK, formation and nuclear translocation of ERK2 dimers to inactivate the ERK2/MAPK signaling pathway, thereby influencing the expression of the transcription factor E-twenty-six-1 (Ets-1) for SLC7A11, resulting in down-regulation of SLC7A11 expression. This subsequently reduces the GSH/GSSG ratio and GPX4 enzyme activity, leading to an increase in lipid peroxidation accumulation, ultimately promoting ferroptosis, and inhibiting BC progression

As the most common malignancy in women globally, BC is commonly treated with conventional surgery combined with chemotherapy. Unfortunately, these treatments often fail due to drug resistance and frequent recurrences [20, 47, 66]. Here, we show that SS can activate the ferroptosis process by inactivating the ERK2/MAPK signaling pathway, thereby contributing to BC treatment (Fig. 8).

Numerous traditional Chinese medicines have shown promise in treating BC. For example, it has been reported that Anomanolide C can inhibit the progression of TNBC cells, while Escin can suppress BC tumor growth [13, 30]. SS has emerged as a potential tumor suppressor in various cancers, including pancreatic cancer, lung adenocarcinoma, and BC [1, 32, 68]. Besides, Akula et al. have reported that SS can inhibit the proliferation and colony-forming ability of MCF-7 cells upon MED12 knockdown [1]. Here, we demonstrate that SS treatment inhibits the proliferation, migration, and invasion of BC cells, outperforming the positive control drug Vincristine. Additionally, the SS treatment reduces the progression of xenograft tumors in vivo. EMT is crucial in tumor cell migration and invasion [50, 67]. Extracellular matrix (ECM) degradation is closely related to tumor migration and invasion, and matrix metalloproteinases (MMP-9, MMP-2) facilitate this process [40]. In this study, SS resulted in the downregulation of MMP-9 and MMP-2, along with the downregulation of Vimentin and the up-regulation of E-cadherin. This suggests that the decreased MMP-9 and MMP-2 may inhibit ECM degradation, making it more challenging for cancer cells to breach the basement membrane, thereby reducing their migration and invasion potential.

Ferroptosis is characterized by impaired synthesis of antioxidant and free radical scavenger GSH, and it is a new form of programmed cell death distinct from apoptosis [36, 58]. System xc^−^ has been reported to maintain GSH homeostasis, and its inhibition disrupts GSH synthesis, decreases GPX4 activity, and ultimately leads to ferroptosis through lipid ROS accumulation [10, 58]. Activation of ferroptosis has been suggested as a beneficial strategy for treating BC [66]. SS activates ferroptosis to inhibit pancreatic cancer and lung adenocarcinoma [32, 68], but its role in BC was previously unreported. Here, we show that SLC7A11 and GPX4 are up-regulated in BC tumor tissues, suggesting the inhibition of ferroptosis. After SS treatment, the expressions of SLC7A11, GPX4, ferritin, and GSH/GSSG ratio were downregulated, along with increased levels of Fe^2^⁺, lipid ROS, and apoptotic cells in both in vivo and in vitro. Additionally, ferroptosis inhibitor Fer-1 reversed these trends, suggesting that SS may regulate the progression of BC by activating ferroptosis.

The MAPK signaling pathway activation could induce BC migration and invasion [31]. ERK2 is an important factor in the MAPK classical signaling pathway [12]. Dysregulation of ERK2 expression has been reported to be associated with many human diseases, including cancer [39]. In BC patients, elevated ERK2 levels correlate with shorter survival and increased migration [8, 17]. Dong et al. reported that SS inhibits the ERK/MAPK signaling pathway in bladder cancer [16], but its effects on BC had not been previously reported. In this study, we employed network pharmacology to identify intersection genes between SS and BC disease targets. Bioinformatics analysis revealed that intersection genes were mainly enriched in the ERK2/MAPK signaling pathway, indicating that SS may participate in treating BC through the ERK2/MAPK signaling pathway. Molecular docking analyses indicated that SS might influence the activity of the ERK2/MAPK signaling pathway by binding to ERK2 or EGFR. Further, through kinetic assay, Co-IP, and site-directed mutagenesis experiments, we demonstrate that SS may influence ERK2 phosphorylation and ERK2/MAPK signaling pathway by affecting the T185 phosphorylation site of ERK2 or the Y1069 phosphorylation site of EGFR. Additionally, phosphorylated ERK2 has been reported to enter the nucleus directly or as a dimer, a process regulated by MEK [19, 29]. This suggests that the interaction of SS with ERK2 may also impact the formation of ERK2 dimers, potentially influencing their nuclear translocation and activation of downstream genes. Therefore, we need further research and validation to clarify the regulation of the ERK2/MAPK pathway by SS, including whether the binding of SS to ERK2 affects its nuclear translocation and whether the extracellular binding of SS to EGFR affects its ligand-receptor binding, thereby influencing the activity of the ERK2/MAPK pathway. What’s more, ERK2 possesses multiple phosphorylation sites for MEK, and we would further explore the effects of SS on other potential phosphorylation sites or the roles of phosphorylation at other sites in ferroptosis of BC.

Inhibition of the MAPK signaling pathway activity has been reported to downregulate SLC7A11 expression, leading to ferroptosis induction [58]. However, whether SS could inactivate the ERK/MAPK signaling pathway and induce ferroptosis to treat BC has not been reported. Our findings reveal that ERK2 and p-ERK2 are elevated in BC patients, alongside the up-regulation of SLC7A11 and GPX4. P-ERK2 and ERK2 expressions positively correlated with SLC7A11, indicating ERK2/MAPK involvement in ferroptosis regulation. Furthermore, following the SS treatment, factors associated with the ERK2/MAPK pathway were downregulated in both in vitro and in vivo, alongside reductions in SLC7A11, GPX4, GSH/GSSG ratio, and ferritin, increased Fe^2^⁺ and lipid ROS levels, and these trends were reversible with Fer-1. Thus, we speculate that the ERK2/MAPK pathway may represent a promising therapeutic target for SS in treating BC, with this process linked to ferroptosis. By using ERK/MAPK activator (TPA) and ferroptosis activator (Era), we show that when combined with TPA, the inhibition of SS on the proliferation, migration, and invasion of BC cells could partially be neutralized, indicating that the ERK2/MAPK signaling pathway may be involved in the ability of SS to regulate BC cells. Moreover, the combined effect of SS and TPA was reversed by Era, suggesting that SS could impact ferroptosis through the ERK2/MAPK signaling pathway, thereby modulating BC progression. Moreover, in vivo xenograft models also demonstrate that SS inhibits the ERK2/MAPK pathway, activating ferroptosis and suppressing BC tumor growth. As a substrate for ERK, Ets-1 regulates MMP-9 to promote cell invasion [14]. A relevant study has confirmed that Ets-1 regulates the expression of SLC7A11 by binding to its gene promoter region [33]. Therefore, the inactivation of the MAPK signaling pathway may inhibit the expression of SLC7A11 through the action of Ets-1 and impair transport processes of the system xc^−^, inducing ferroptosis. Changes in Ets-1 expression in our study align with this hypothesis.

However, the regulation of the ERK2/MAPK pathway by SS and the regulation mechanism of SS on BC treatment are both complex and need to be further explored in future studies.

## Conclusion

In summary, Solasonine demonstrates significant anti-tumor effects against breast carcinoma by inducing ferroptosis by inactivating the ERK2/MAPK signaling pathway in both in vivo and in vitro. This study shows the considerable potential of Solasonine in breast cancer treatment, providing a valuable theoretical foundation for future clinical applications.

## Supplementary Information

Supplementary Material 1.

Supplementary Material 2.

## Data Availability

The data that support the findings of this study are available from the corresponding author Xingsong Tian,upon reasonable request.

## Abbreviations

BC: Breast carcinoma
BSA: Bovine serum albumin
Co-IP: Co-immunoprecipitation
DMEM: Dulbecco’s modified Eagle’s medium
DMSO: Dimethyl sulfoxide
ECM: Extracellular matrix
EGFR: Epidermal growth factor receptor
EMT: Epithelial-mesenchymal transition
Era: Erastin
ERK2: Extracellular signal-regulated kinase 2
Ets-1: E-twenty-six-1
Fer-1: Ferristatin-1
GEPIA: Gene Expression Profiling Interactive Analysis 2
G6PD: Glucose-6-phosphate dehydrogenase
GO: Gene Ontology
GPX: Glutathione peroxidase
GPX4: Glutathione peroxidase 4
GSH: Glutathione
GSSG: Oxidized glutathione
HCC: Hepatocellular carcinoma
HRP: Horseradish peroxidase
IHC: Immunohistochemical
KEGG: Kyoto Encyclopedia of Genes and Genomes
Ki: Estimated inhibition constants
LDH: Lactate dehydrogenase
lipid ROS: Lipid reactive oxygen species
MAPK: Mitogen-activated protein kinase
MEK: Mitogen-activated extracellular signal-regulated kinase
NSCLC: Non-small cell lung carcinoma
PBS: Phosphate saline buffer
PFA: Paraformaldehyde
PPI: Protein–protein interactions
SD: Standard deviation
SLC7A11: Solute carrier family 7 member 11
SS: Solasonine
TCM: Traditional Chinese medicine
TCMSP: Traditional Chinese Medicine Systems Pharmacology
TNBC: Triple-negative breast cancer
TPA: 12-O-tetradecanoyl phorbol-13-acetate
WB: Western blot
