# Supplementary material for: Comprehensive network pharmacology and experimental study to investigate the effect and mechanism of solasonine on breast carcinoma treatment
Source: Cancer Cell Int. 2025 Feb 17;25:49. doi: 10.1186/s12935-025-03665-6 (PMC11834262; doi:10.1186/s12935-025-03665-6)
Supplement: Supplementary file 1 — Supplementary Material 1. [file 12935_2025_3665_MOESM1_ESM.docx]

**Supplemental figures**

**Fig. S_1_.**


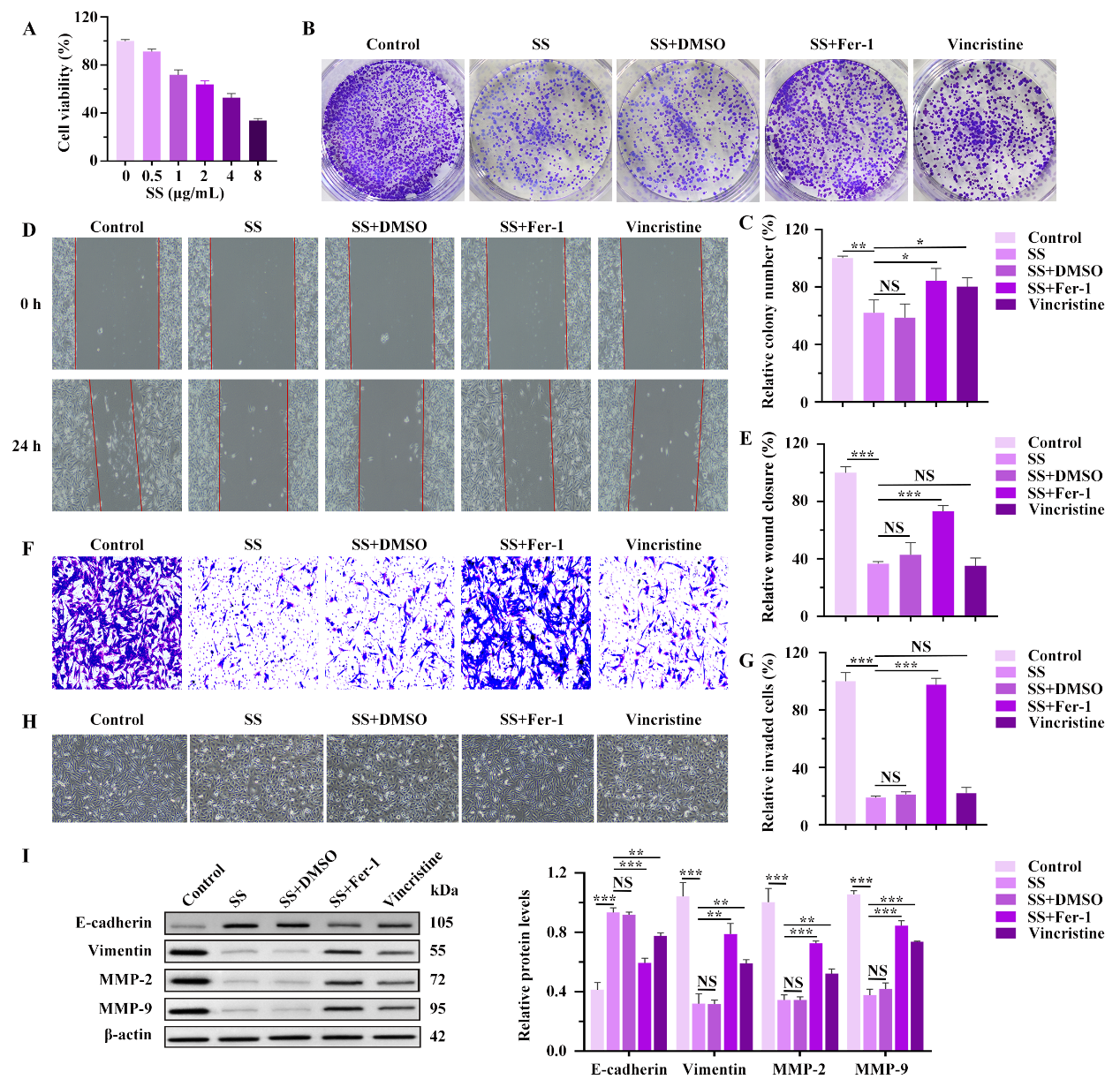


**Fig. S_1_. SS inhibits the proliferation, migration, and invasion of MDA-MB-231 cells through ferroptosis.** (**A**) CCK8 assay. (**B**) Clone formation assay and its quantitation (**C**). (**D**) Wound-healing assay and its quantitation (**E**). Transwell matrigel invasion assay (**F**) and its quantitation (**G**). (**H**) The morphological changes in MDA-MB-231 cells of each group were observed by microscope. (**I**) The expression levels of epithelial-mesenchymal transition (EMT)-related factors detected by WB. **p* < 0.05, ***p* < 0.01, ****p* < 0.001.

**Fig. S_2_.**


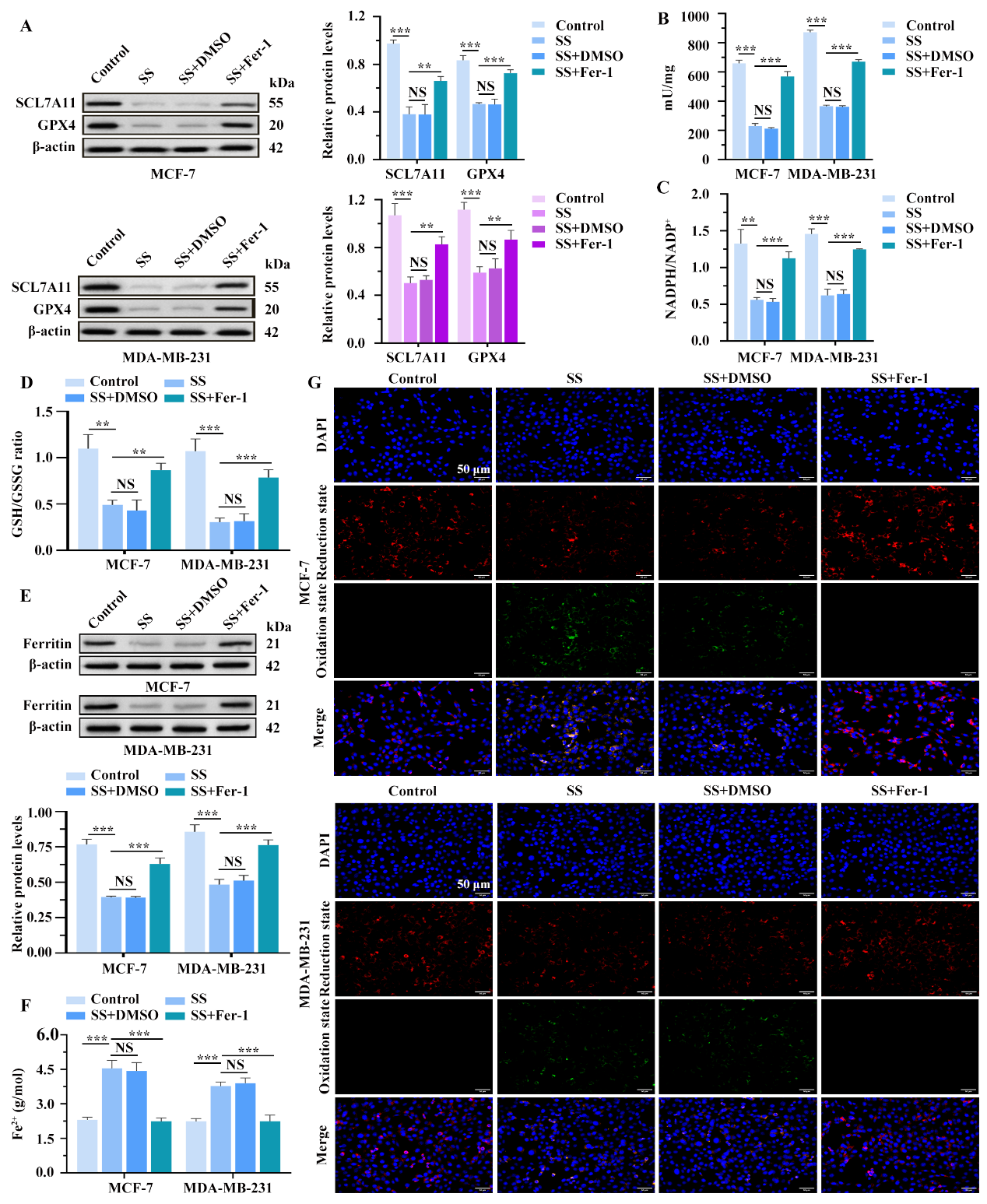


**Fig. S_2_. SS activates ferroptosis in BC cells.** (**A**) The expression levels of SLC7A11 and GPX4 were detected by WB. (**B**) The activity of glutathione peroxidase (GPX) was detected using a GPX assay kit. (**C**) The NADPH/NADP^+^ ratio was measured using a NADP^+^/NADPH detection kit. (**D**) Measurement of reduced glutathione (GSH)/oxidized glutathione (GSSG) ratio by GSH and GSSG assay kit. (**E**) The expression of ferritin was detected by WB. (**F**) Iron levels were detected using the iron assay kit. (**G**) Detection of lipid reactive oxygen species (ROS) using C11-BODIPY 581/591 kit. ***p* < 0.01, ****p* < 0.001.

**Fig. S_3_.**


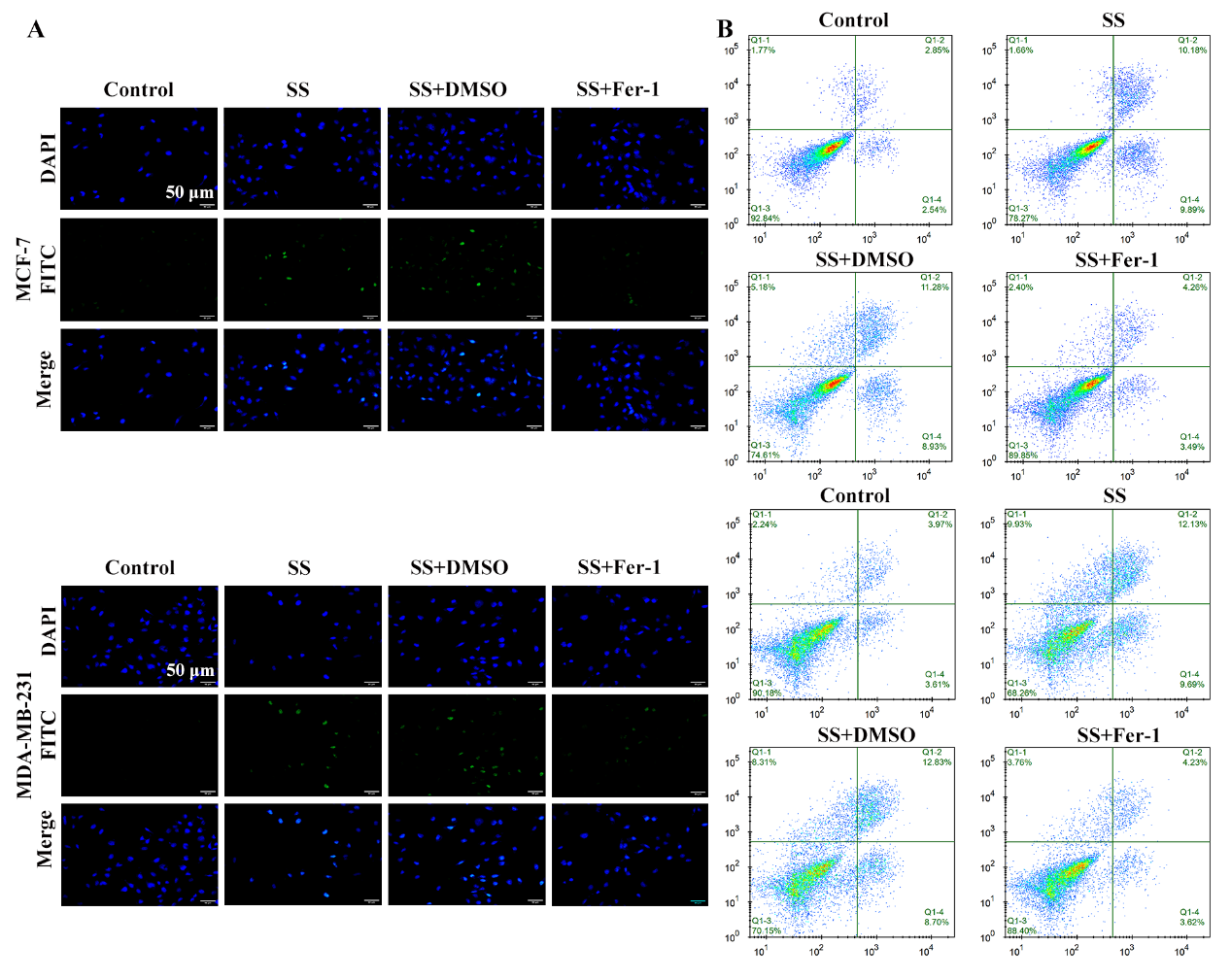


**Fig. S_3_. SS activates ferroptosis to promote BC cell death.** (**A**) TUNEL assay. (**B**) The apoptosis of BC cells was detected by flow cytometry.

**Fig. S_4_.**


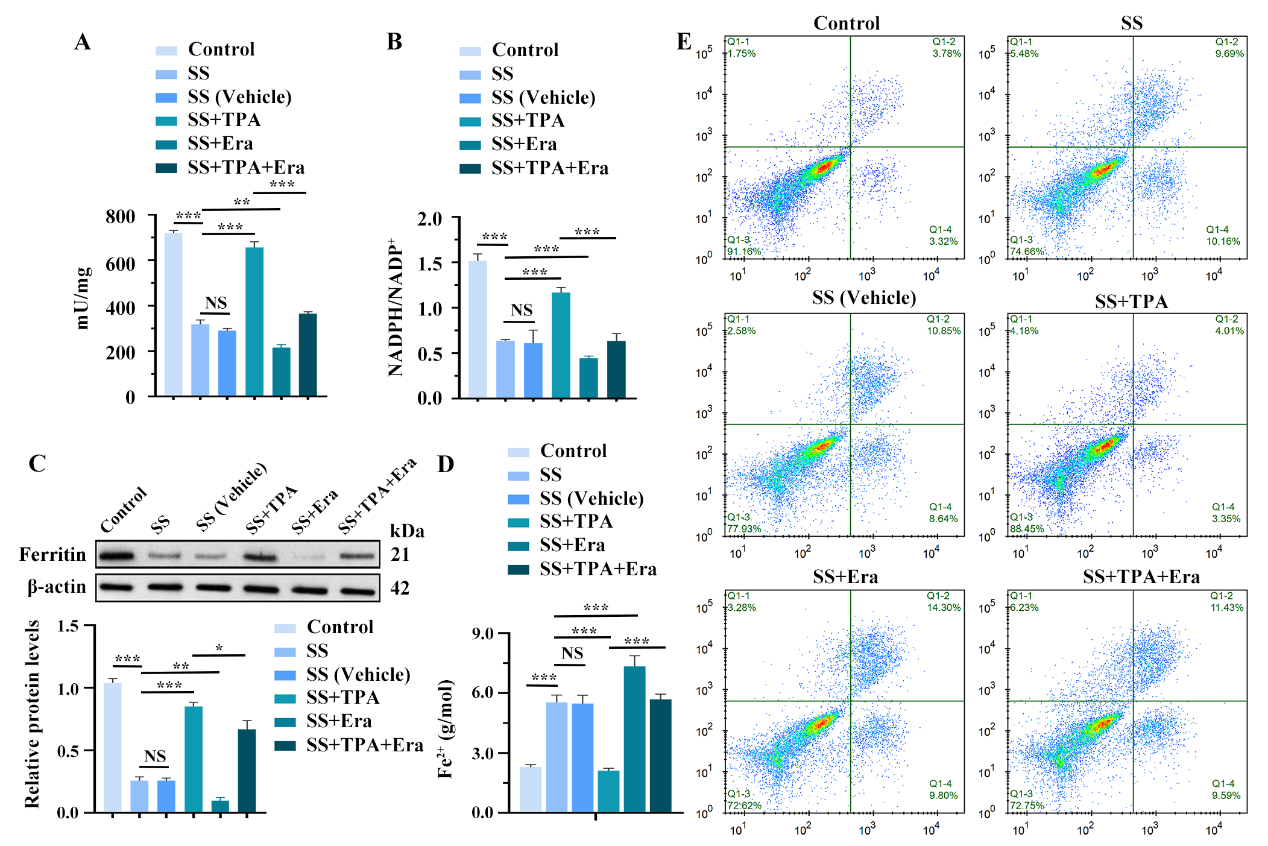


**Fig. S_4_. SS inhibits the ERK2/MAPK signaling pathway to activate ferroptosis in MCF-7 cells.** (**A**) The activity of GPX was detected using a GPX assay kit. (**B**) The NADPH/NADP^+^ ratio was measured using a NADP^+^/NADPH detection kit. (**C**) The expression of ferritin was detected by WB. (**D**) Iron levels were detected using the iron assay kit. (**E**) The apoptosis of MCF-7 cells was detected by flow cytometry. **p* < 0.05, ***p* < 0.01, ****p* < 0.001.

**Fig. S_5_.**


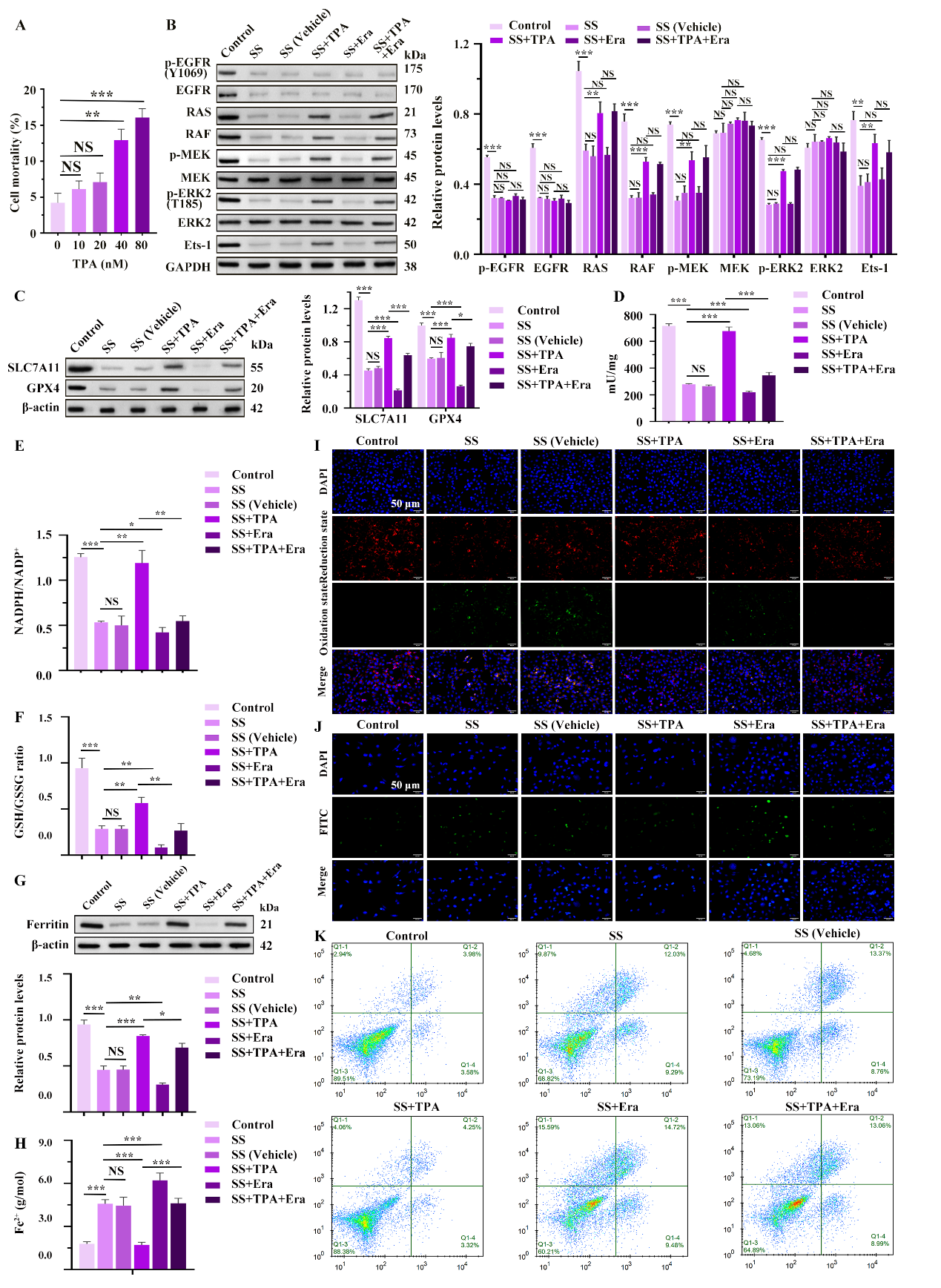


**Fig. S_5_. SS inhibits the ERK2/MAPK signaling pathway to activate ferroptosis and promote the death of MDA-MB-231 cells.** (**A**) Extracellular lactate dehydrogenase (LDH) release was detected by a LDH cytotoxicity detection kit. (**B**) The expressions of ERK2/MAPK signaling pathway-related factors in MDA-MB-231 cells treated with SS were analyzed by WB and its quantitation. (**C**) The expression levels of SLC7A11 and GPX4 were detected by WB. (**D**) The activity of GPX was detected using a GPX assay kit. (**E**) The NADPH/NADP^+^ ratio was measured using a NADP^+^/NADPH detection kit. (**F**) Measurement of GSH/GSSG ratio by GSH and GSSG assay kit. (**G**) The expression of ferritin was detected by WB. (**H**) Iron levels were detected using the iron assay kit. (**I**) Detection of lipid ROS using C11-BODIPY 581/591 kit. (**J**) TUNEL assay. (**K**) The apoptosis of MDA-MB-231 cells was detected by flow cytometry. **p* < 0.05, ***p* < 0.01, ****p* < 0.001.

**Fig. S_6_.**


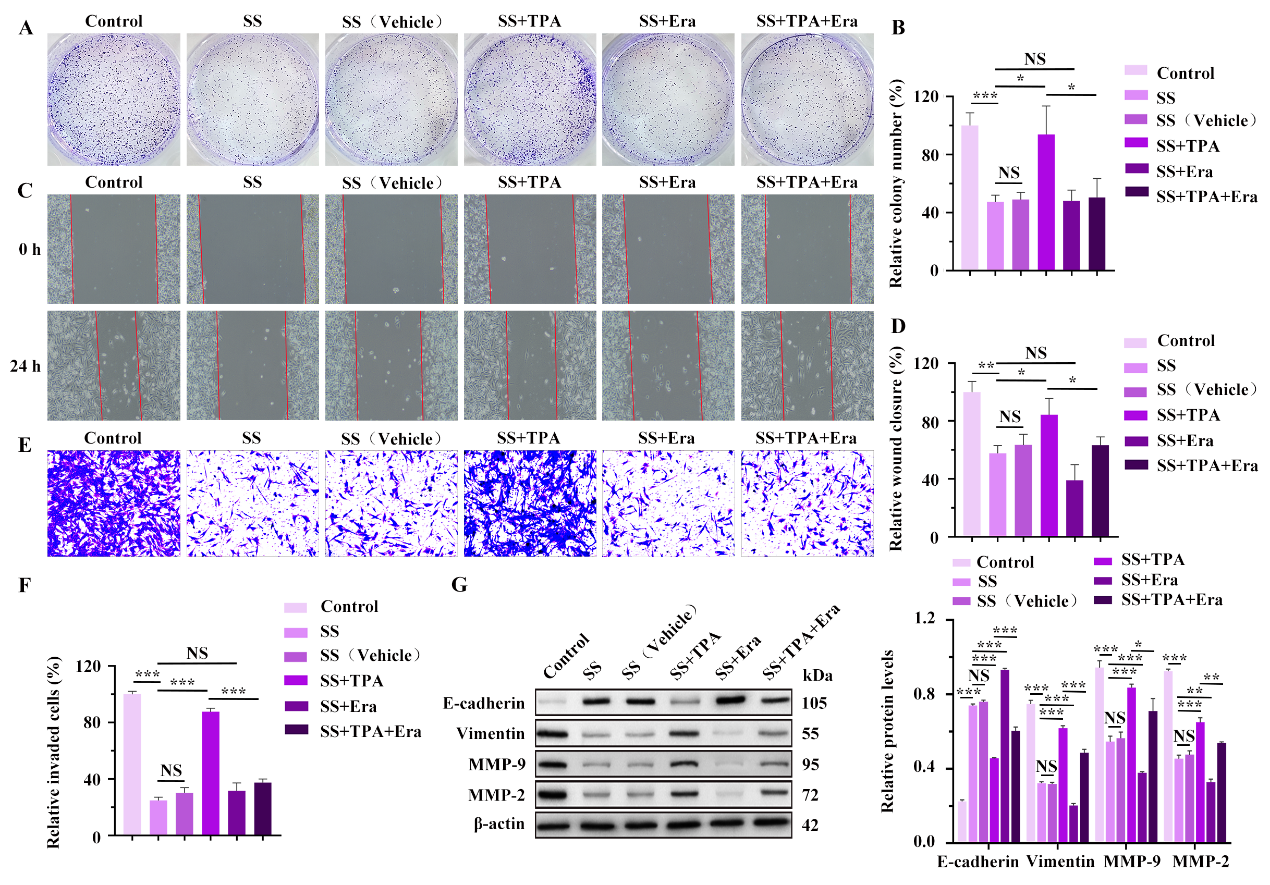


**Fig. S_6_. SS activates ferroptosis by suppressing the ERK2/MAPK signaling pathway to inhibit the proliferation, migration, and invasion of MDA-MB-231 cells.** (**A**) Clone formation assay and its quantitation (**B**). (**C**) Wound-healing assay and its quantitation (**D**). (**E**) Transwell matrigel invasion assay and its quantitation graph (**F**). (**G**) The expression levels of EMT-related factors were detected by WB. **p* < 0.05, ***p* < 0.01, ****p* < 0.001.

**Fig. S_7_.**


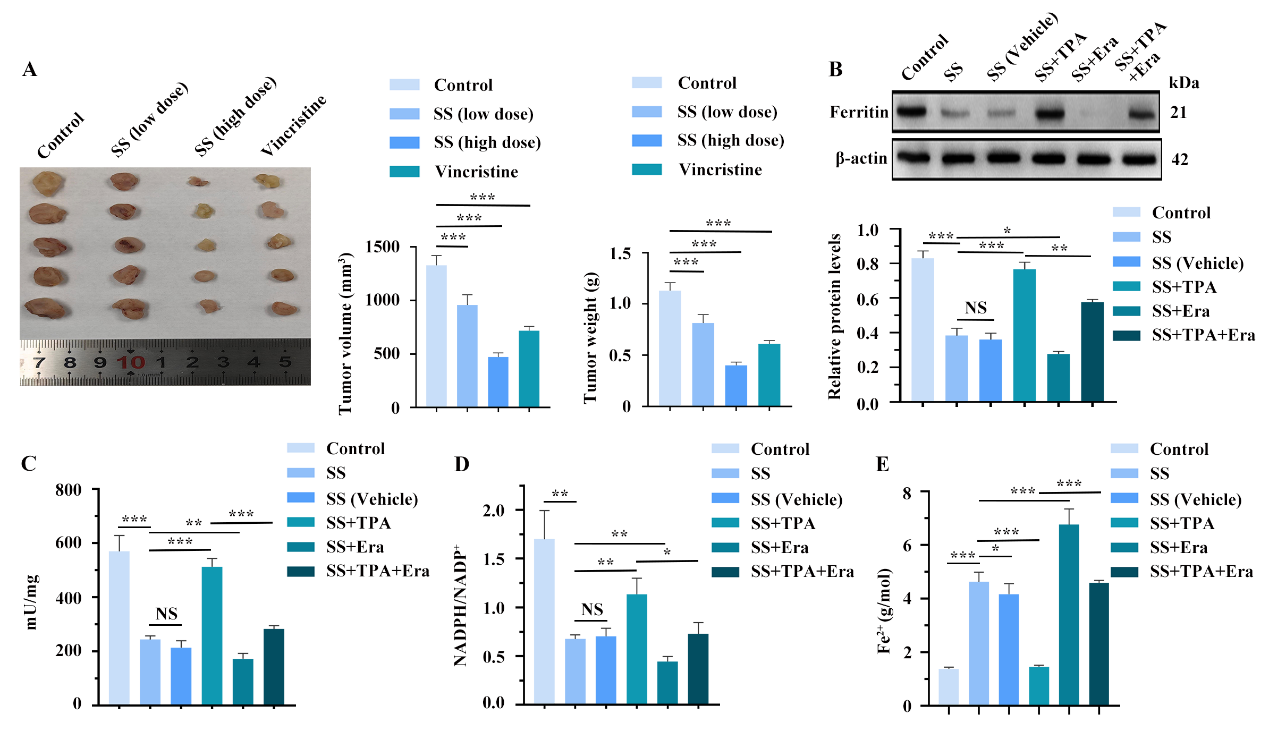


**Fig. S_7_. SS induces ferroptosis by inhibiting the ERK2/MAPK signaling pathway to regulate the development of BC.** (**A**) Inhibitory effect of low and high-dose SS on the tumor (n=5). (**B**) The expression of ferritin was detected by WB (n=5). (**C**) The activity of GPX was detected using a GPX assay kit. (**D**) The NADPH/NADP^+^ ratio was measured using a NADP^+^/NADPH detection kit. (**E**) Iron levels were detected using an iron assay kit (n=5). **p* < 0.05, ***p* < 0.01, ****p* < 0.001.
