# Supplementary figures and images for: Comprehensive network pharmacology and experimental study to investigate the effect and mechanism of solasonine on breast carcinoma treatment (part 2 of 2)
Source: Cancer Cell Int. 2025 Feb 17;25:49. doi: 10.1186/s12935-025-03665-6 (PMC11834262; doi:10.1186/s12935-025-03665-6)

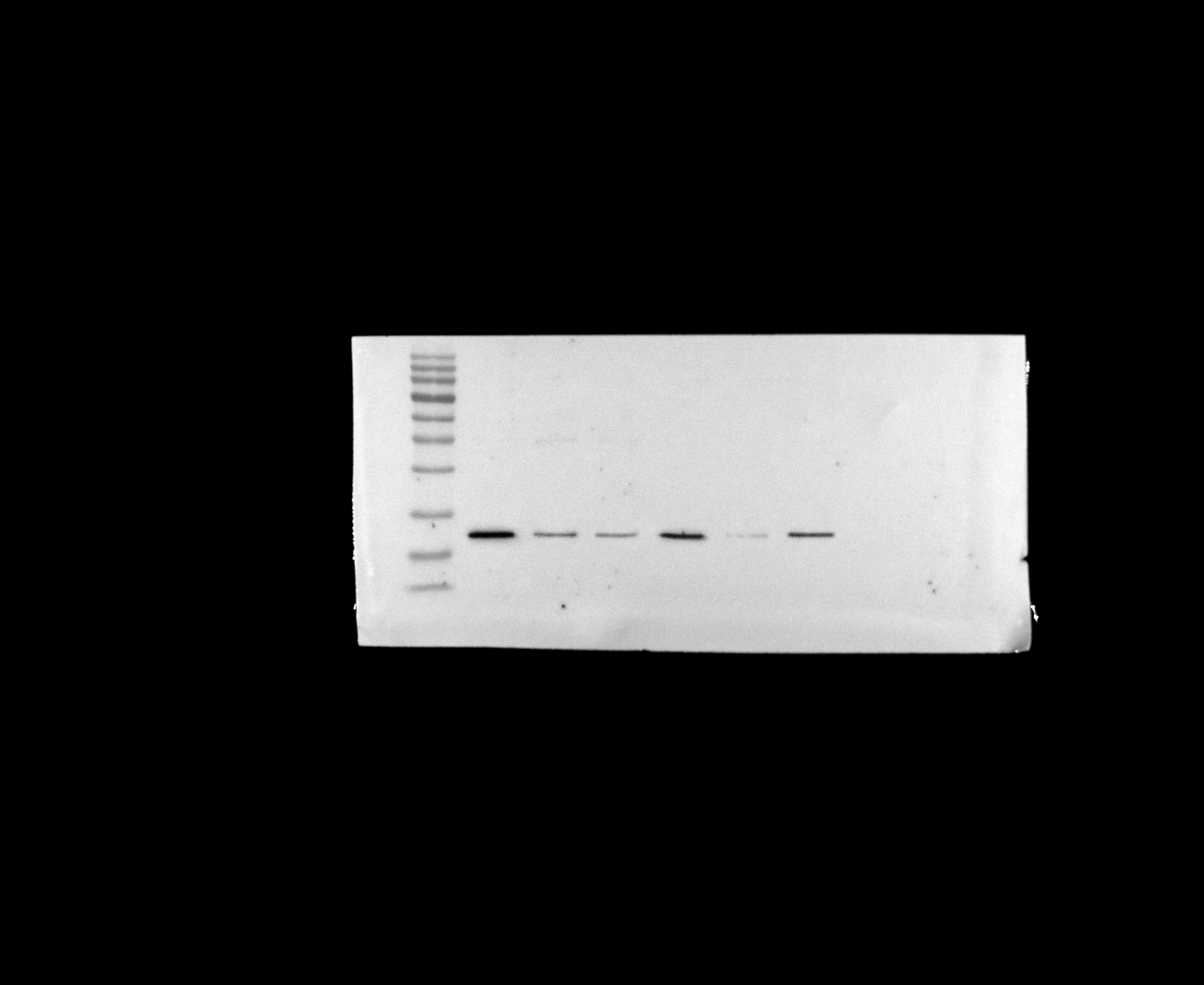

Supplement: Supplementary file 2 — Supplementary Material 2. [file 12935_2025_3665_MOESM2_ESM.zip › Supplementary Material 2/Figure S4/Figure S4C/Ferritin.tif]

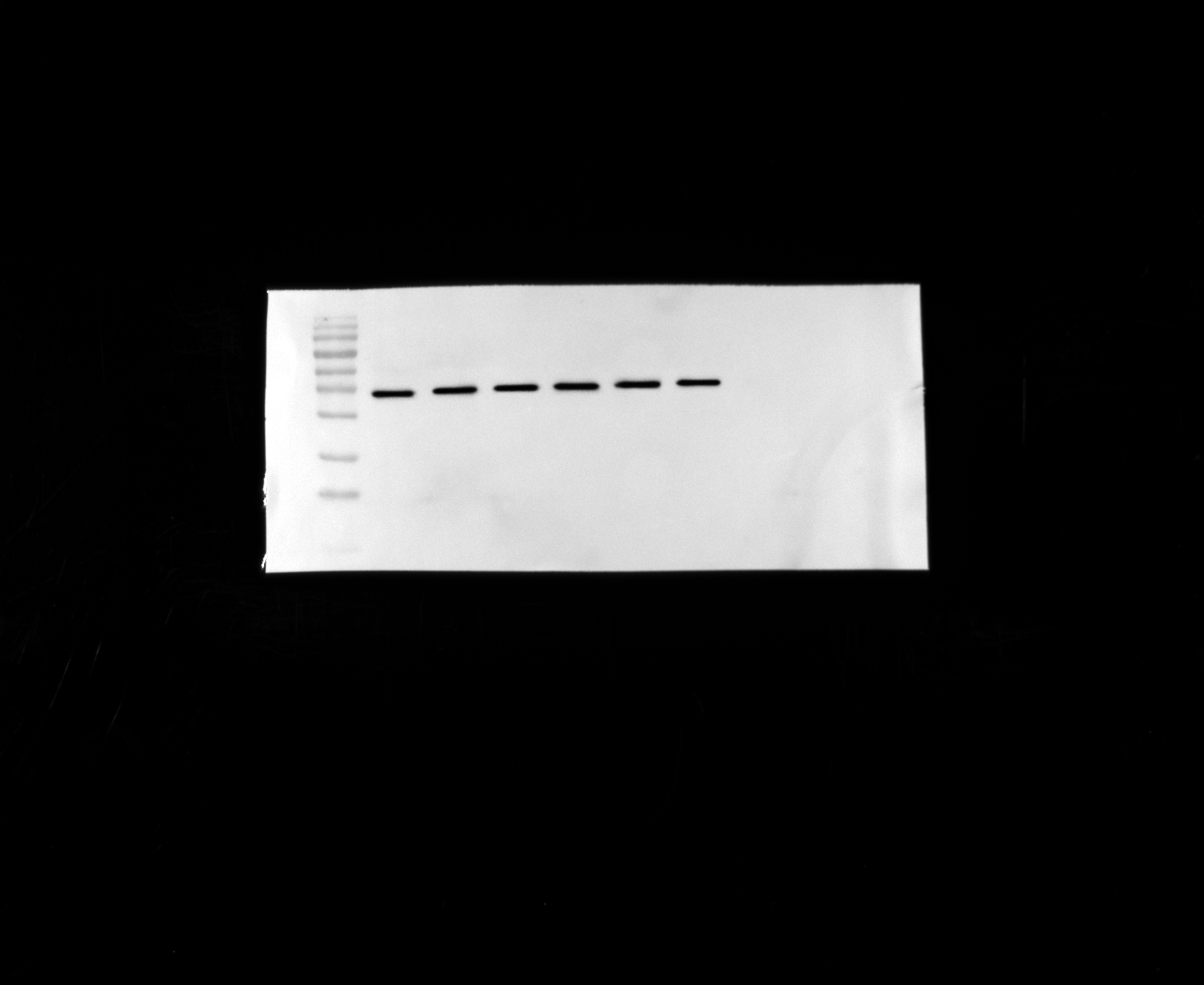

Supplement: Supplementary file 2 — Supplementary Material 2. [file 12935_2025_3665_MOESM2_ESM.zip › Supplementary Material 2/Figure S4/Figure S4C/β-actin.tif]

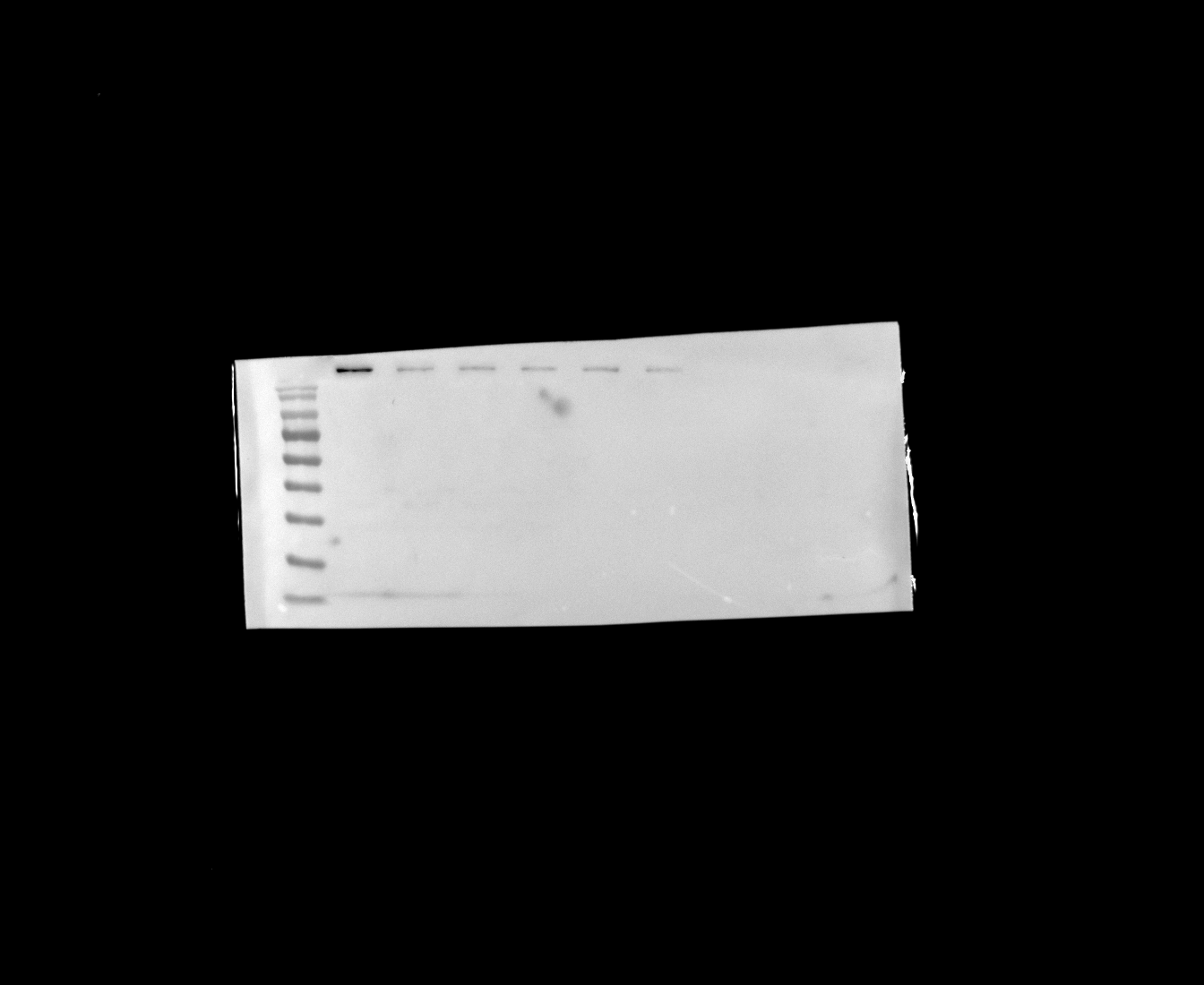

Supplement: Supplementary file 2 — Supplementary Material 2. [file 12935_2025_3665_MOESM2_ESM.zip › Supplementary Material 2/Figure S5/Figure S5B/EGFR .tif]

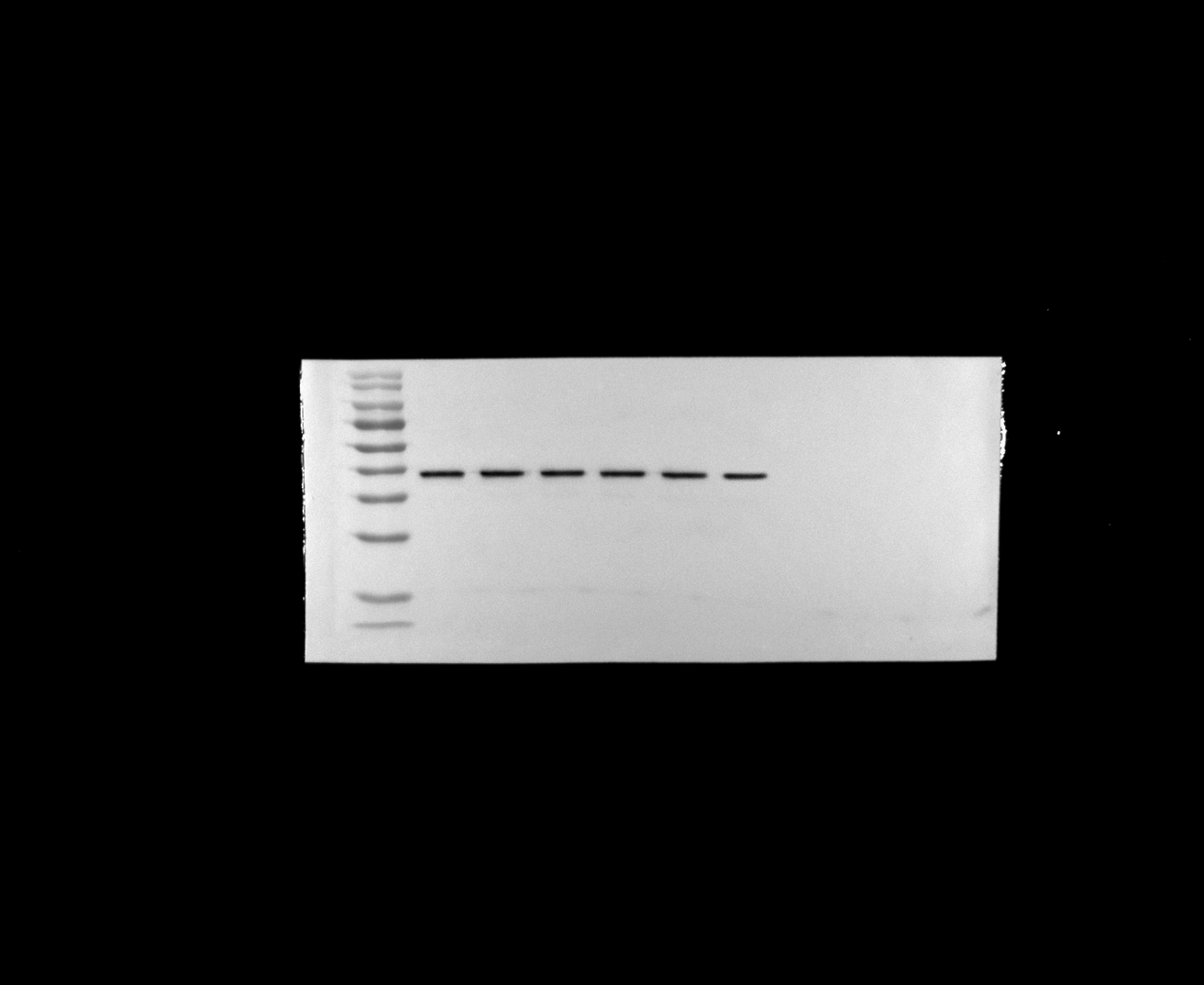

Supplement: Supplementary file 2 — Supplementary Material 2. [file 12935_2025_3665_MOESM2_ESM.zip › Supplementary Material 2/Figure S5/Figure S5B/ERK2.tif]

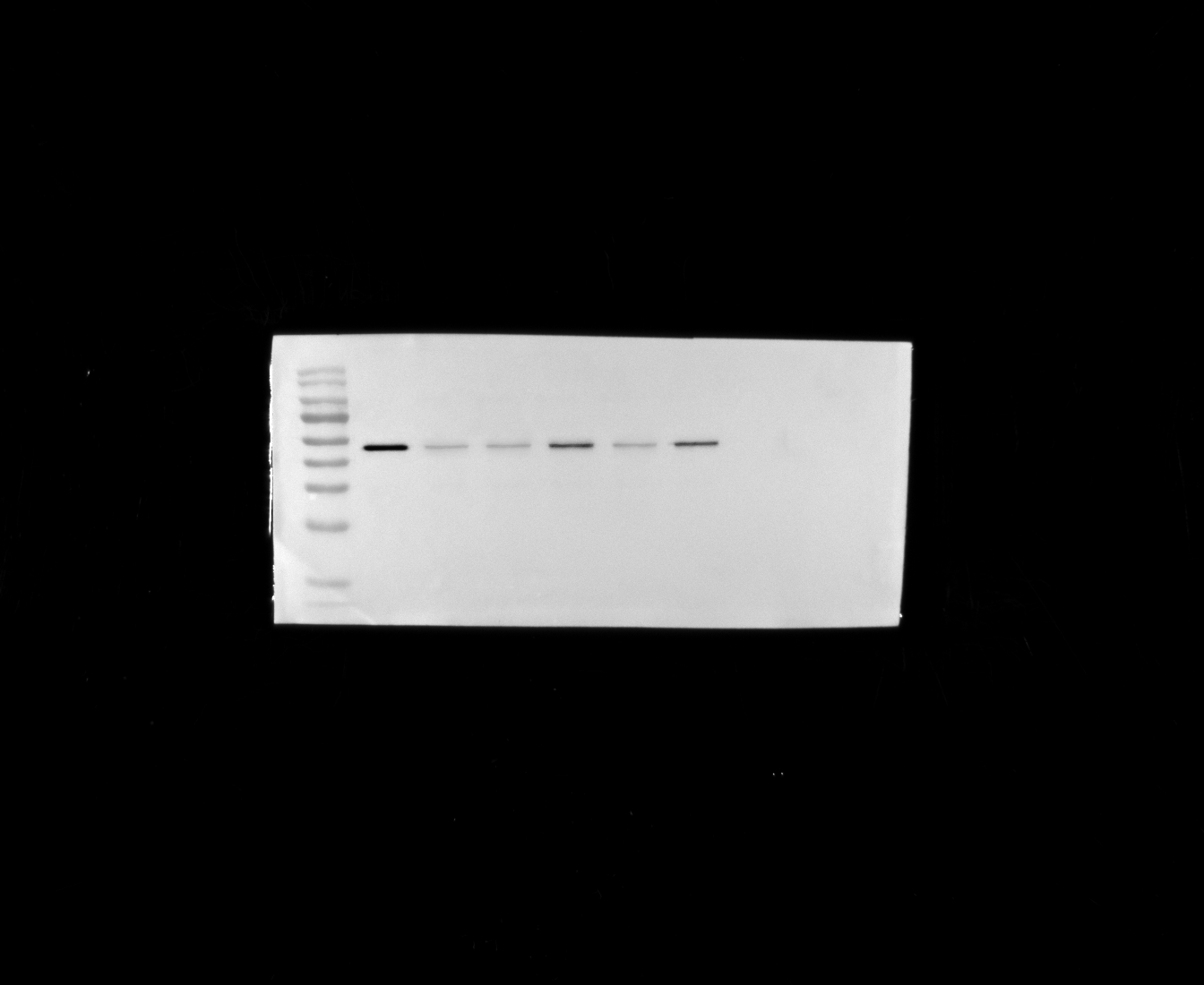

Supplement: Supplementary file 2 — Supplementary Material 2. [file 12935_2025_3665_MOESM2_ESM.zip › Supplementary Material 2/Figure S5/Figure S5B/Ets-1.tif]

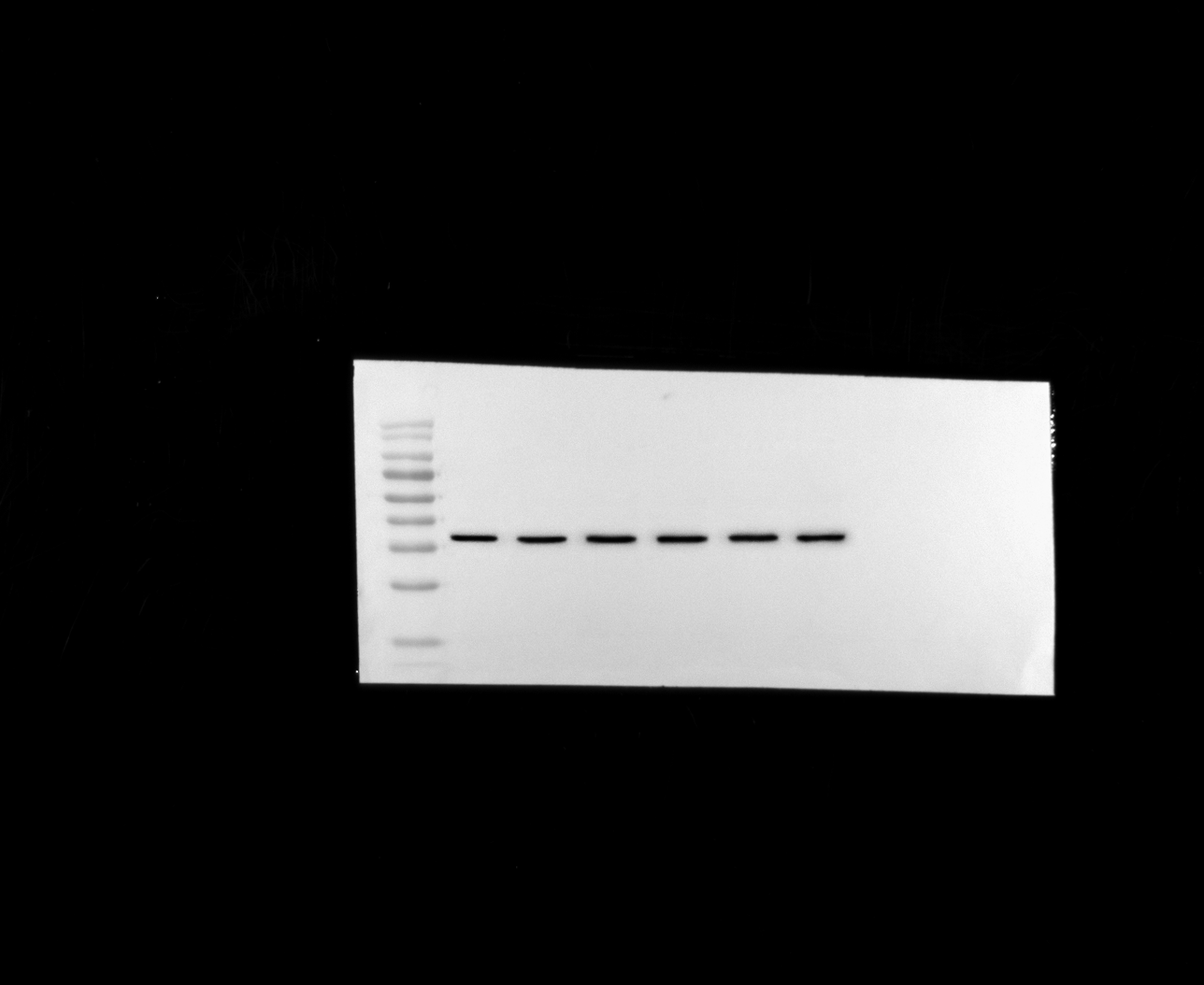

Supplement: Supplementary file 2 — Supplementary Material 2. [file 12935_2025_3665_MOESM2_ESM.zip › Supplementary Material 2/Figure S5/Figure S5B/GAPDH.tif]

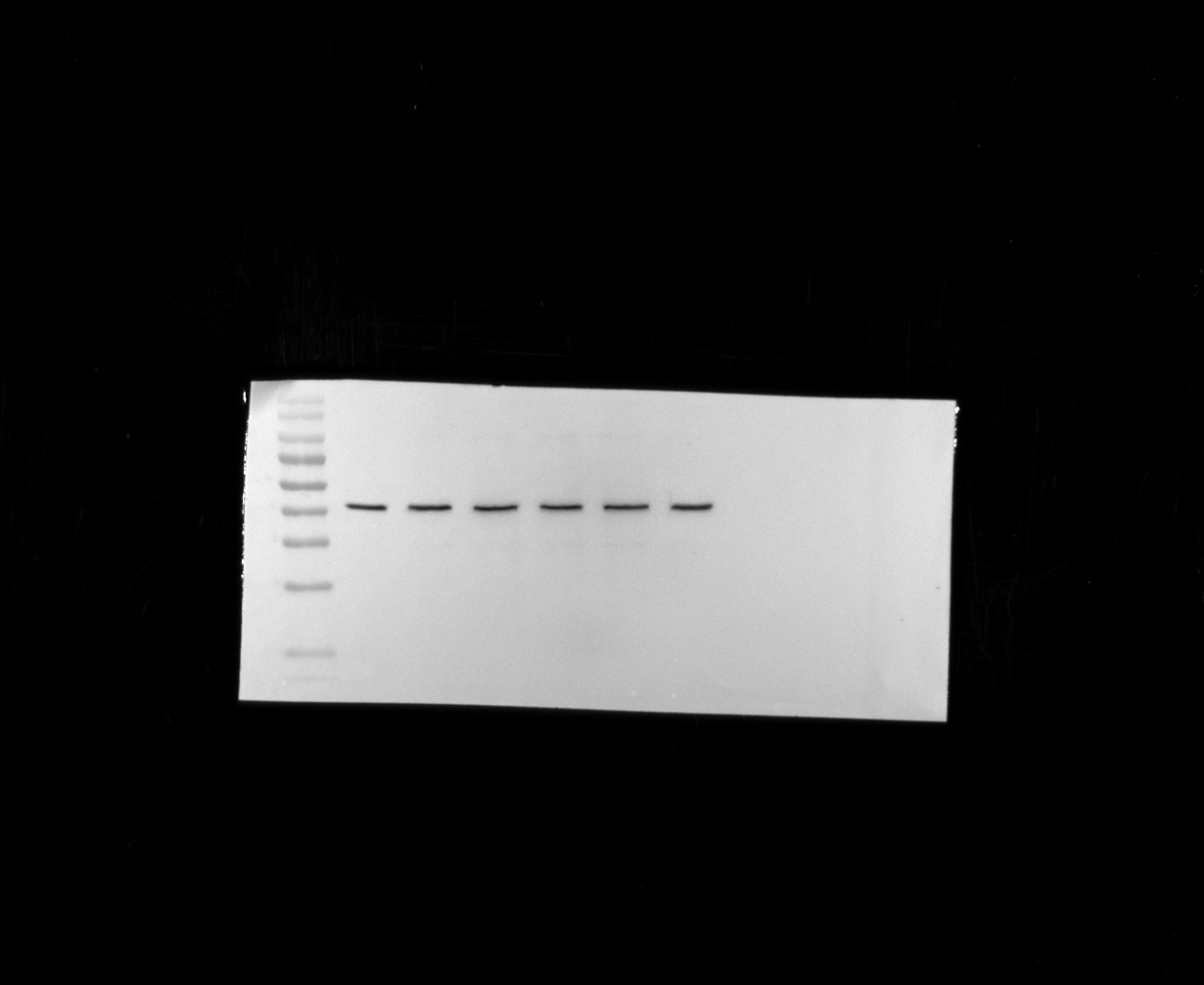

Supplement: Supplementary file 2 — Supplementary Material 2. [file 12935_2025_3665_MOESM2_ESM.zip › Supplementary Material 2/Figure S5/Figure S5B/MEK.tif]

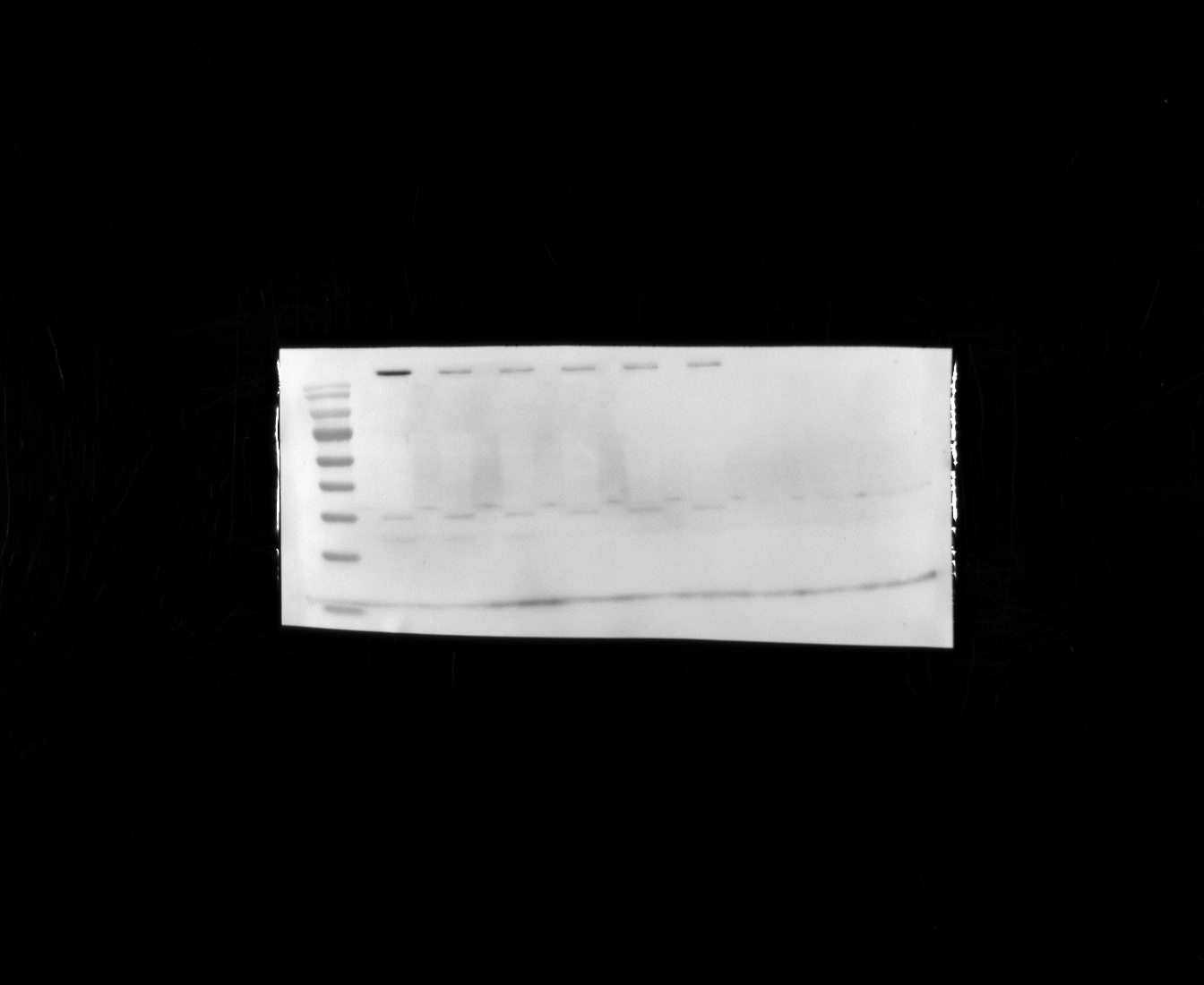

Supplement: Supplementary file 2 — Supplementary Material 2. [file 12935_2025_3665_MOESM2_ESM.zip › Supplementary Material 2/Figure S5/Figure S5B/p-EGFR(Y1069) .tif]

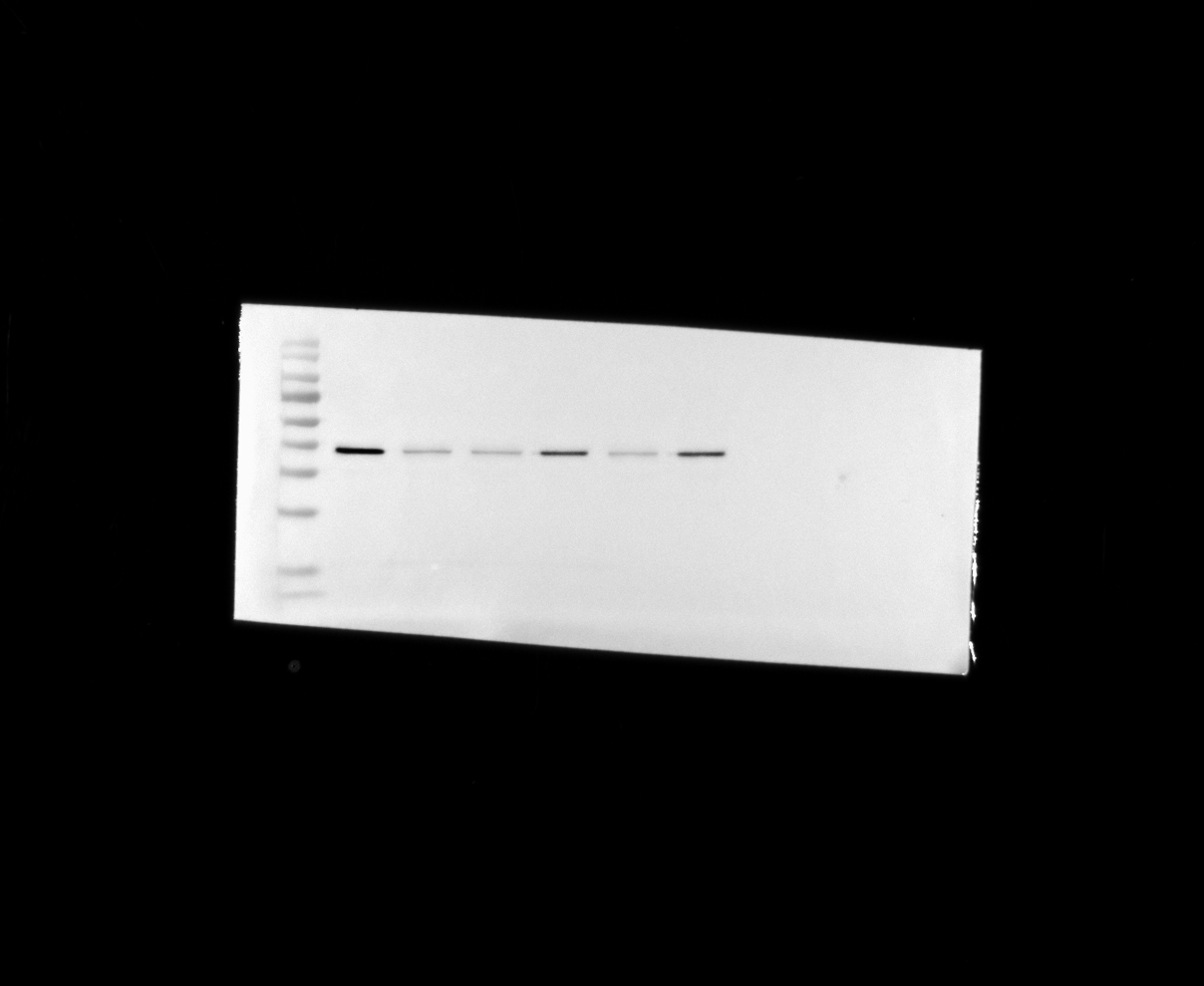

Supplement: Supplementary file 2 — Supplementary Material 2. [file 12935_2025_3665_MOESM2_ESM.zip › Supplementary Material 2/Figure S5/Figure S5B/p-ERK2(T185).tif]

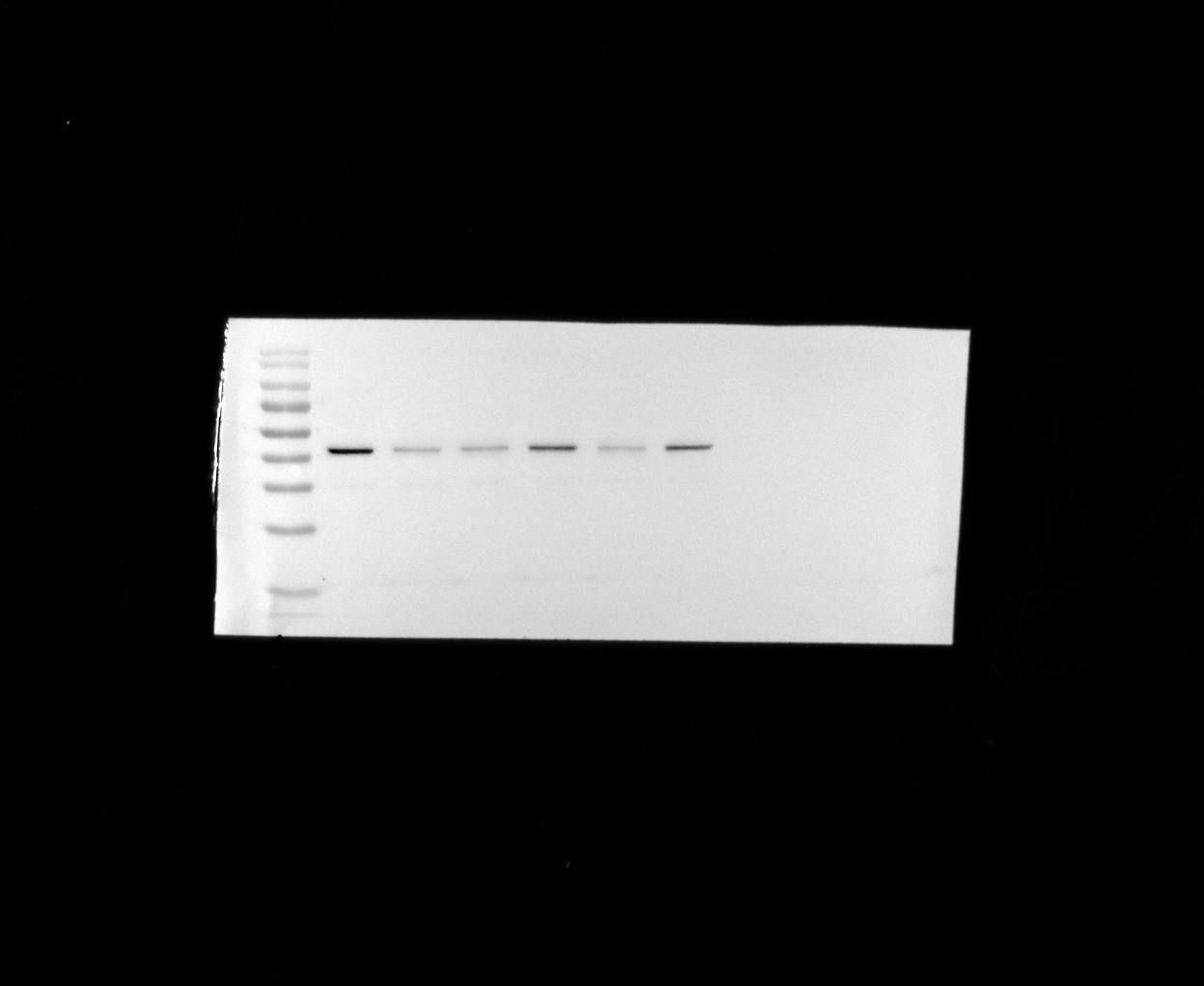

Supplement: Supplementary file 2 — Supplementary Material 2. [file 12935_2025_3665_MOESM2_ESM.zip › Supplementary Material 2/Figure S5/Figure S5B/p-MEK.tif]

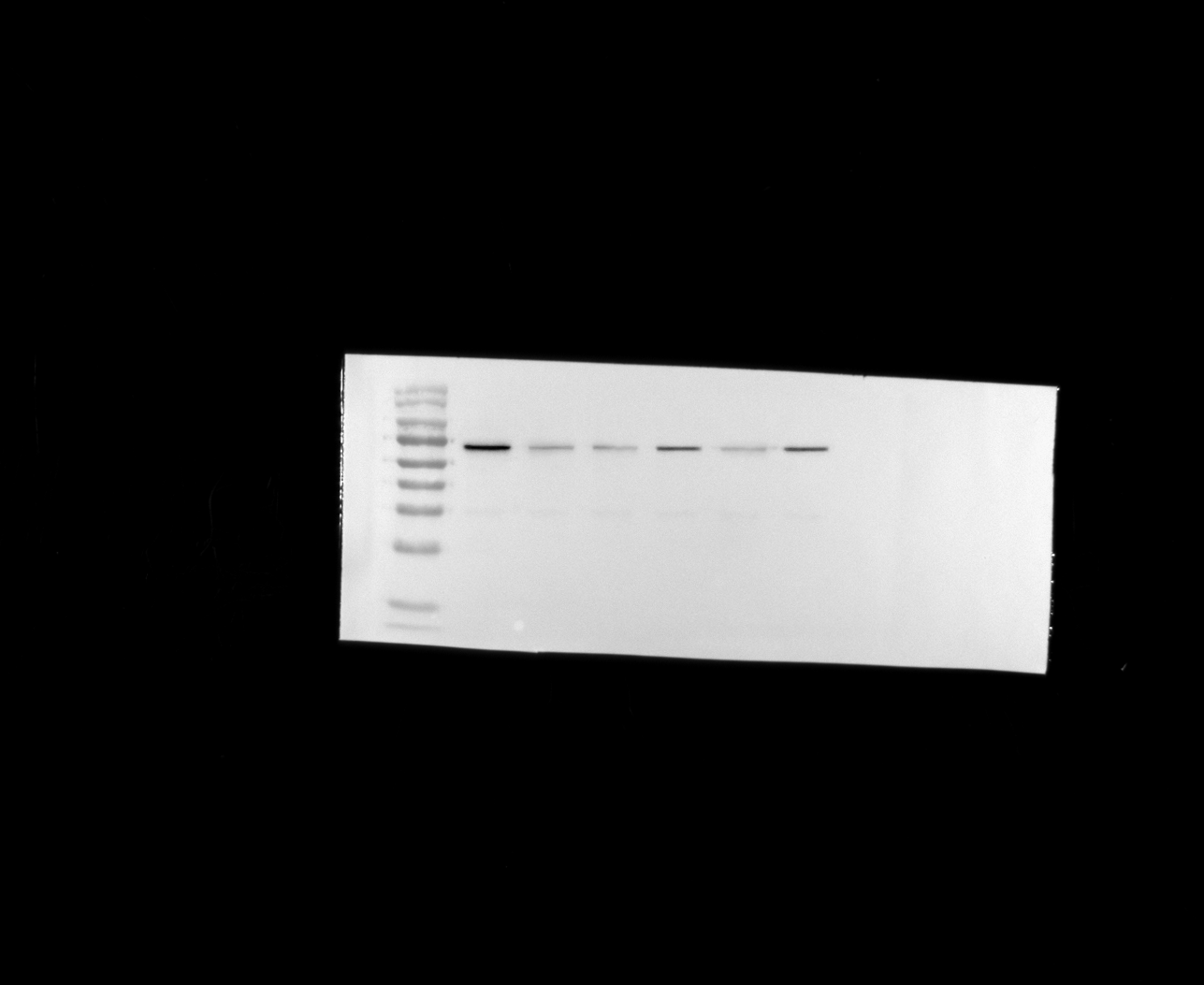

Supplement: Supplementary file 2 — Supplementary Material 2. [file 12935_2025_3665_MOESM2_ESM.zip › Supplementary Material 2/Figure S5/Figure S5B/RAF.tif]

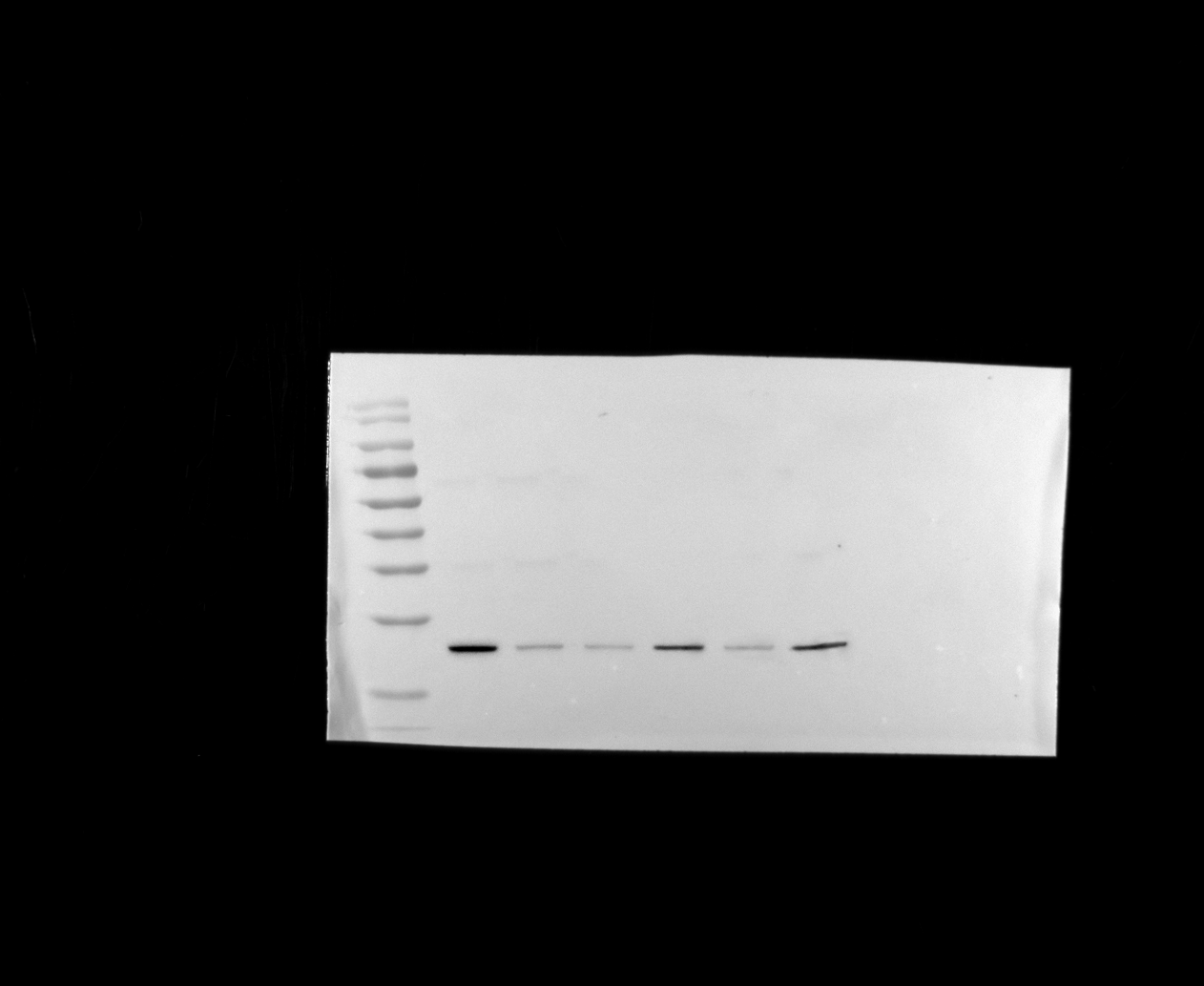

Supplement: Supplementary file 2 — Supplementary Material 2. [file 12935_2025_3665_MOESM2_ESM.zip › Supplementary Material 2/Figure S5/Figure S5B/RAS.tif]

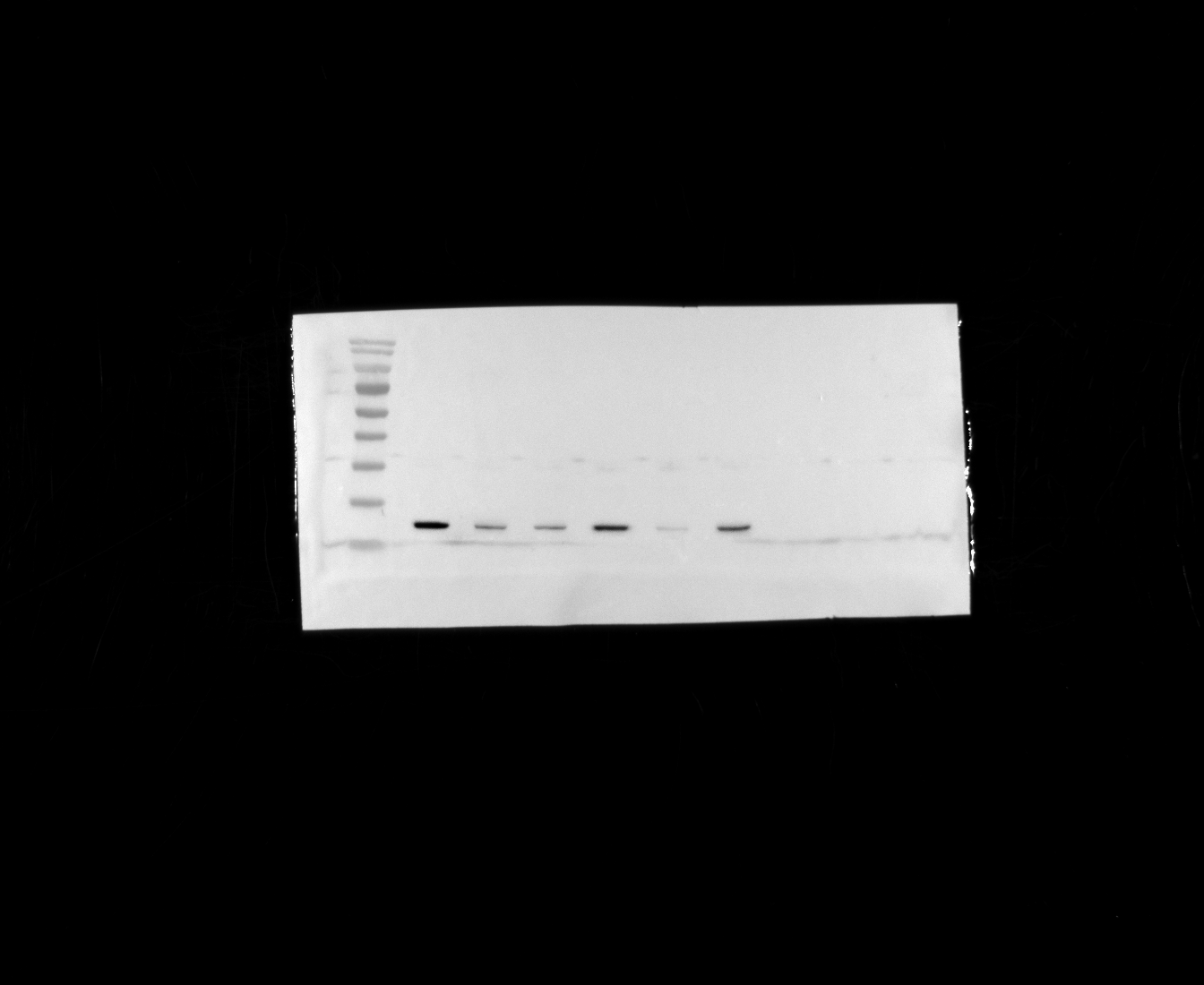

Supplement: Supplementary file 2 — Supplementary Material 2. [file 12935_2025_3665_MOESM2_ESM.zip › Supplementary Material 2/Figure S5/Figure S5C/GPX4.tif]

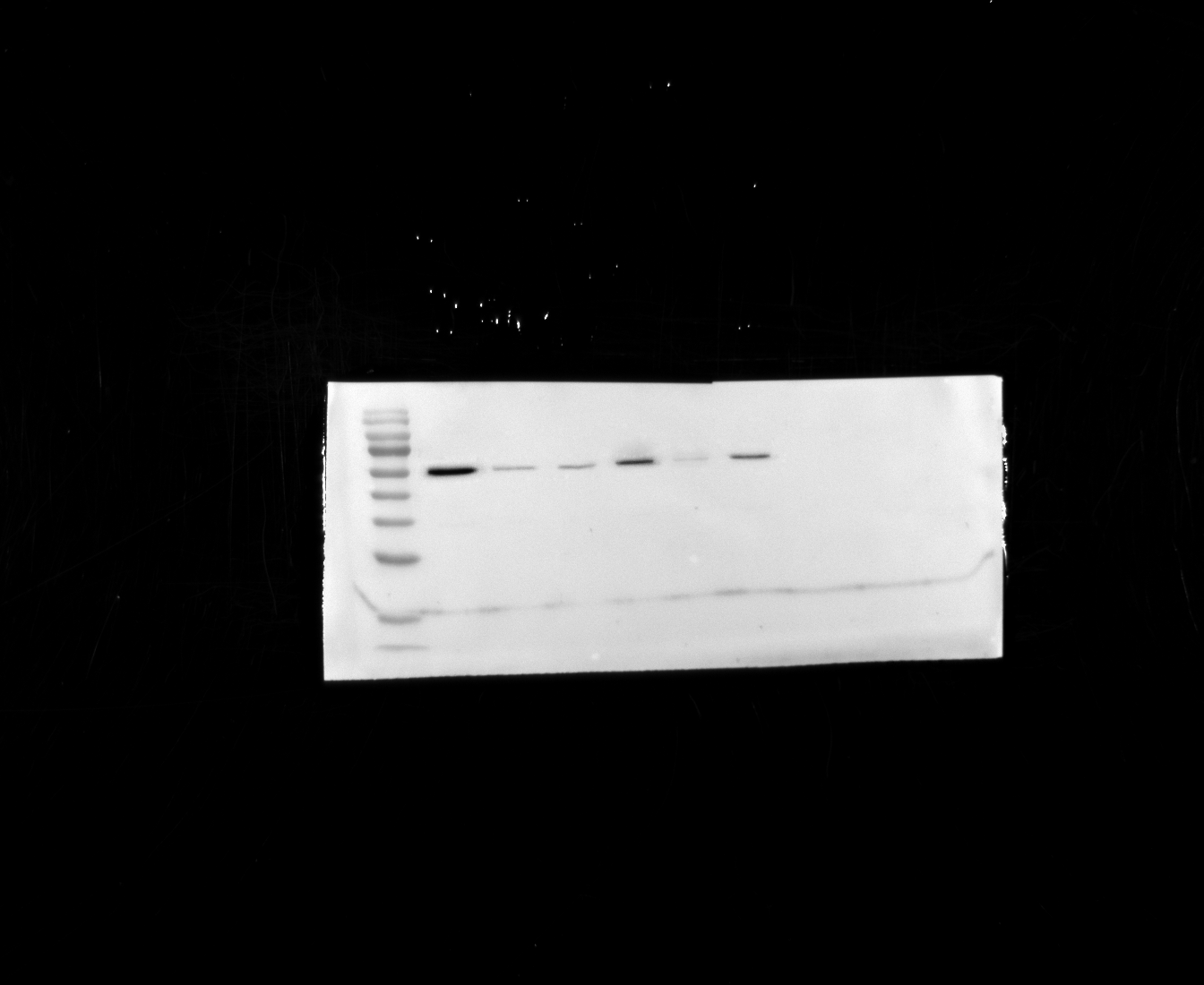

Supplement: Supplementary file 2 — Supplementary Material 2. [file 12935_2025_3665_MOESM2_ESM.zip › Supplementary Material 2/Figure S5/Figure S5C/SLC7A11.tif]

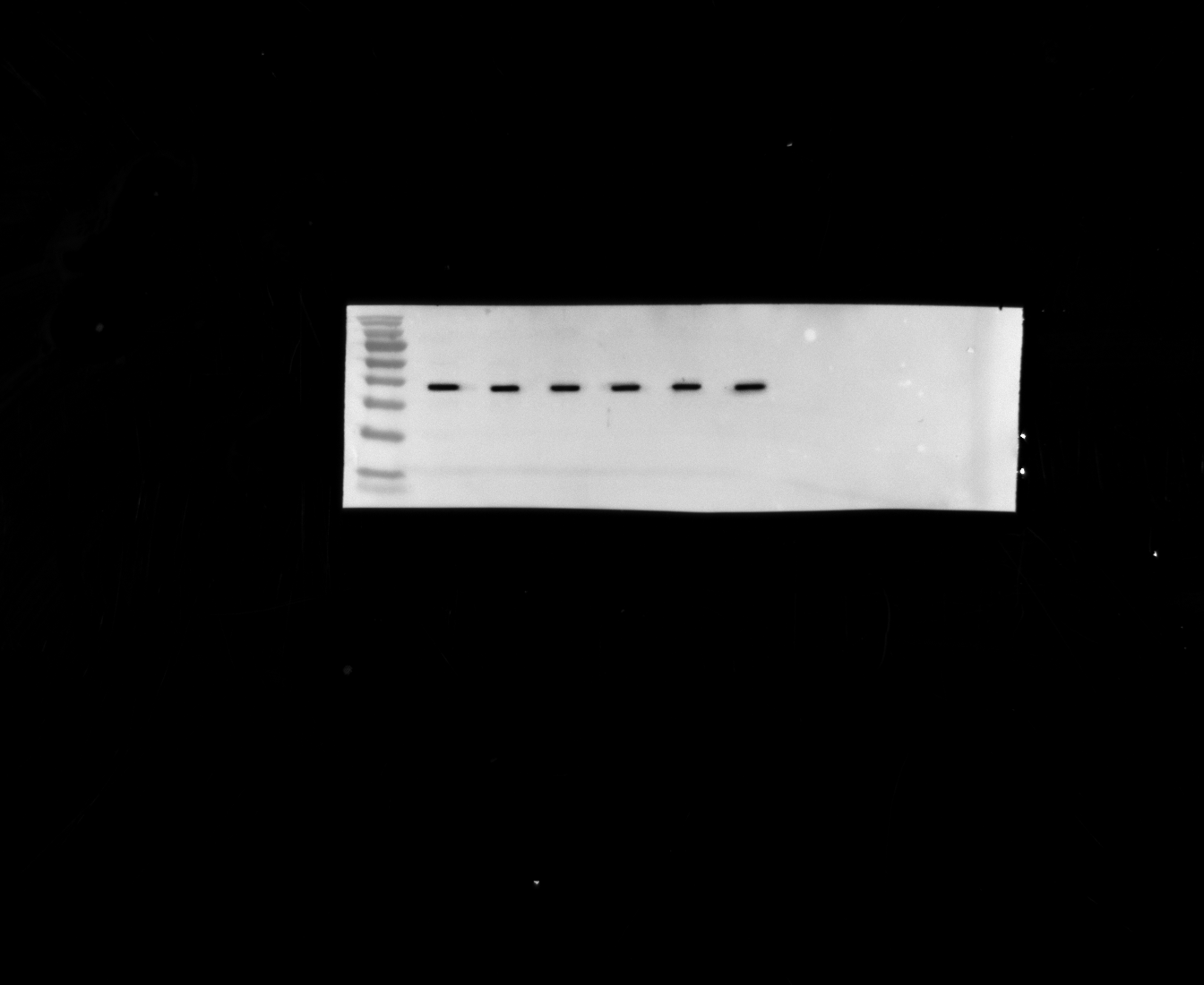

Supplement: Supplementary file 2 — Supplementary Material 2. [file 12935_2025_3665_MOESM2_ESM.zip › Supplementary Material 2/Figure S5/Figure S5C/β-actin.tif]

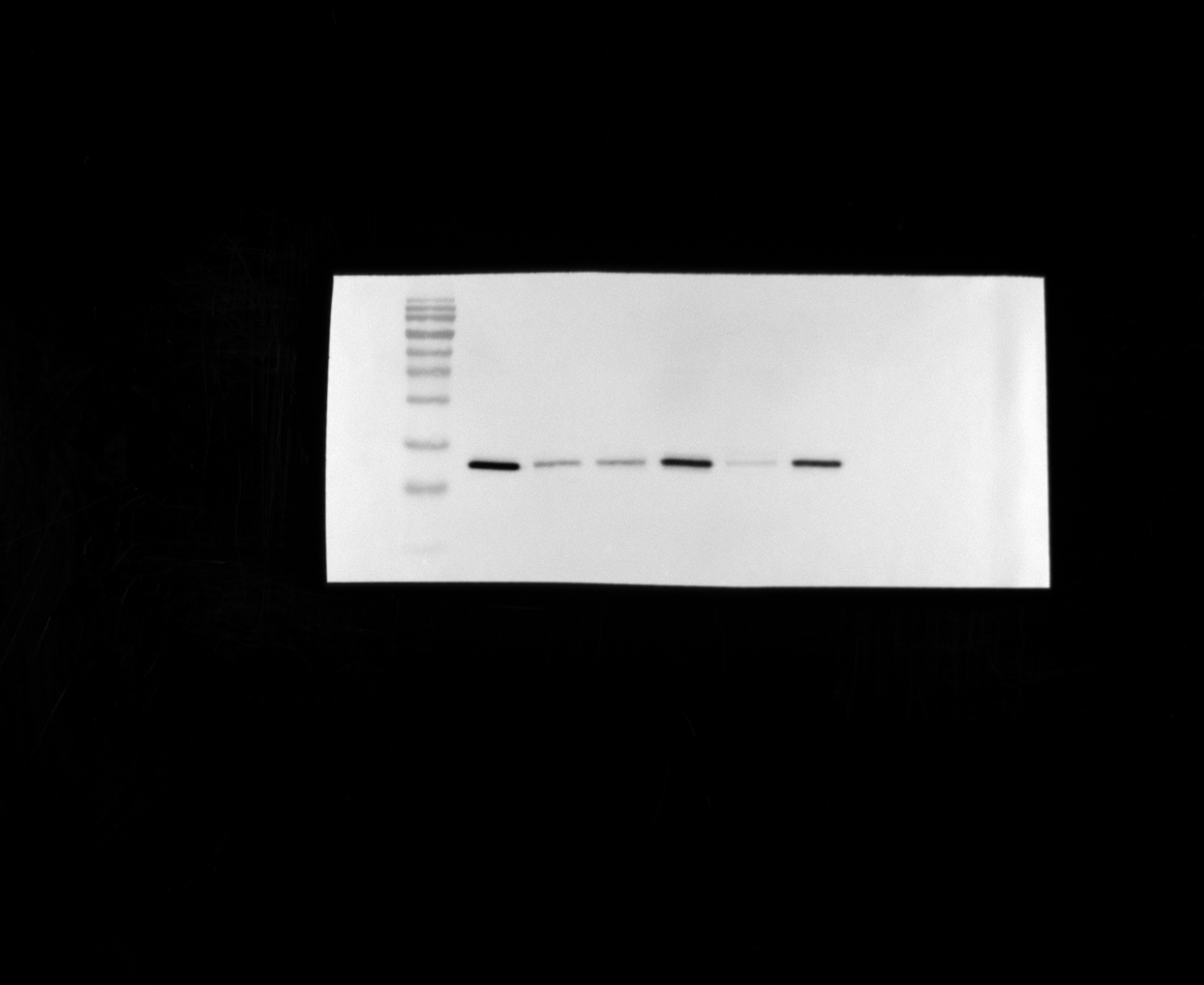

Supplement: Supplementary file 2 — Supplementary Material 2. [file 12935_2025_3665_MOESM2_ESM.zip › Supplementary Material 2/Figure S5/Figure S5G/Ferritin.tif]

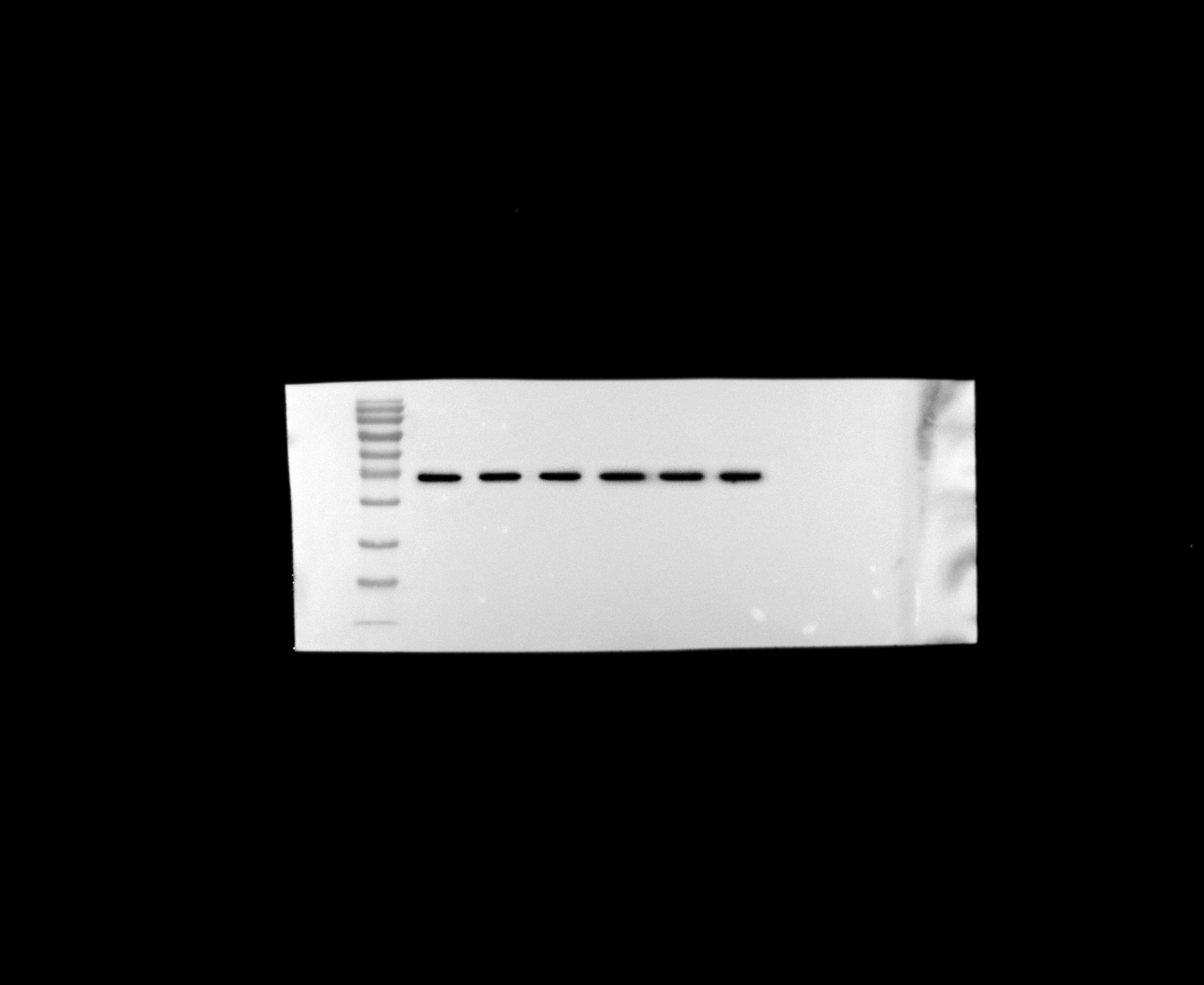

Supplement: Supplementary file 2 — Supplementary Material 2. [file 12935_2025_3665_MOESM2_ESM.zip › Supplementary Material 2/Figure S5/Figure S5G/β-actin.tif]

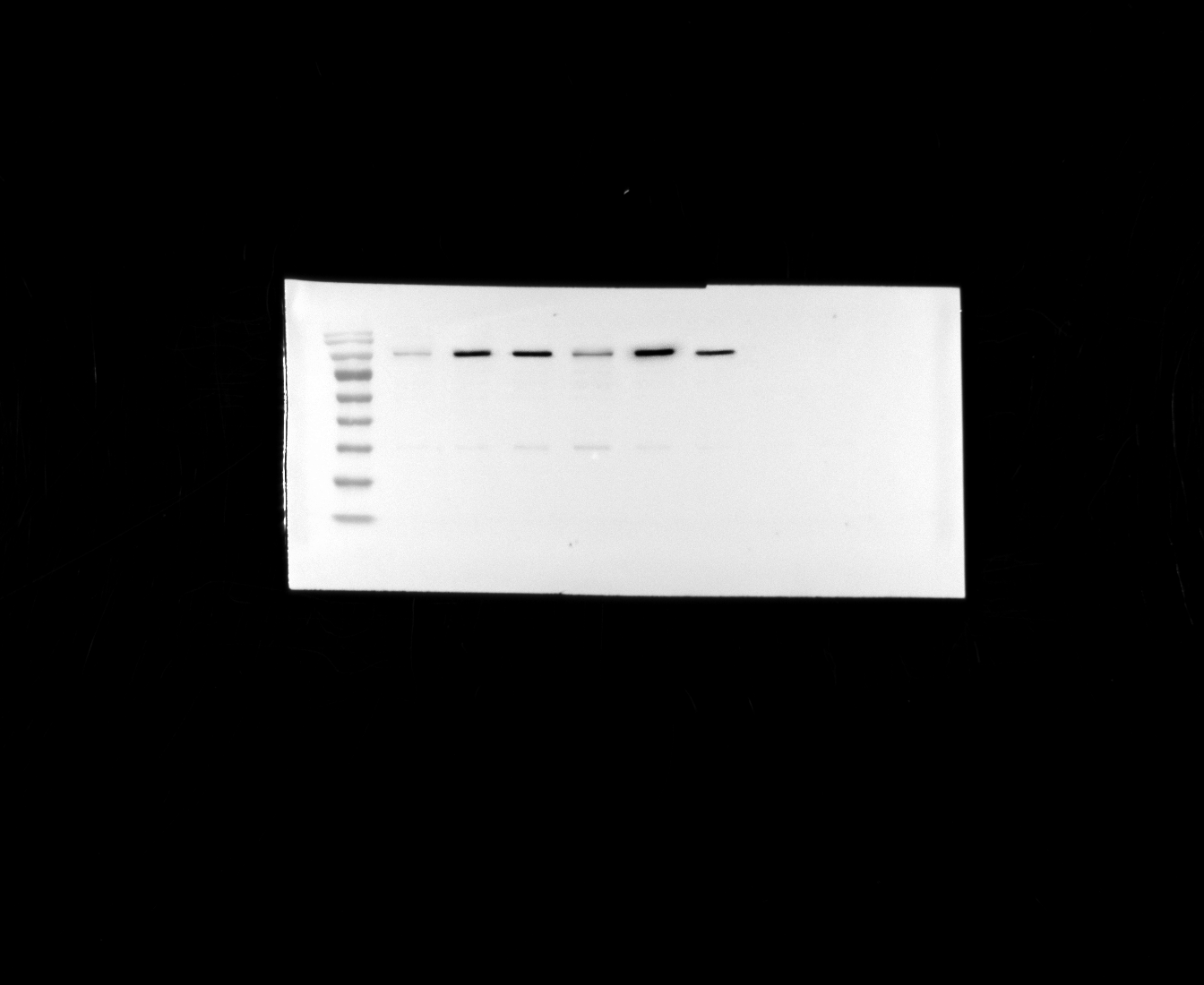

Supplement: Supplementary file 2 — Supplementary Material 2. [file 12935_2025_3665_MOESM2_ESM.zip › Supplementary Material 2/Figure S6/Figure S6G/E-cadherin.tif]

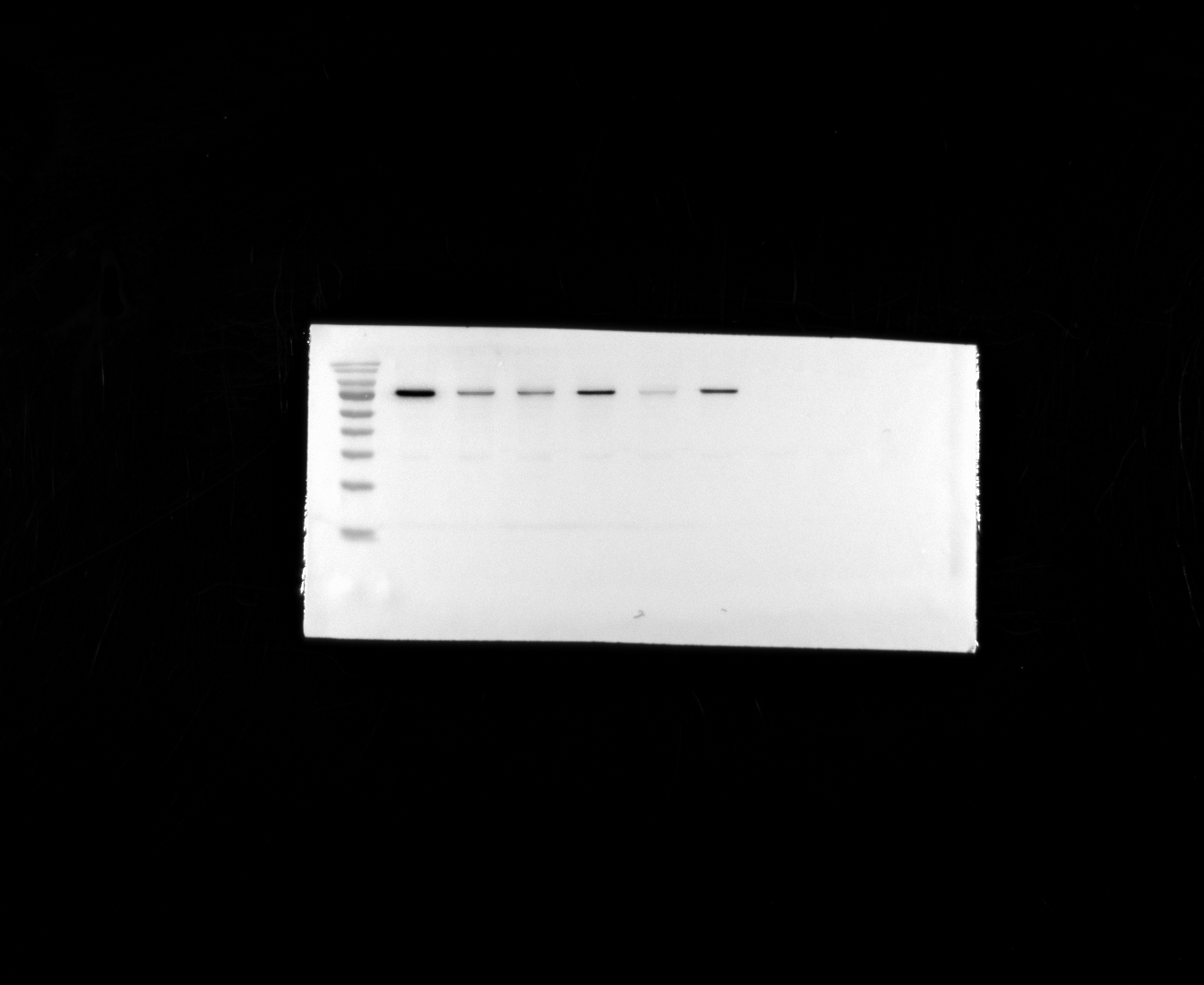

Supplement: Supplementary file 2 — Supplementary Material 2. [file 12935_2025_3665_MOESM2_ESM.zip › Supplementary Material 2/Figure S6/Figure S6G/MMP-2.tif]

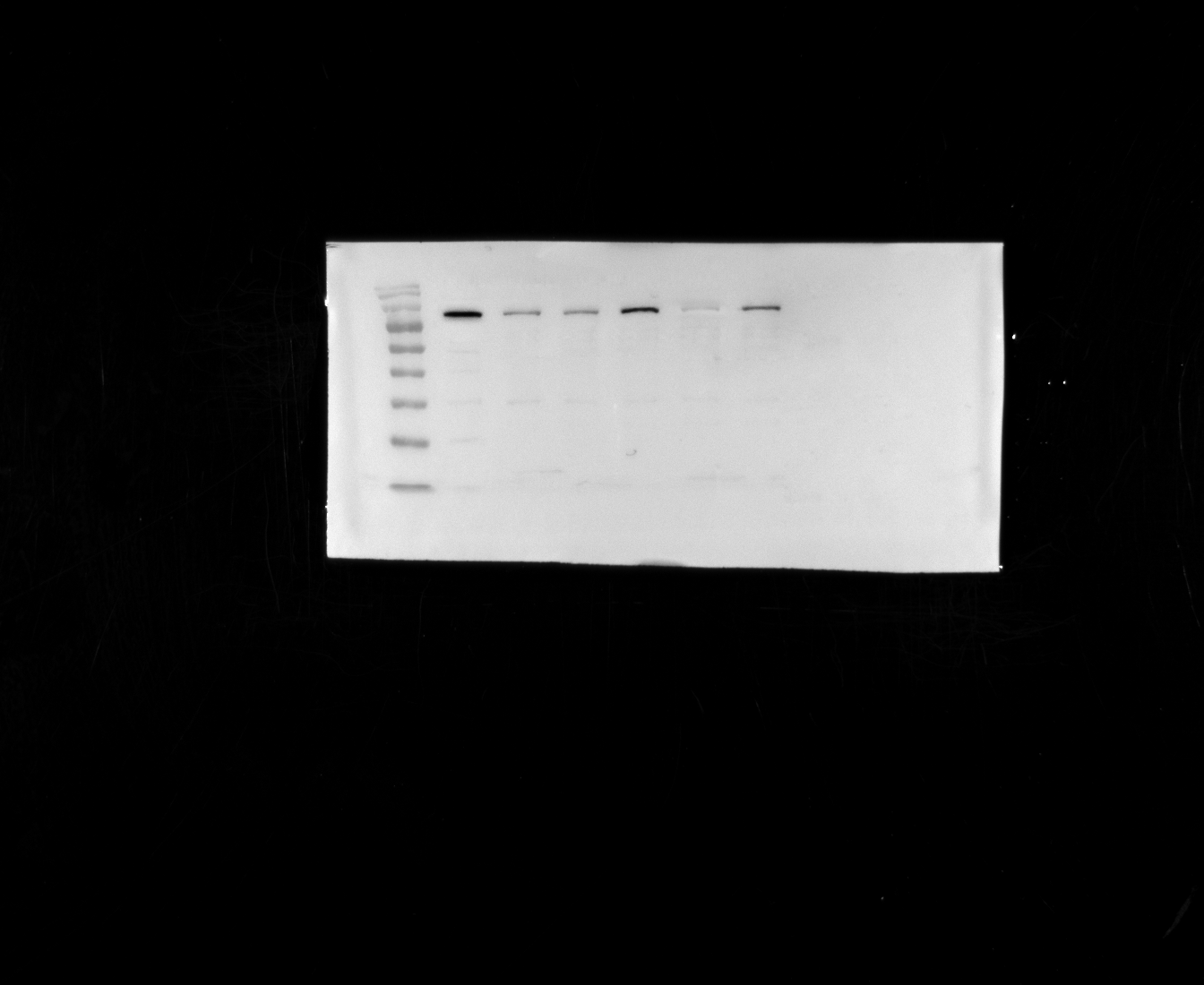

Supplement: Supplementary file 2 — Supplementary Material 2. [file 12935_2025_3665_MOESM2_ESM.zip › Supplementary Material 2/Figure S6/Figure S6G/MMP-9.tif]

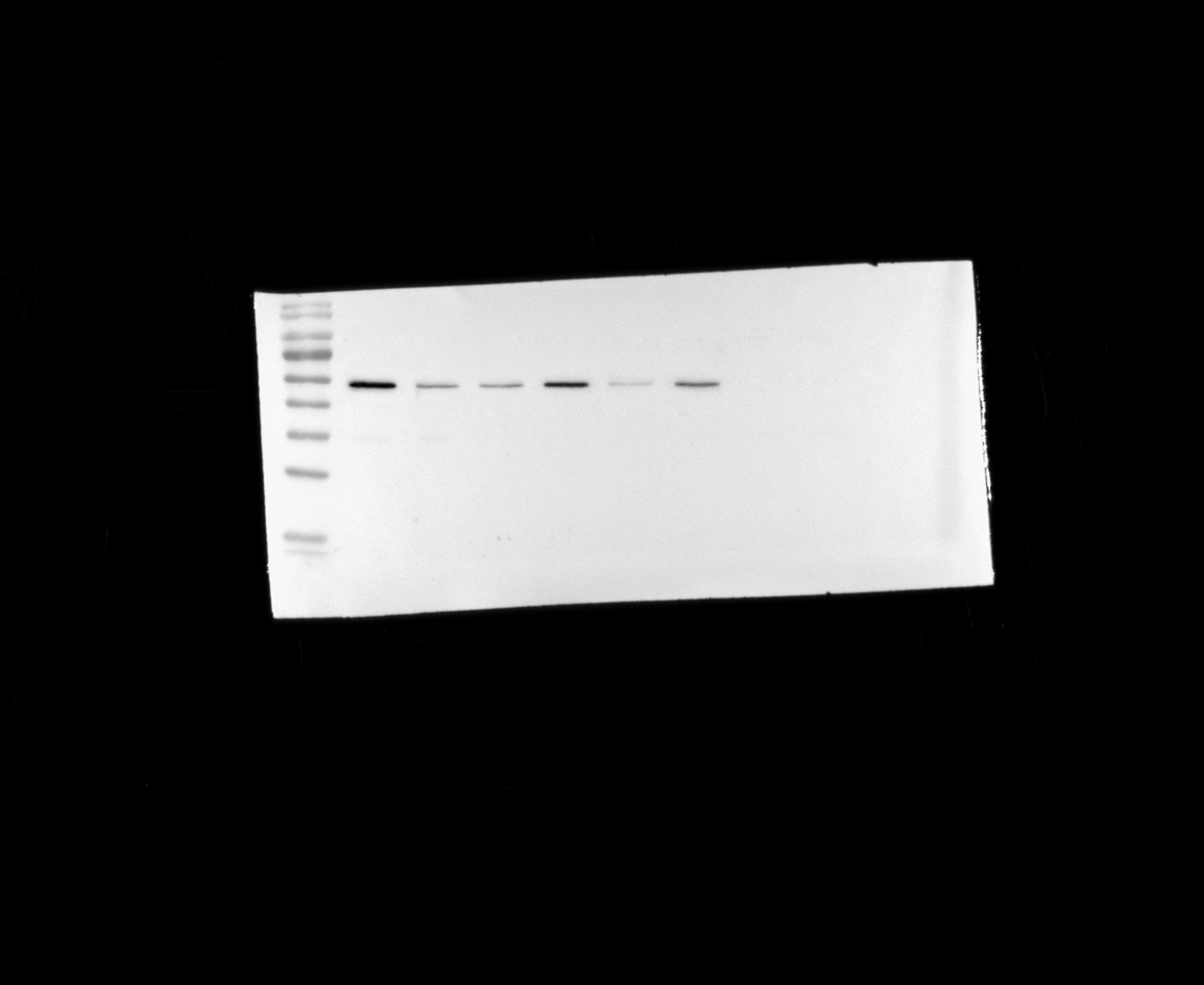

Supplement: Supplementary file 2 — Supplementary Material 2. [file 12935_2025_3665_MOESM2_ESM.zip › Supplementary Material 2/Figure S6/Figure S6G/Vimentin.tif]

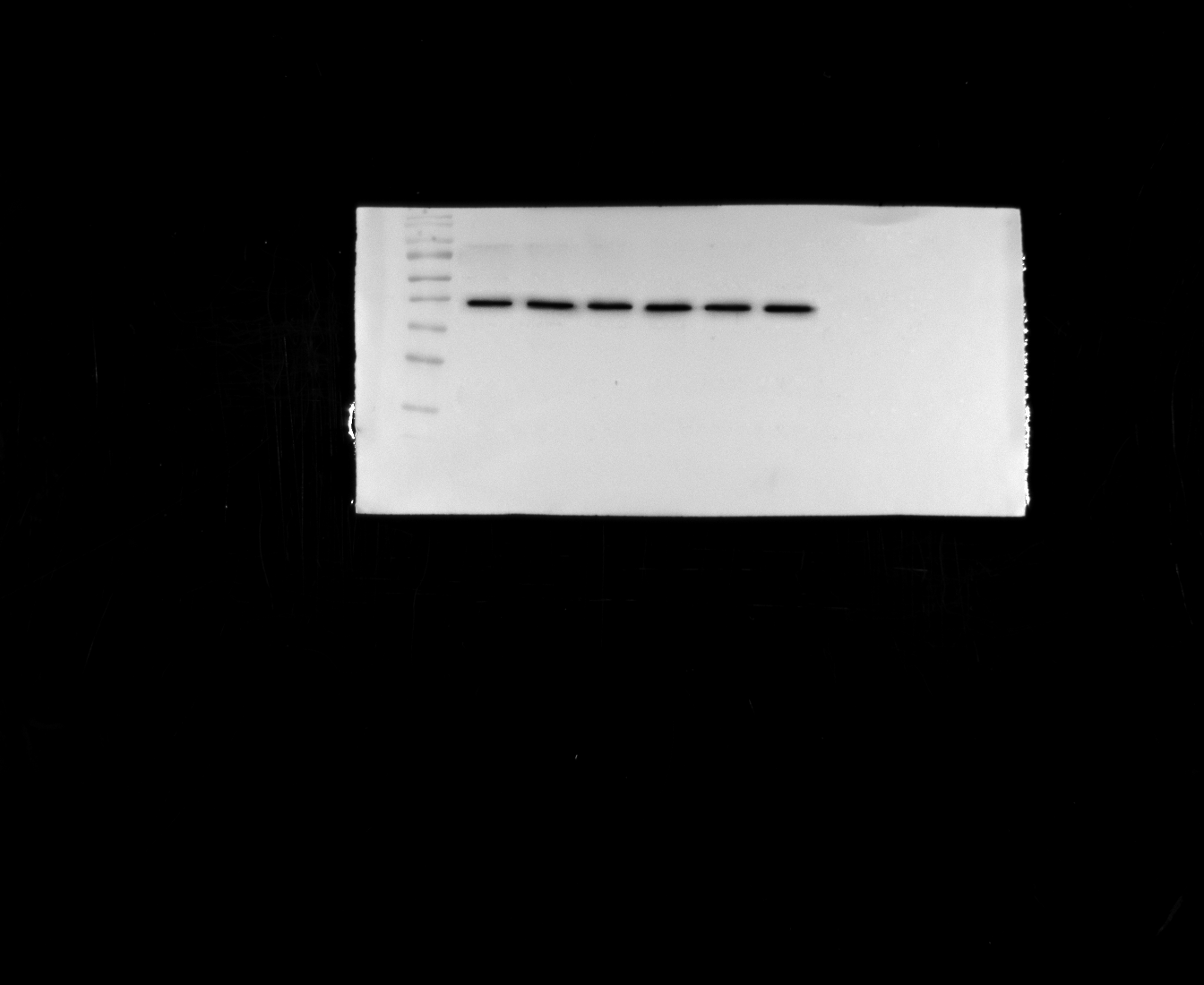

Supplement: Supplementary file 2 — Supplementary Material 2. [file 12935_2025_3665_MOESM2_ESM.zip › Supplementary Material 2/Figure S6/Figure S6G/β-actin.tif]

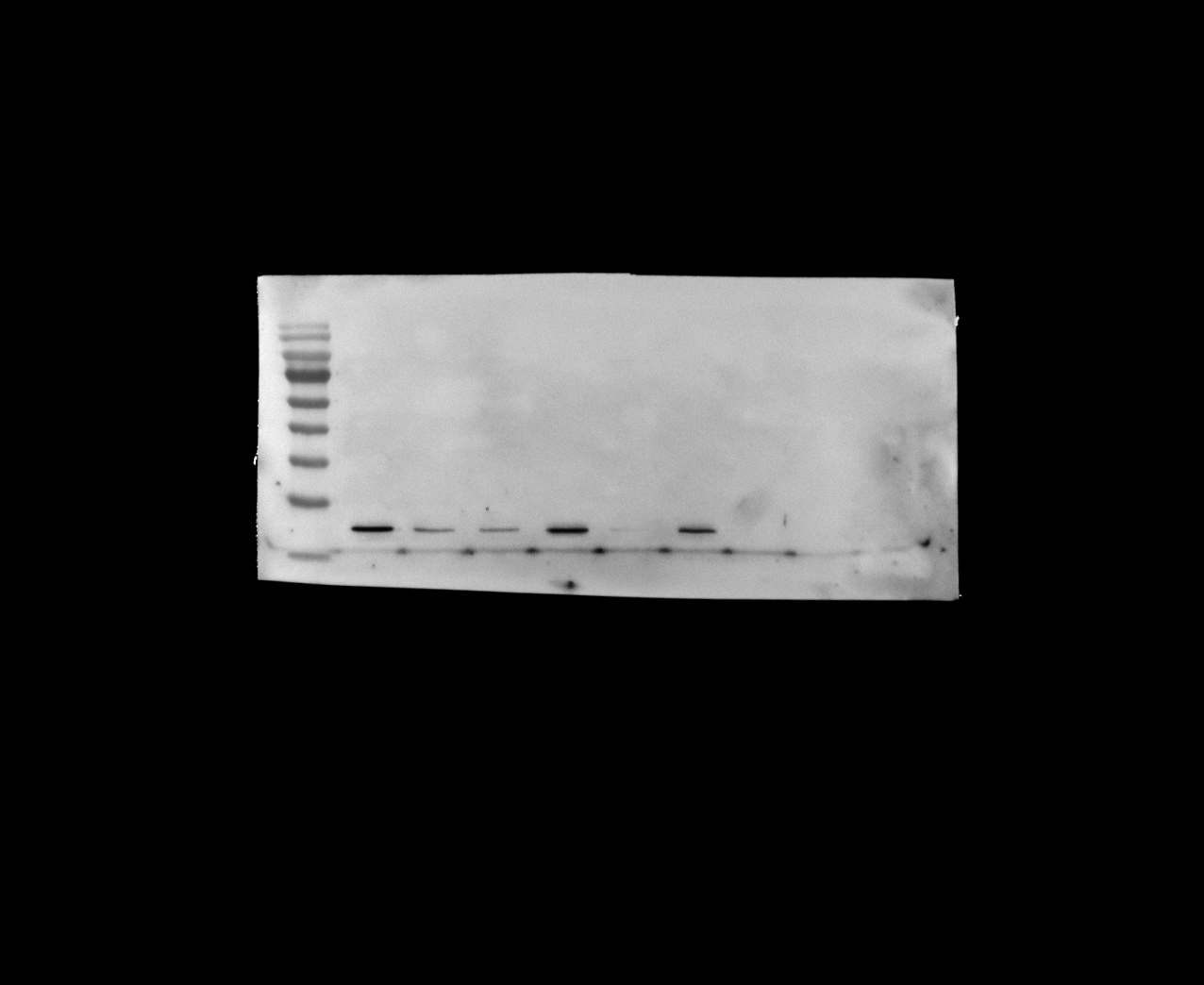

Supplement: Supplementary file 2 — Supplementary Material 2. [file 12935_2025_3665_MOESM2_ESM.zip › Supplementary Material 2/Figure S7/Figure S7B/Ferritin.tif]

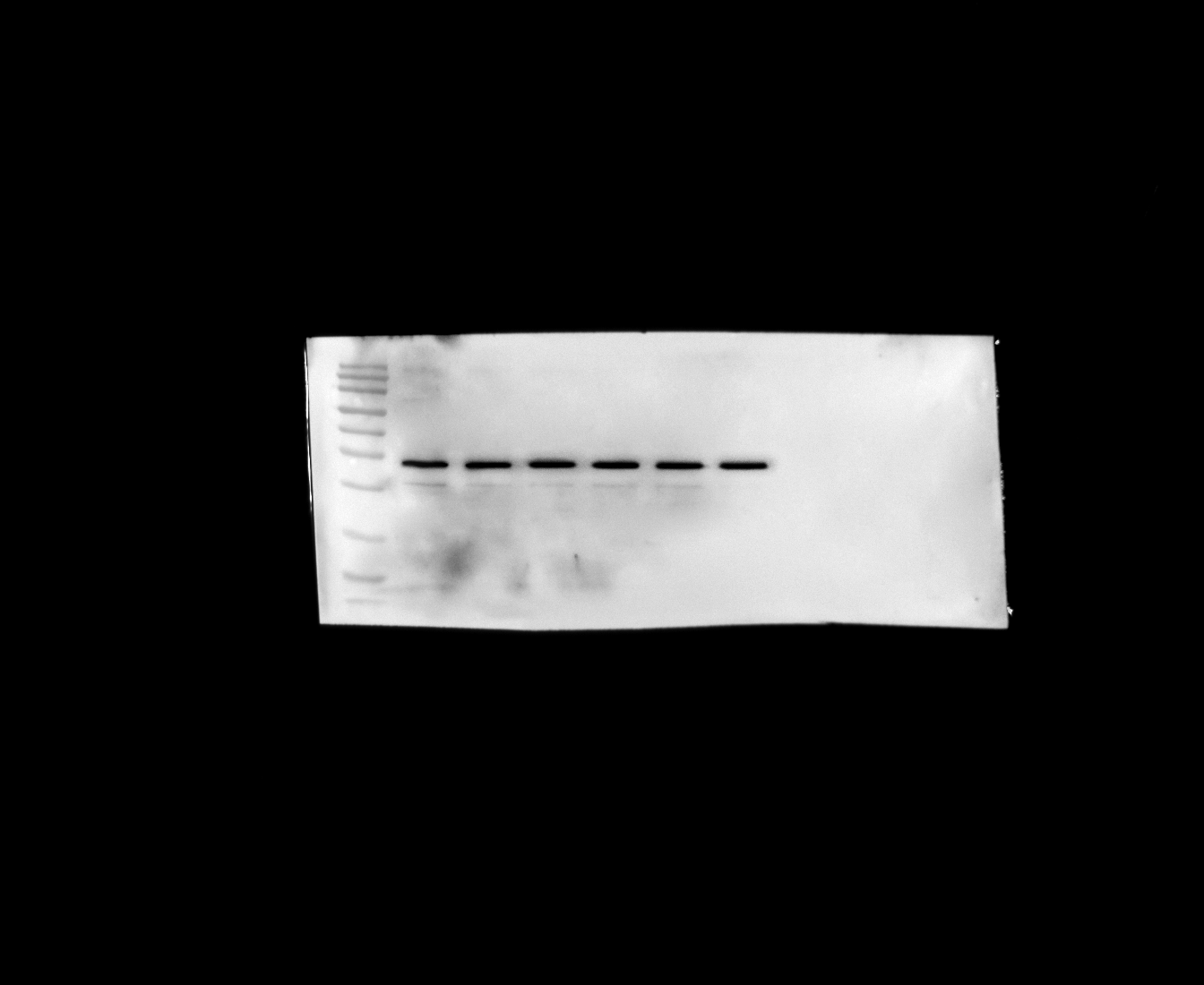

Supplement: Supplementary file 2 — Supplementary Material 2. [file 12935_2025_3665_MOESM2_ESM.zip › Supplementary Material 2/Figure S7/Figure S7B/β-actin.tif]
