# Supplementary figures and images for: Comprehensive network pharmacology and experimental study to investigate the effect and mechanism of solasonine on breast carcinoma treatment (part 1 of 2)
Source: Cancer Cell Int. 2025 Feb 17;25:49. doi: 10.1186/s12935-025-03665-6 (PMC11834262; doi:10.1186/s12935-025-03665-6)

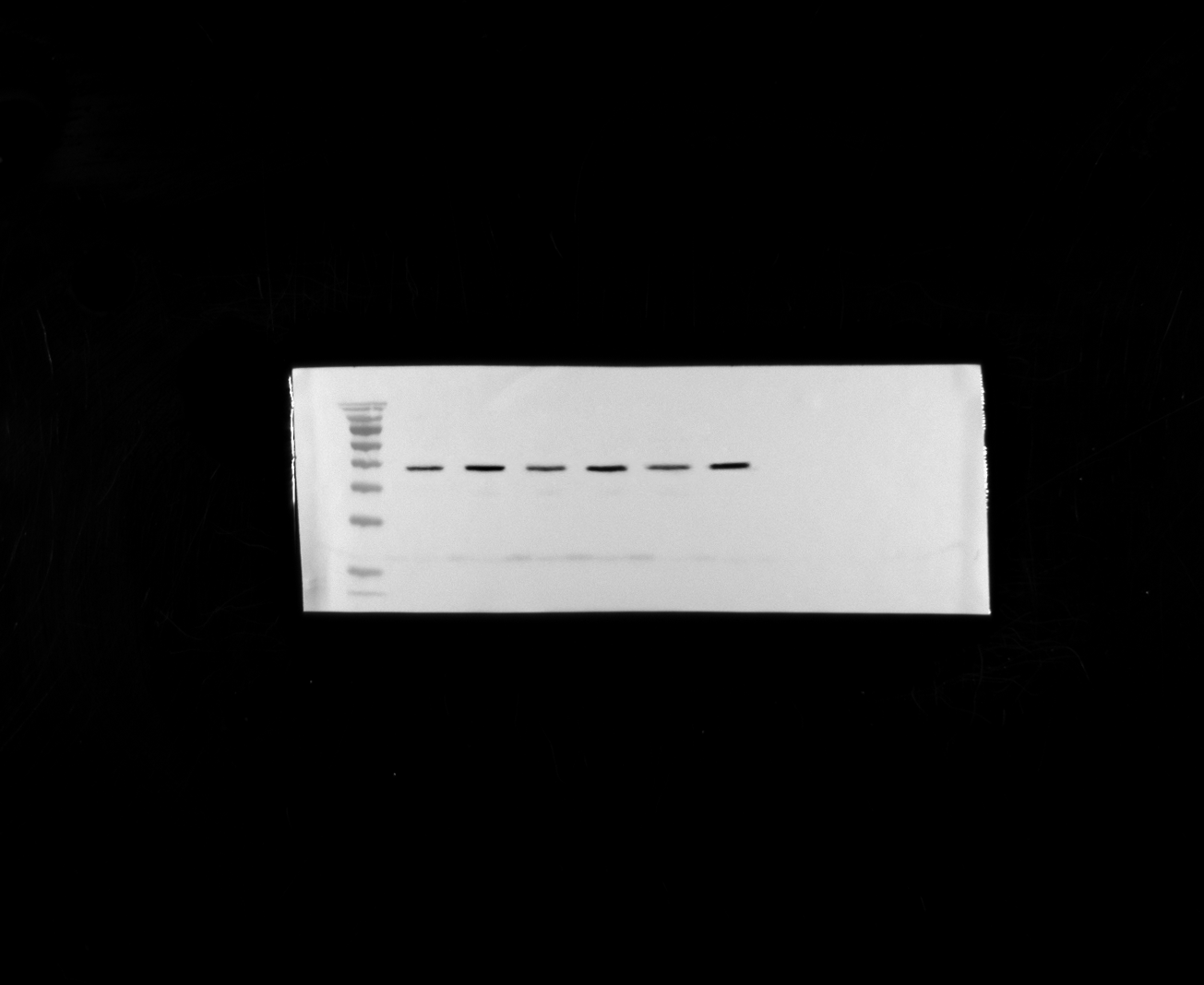

Supplement: Supplementary file 2 — Supplementary Material 2. [file 12935_2025_3665_MOESM2_ESM.zip › Supplementary Material 2/Figure 2/Figure 2C/1/ERK2.tif]

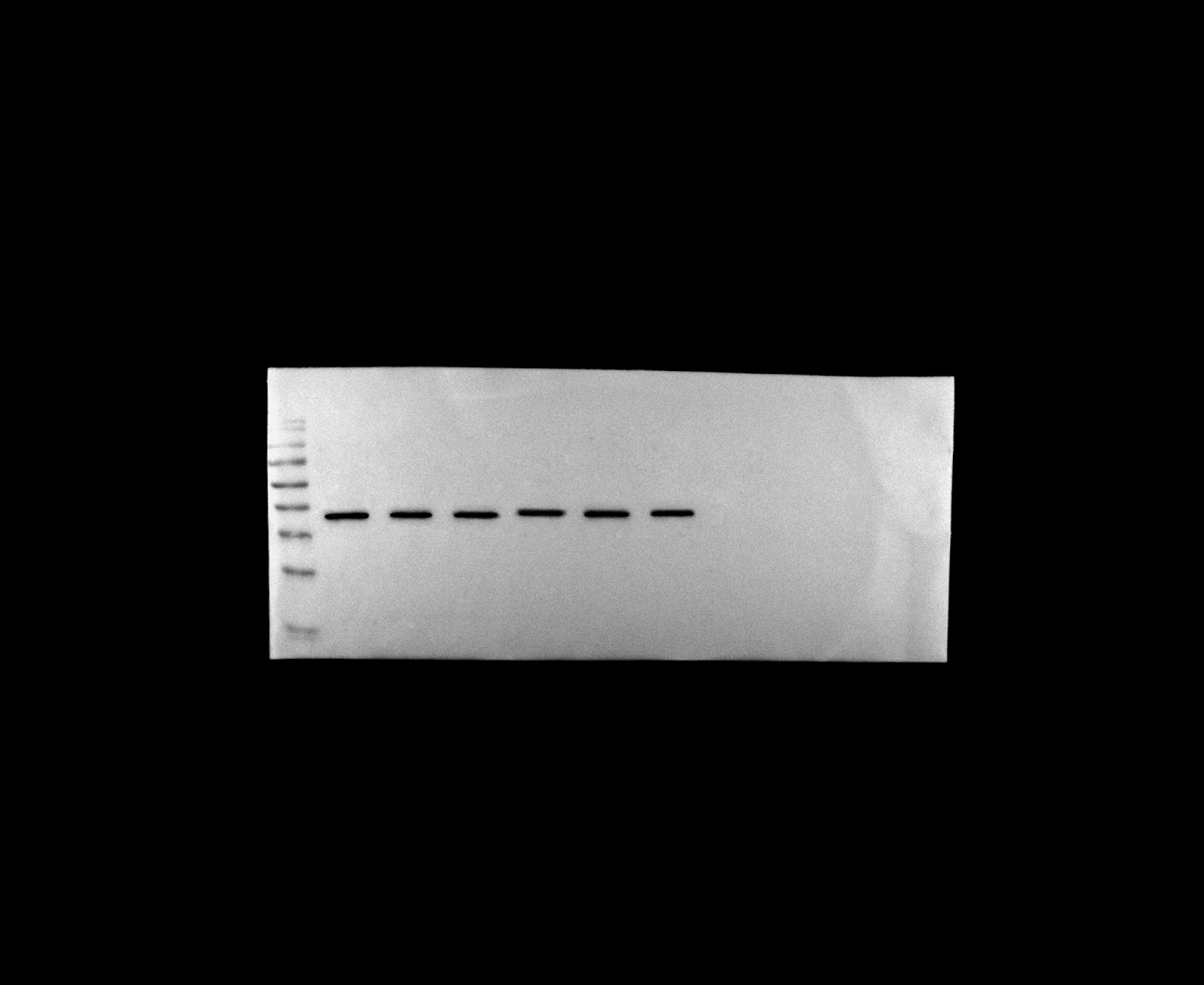

Supplement: Supplementary file 2 — Supplementary Material 2. [file 12935_2025_3665_MOESM2_ESM.zip › Supplementary Material 2/Figure 2/Figure 2C/1/GAPDH.tif]

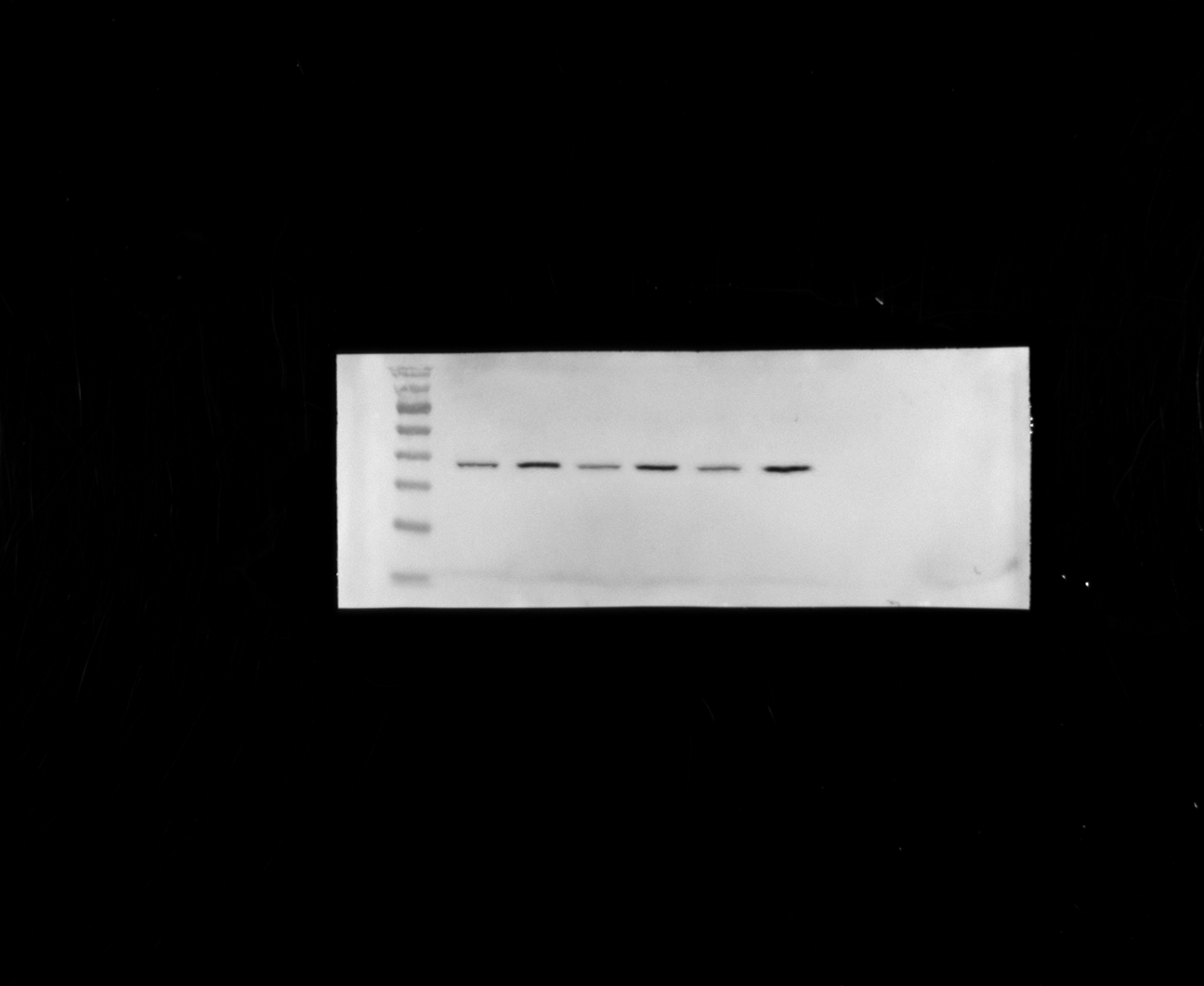

Supplement: Supplementary file 2 — Supplementary Material 2. [file 12935_2025_3665_MOESM2_ESM.zip › Supplementary Material 2/Figure 2/Figure 2C/1/p-ERK2(T185).tif]

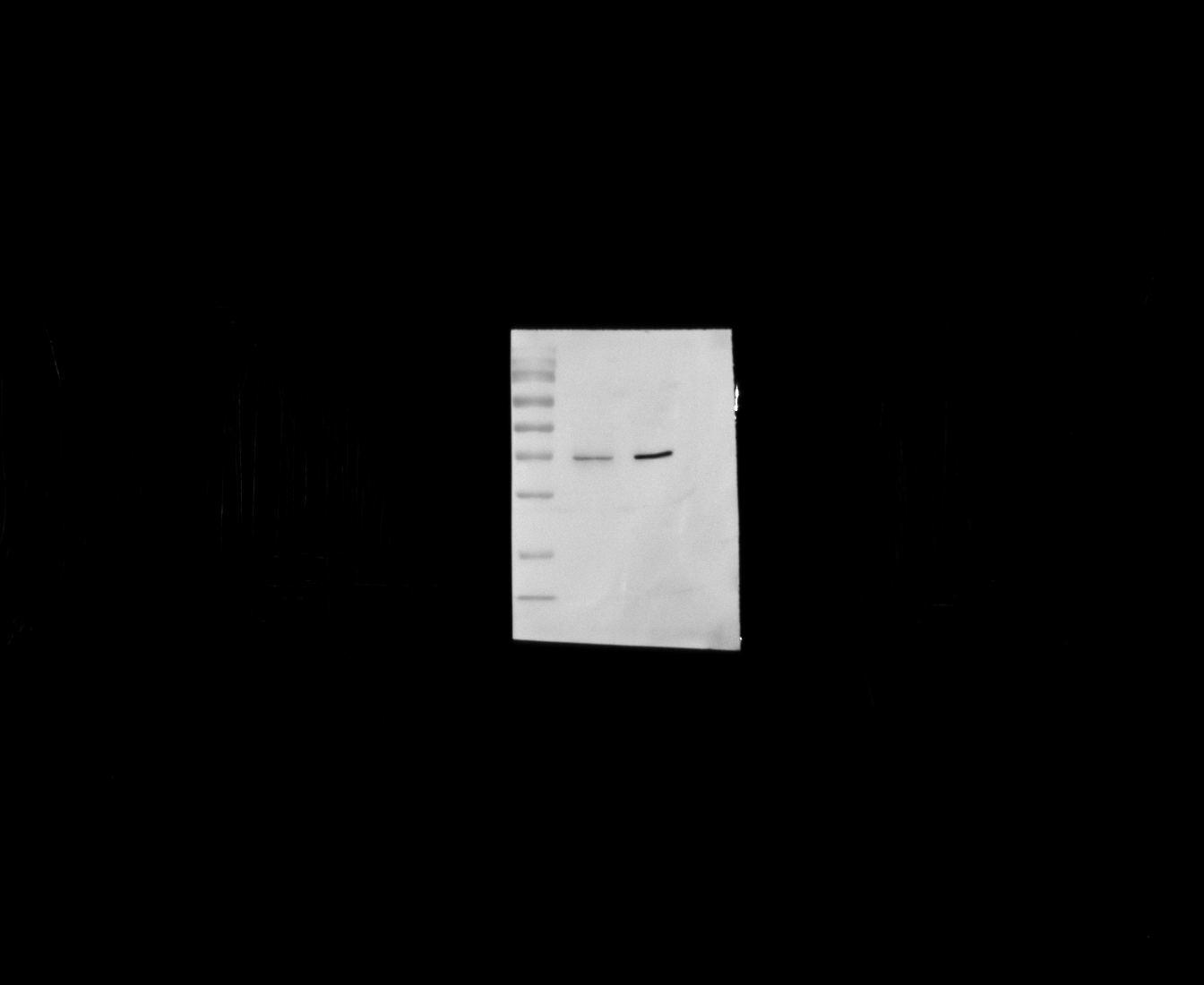

Supplement: Supplementary file 2 — Supplementary Material 2. [file 12935_2025_3665_MOESM2_ESM.zip › Supplementary Material 2/Figure 2/Figure 2C/2/ERK2.tif]

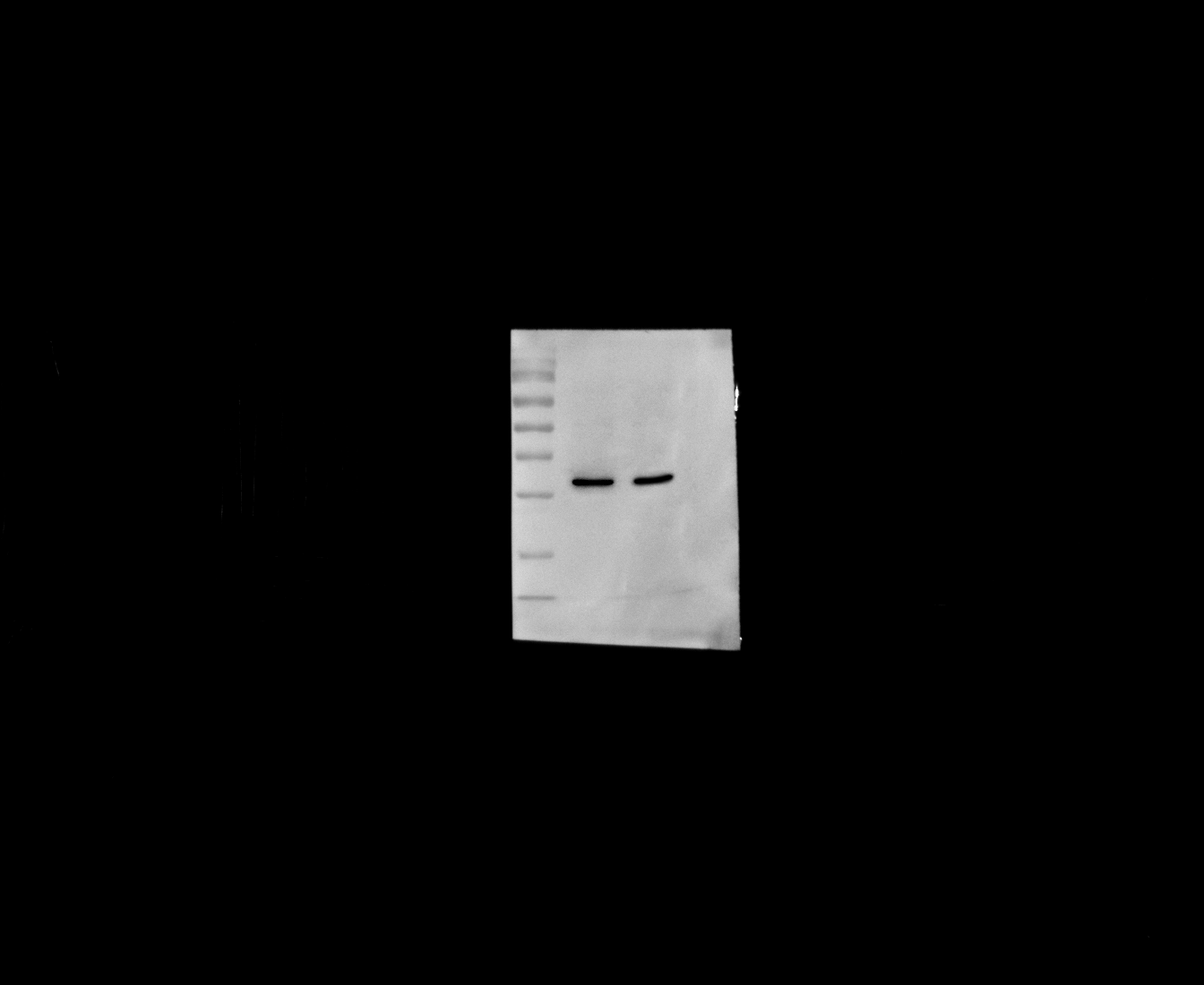

Supplement: Supplementary file 2 — Supplementary Material 2. [file 12935_2025_3665_MOESM2_ESM.zip › Supplementary Material 2/Figure 2/Figure 2C/2/GAPDH.tif]

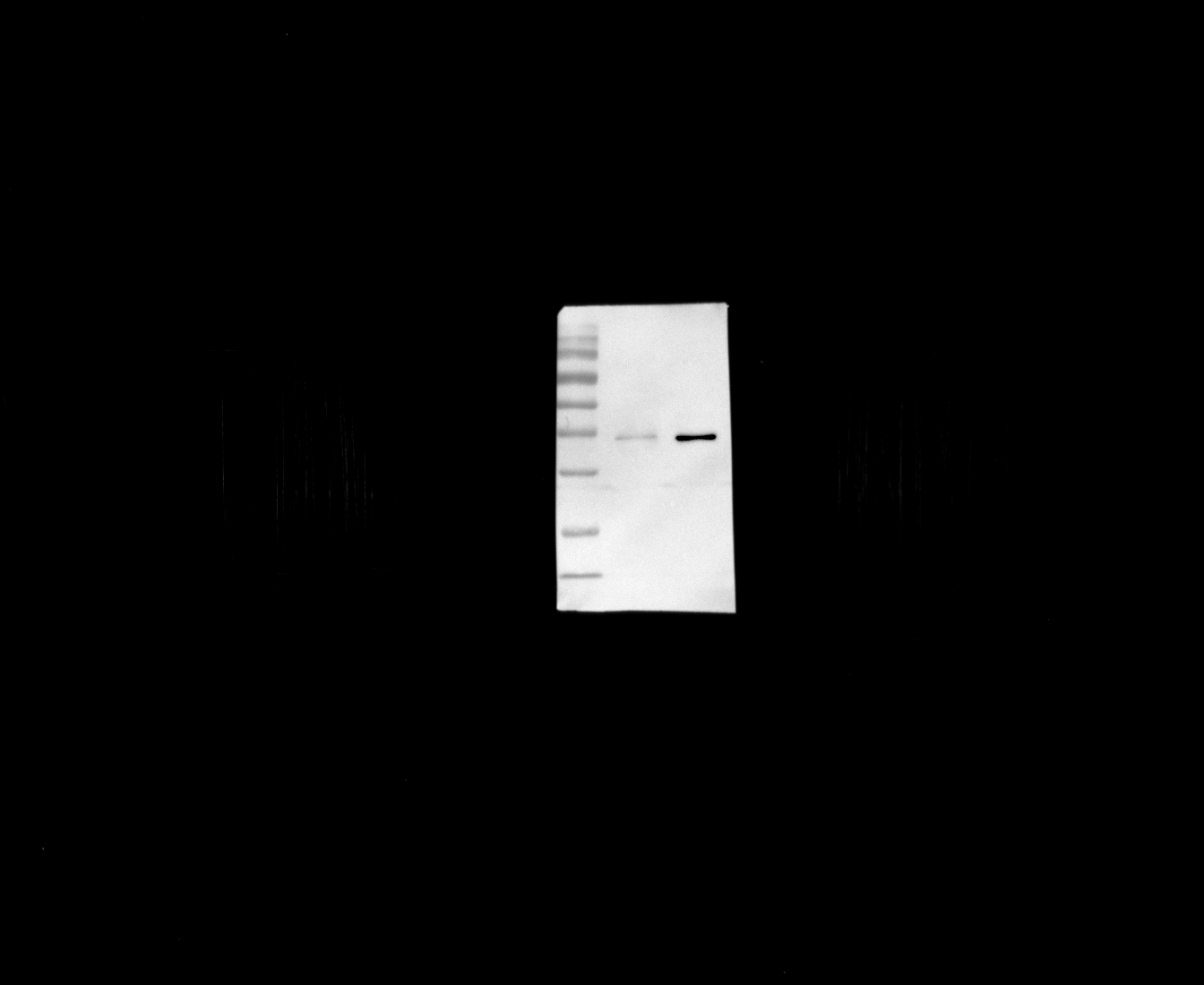

Supplement: Supplementary file 2 — Supplementary Material 2. [file 12935_2025_3665_MOESM2_ESM.zip › Supplementary Material 2/Figure 2/Figure 2C/2/p-ERK2(T185).tif]

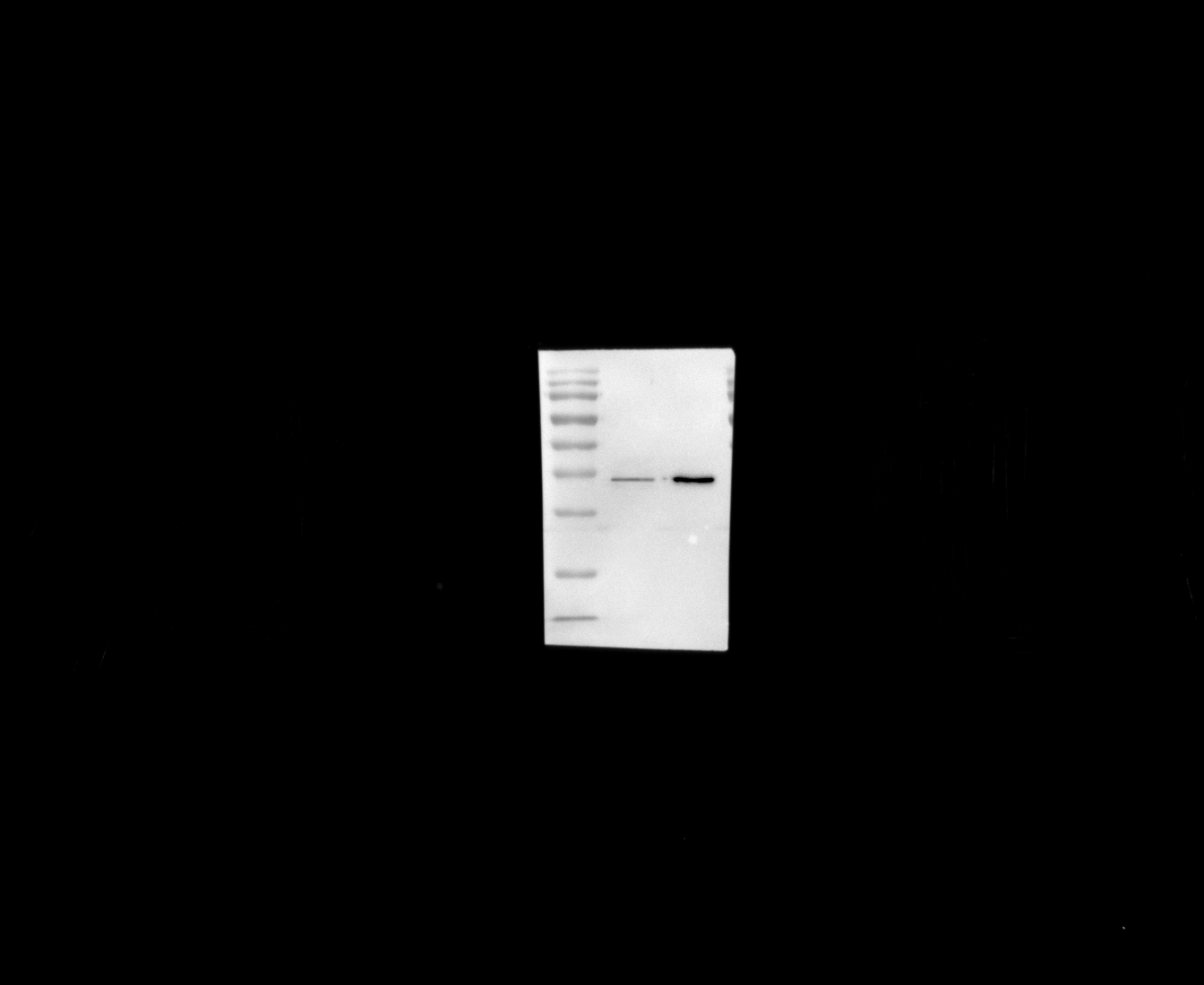

Supplement: Supplementary file 2 — Supplementary Material 2. [file 12935_2025_3665_MOESM2_ESM.zip › Supplementary Material 2/Figure 2/Figure 2C/3/ERK2.tif]

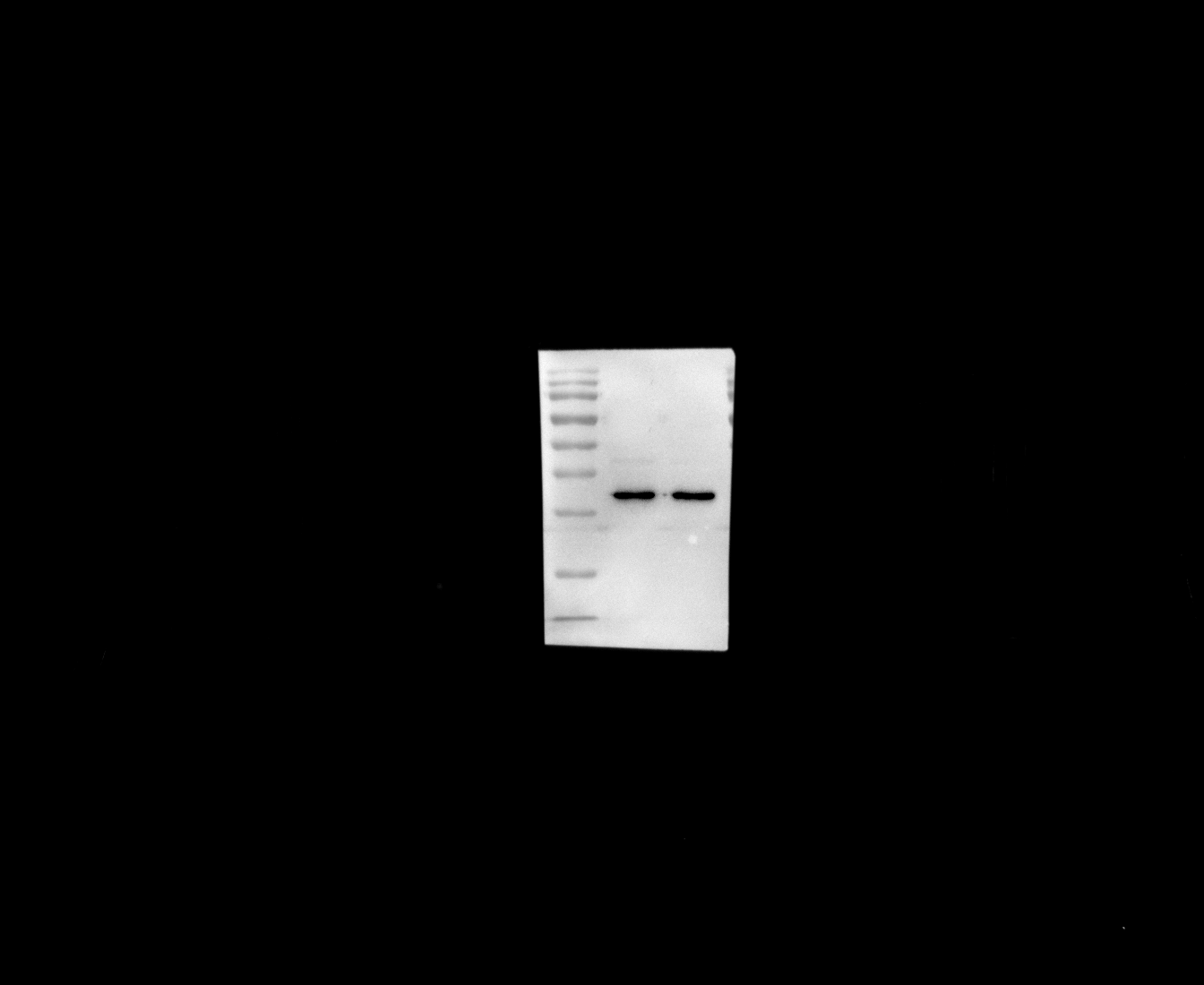

Supplement: Supplementary file 2 — Supplementary Material 2. [file 12935_2025_3665_MOESM2_ESM.zip › Supplementary Material 2/Figure 2/Figure 2C/3/GAPDH.tif]

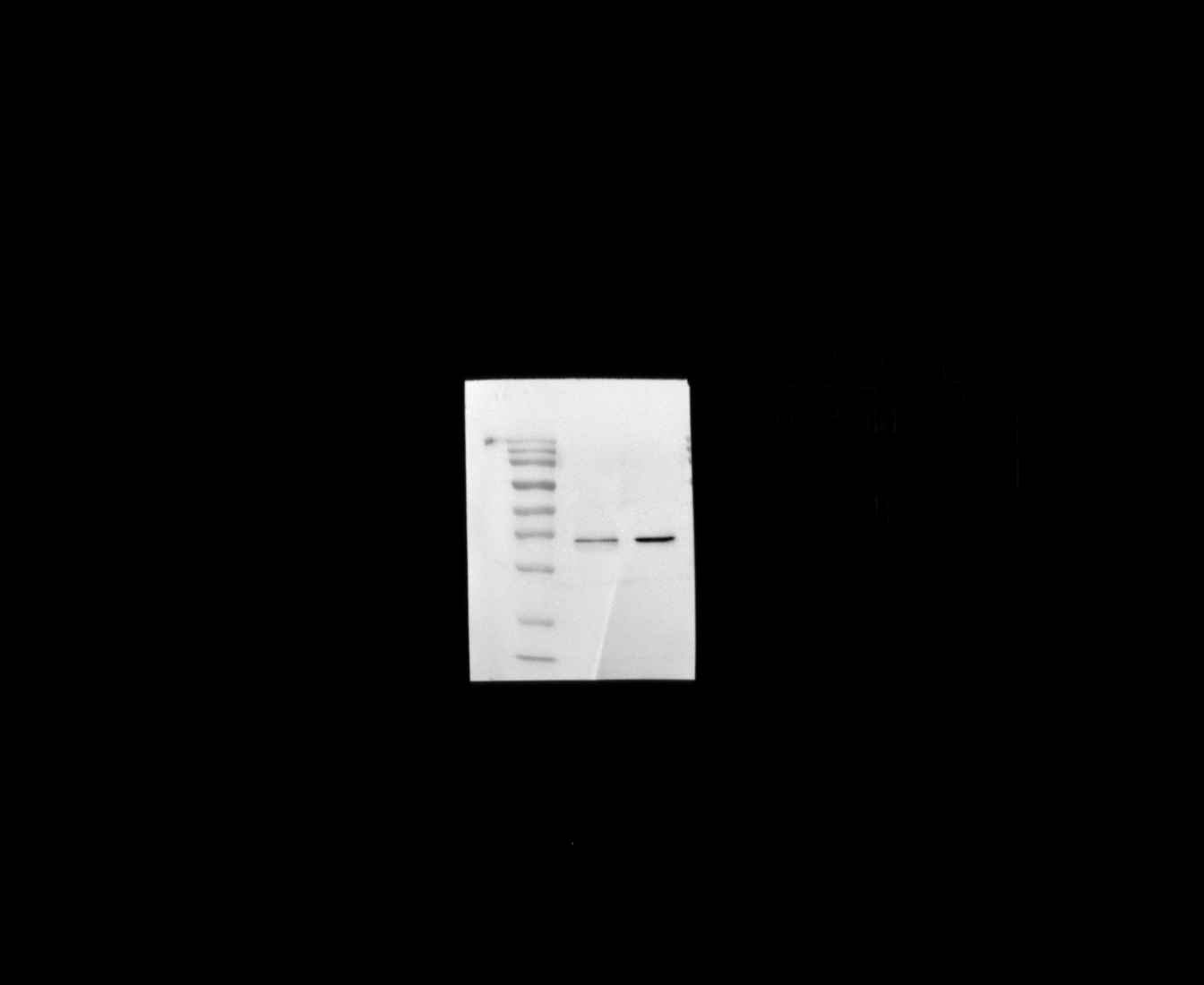

Supplement: Supplementary file 2 — Supplementary Material 2. [file 12935_2025_3665_MOESM2_ESM.zip › Supplementary Material 2/Figure 2/Figure 2C/3/p-ERK2(T185).tif]

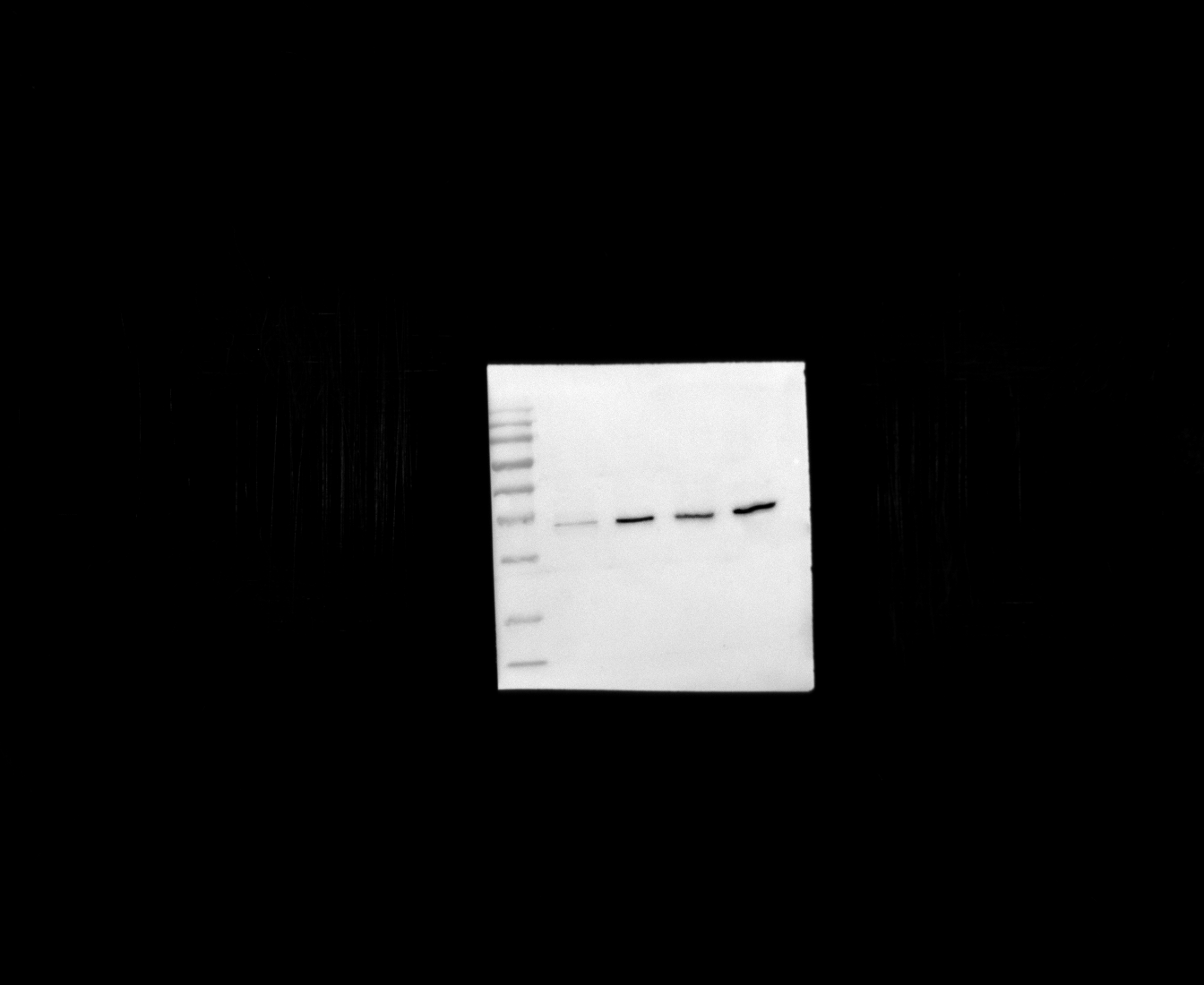

Supplement: Supplementary file 2 — Supplementary Material 2. [file 12935_2025_3665_MOESM2_ESM.zip › Supplementary Material 2/Figure 2/Figure 2C/4/ERK2.tif]

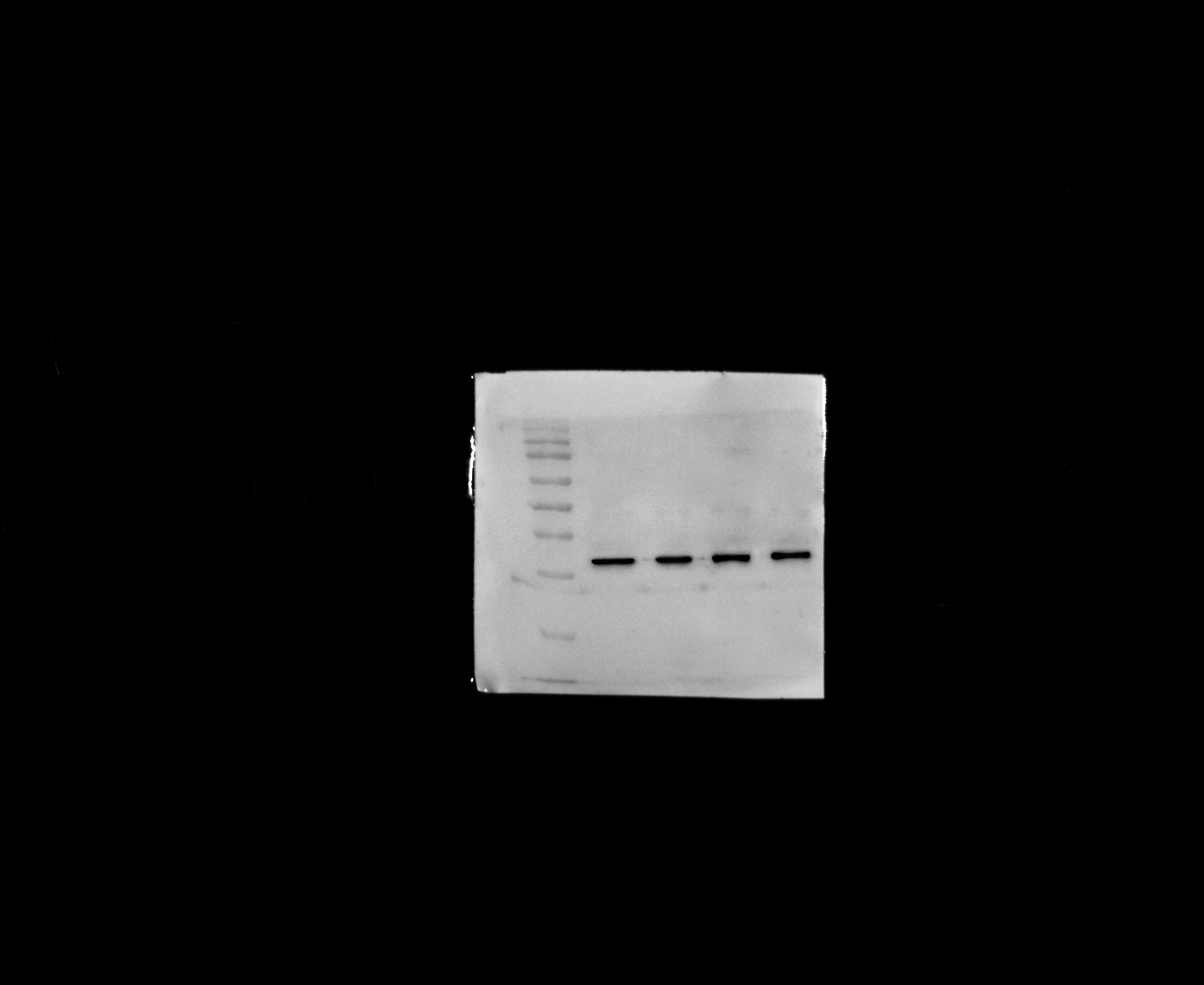

Supplement: Supplementary file 2 — Supplementary Material 2. [file 12935_2025_3665_MOESM2_ESM.zip › Supplementary Material 2/Figure 2/Figure 2C/4/GAPDH.tif]

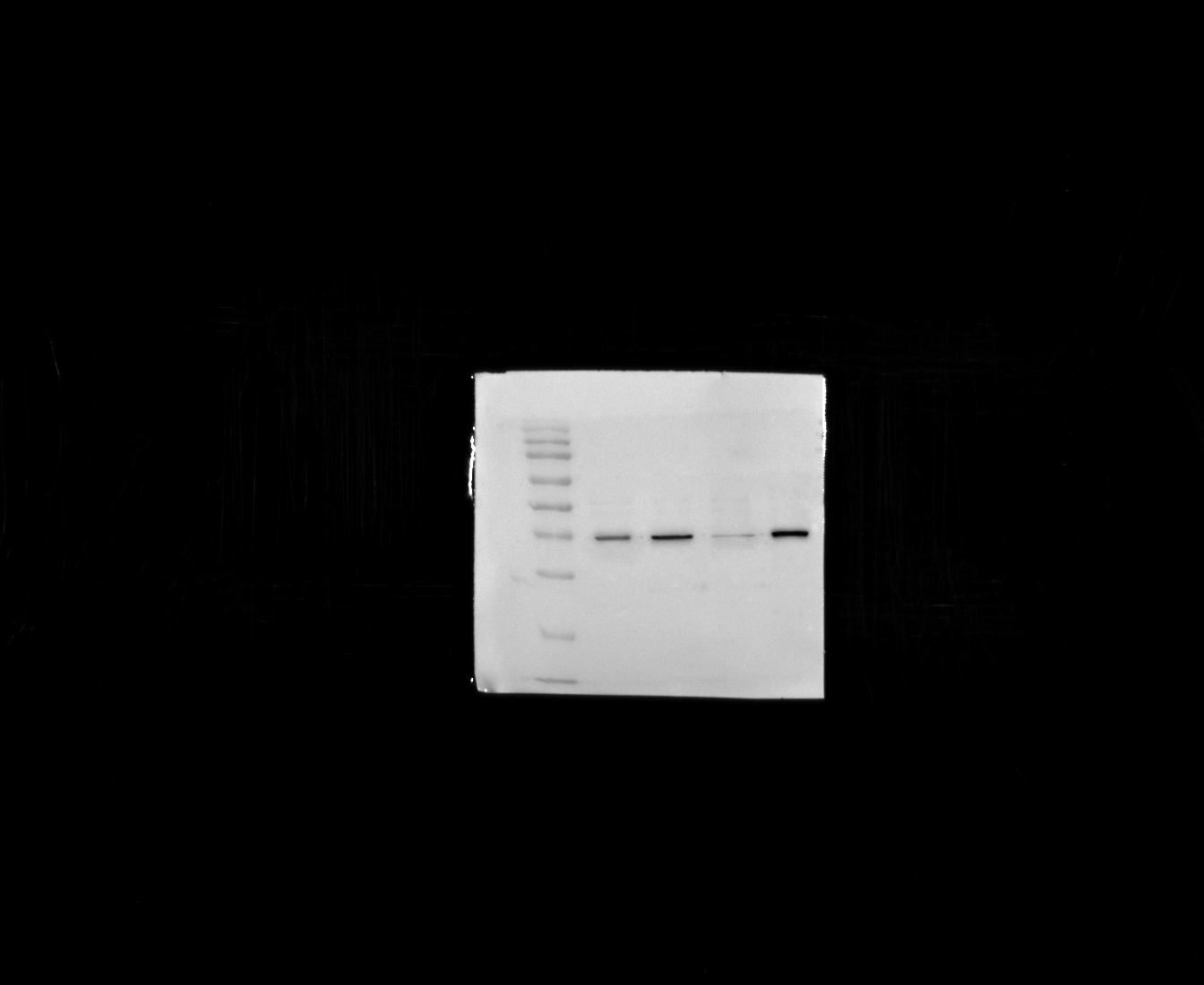

Supplement: Supplementary file 2 — Supplementary Material 2. [file 12935_2025_3665_MOESM2_ESM.zip › Supplementary Material 2/Figure 2/Figure 2C/4/p-ERK2(T185).tif]

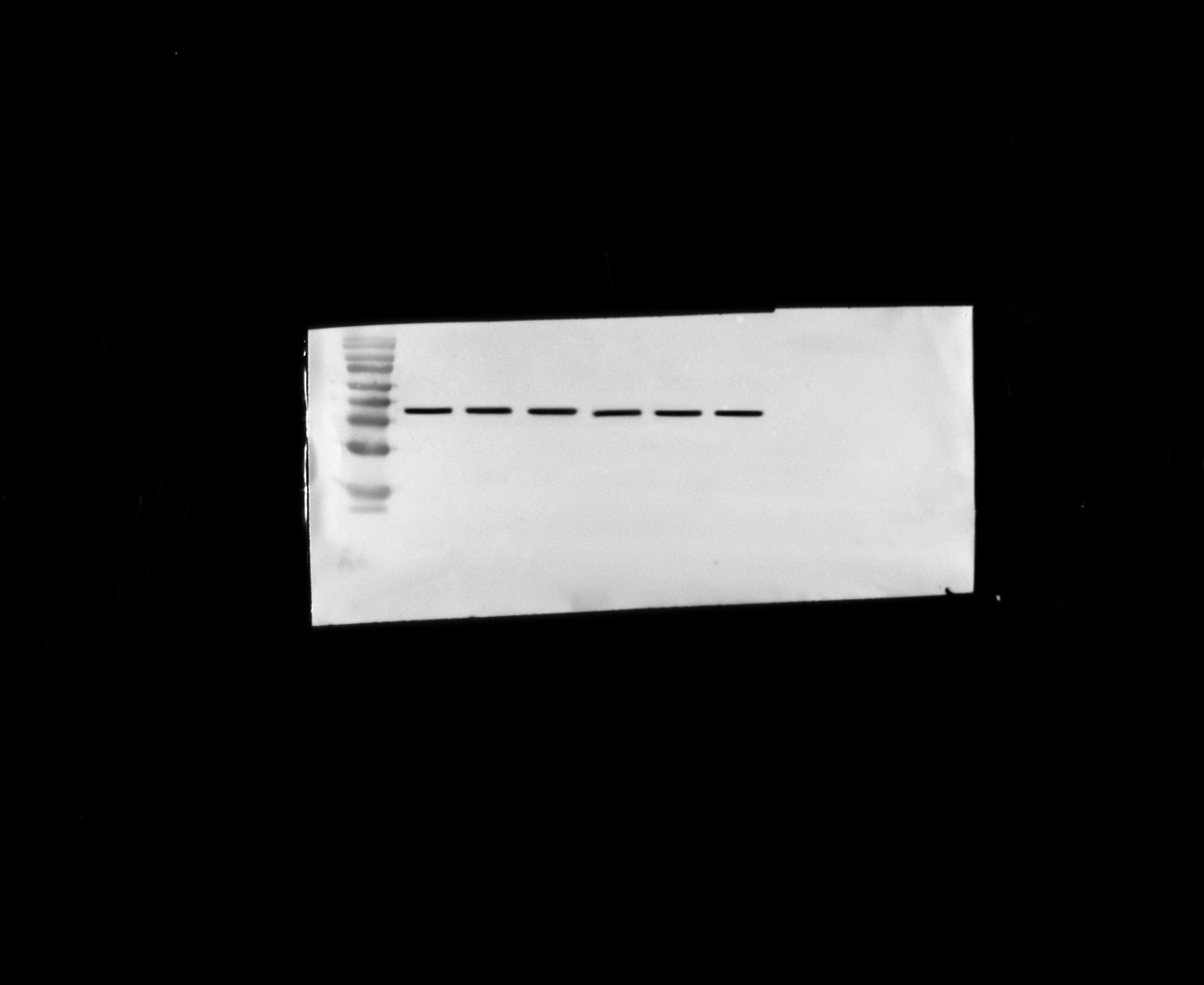

Supplement: Supplementary file 2 — Supplementary Material 2. [file 12935_2025_3665_MOESM2_ESM.zip › Supplementary Material 2/Figure 2/Figure 2E/1/GAPDH.tif]

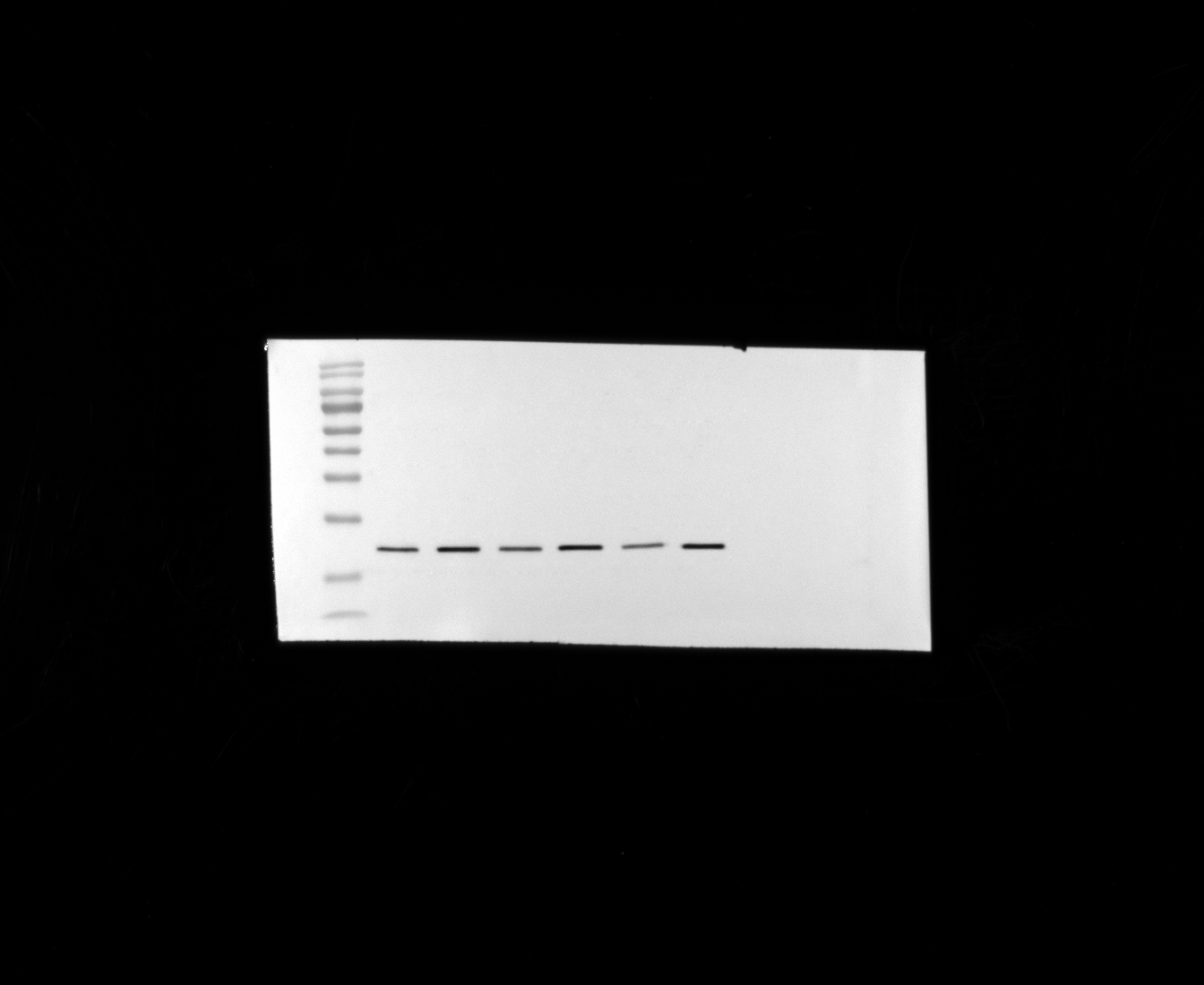

Supplement: Supplementary file 2 — Supplementary Material 2. [file 12935_2025_3665_MOESM2_ESM.zip › Supplementary Material 2/Figure 2/Figure 2E/1/GPX4.tif]

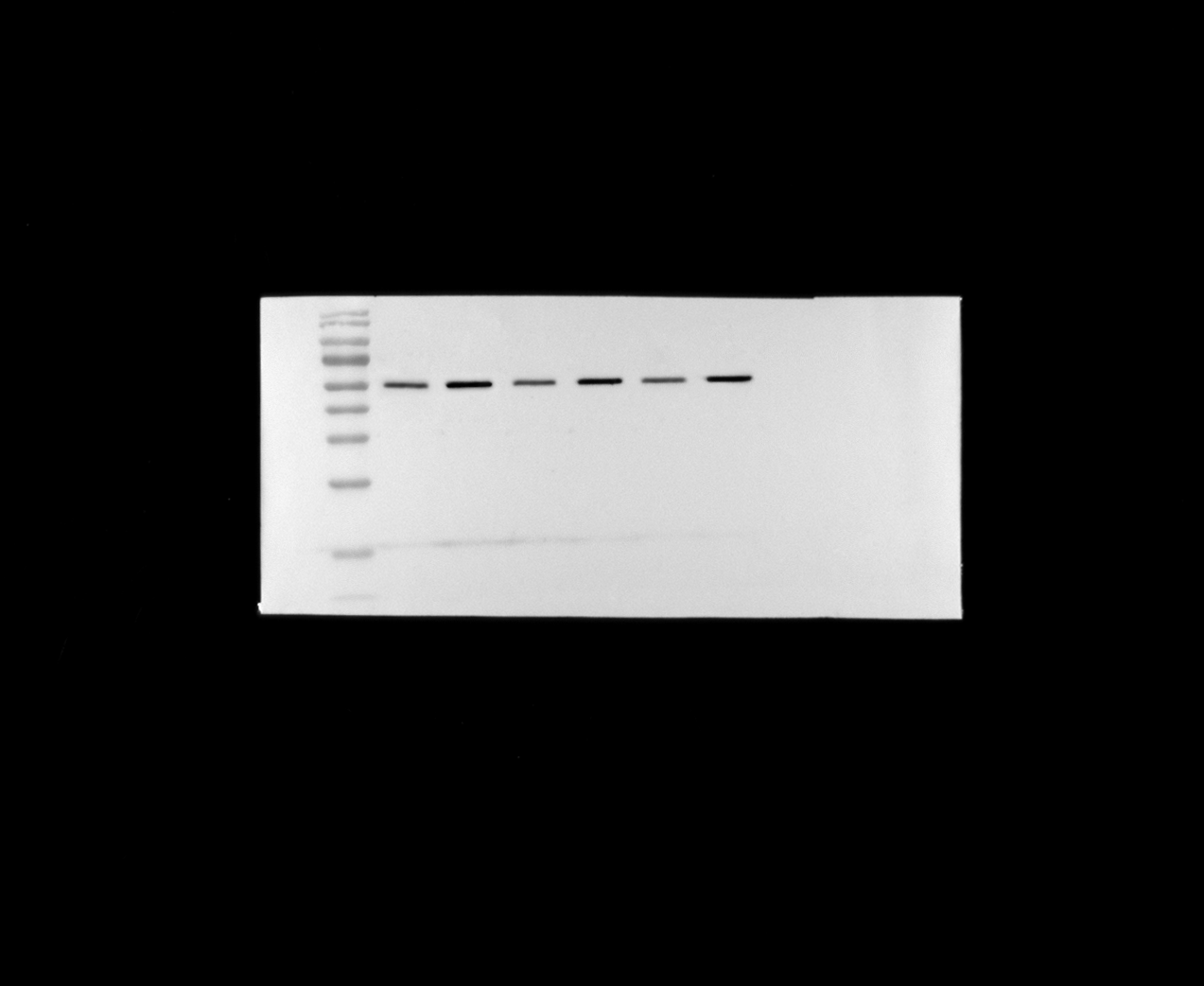

Supplement: Supplementary file 2 — Supplementary Material 2. [file 12935_2025_3665_MOESM2_ESM.zip › Supplementary Material 2/Figure 2/Figure 2E/1/SLC7A11.tif]

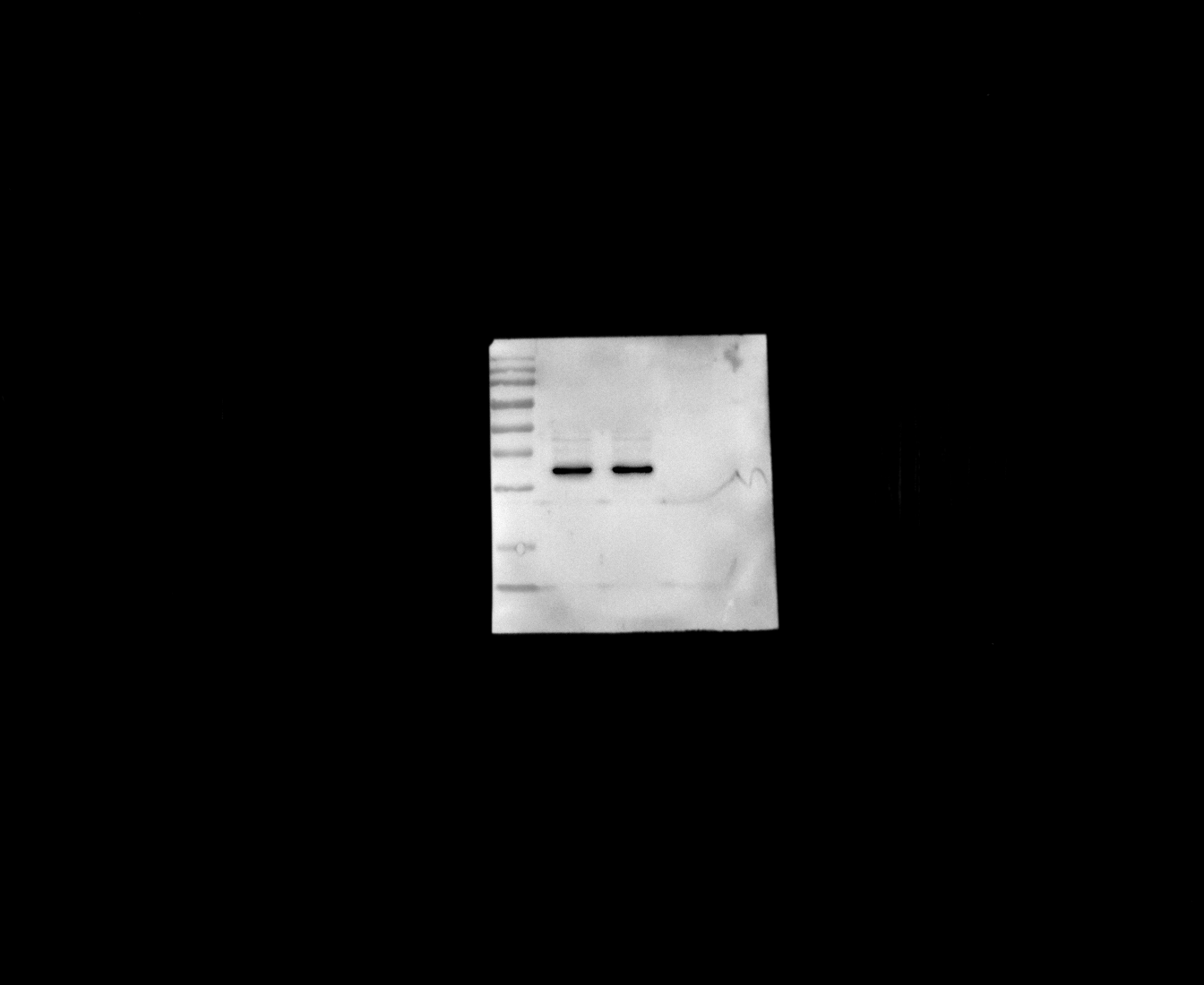

Supplement: Supplementary file 2 — Supplementary Material 2. [file 12935_2025_3665_MOESM2_ESM.zip › Supplementary Material 2/Figure 2/Figure 2E/2/GAPDH.tif]

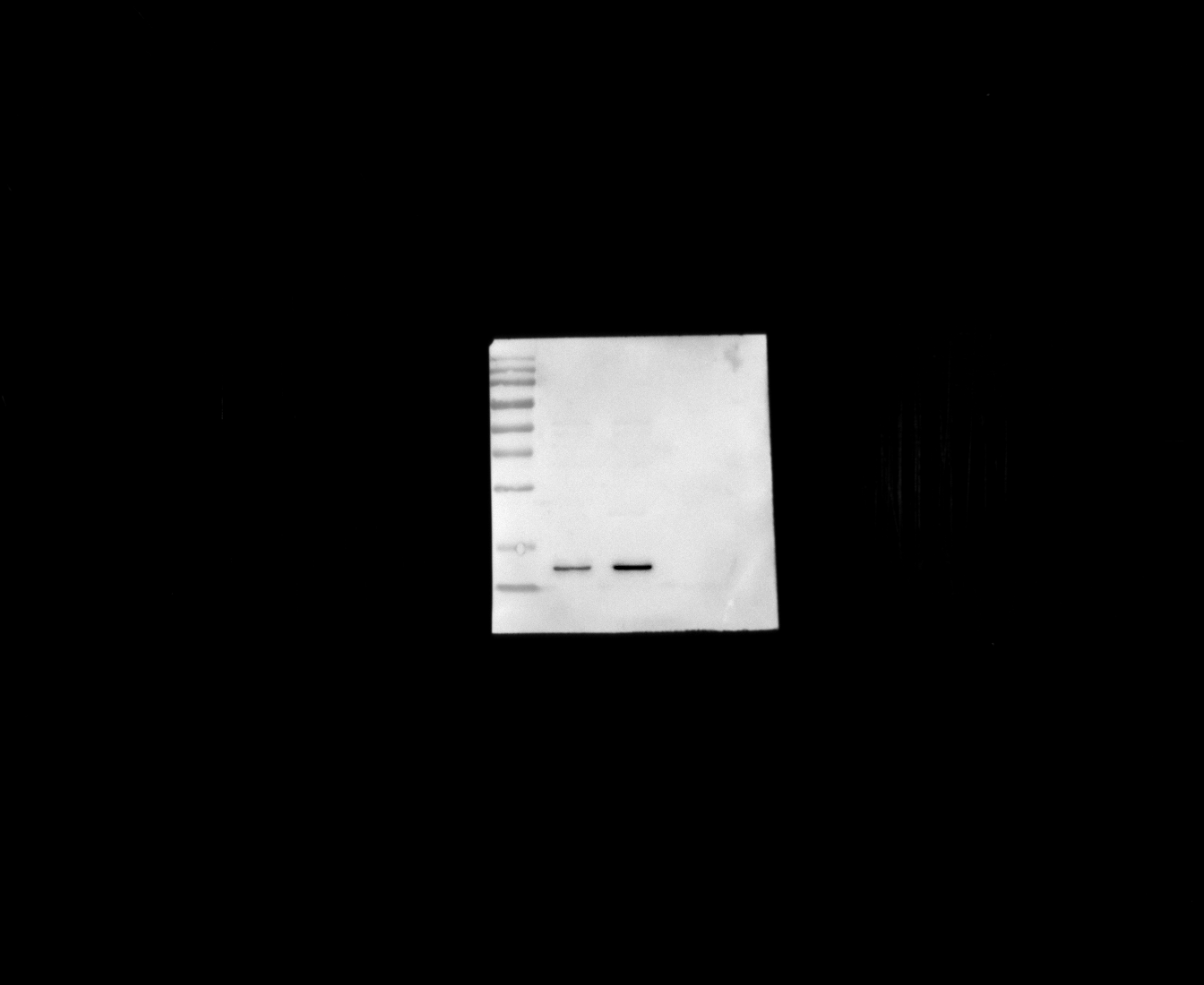

Supplement: Supplementary file 2 — Supplementary Material 2. [file 12935_2025_3665_MOESM2_ESM.zip › Supplementary Material 2/Figure 2/Figure 2E/2/GPX4.tif]

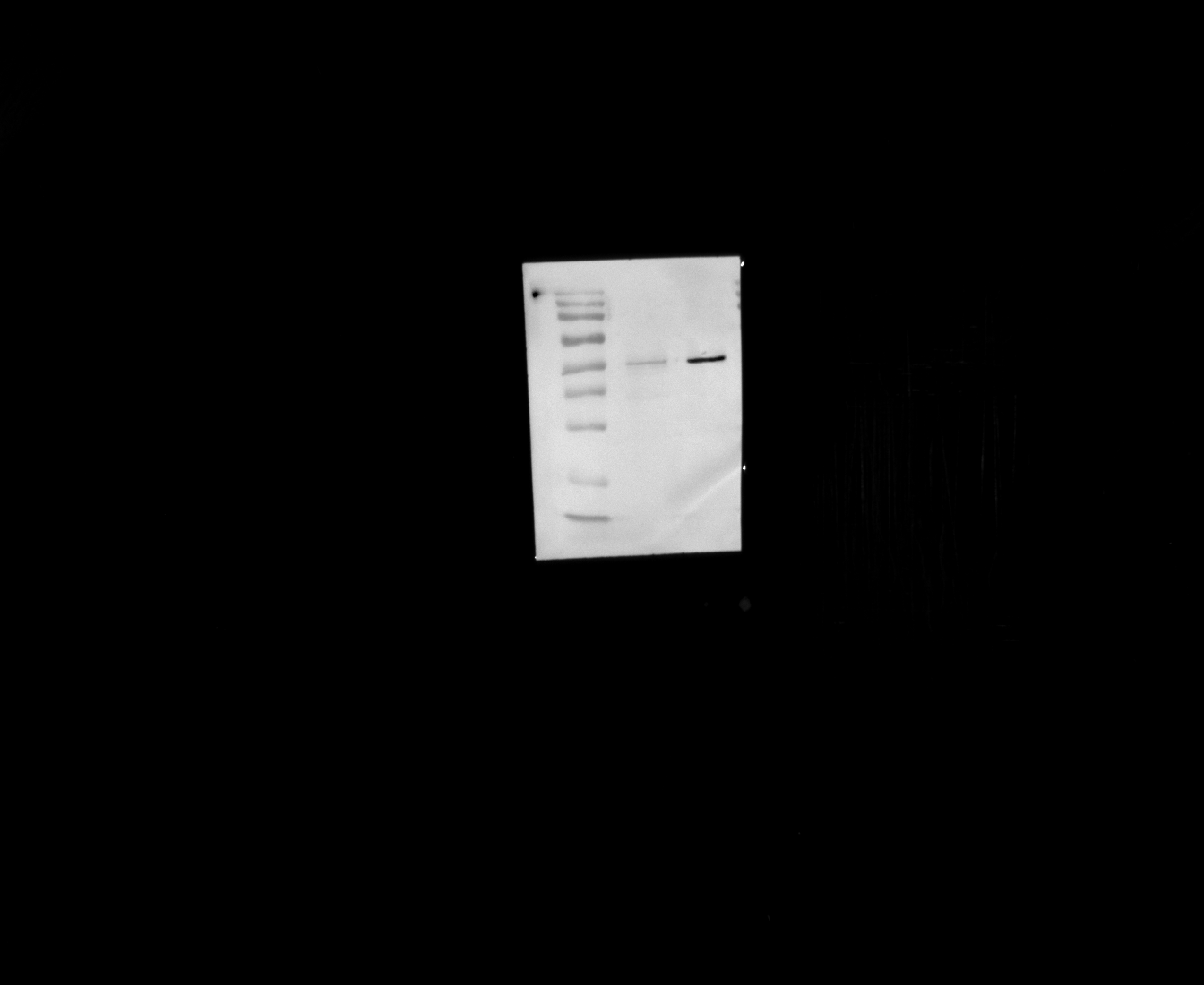

Supplement: Supplementary file 2 — Supplementary Material 2. [file 12935_2025_3665_MOESM2_ESM.zip › Supplementary Material 2/Figure 2/Figure 2E/2/SLC7A11.tif]

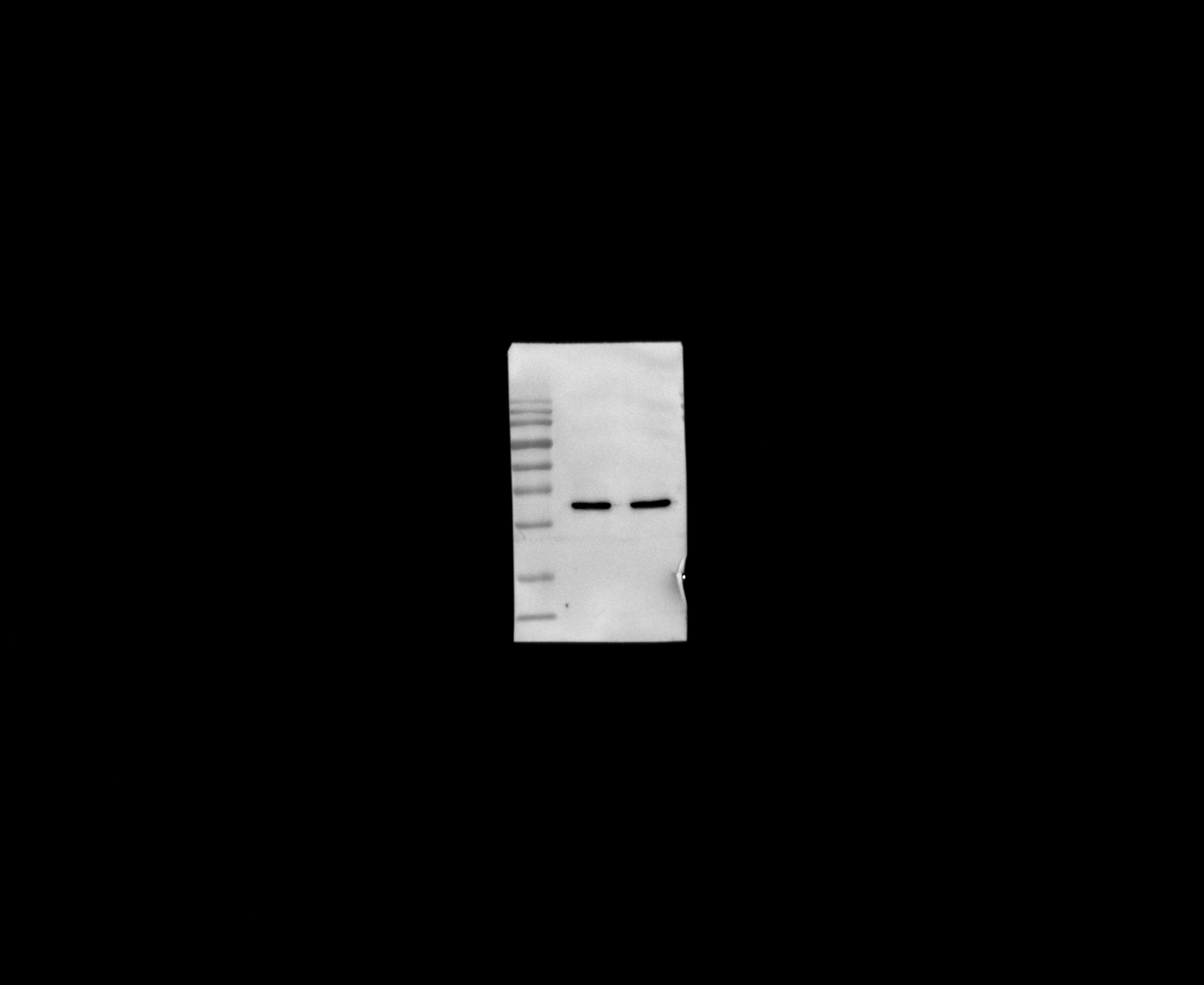

Supplement: Supplementary file 2 — Supplementary Material 2. [file 12935_2025_3665_MOESM2_ESM.zip › Supplementary Material 2/Figure 2/Figure 2E/3/GAPDH.tif]

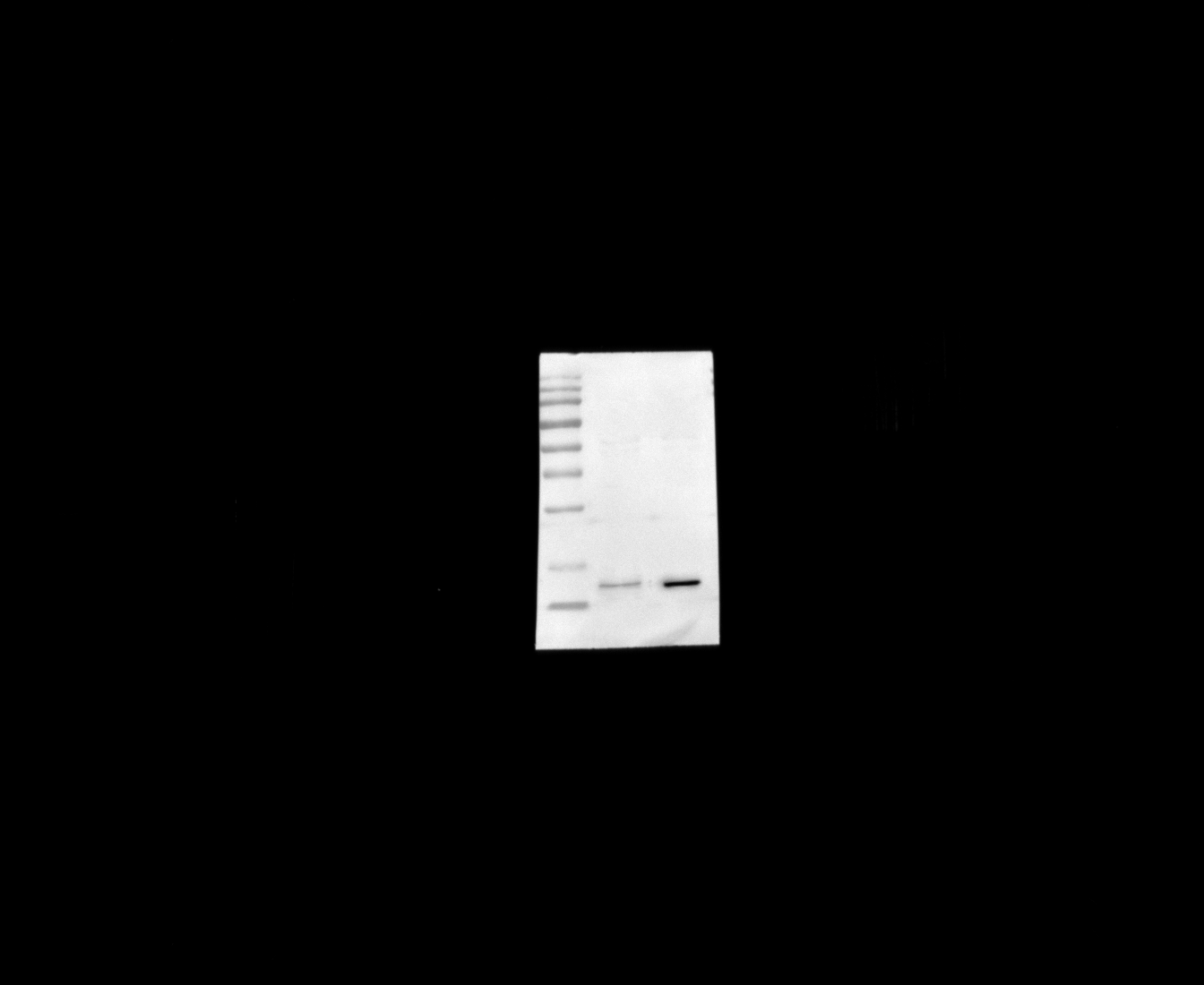

Supplement: Supplementary file 2 — Supplementary Material 2. [file 12935_2025_3665_MOESM2_ESM.zip › Supplementary Material 2/Figure 2/Figure 2E/3/GPX4.tif]

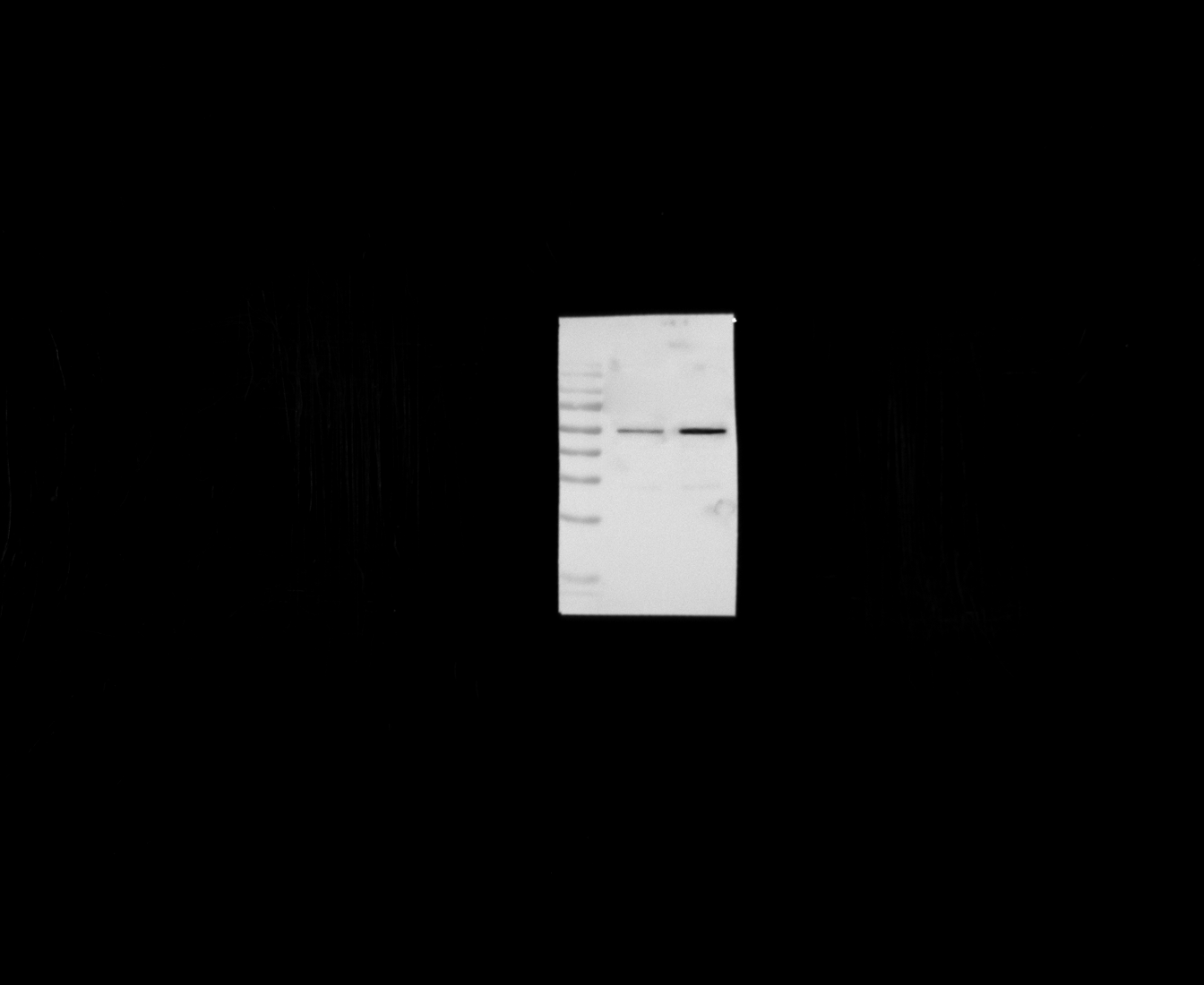

Supplement: Supplementary file 2 — Supplementary Material 2. [file 12935_2025_3665_MOESM2_ESM.zip › Supplementary Material 2/Figure 2/Figure 2E/3/SLC7A11.tif]

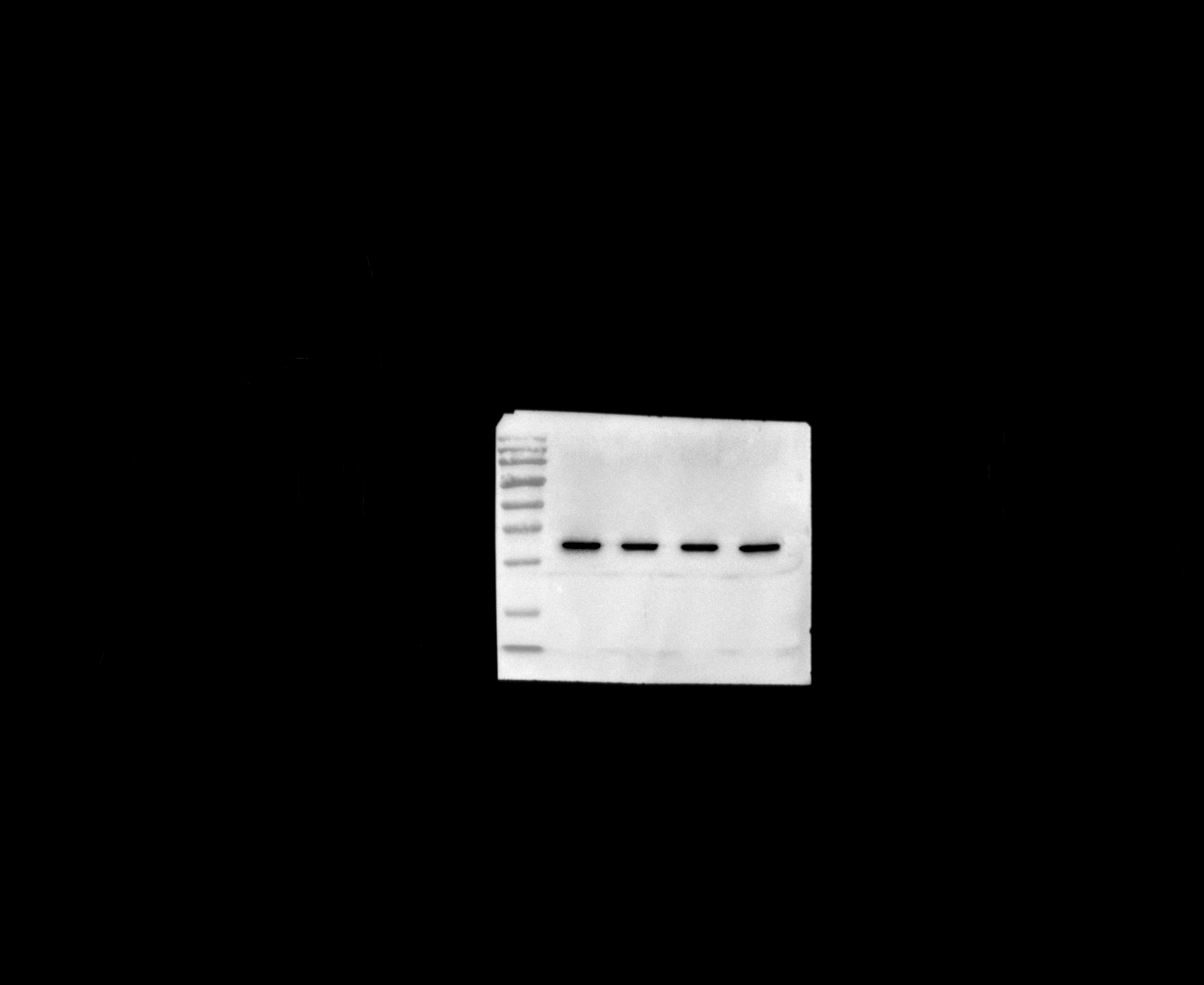

Supplement: Supplementary file 2 — Supplementary Material 2. [file 12935_2025_3665_MOESM2_ESM.zip › Supplementary Material 2/Figure 2/Figure 2E/4/GAPDH.tif]

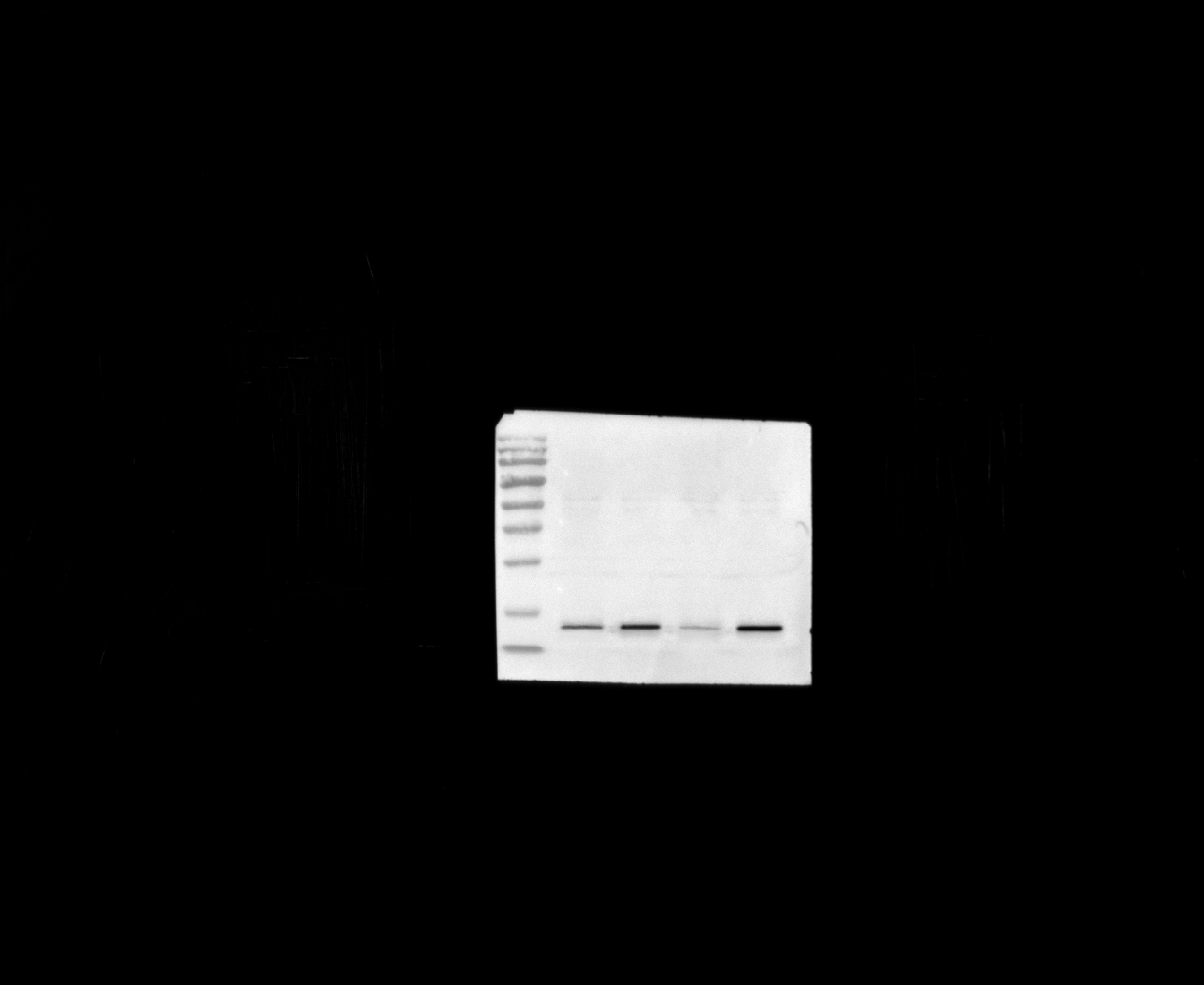

Supplement: Supplementary file 2 — Supplementary Material 2. [file 12935_2025_3665_MOESM2_ESM.zip › Supplementary Material 2/Figure 2/Figure 2E/4/GPX4.tif]

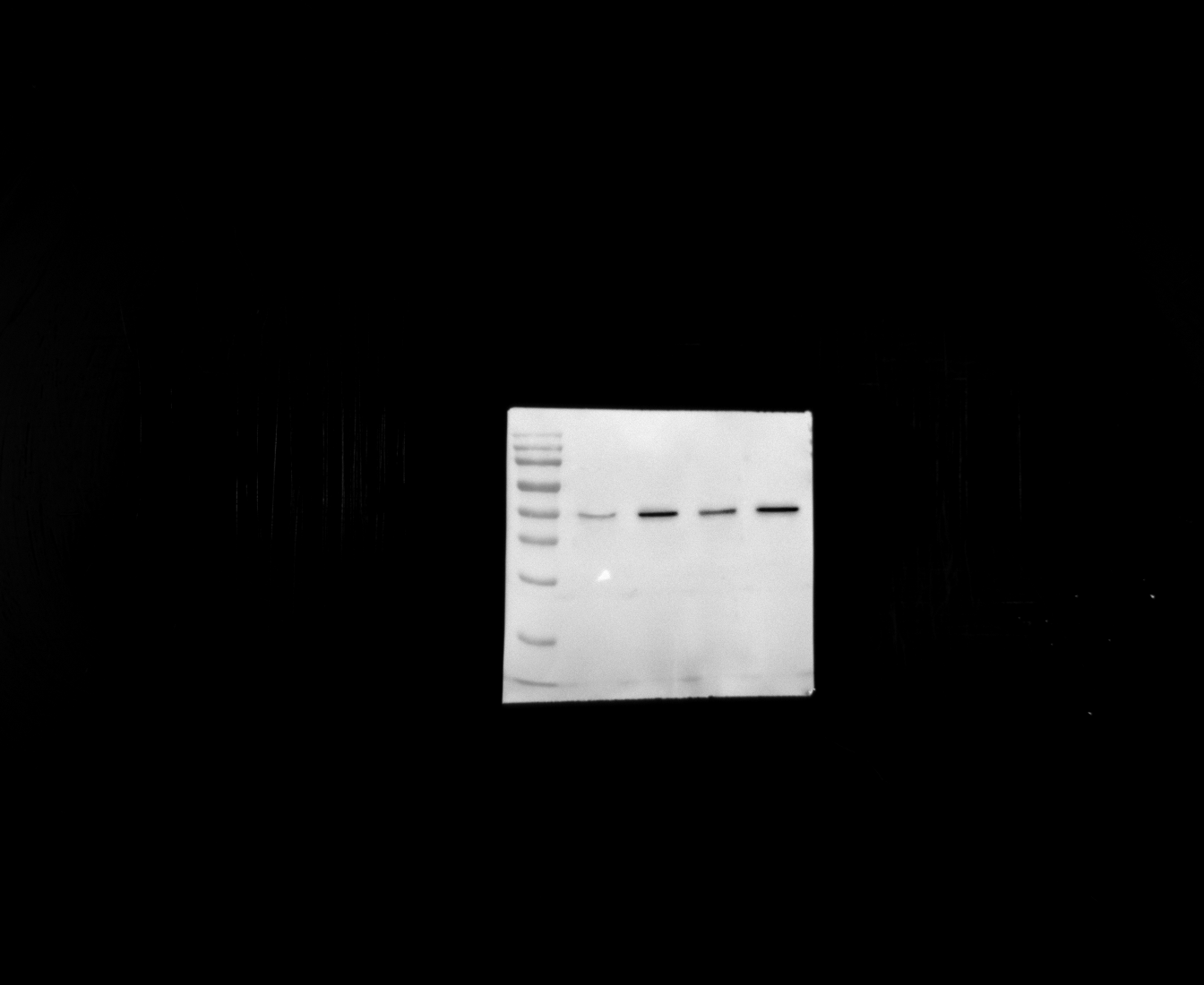

Supplement: Supplementary file 2 — Supplementary Material 2. [file 12935_2025_3665_MOESM2_ESM.zip › Supplementary Material 2/Figure 2/Figure 2E/4/SLC7A11.tif]

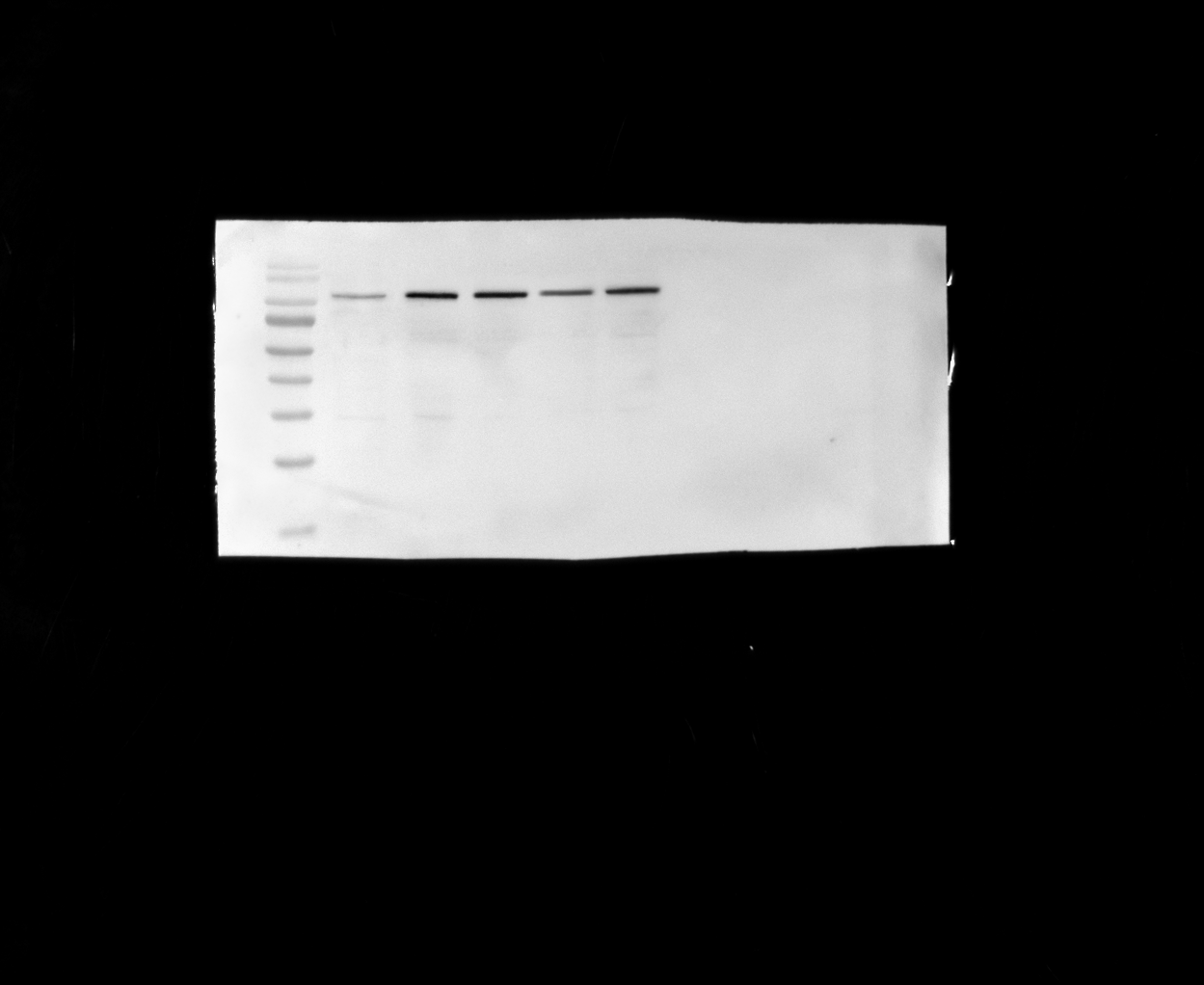

Supplement: Supplementary file 2 — Supplementary Material 2. [file 12935_2025_3665_MOESM2_ESM.zip › Supplementary Material 2/Figure 3/Figure 3I/E-cadherin.tif]

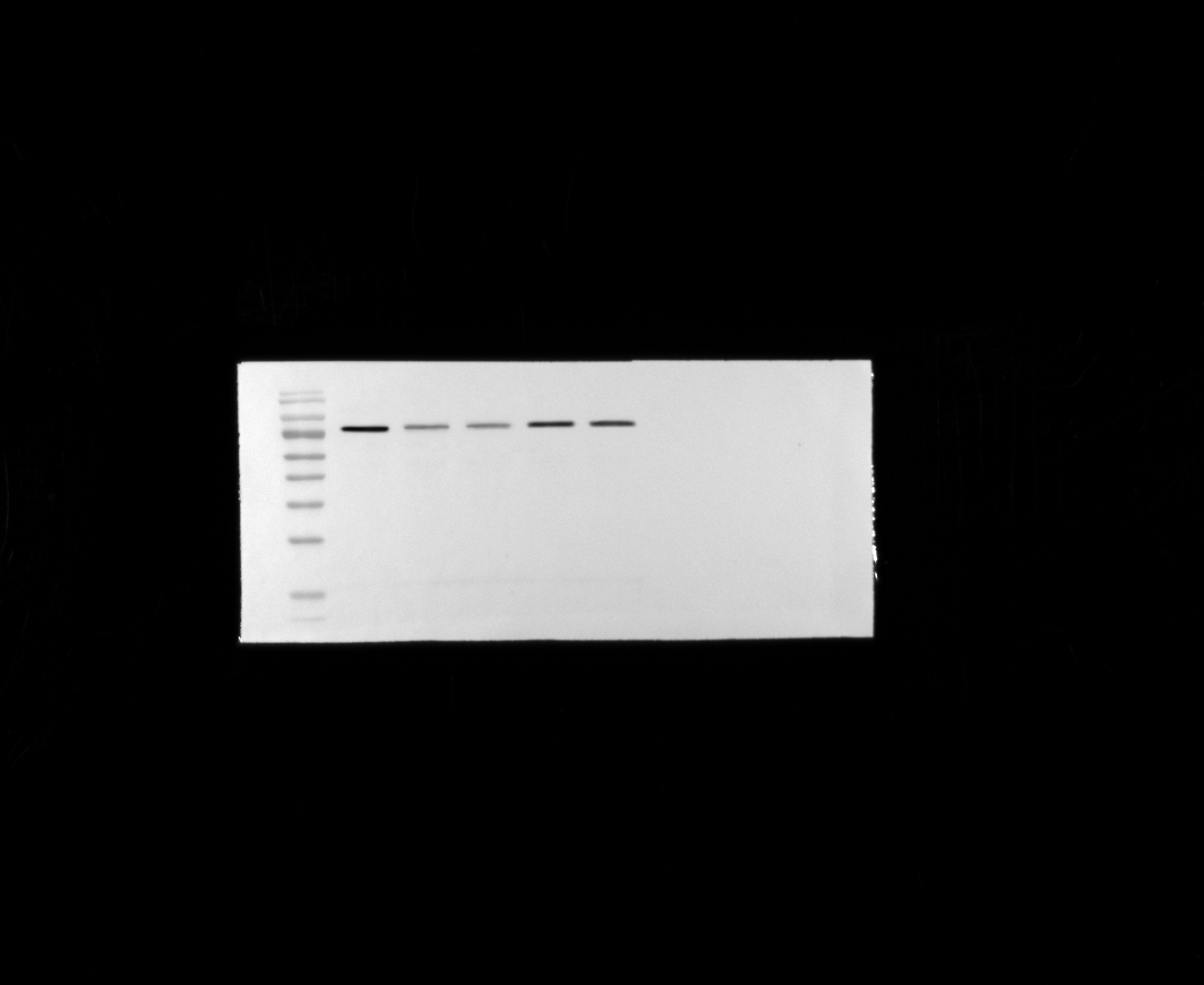

Supplement: Supplementary file 2 — Supplementary Material 2. [file 12935_2025_3665_MOESM2_ESM.zip › Supplementary Material 2/Figure 3/Figure 3I/MMP-2.tif]

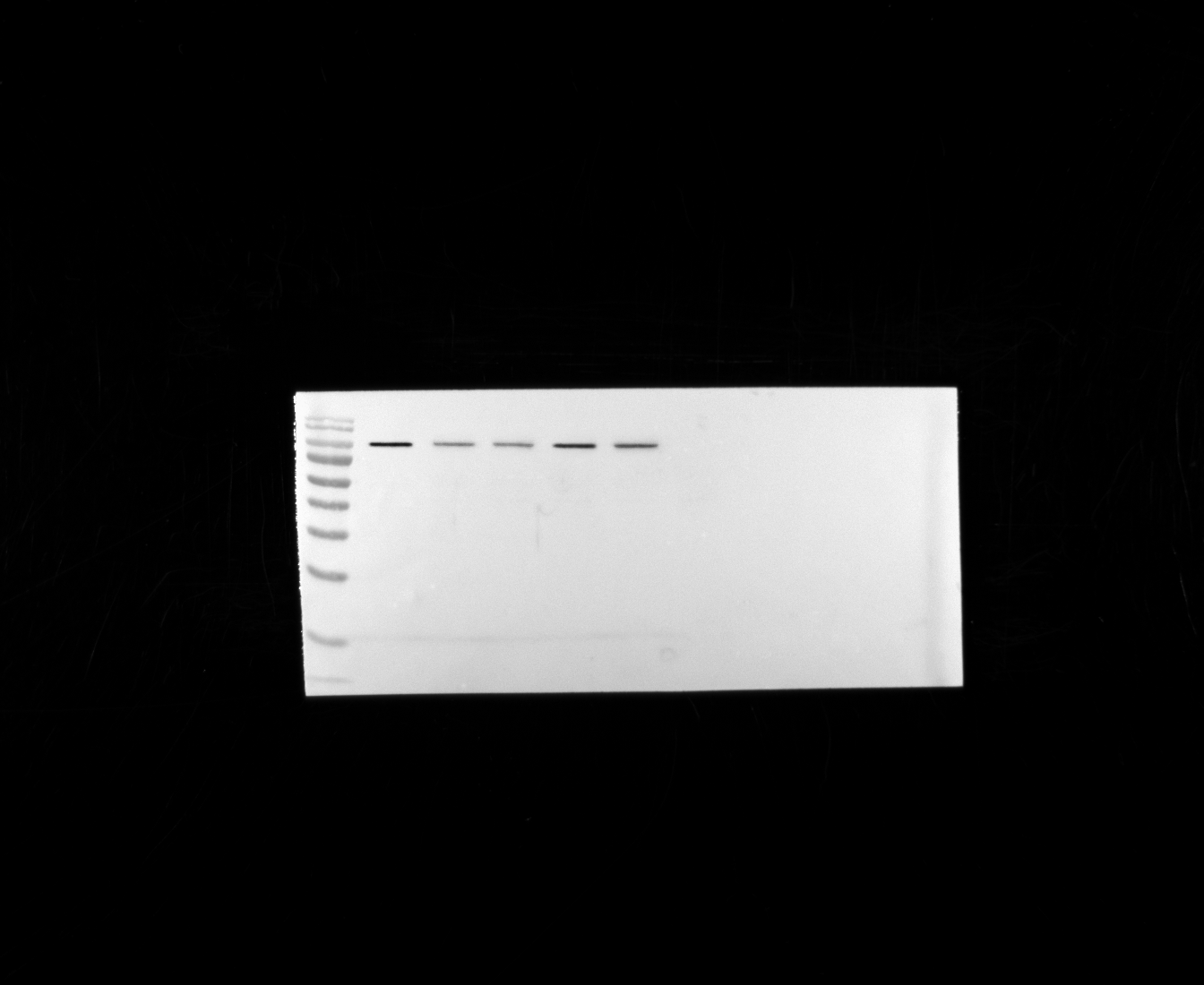

Supplement: Supplementary file 2 — Supplementary Material 2. [file 12935_2025_3665_MOESM2_ESM.zip › Supplementary Material 2/Figure 3/Figure 3I/MMP-9.tif]

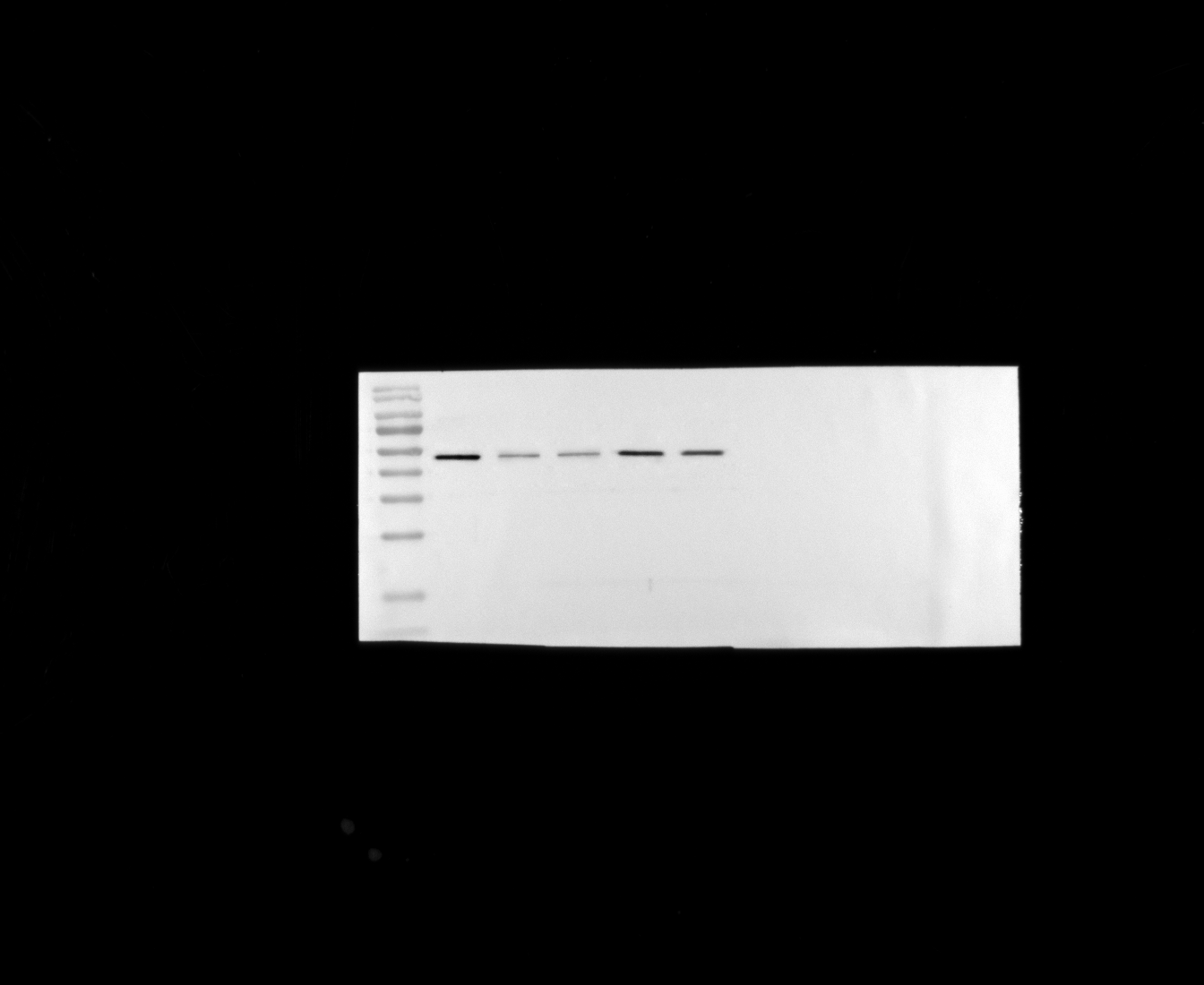

Supplement: Supplementary file 2 — Supplementary Material 2. [file 12935_2025_3665_MOESM2_ESM.zip › Supplementary Material 2/Figure 3/Figure 3I/Vimentin.tif]

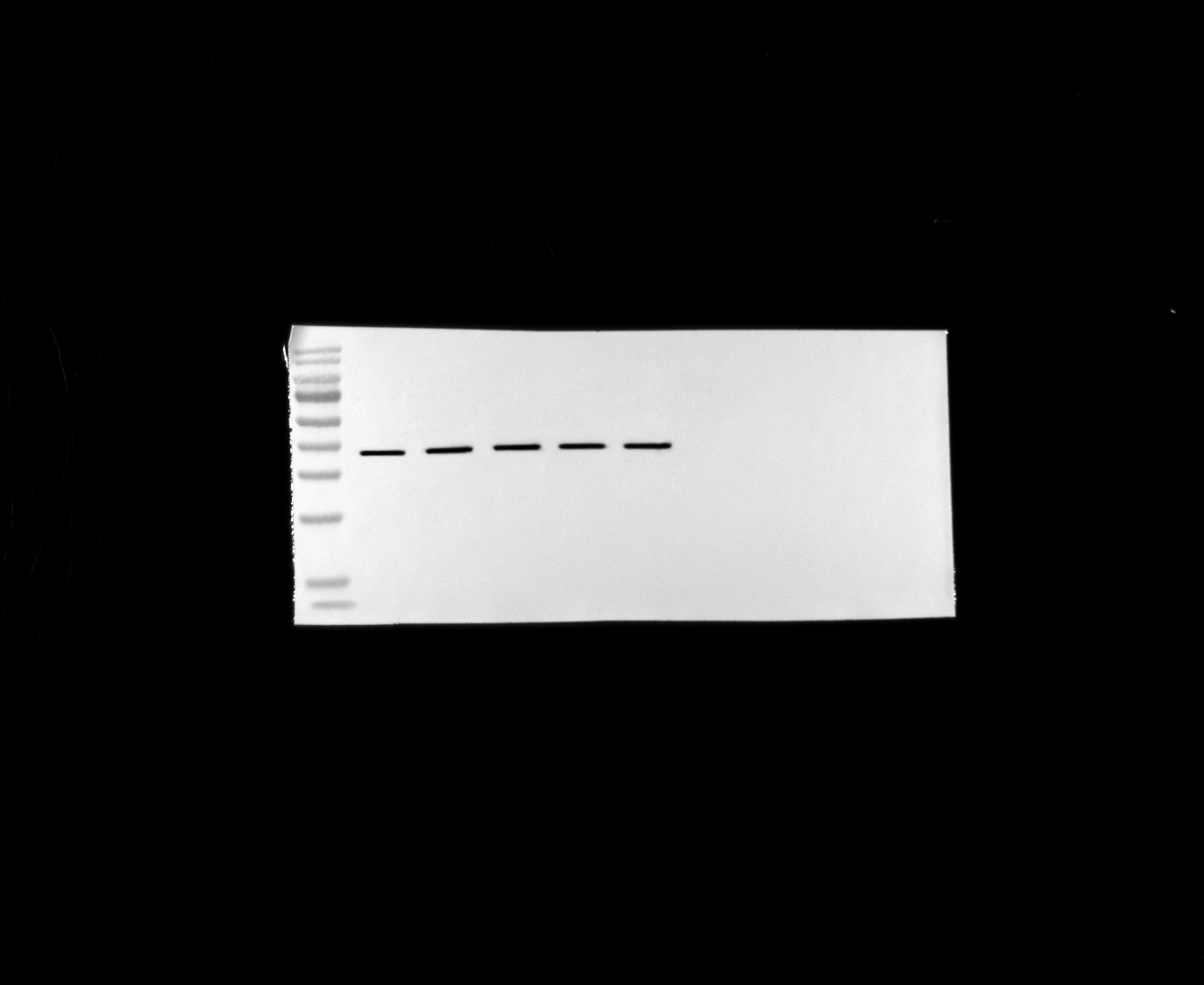

Supplement: Supplementary file 2 — Supplementary Material 2. [file 12935_2025_3665_MOESM2_ESM.zip › Supplementary Material 2/Figure 3/Figure 3I/β-actin.tif]

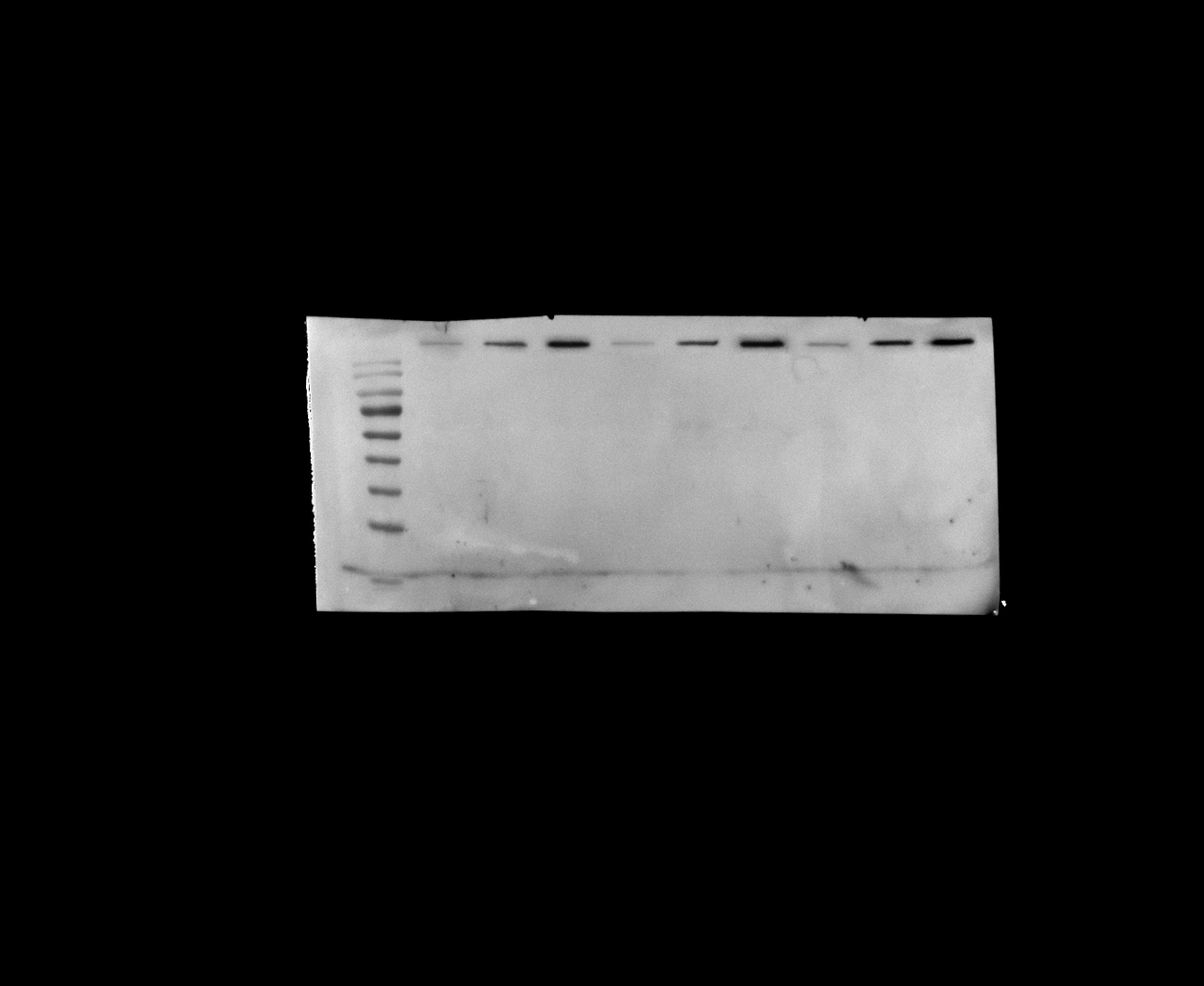

Supplement: Supplementary file 2 — Supplementary Material 2. [file 12935_2025_3665_MOESM2_ESM.zip › Supplementary Material 2/Figure 5/Figure 5A/EGFR.tif]

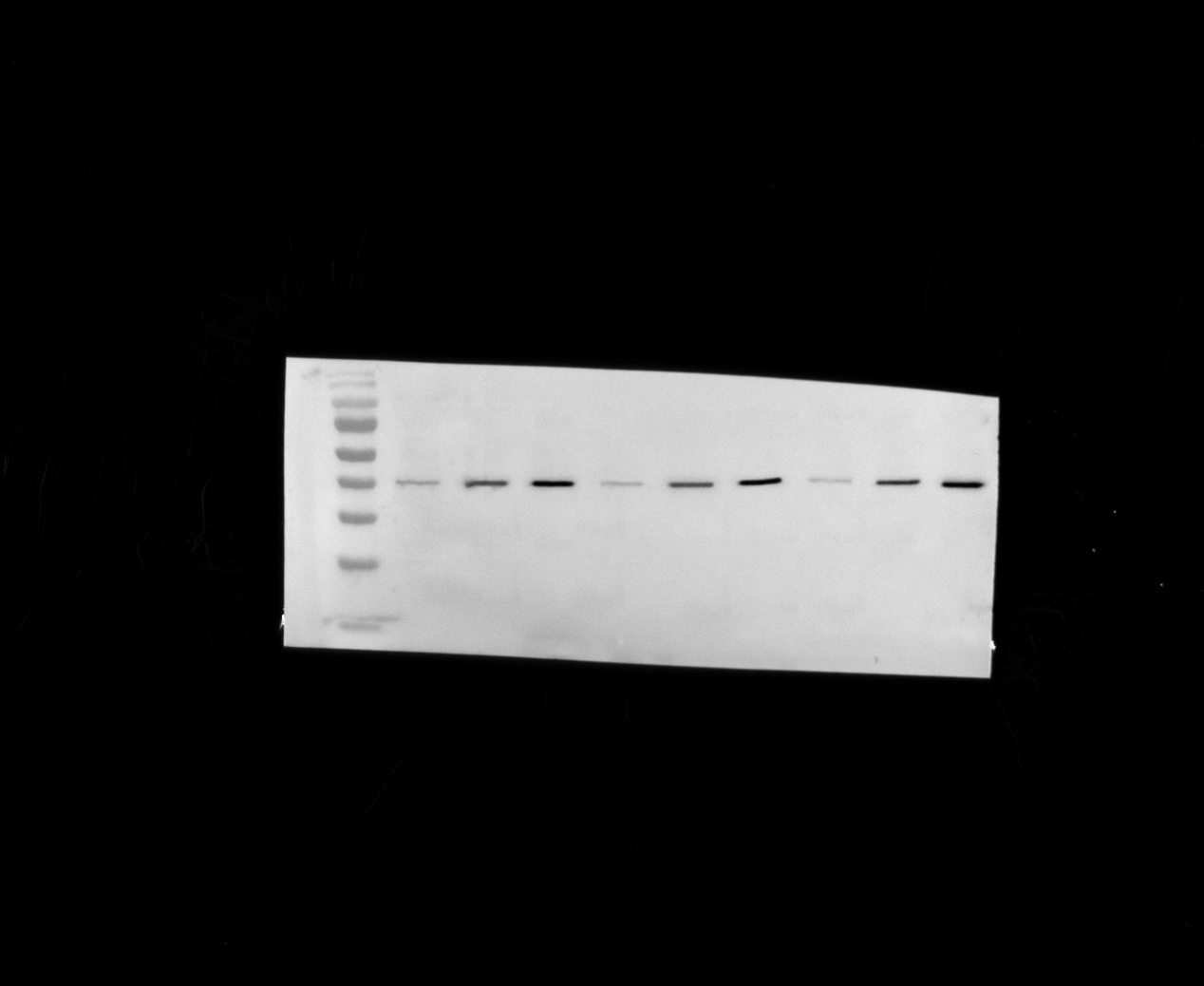

Supplement: Supplementary file 2 — Supplementary Material 2. [file 12935_2025_3665_MOESM2_ESM.zip › Supplementary Material 2/Figure 5/Figure 5A/ERK2.tif]

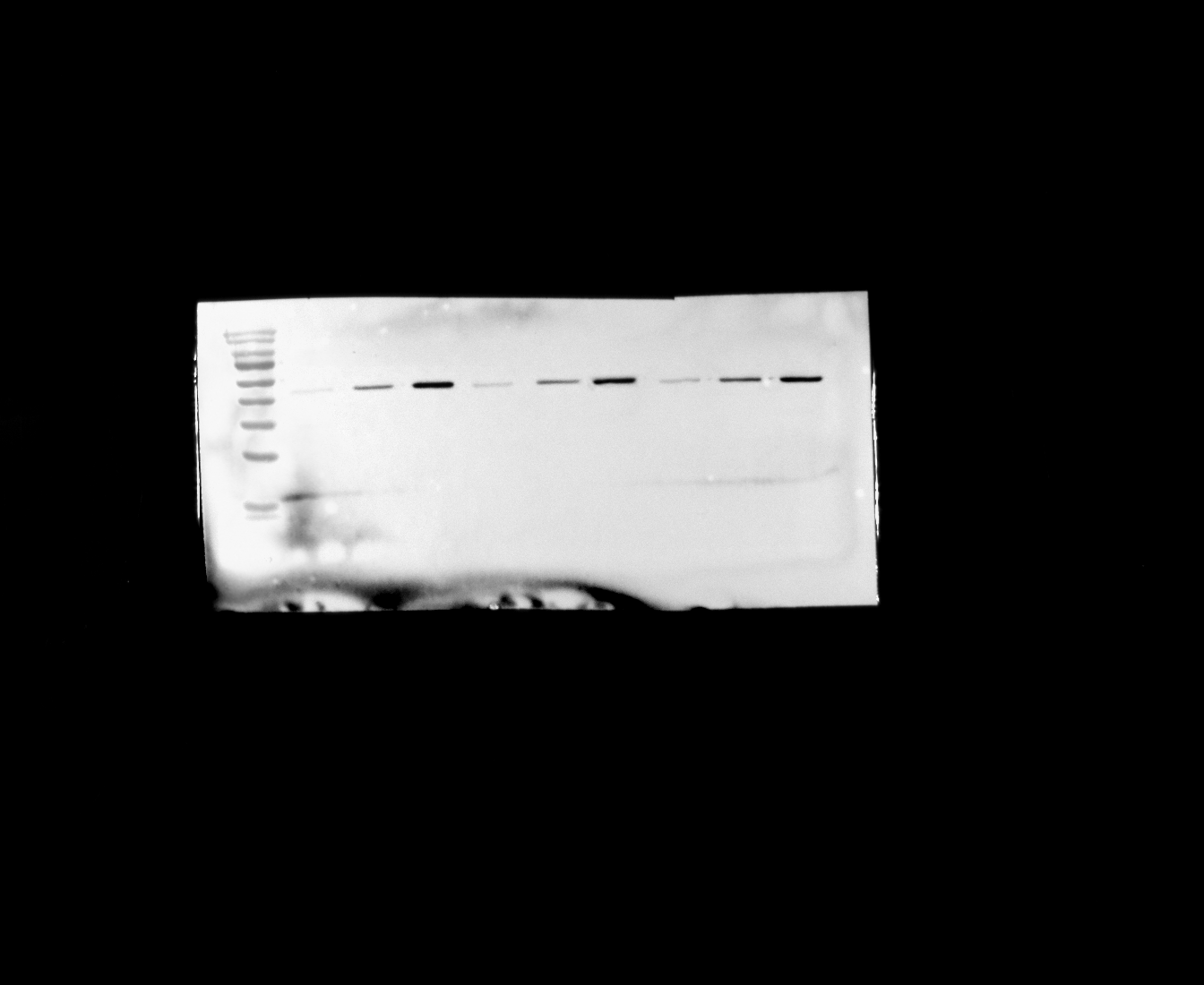

Supplement: Supplementary file 2 — Supplementary Material 2. [file 12935_2025_3665_MOESM2_ESM.zip › Supplementary Material 2/Figure 5/Figure 5A/Ets-1.tif]

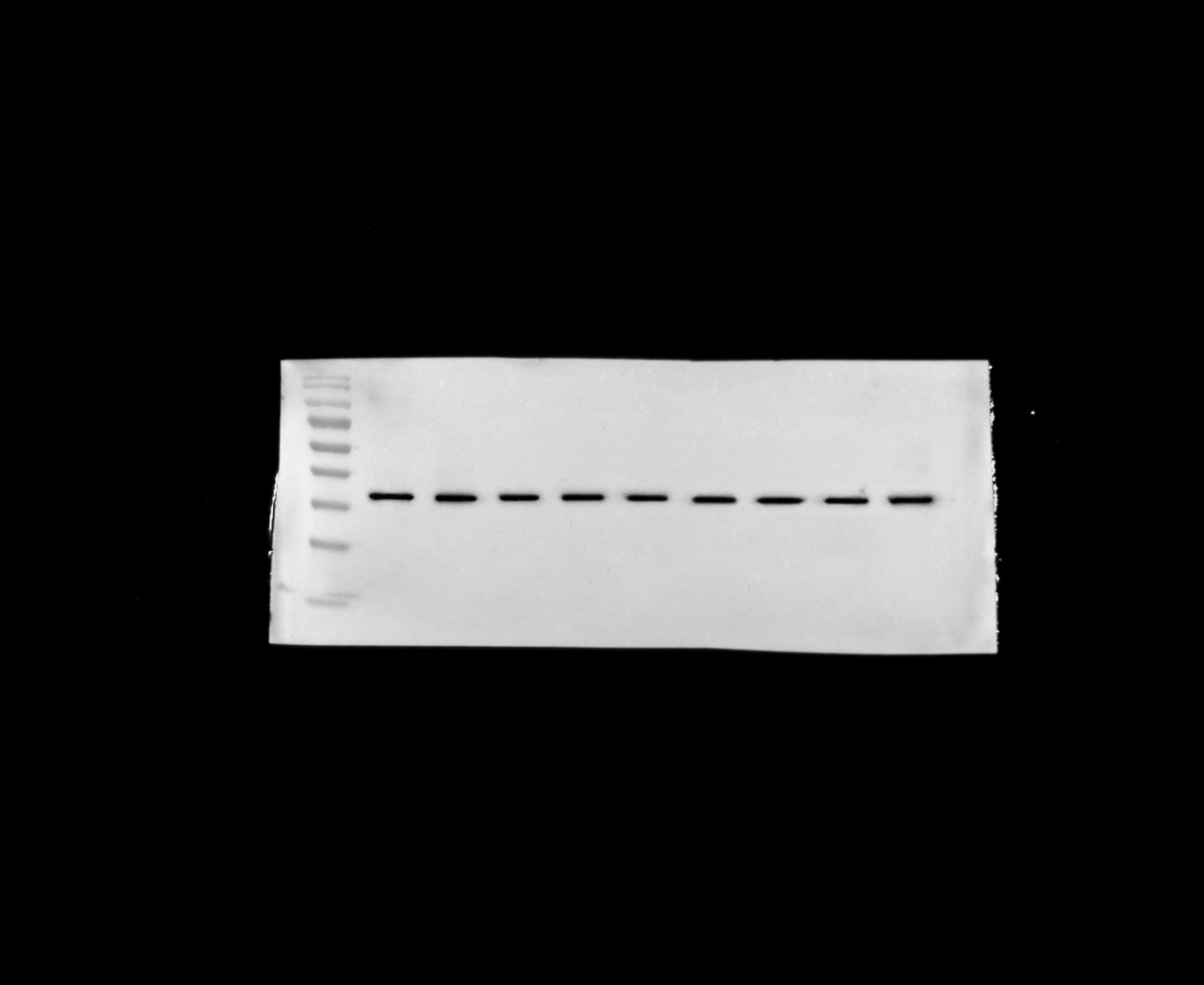

Supplement: Supplementary file 2 — Supplementary Material 2. [file 12935_2025_3665_MOESM2_ESM.zip › Supplementary Material 2/Figure 5/Figure 5A/GAPDH.tif]

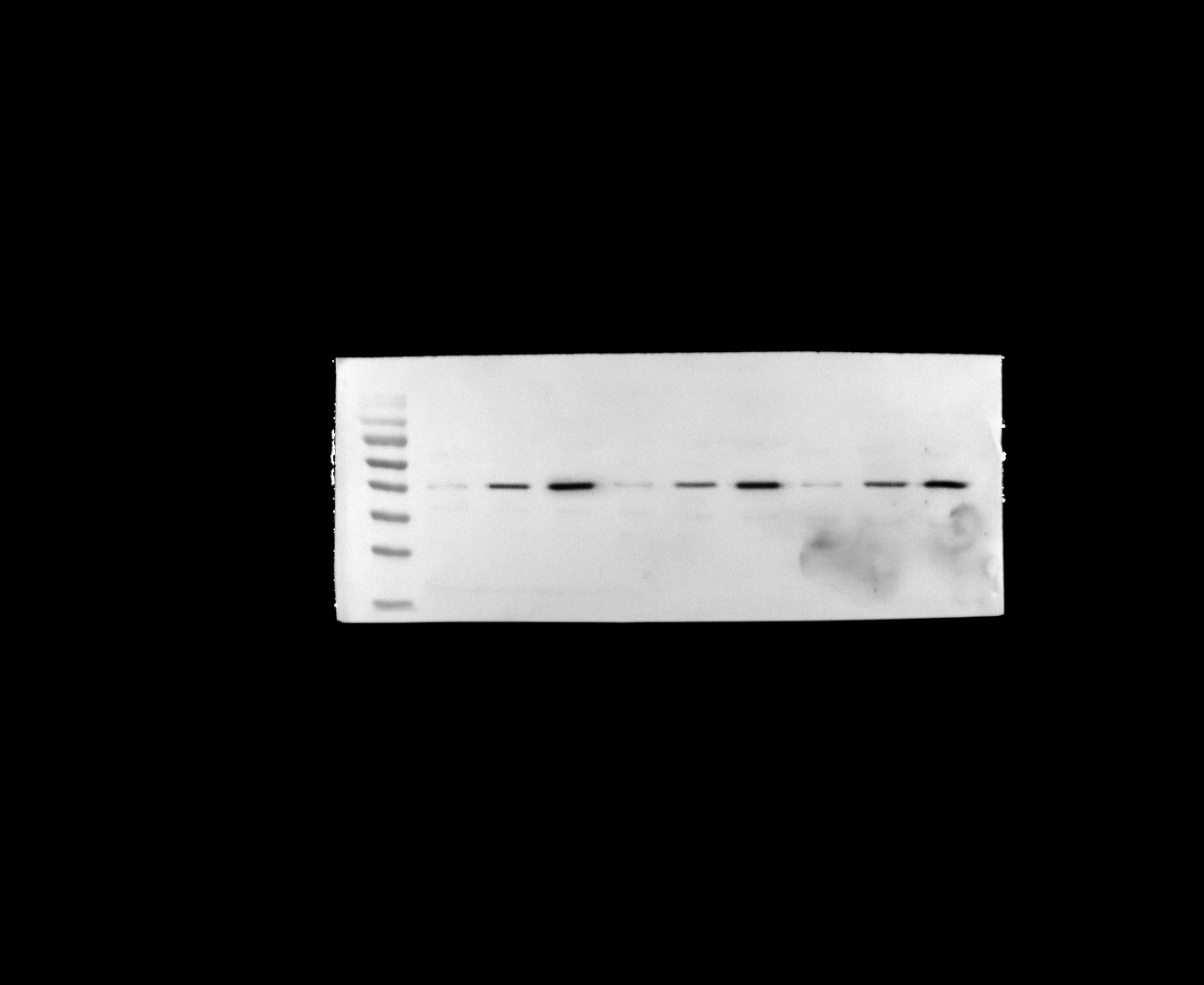

Supplement: Supplementary file 2 — Supplementary Material 2. [file 12935_2025_3665_MOESM2_ESM.zip › Supplementary Material 2/Figure 5/Figure 5A/MEK.tif]

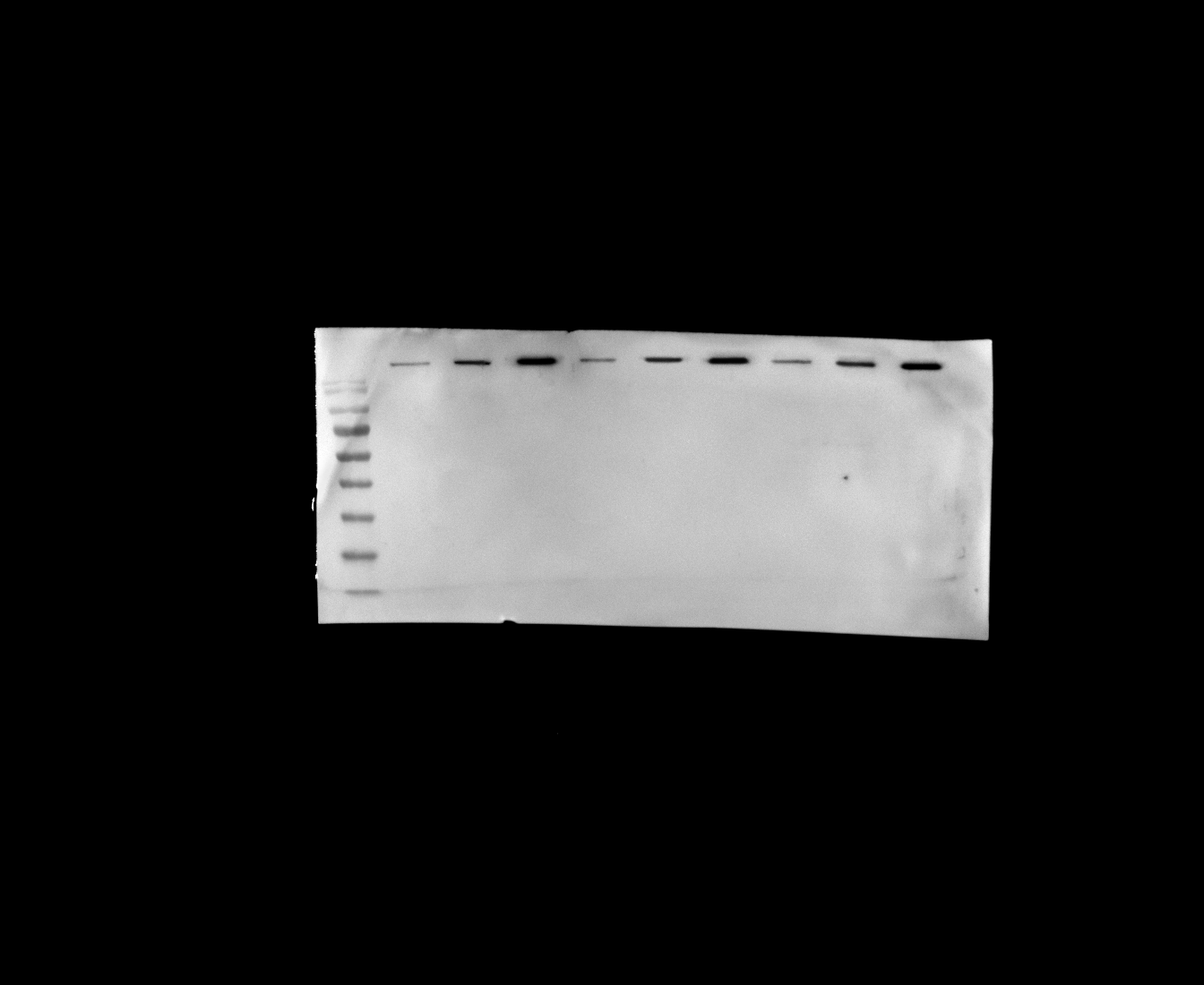

Supplement: Supplementary file 2 — Supplementary Material 2. [file 12935_2025_3665_MOESM2_ESM.zip › Supplementary Material 2/Figure 5/Figure 5A/p-EGFR(Y1069).tif]

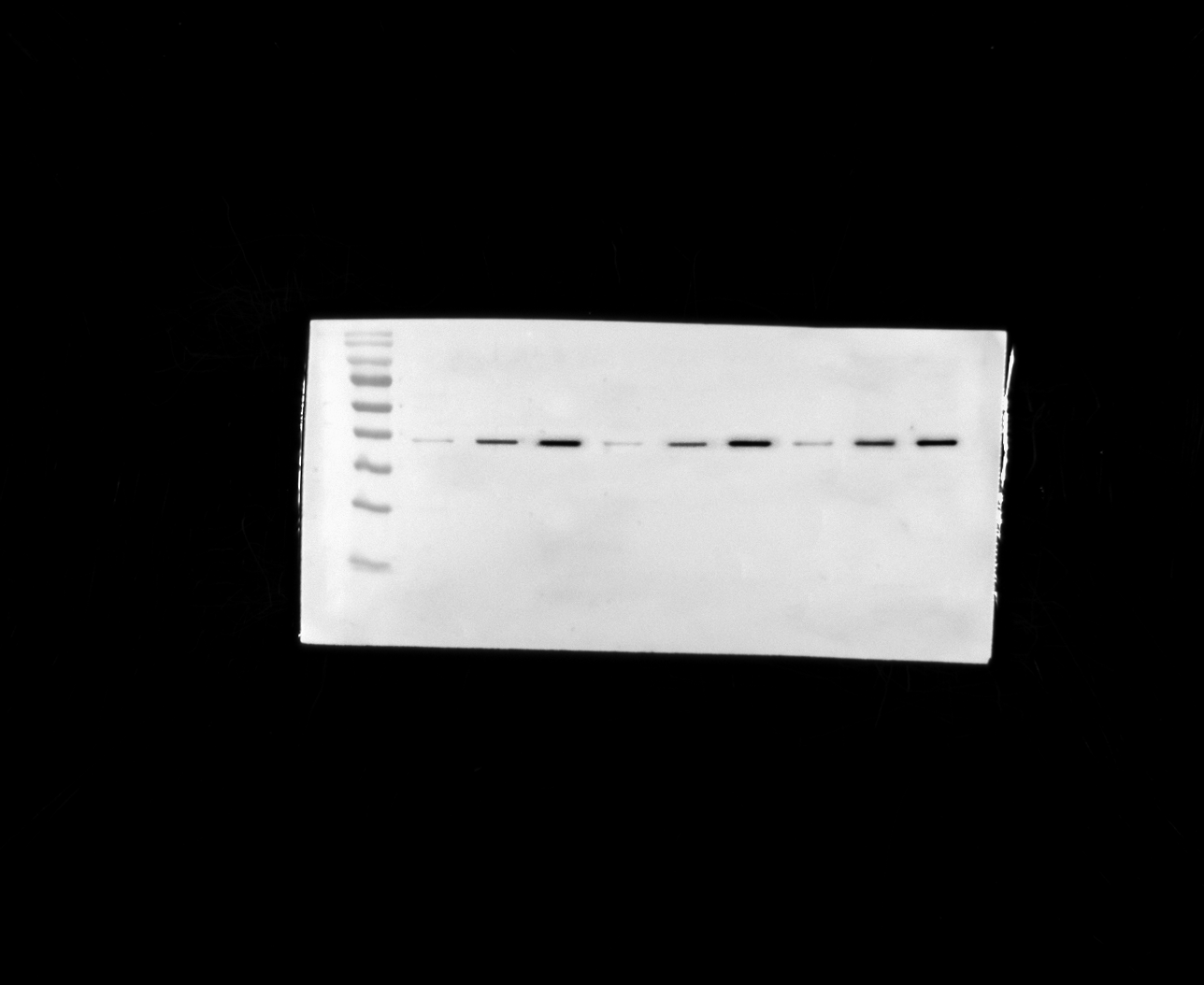

Supplement: Supplementary file 2 — Supplementary Material 2. [file 12935_2025_3665_MOESM2_ESM.zip › Supplementary Material 2/Figure 5/Figure 5A/P-ERK2(T185) .tif]

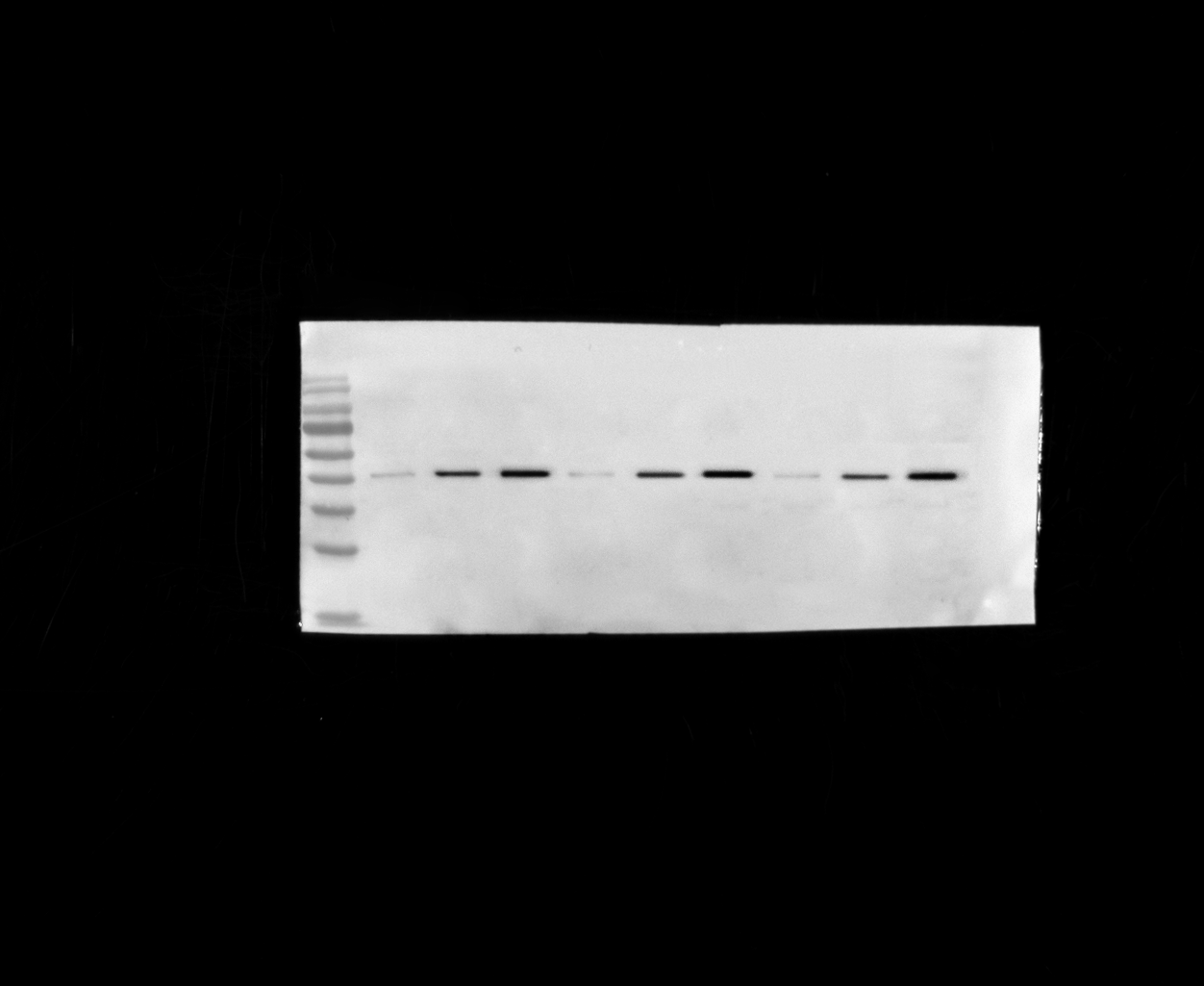

Supplement: Supplementary file 2 — Supplementary Material 2. [file 12935_2025_3665_MOESM2_ESM.zip › Supplementary Material 2/Figure 5/Figure 5A/p-MEK.tif]

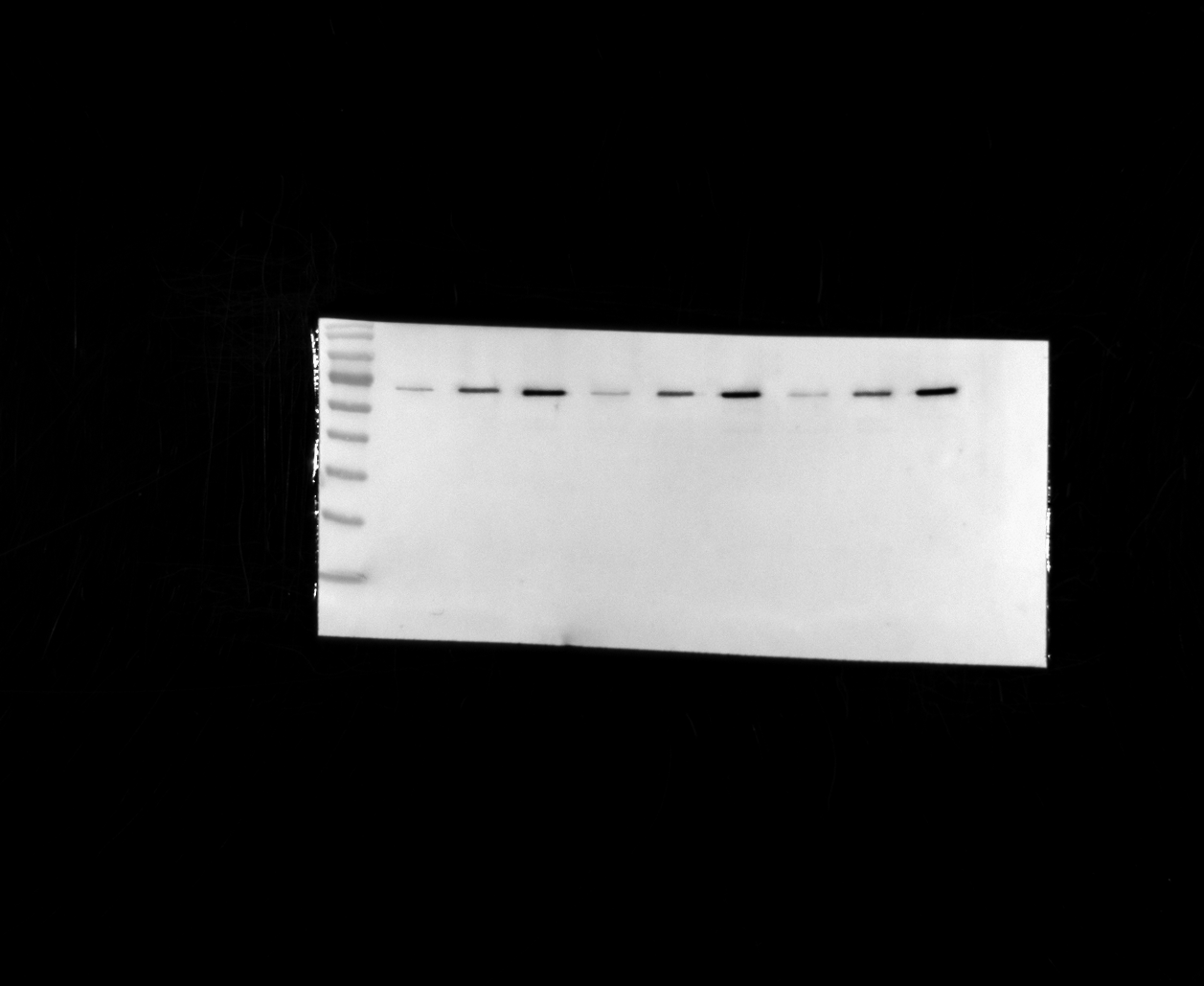

Supplement: Supplementary file 2 — Supplementary Material 2. [file 12935_2025_3665_MOESM2_ESM.zip › Supplementary Material 2/Figure 5/Figure 5A/RAF.tif]

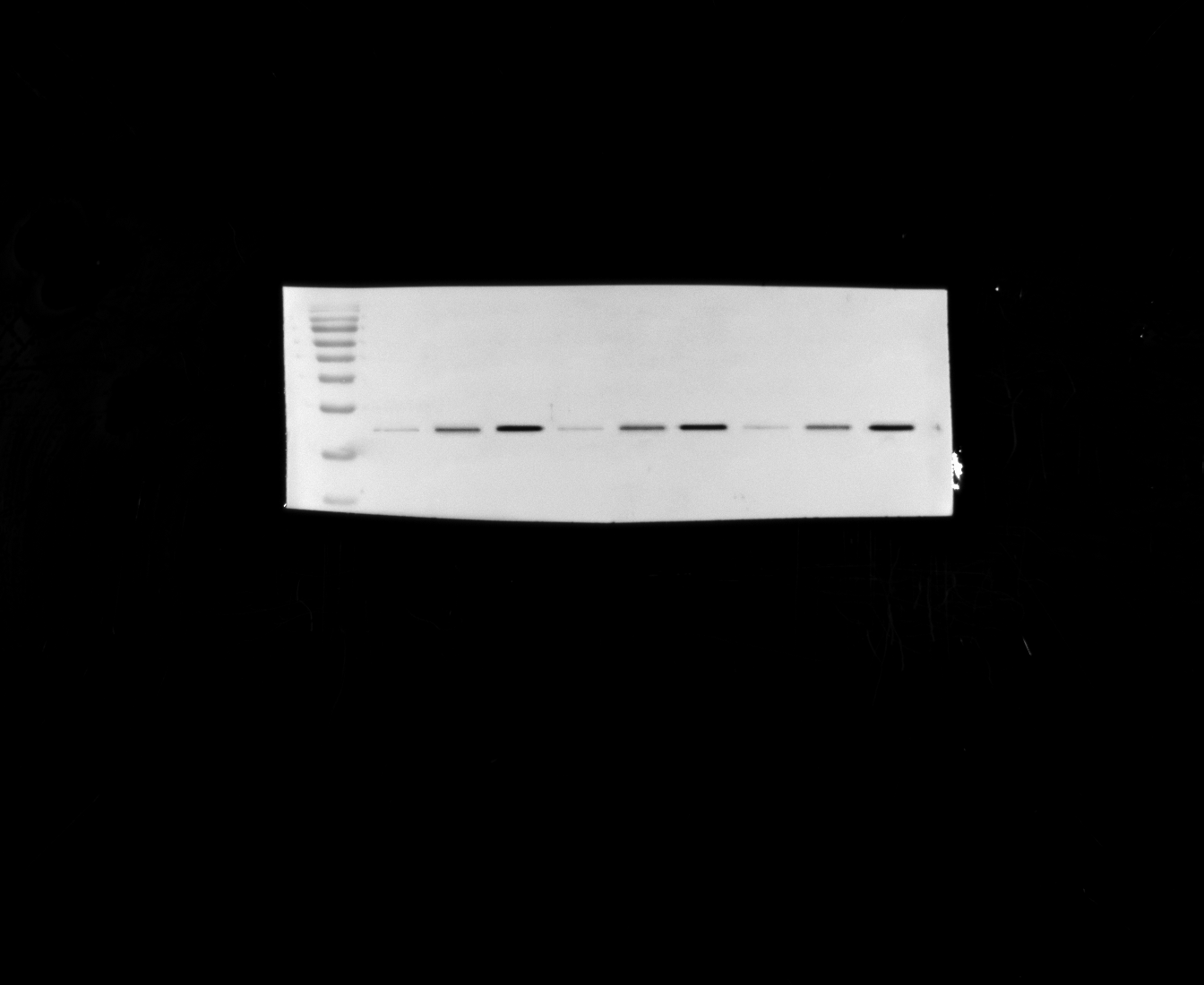

Supplement: Supplementary file 2 — Supplementary Material 2. [file 12935_2025_3665_MOESM2_ESM.zip › Supplementary Material 2/Figure 5/Figure 5A/RAS.tif]

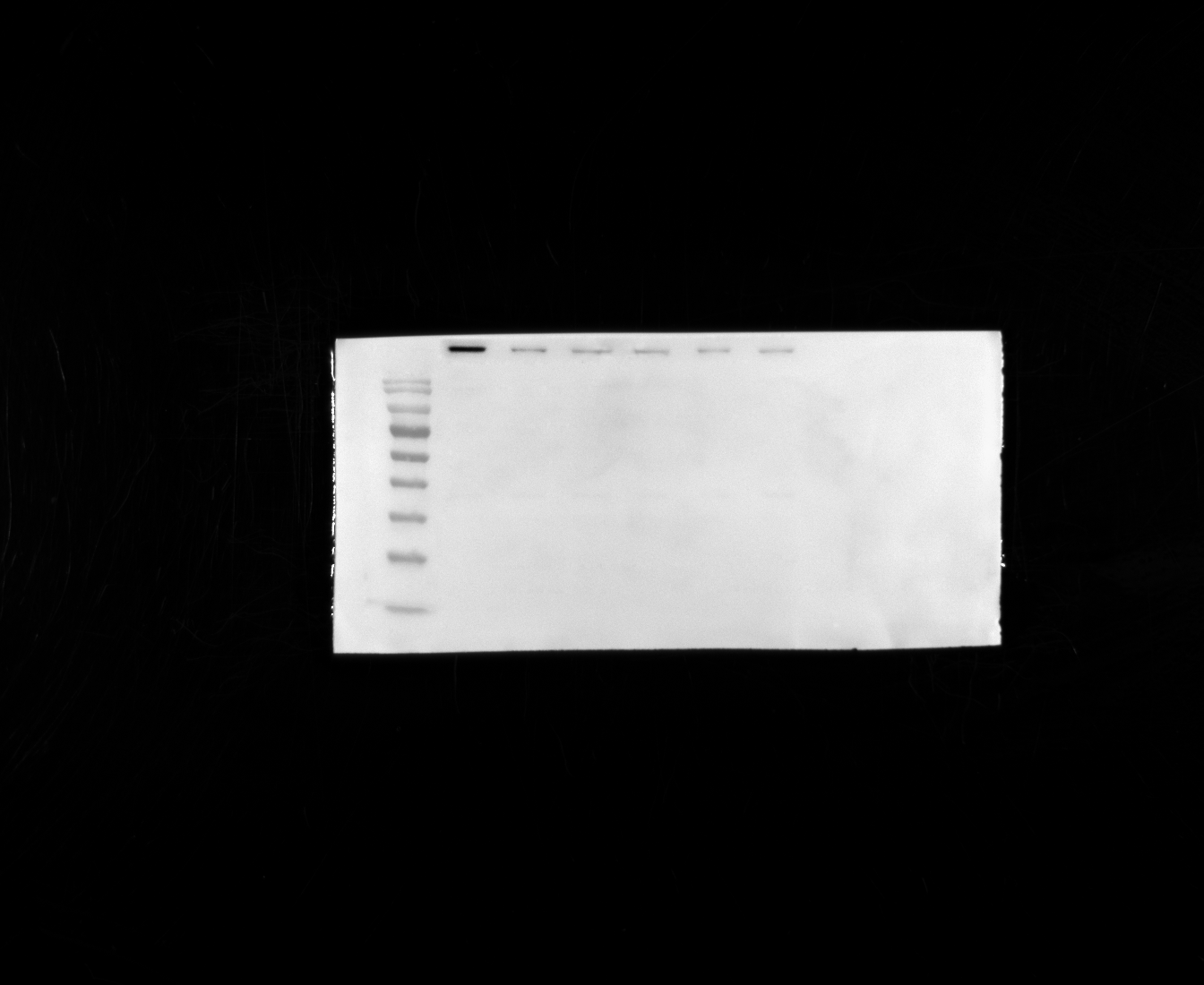

Supplement: Supplementary file 2 — Supplementary Material 2. [file 12935_2025_3665_MOESM2_ESM.zip › Supplementary Material 2/Figure 5/Figure 5C/EGFR.tif]

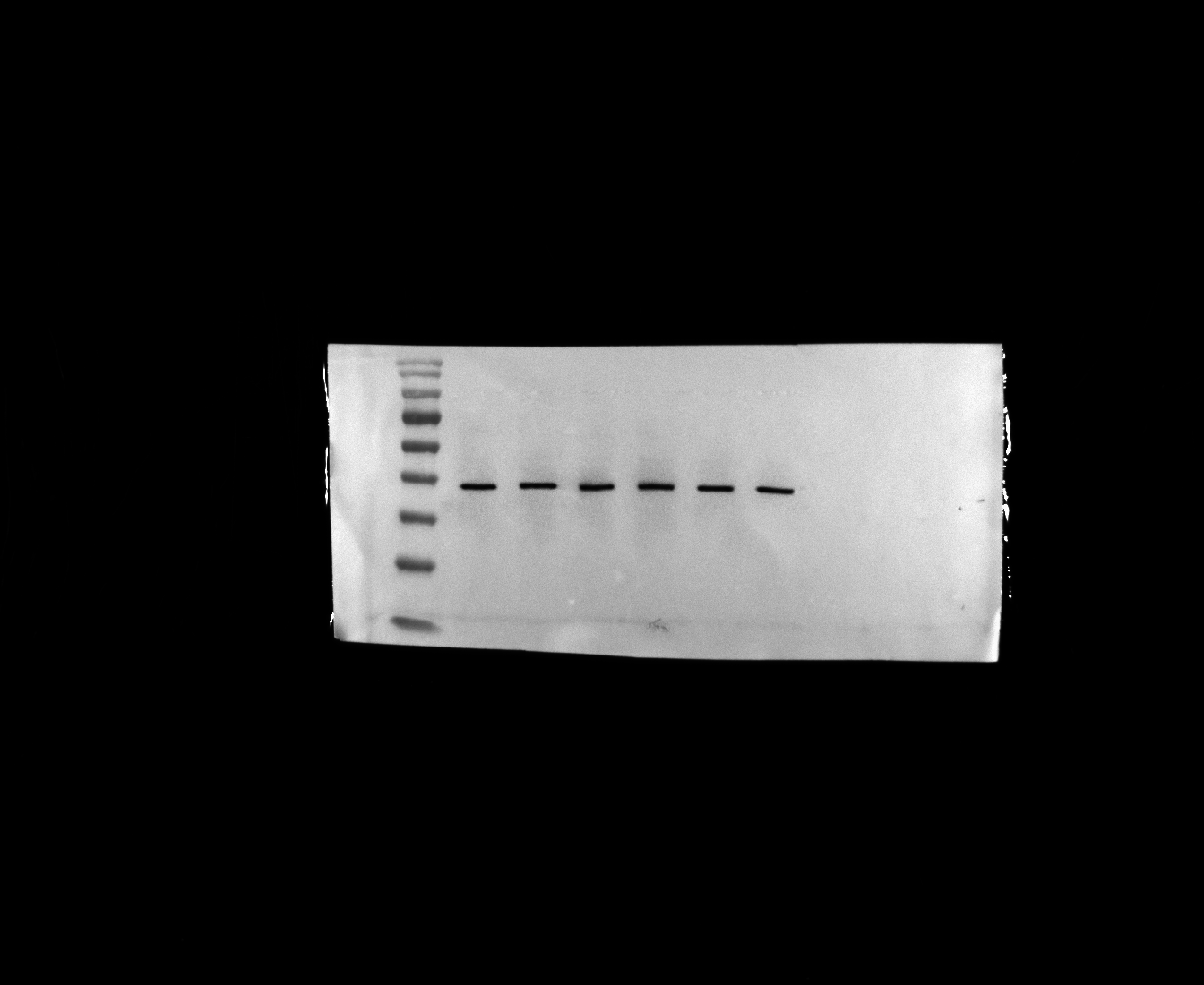

Supplement: Supplementary file 2 — Supplementary Material 2. [file 12935_2025_3665_MOESM2_ESM.zip › Supplementary Material 2/Figure 5/Figure 5C/ERK2.tif]

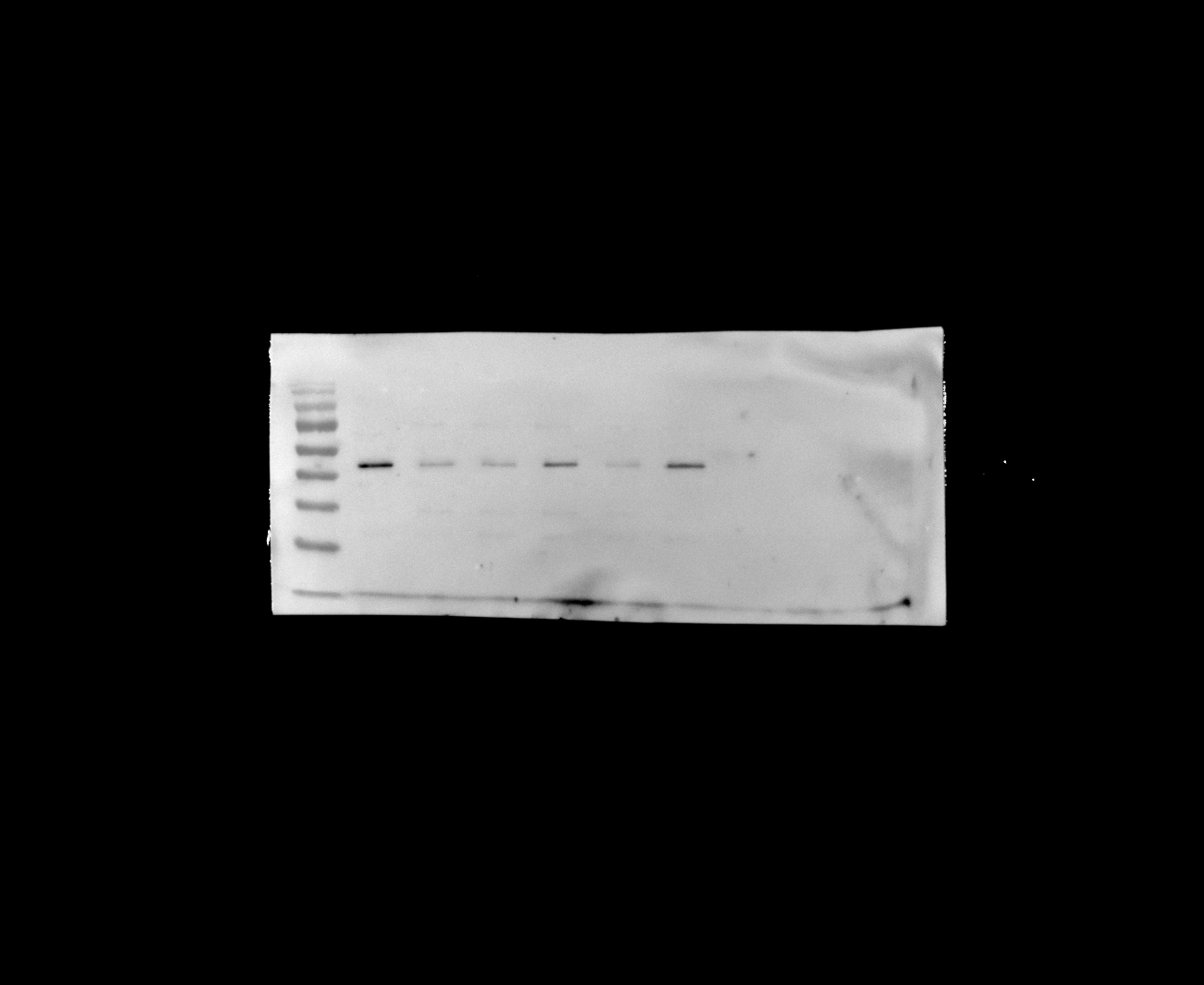

Supplement: Supplementary file 2 — Supplementary Material 2. [file 12935_2025_3665_MOESM2_ESM.zip › Supplementary Material 2/Figure 5/Figure 5C/Ets-1.tif]

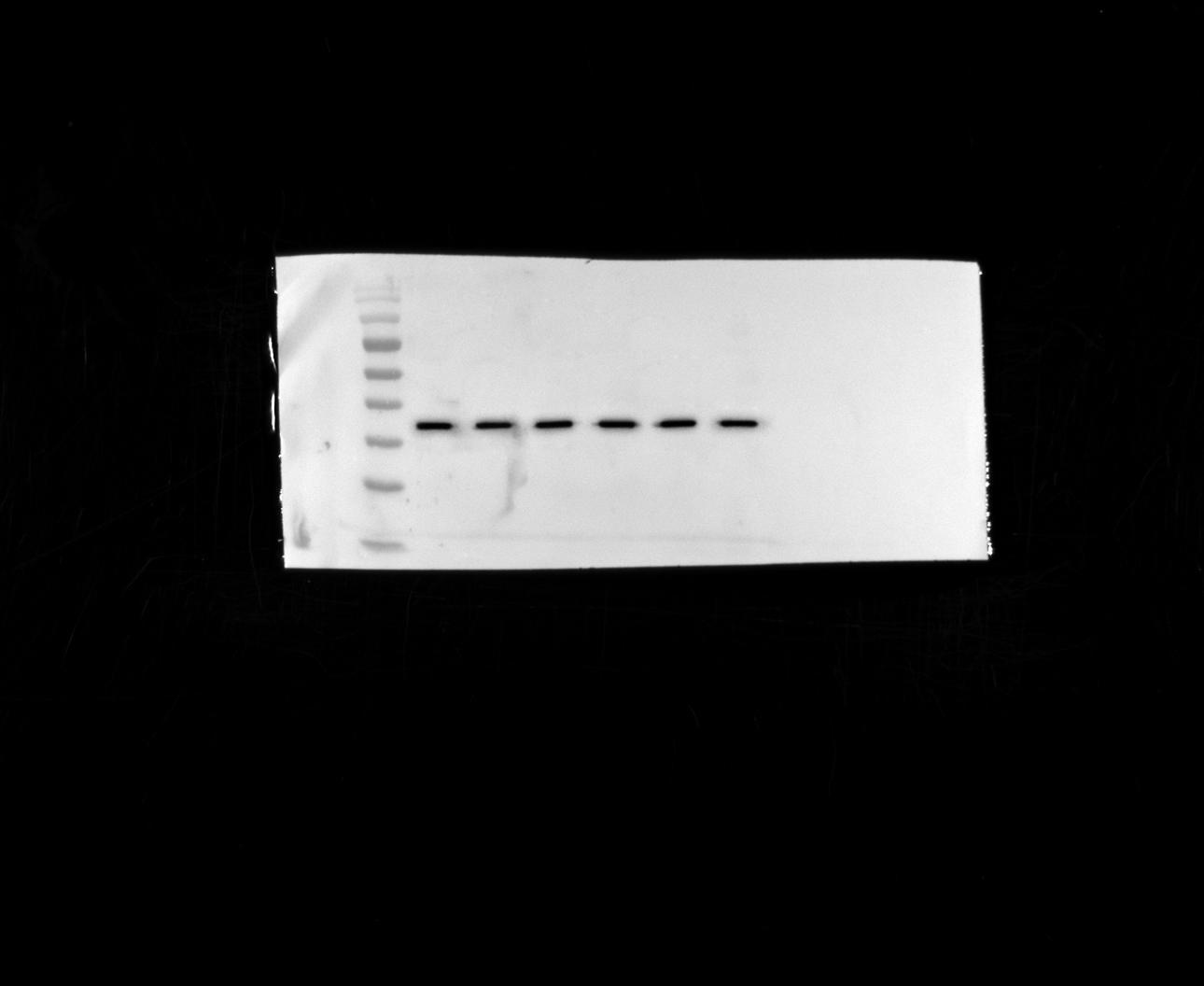

Supplement: Supplementary file 2 — Supplementary Material 2. [file 12935_2025_3665_MOESM2_ESM.zip › Supplementary Material 2/Figure 5/Figure 5C/GAPDH.tif]

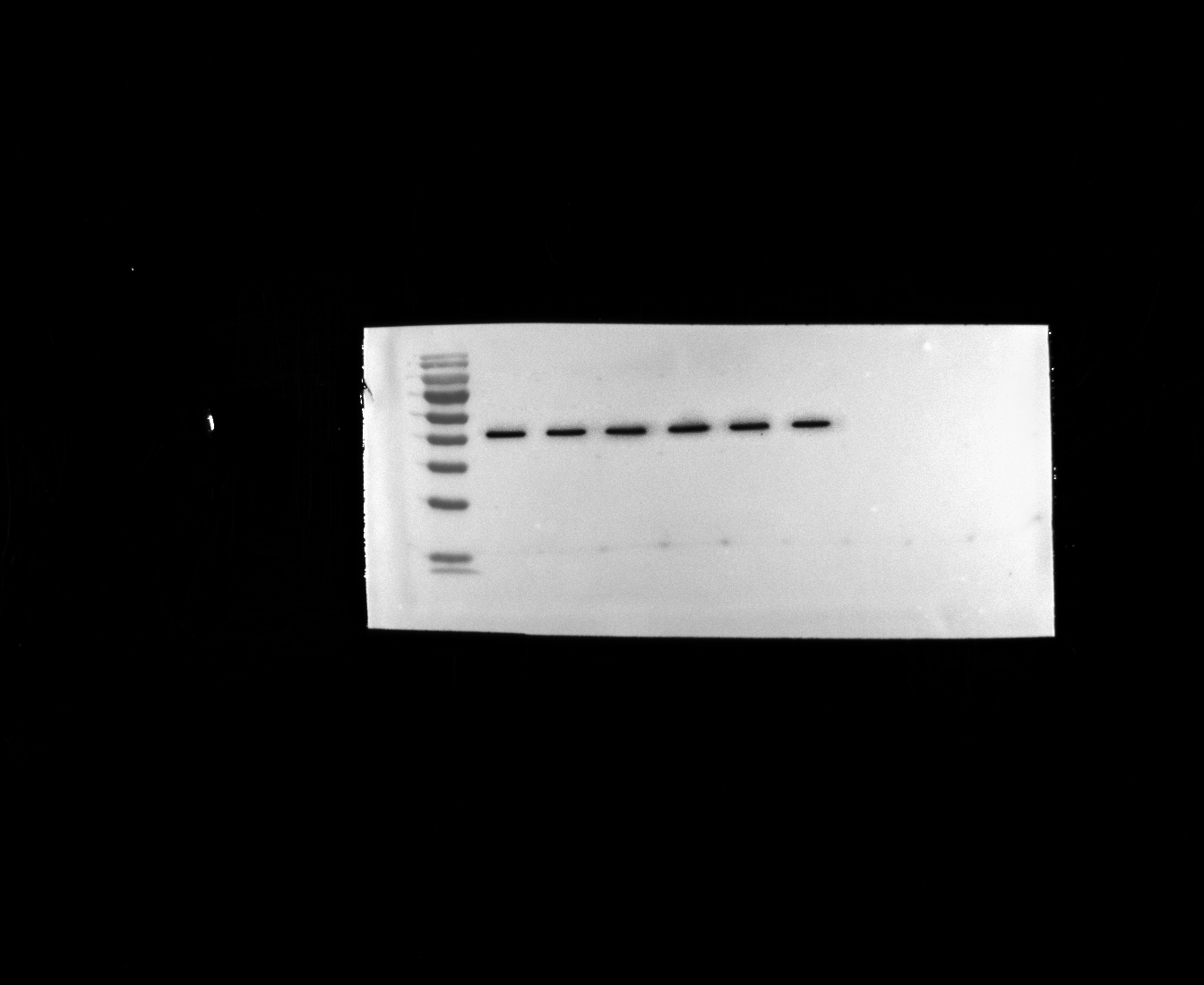

Supplement: Supplementary file 2 — Supplementary Material 2. [file 12935_2025_3665_MOESM2_ESM.zip › Supplementary Material 2/Figure 5/Figure 5C/MEK.tif]

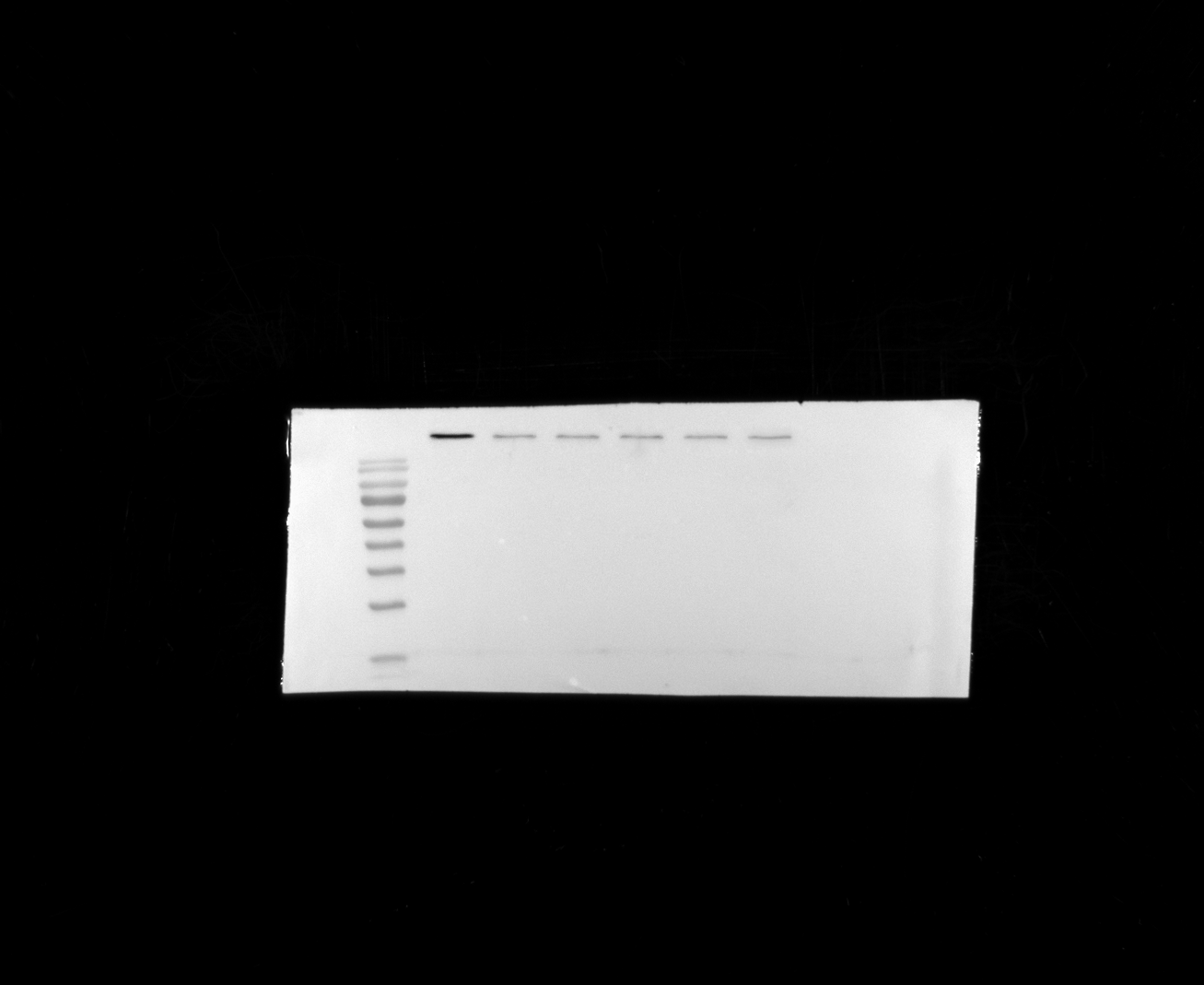

Supplement: Supplementary file 2 — Supplementary Material 2. [file 12935_2025_3665_MOESM2_ESM.zip › Supplementary Material 2/Figure 5/Figure 5C/p-EGFR(Y1069) .tif]

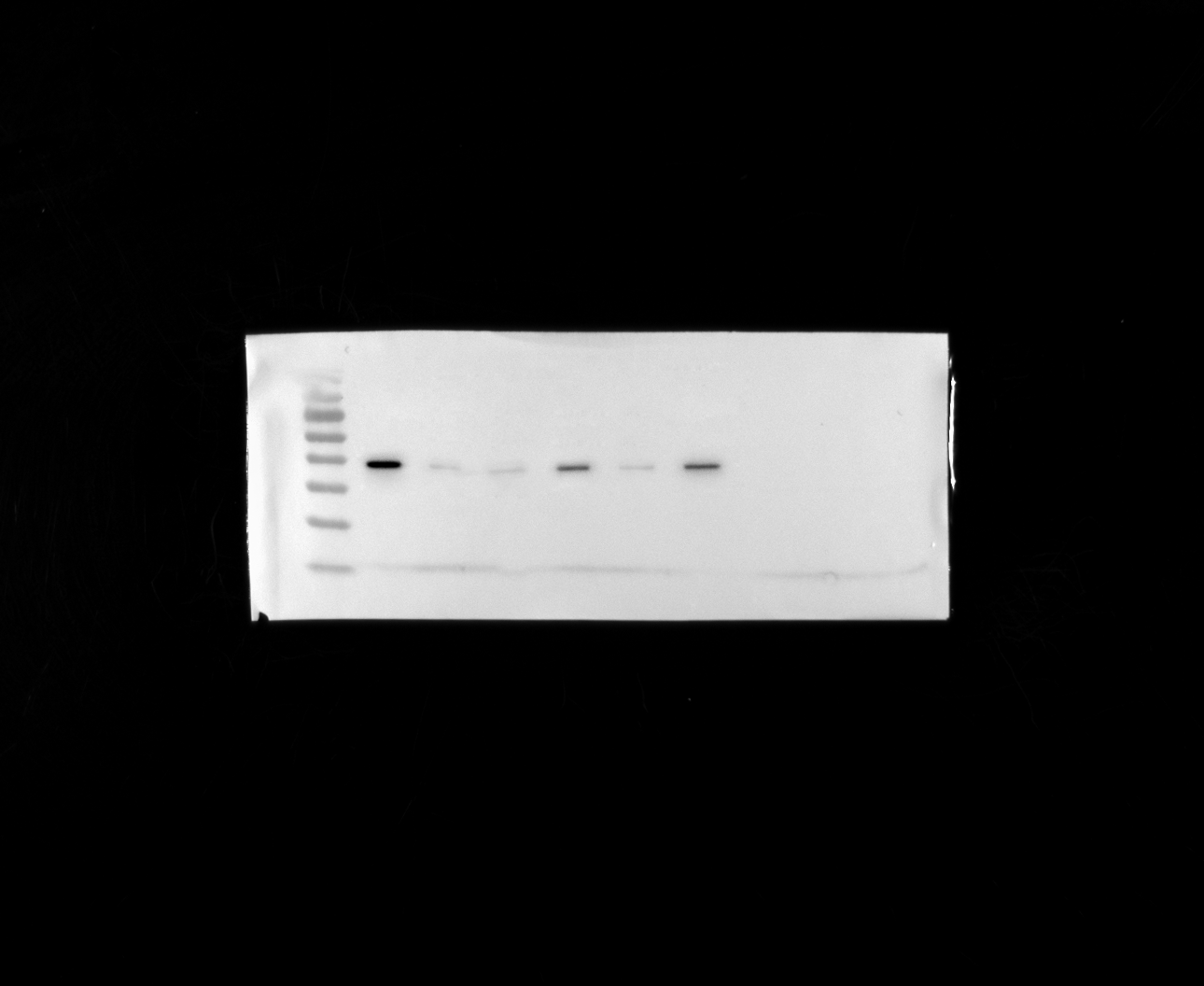

Supplement: Supplementary file 2 — Supplementary Material 2. [file 12935_2025_3665_MOESM2_ESM.zip › Supplementary Material 2/Figure 5/Figure 5C/p-ERK2(T185).tif]

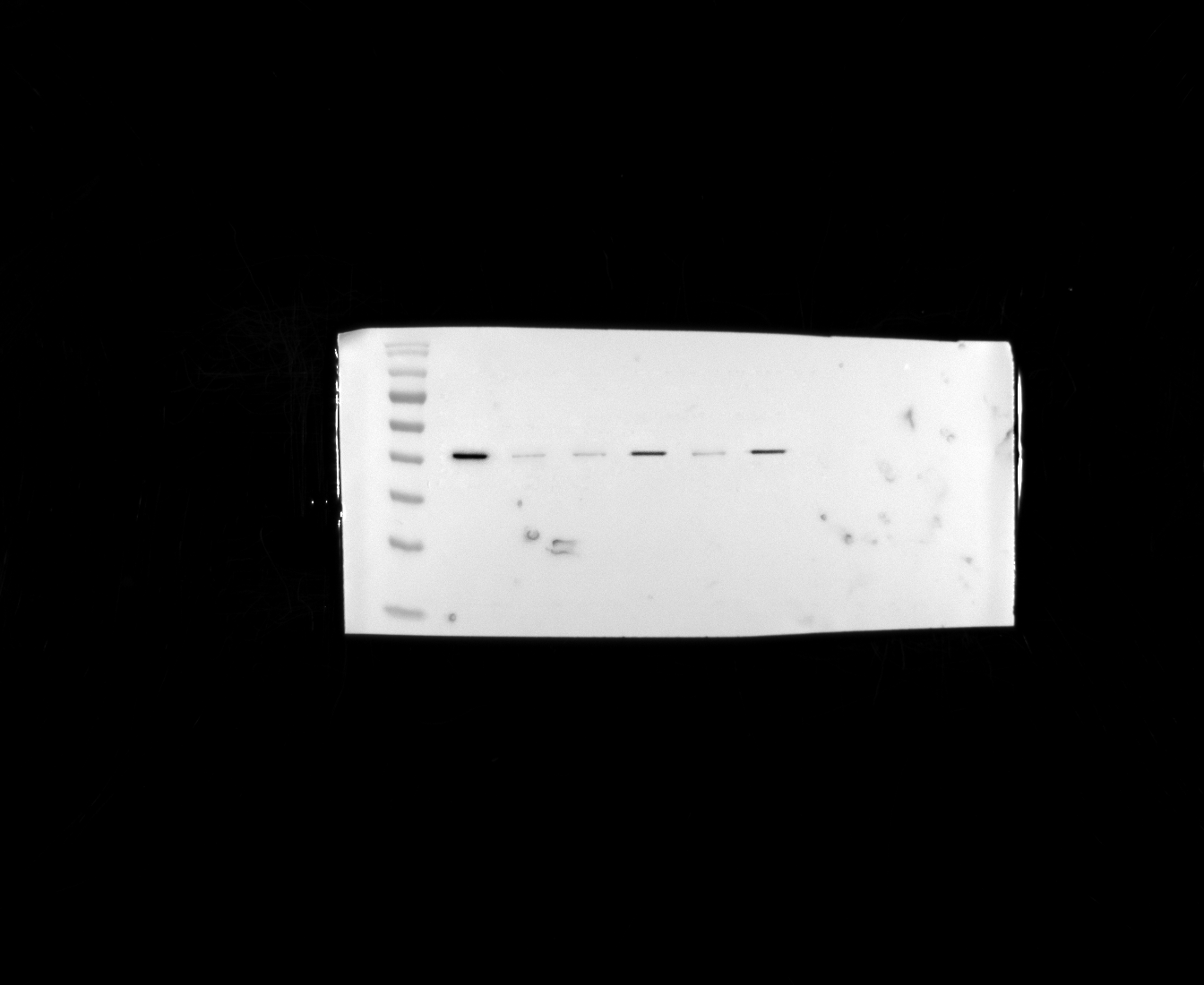

Supplement: Supplementary file 2 — Supplementary Material 2. [file 12935_2025_3665_MOESM2_ESM.zip › Supplementary Material 2/Figure 5/Figure 5C/P-MEK.tif]

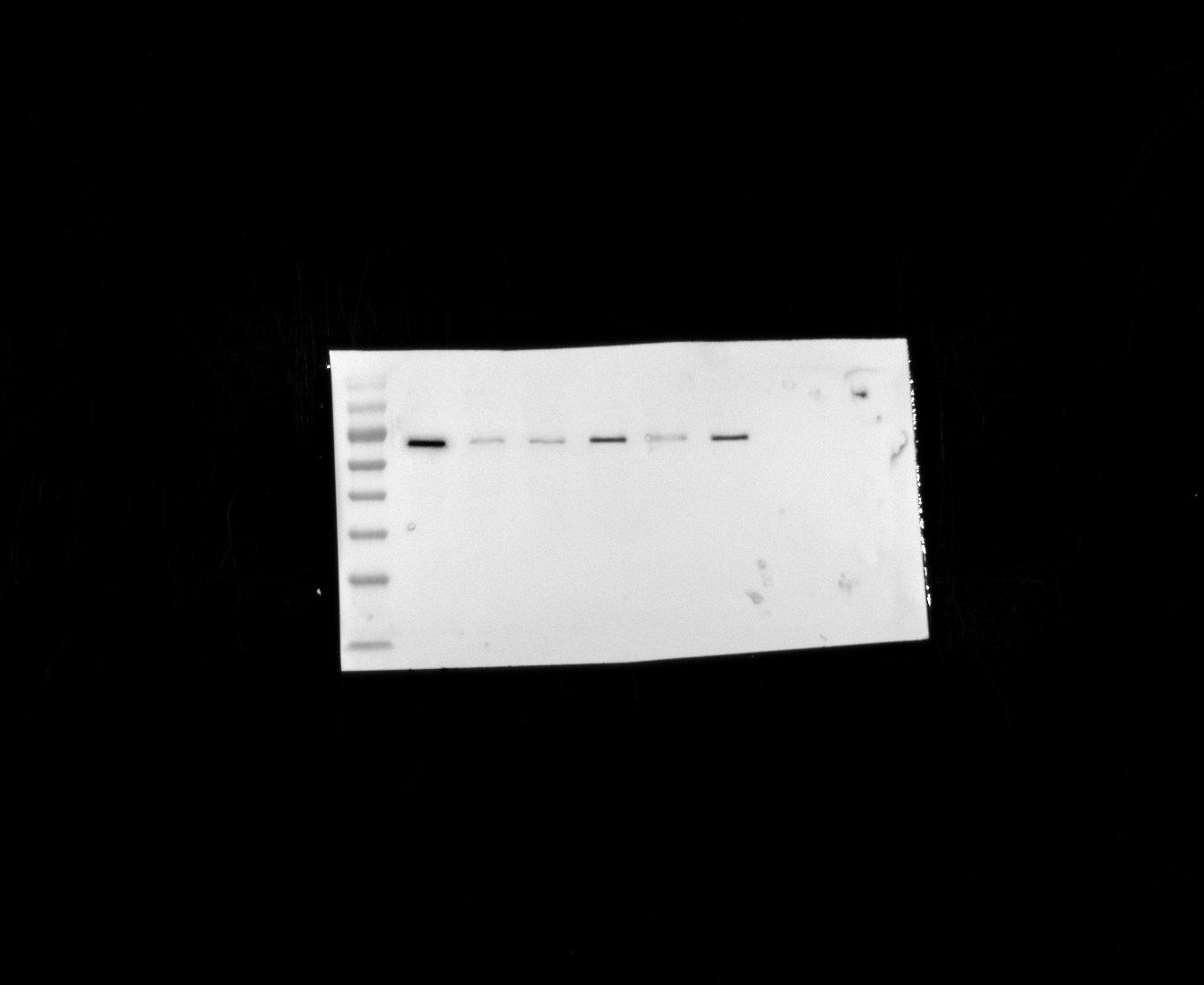

Supplement: Supplementary file 2 — Supplementary Material 2. [file 12935_2025_3665_MOESM2_ESM.zip › Supplementary Material 2/Figure 5/Figure 5C/RAF.tif]

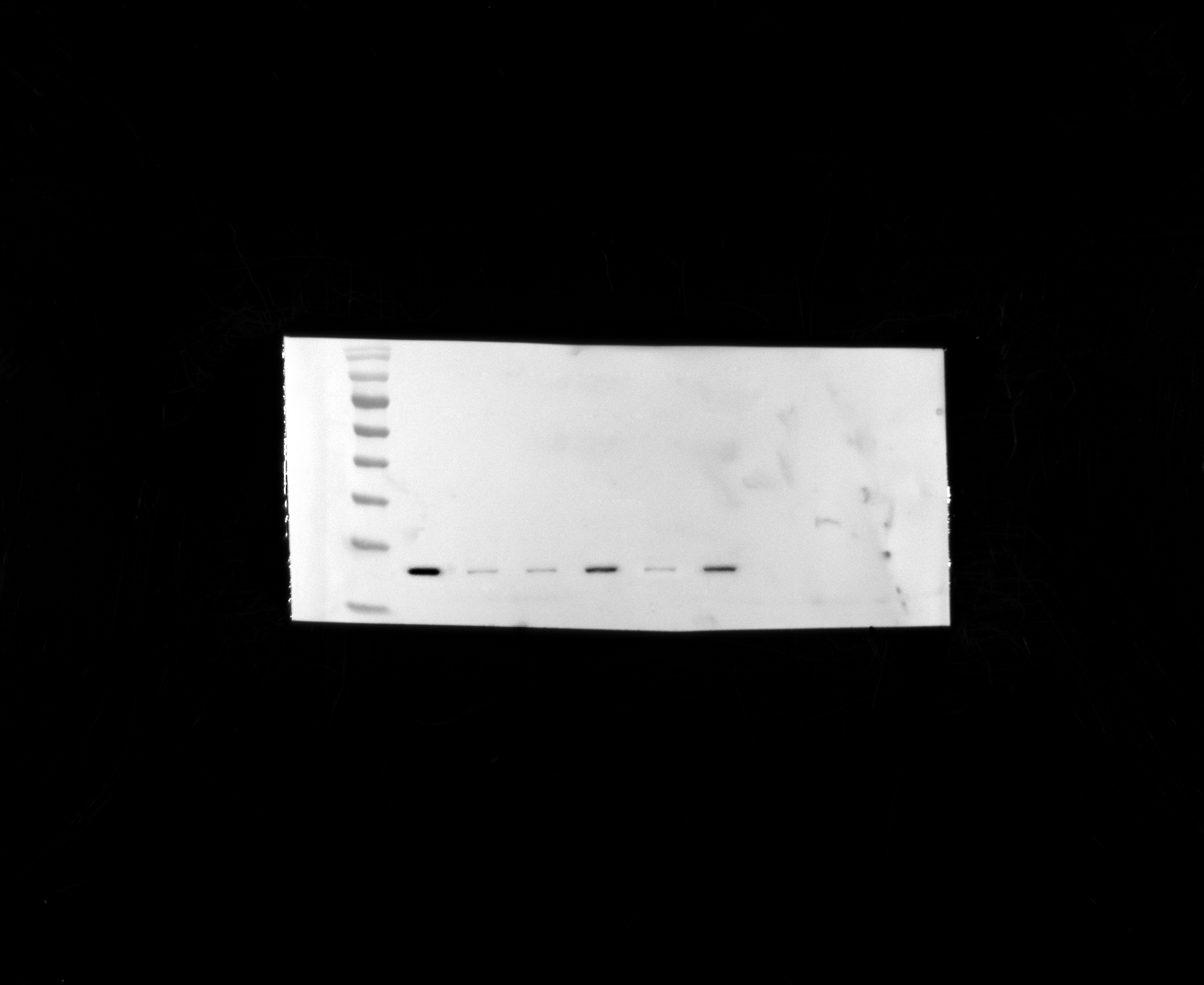

Supplement: Supplementary file 2 — Supplementary Material 2. [file 12935_2025_3665_MOESM2_ESM.zip › Supplementary Material 2/Figure 5/Figure 5C/RAS.tif]

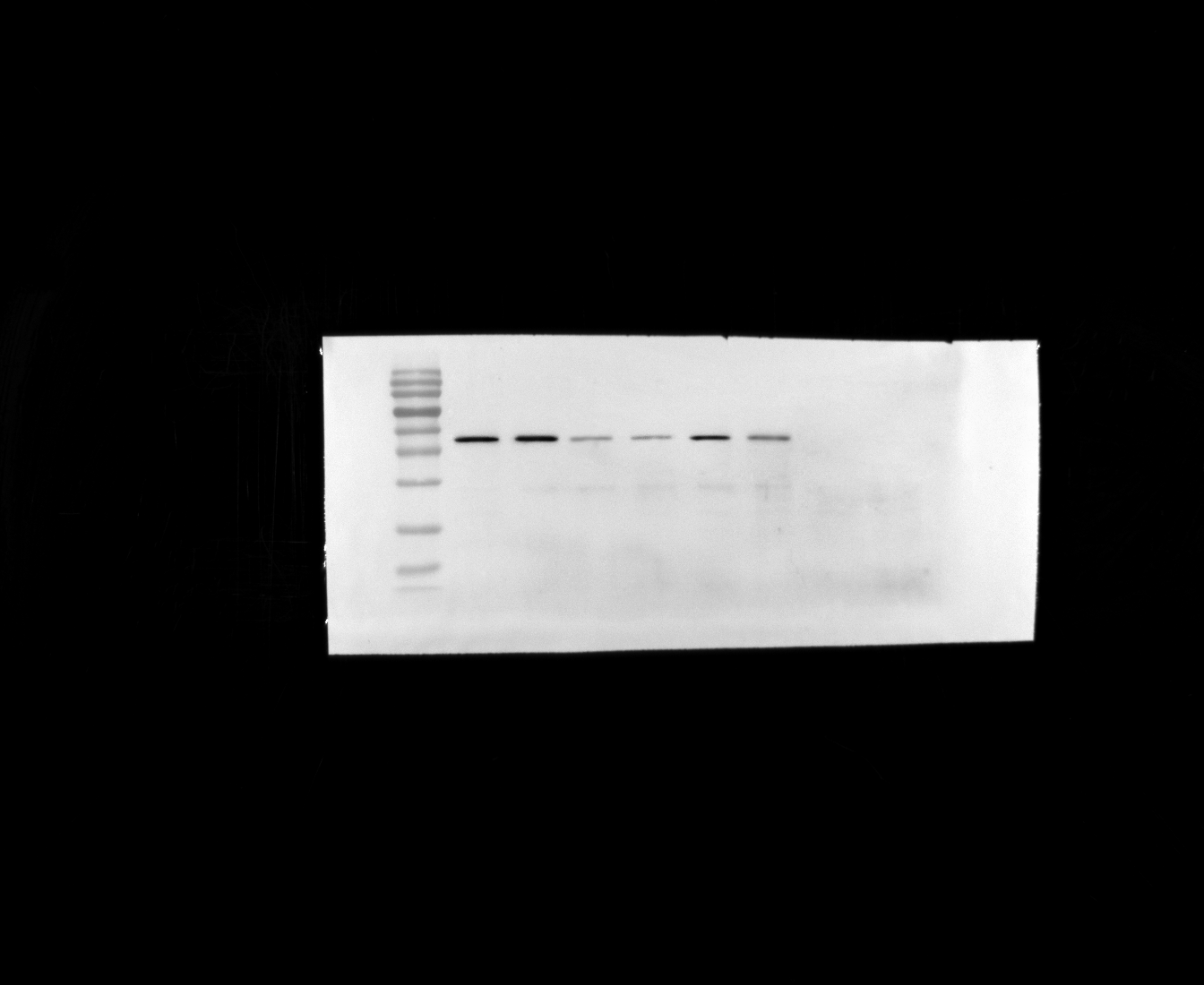

Supplement: Supplementary file 2 — Supplementary Material 2. [file 12935_2025_3665_MOESM2_ESM.zip › Supplementary Material 2/Figure 5/Figure 5D/MEK.tif]

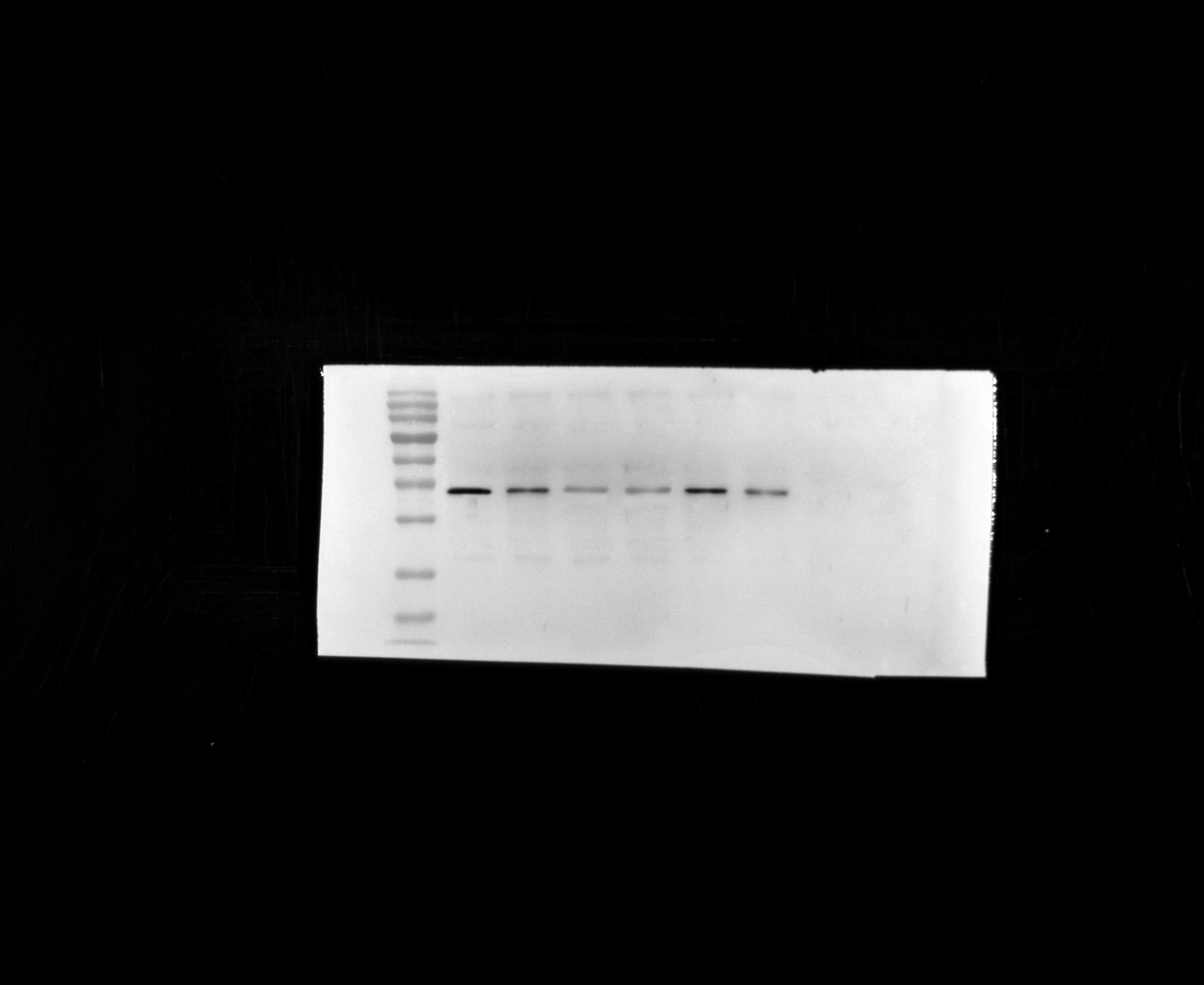

Supplement: Supplementary file 2 — Supplementary Material 2. [file 12935_2025_3665_MOESM2_ESM.zip › Supplementary Material 2/Figure 5/Figure 5D/p-ERK2.tif]

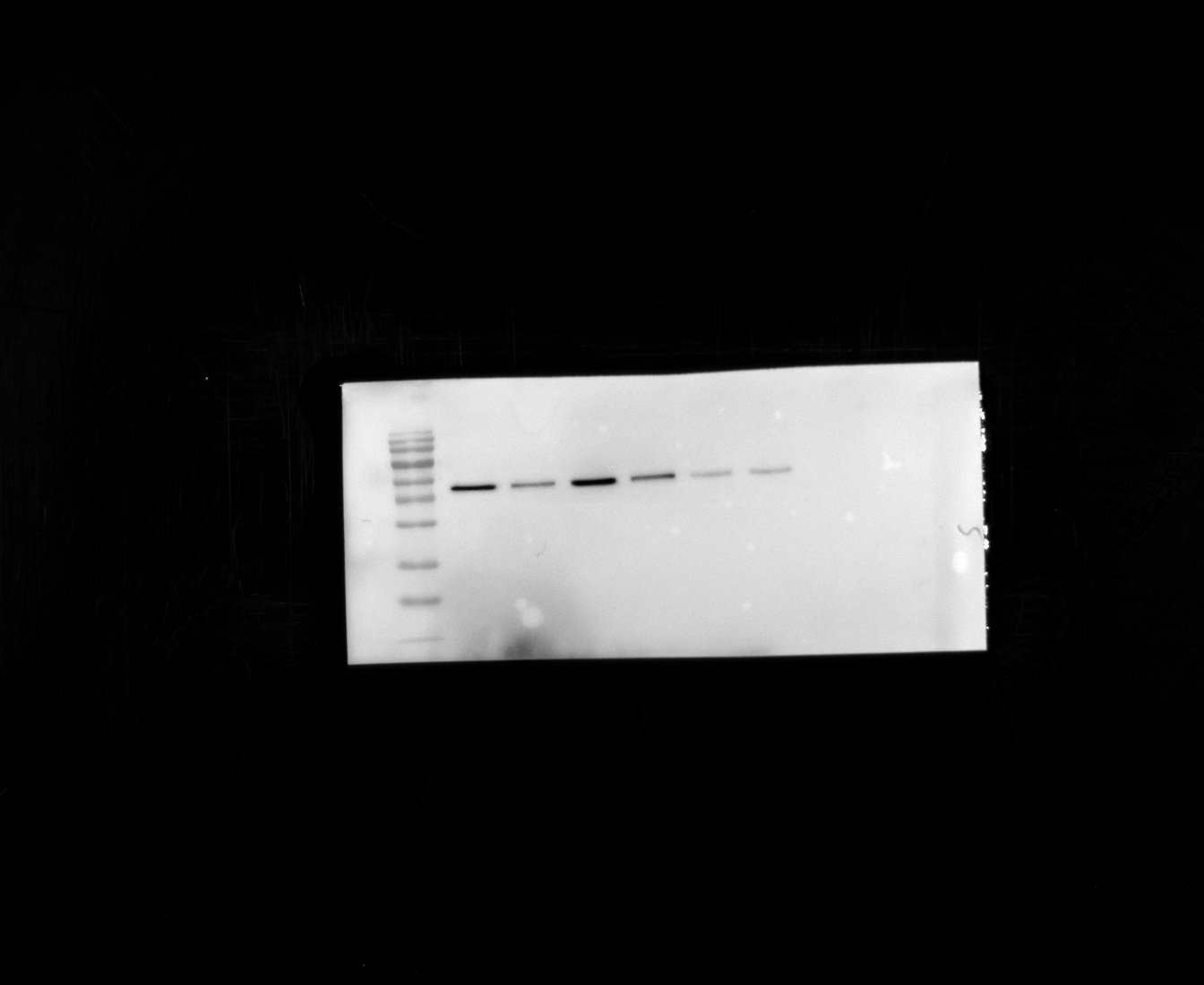

Supplement: Supplementary file 2 — Supplementary Material 2. [file 12935_2025_3665_MOESM2_ESM.zip › Supplementary Material 2/Figure 5/Figure 5E/Ets-1.tif]

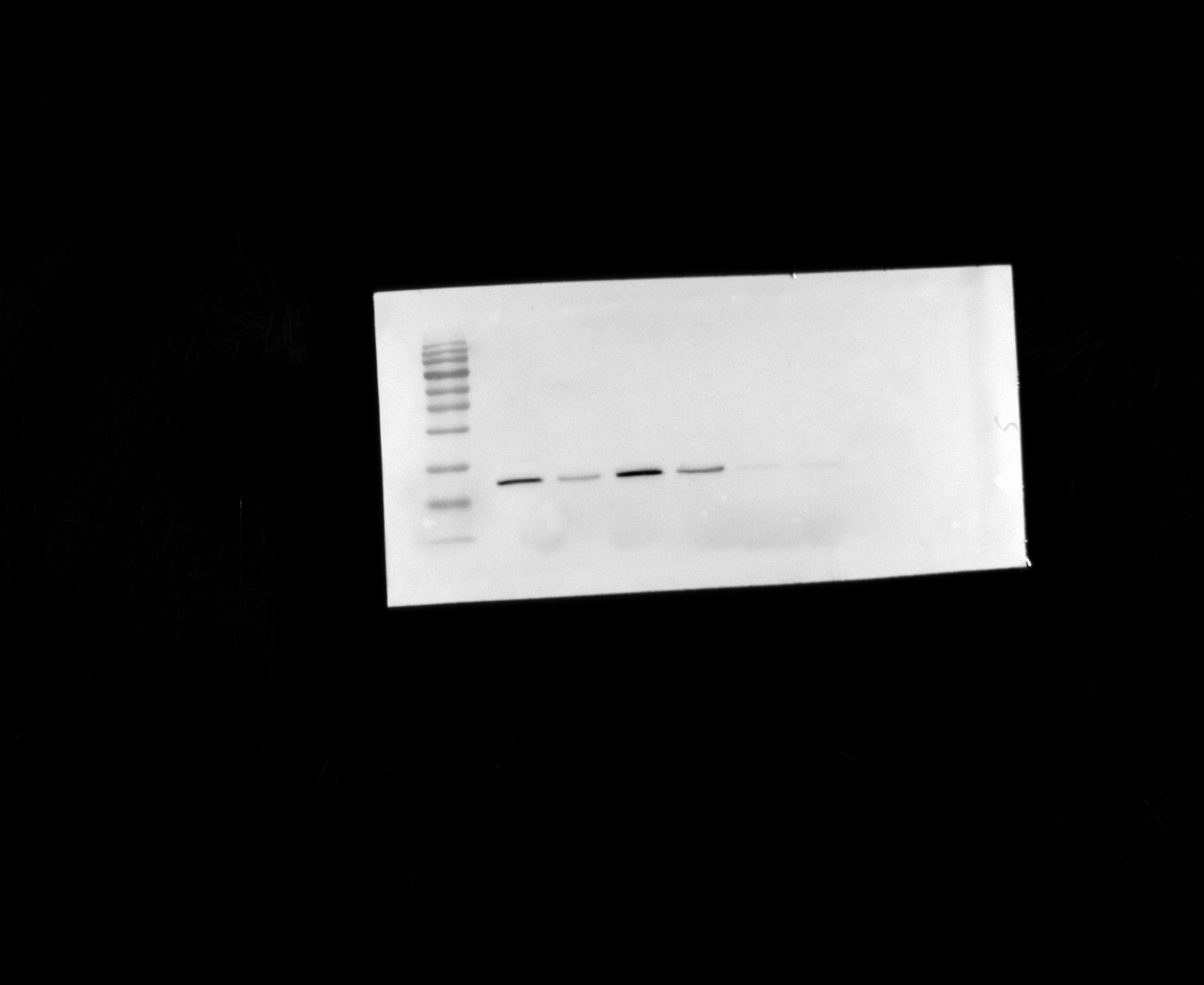

Supplement: Supplementary file 2 — Supplementary Material 2. [file 12935_2025_3665_MOESM2_ESM.zip › Supplementary Material 2/Figure 5/Figure 5E/Ferritin.tif]

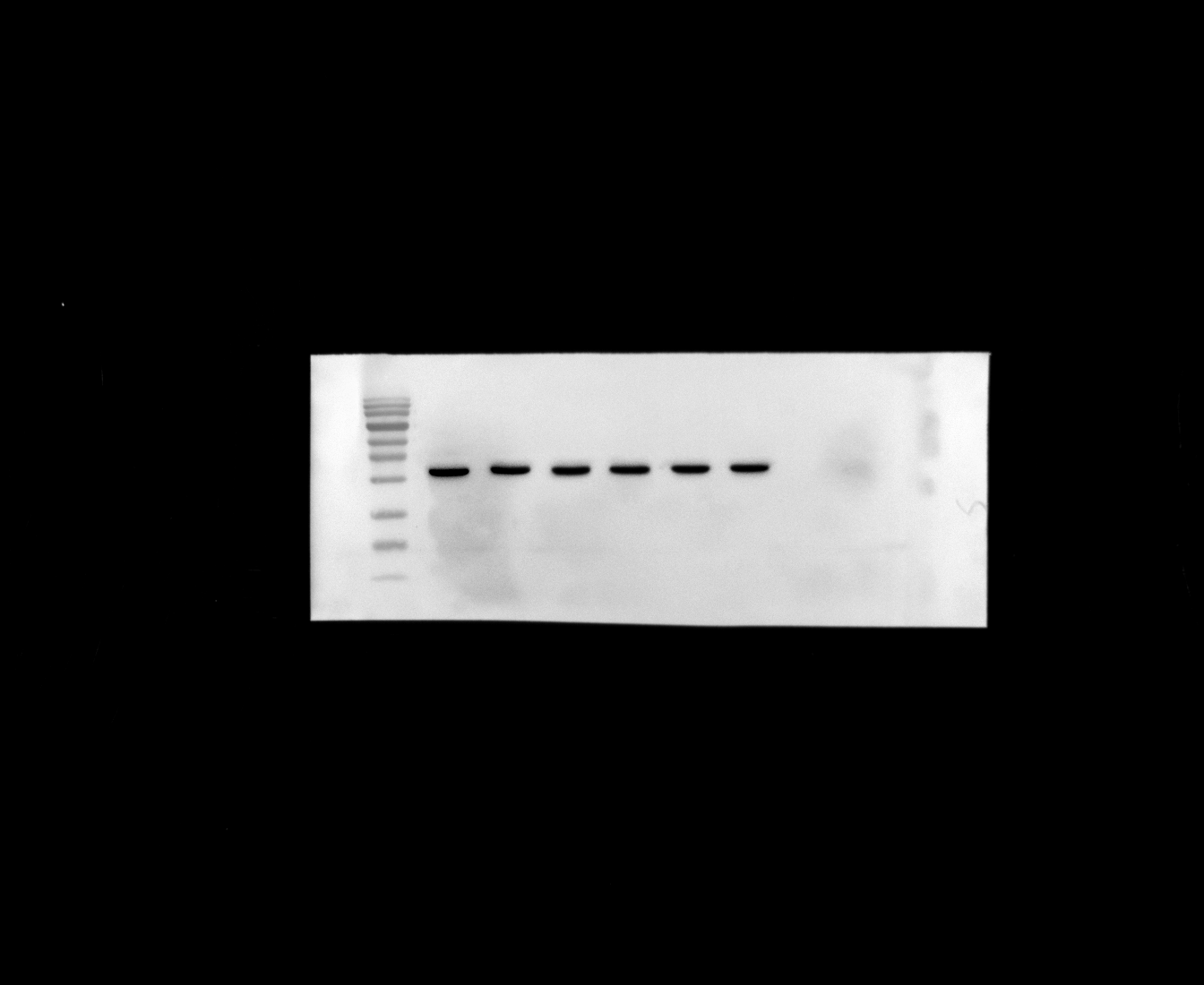

Supplement: Supplementary file 2 — Supplementary Material 2. [file 12935_2025_3665_MOESM2_ESM.zip › Supplementary Material 2/Figure 5/Figure 5E/GAPDH.tif]

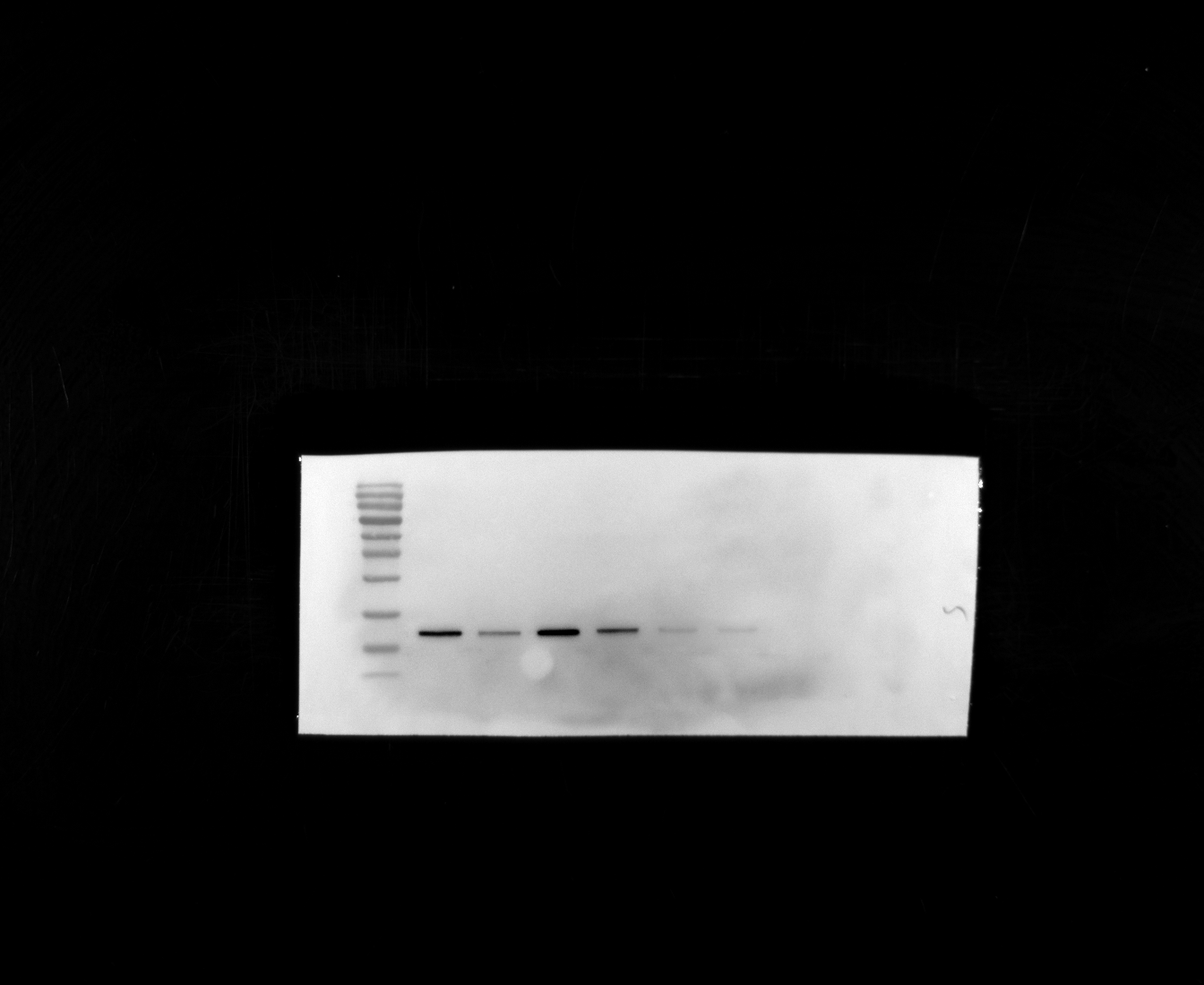

Supplement: Supplementary file 2 — Supplementary Material 2. [file 12935_2025_3665_MOESM2_ESM.zip › Supplementary Material 2/Figure 5/Figure 5E/GPX4.tif]

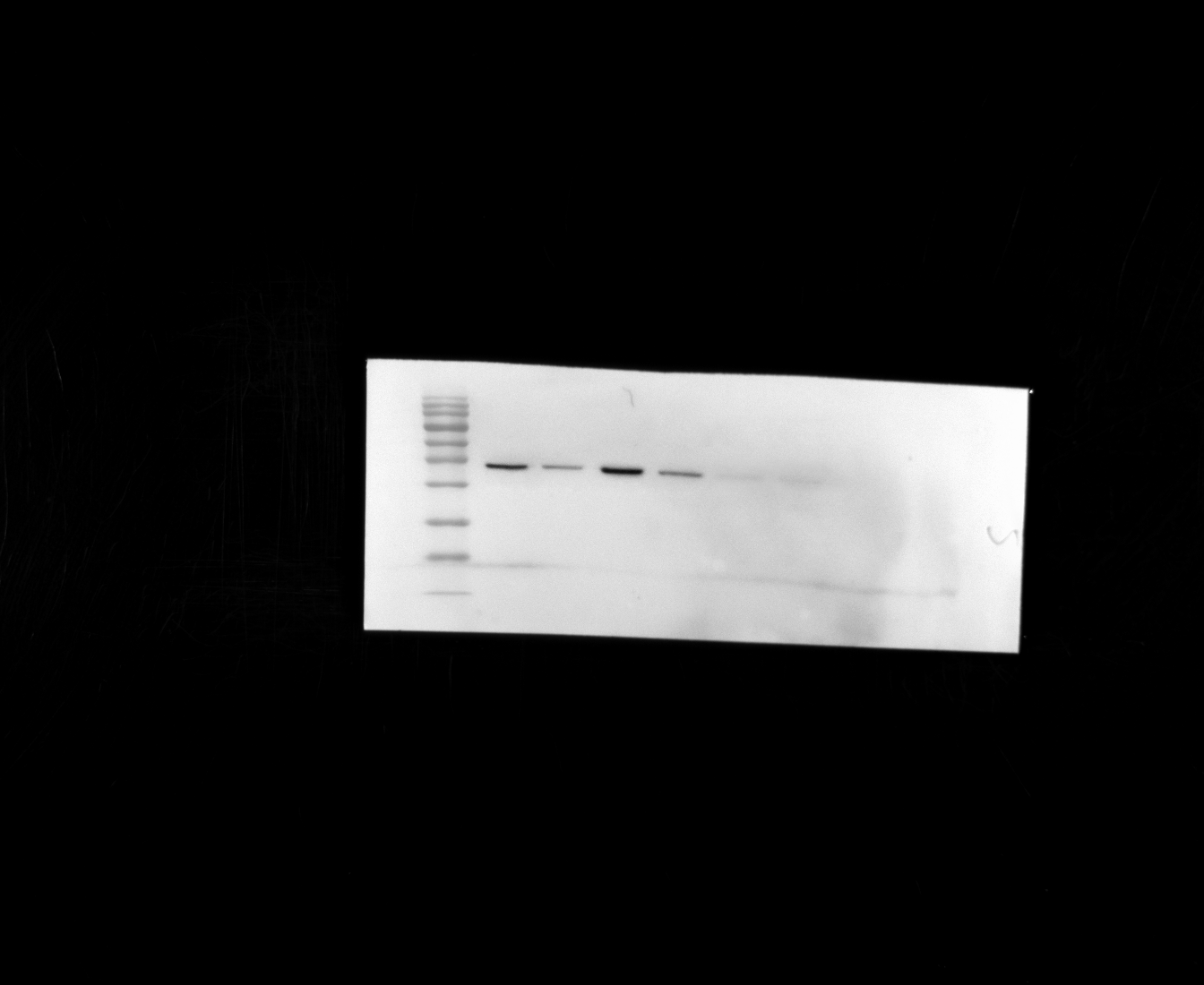

Supplement: Supplementary file 2 — Supplementary Material 2. [file 12935_2025_3665_MOESM2_ESM.zip › Supplementary Material 2/Figure 5/Figure 5E/p-ERK2(T185).tif]

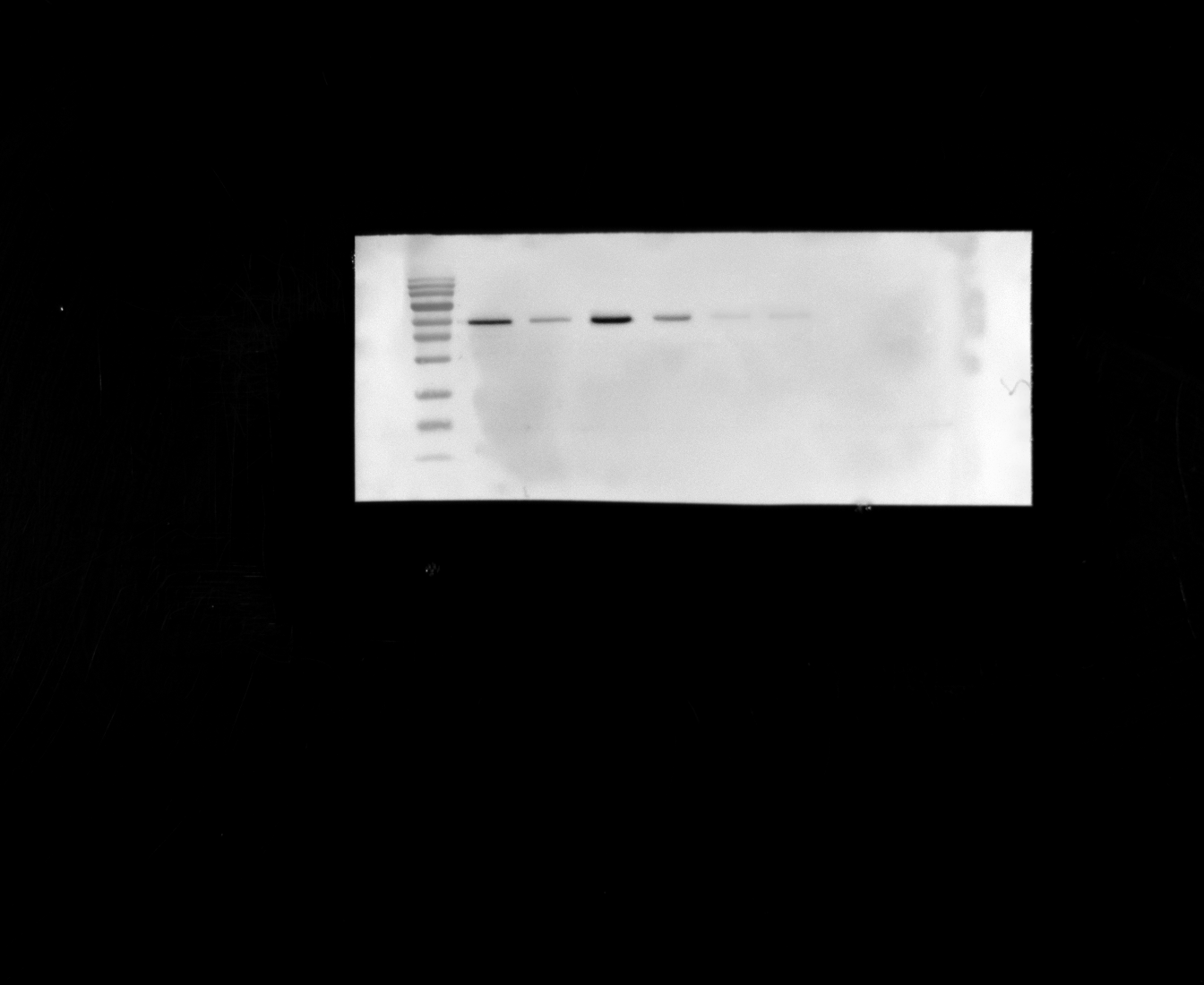

Supplement: Supplementary file 2 — Supplementary Material 2. [file 12935_2025_3665_MOESM2_ESM.zip › Supplementary Material 2/Figure 5/Figure 5E/SLC7A11.tif]

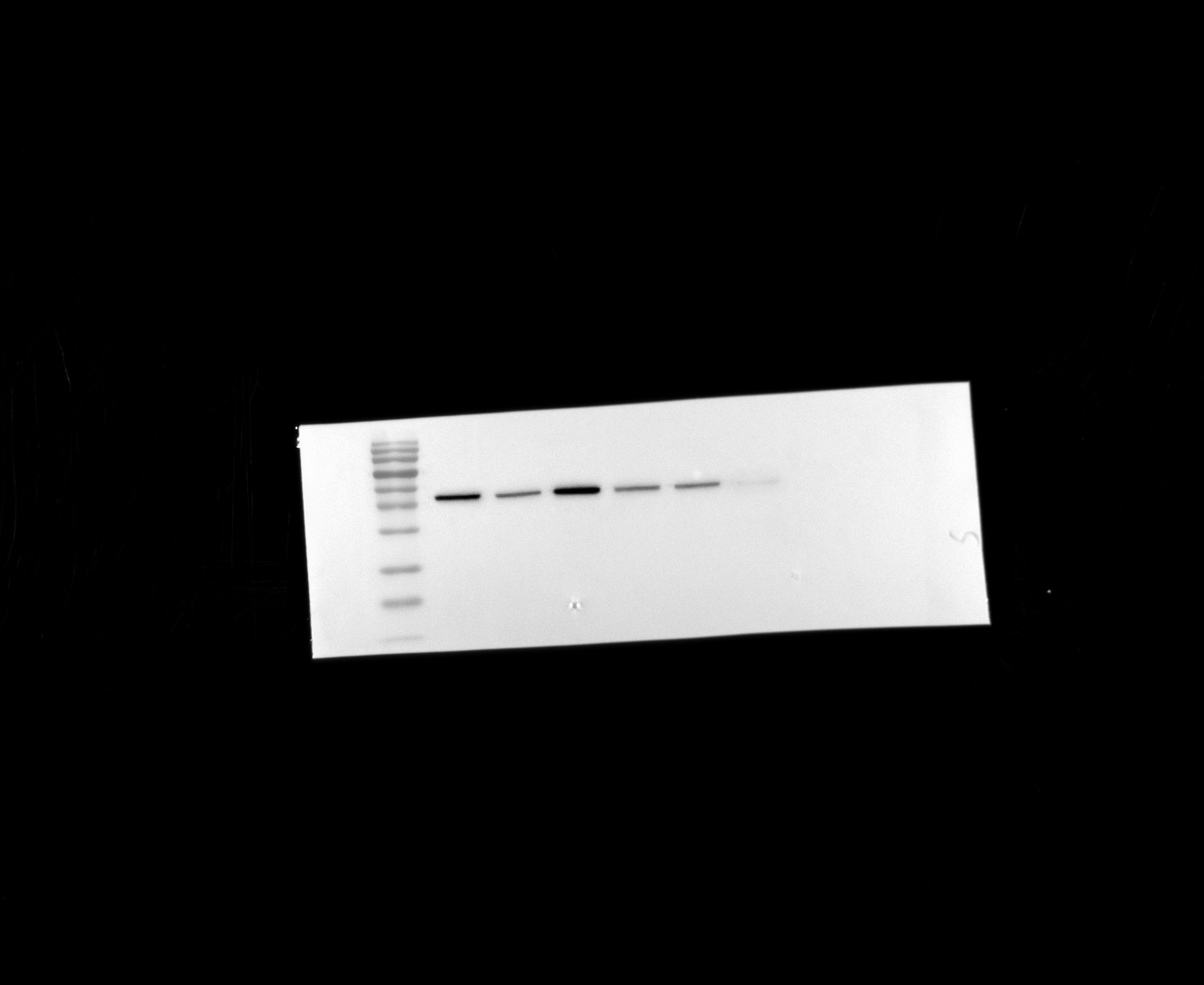

Supplement: Supplementary file 2 — Supplementary Material 2. [file 12935_2025_3665_MOESM2_ESM.zip › Supplementary Material 2/Figure 5/Figure 5G/Ets-1.tif]

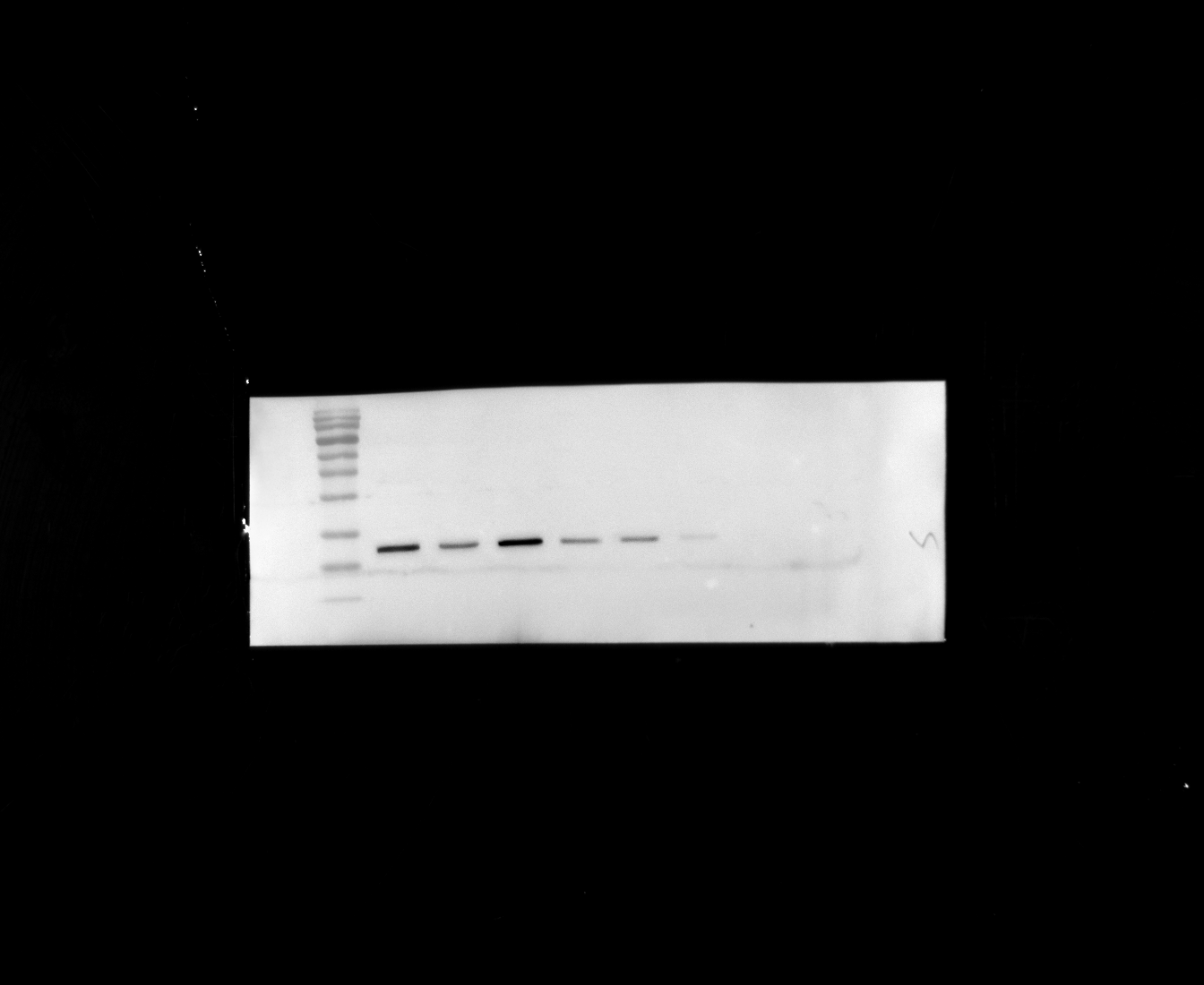

Supplement: Supplementary file 2 — Supplementary Material 2. [file 12935_2025_3665_MOESM2_ESM.zip › Supplementary Material 2/Figure 5/Figure 5G/Ferritin.tif]

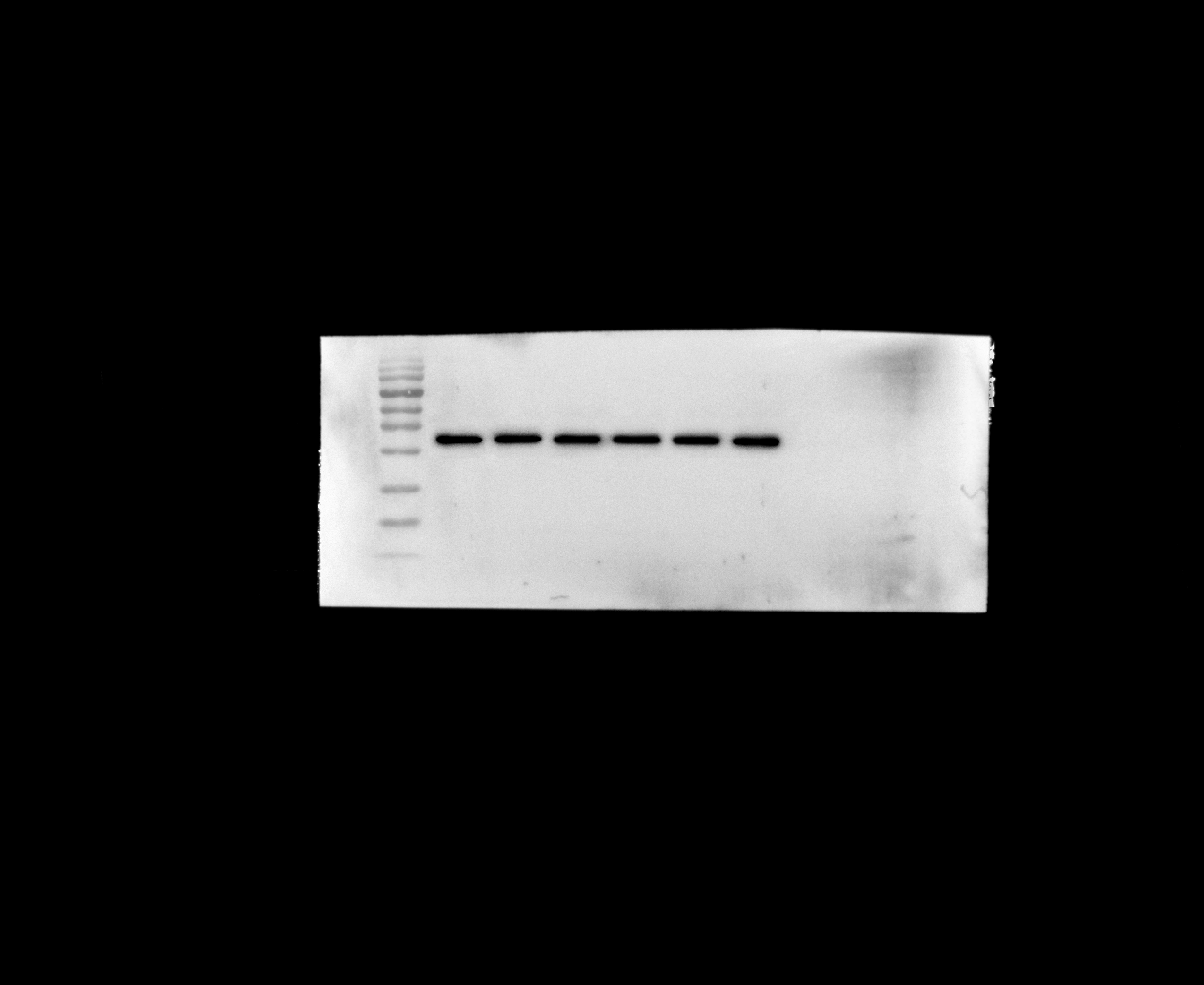

Supplement: Supplementary file 2 — Supplementary Material 2. [file 12935_2025_3665_MOESM2_ESM.zip › Supplementary Material 2/Figure 5/Figure 5G/GPADH.tif]

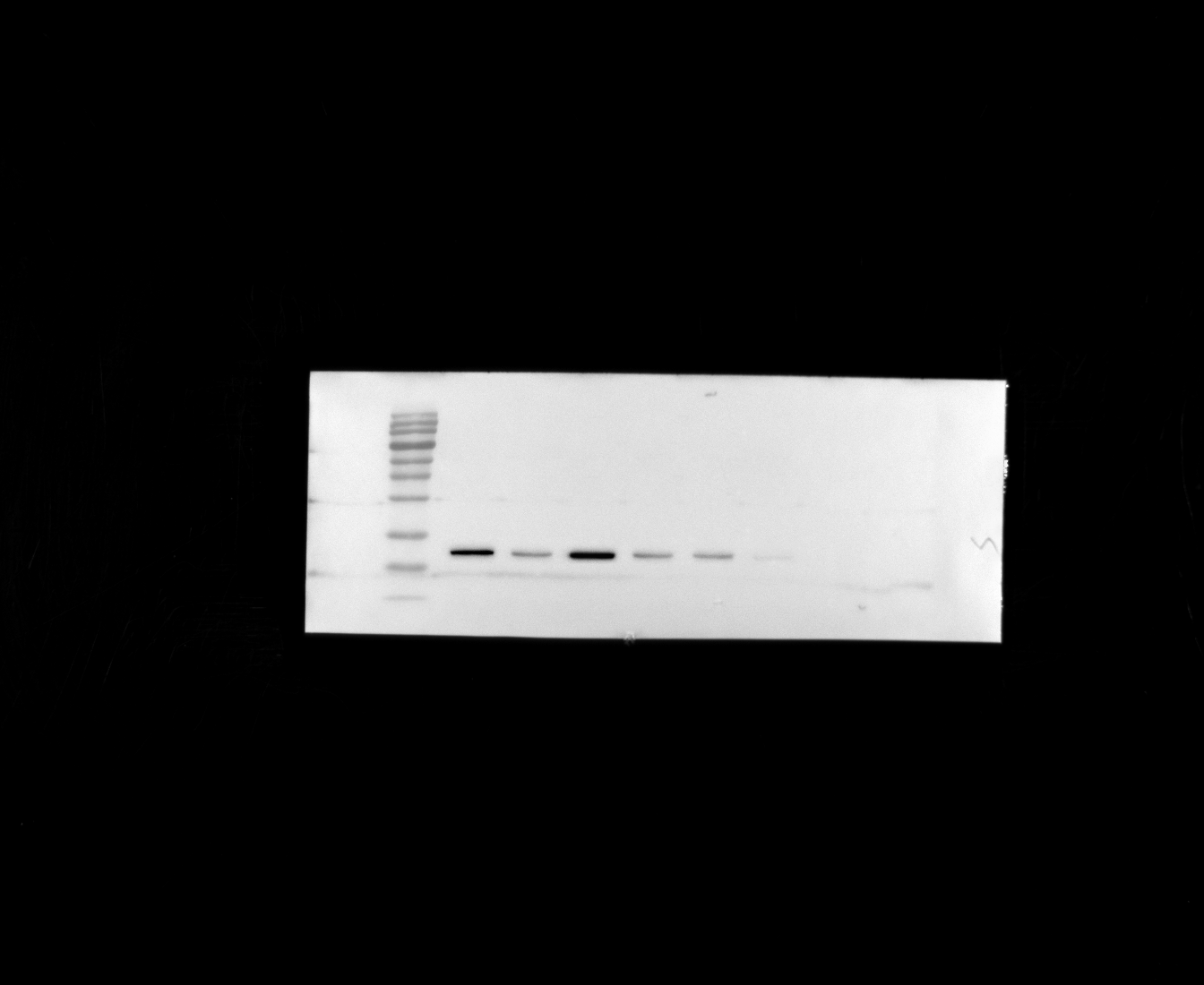

Supplement: Supplementary file 2 — Supplementary Material 2. [file 12935_2025_3665_MOESM2_ESM.zip › Supplementary Material 2/Figure 5/Figure 5G/GPX4.tif]

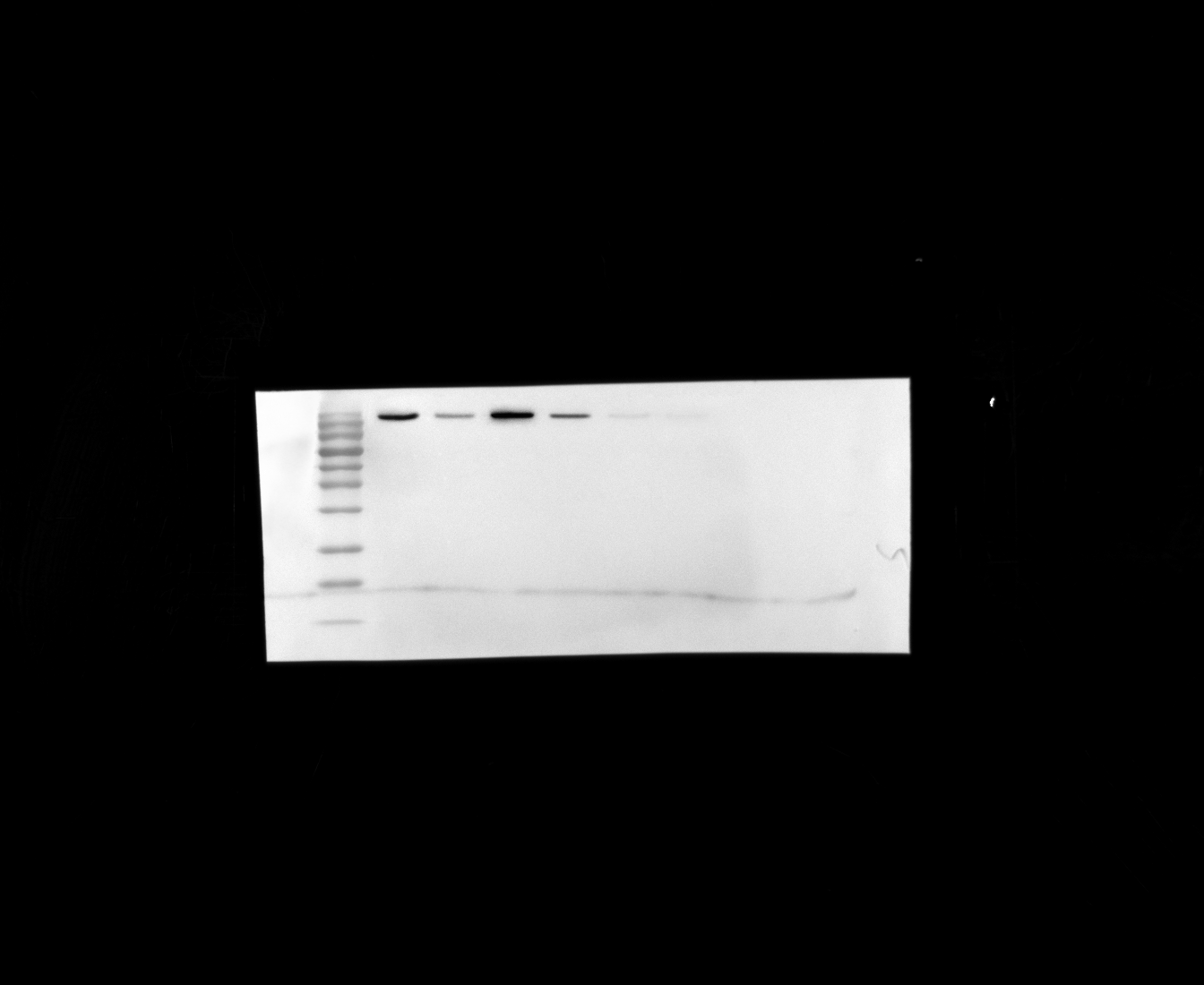

Supplement: Supplementary file 2 — Supplementary Material 2. [file 12935_2025_3665_MOESM2_ESM.zip › Supplementary Material 2/Figure 5/Figure 5G/p-EGFR(Y1069).tif]

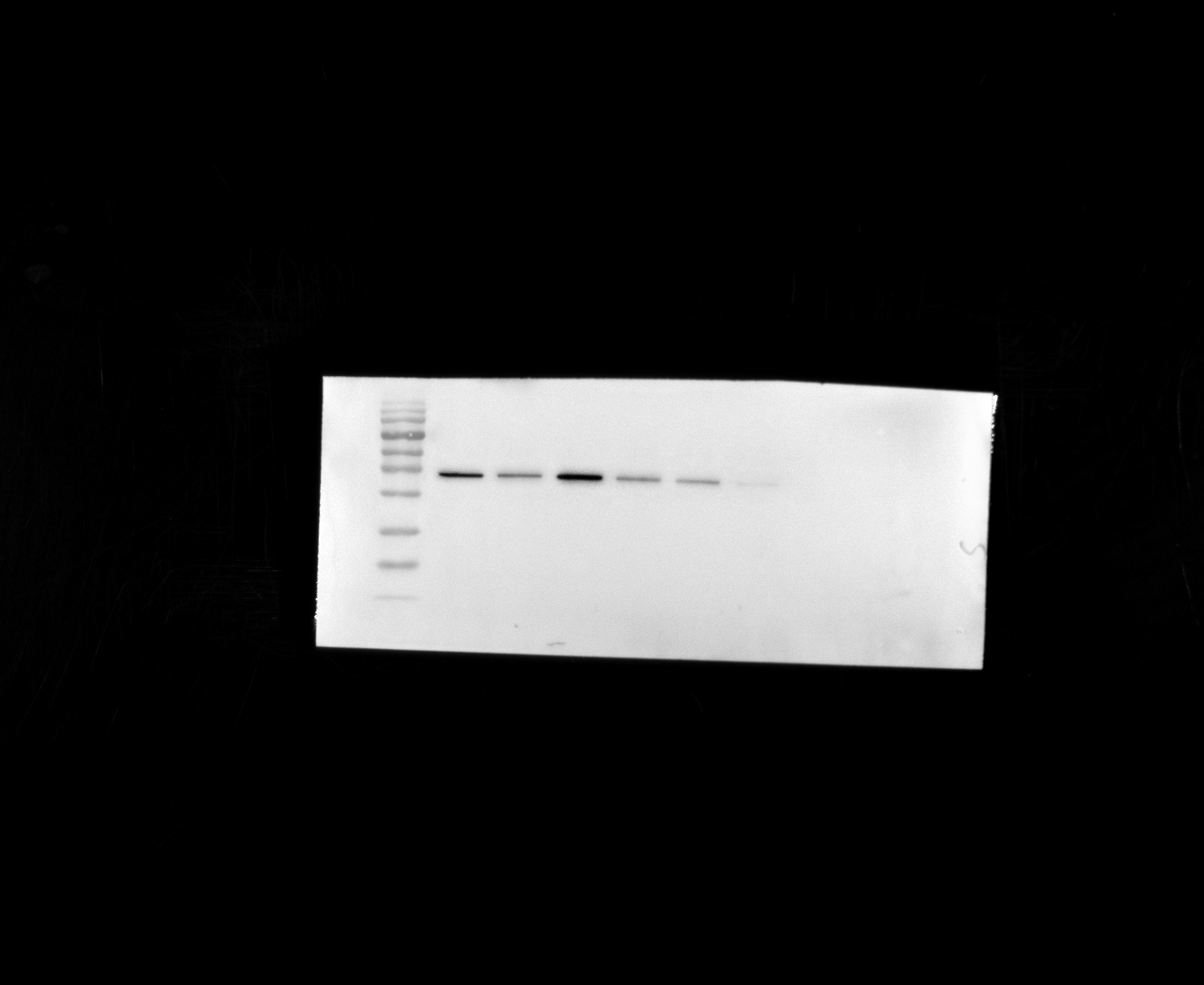

Supplement: Supplementary file 2 — Supplementary Material 2. [file 12935_2025_3665_MOESM2_ESM.zip › Supplementary Material 2/Figure 5/Figure 5G/P-ERK2(T185 ).tif]

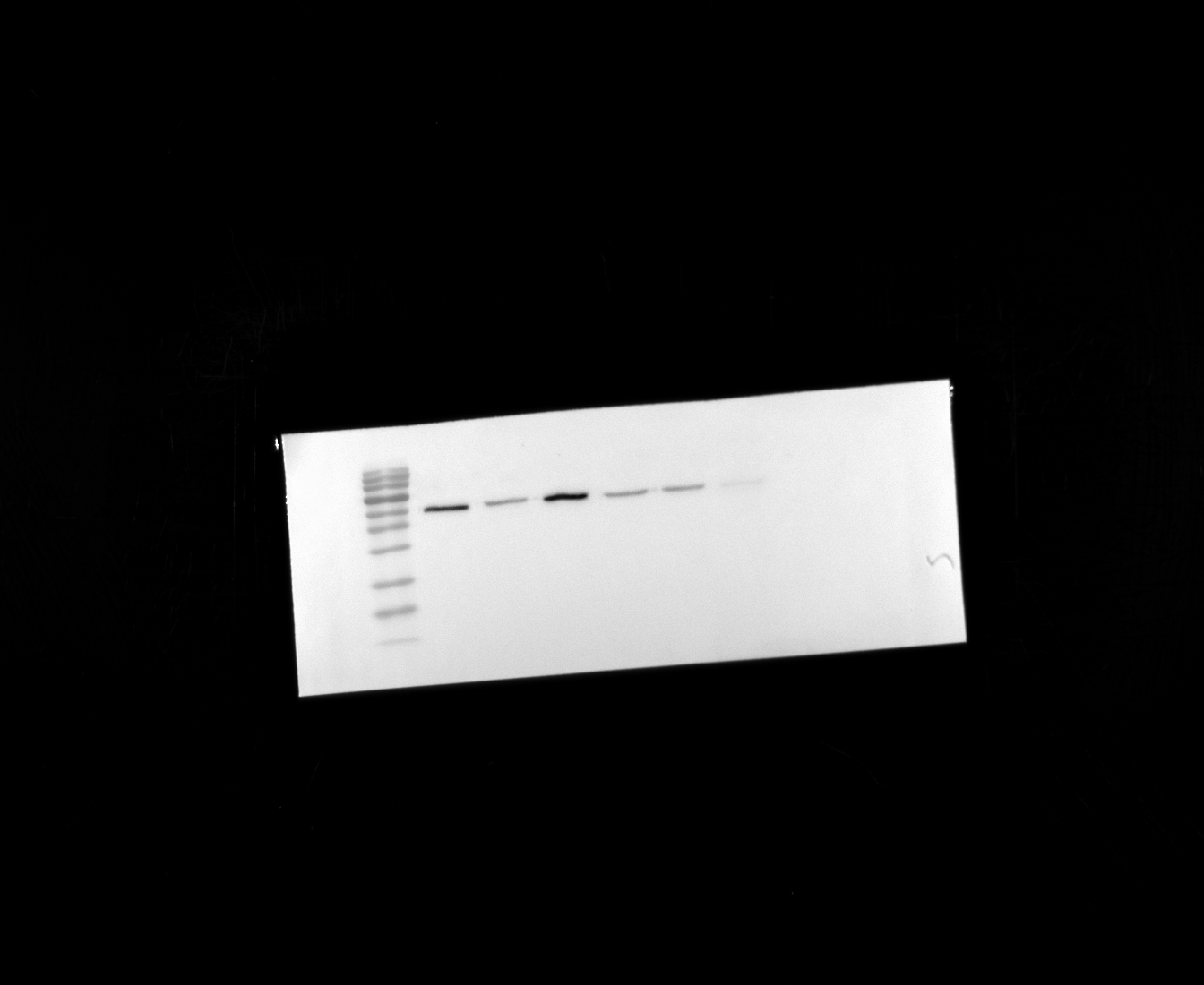

Supplement: Supplementary file 2 — Supplementary Material 2. [file 12935_2025_3665_MOESM2_ESM.zip › Supplementary Material 2/Figure 5/Figure 5G/SLC7A11.tif]

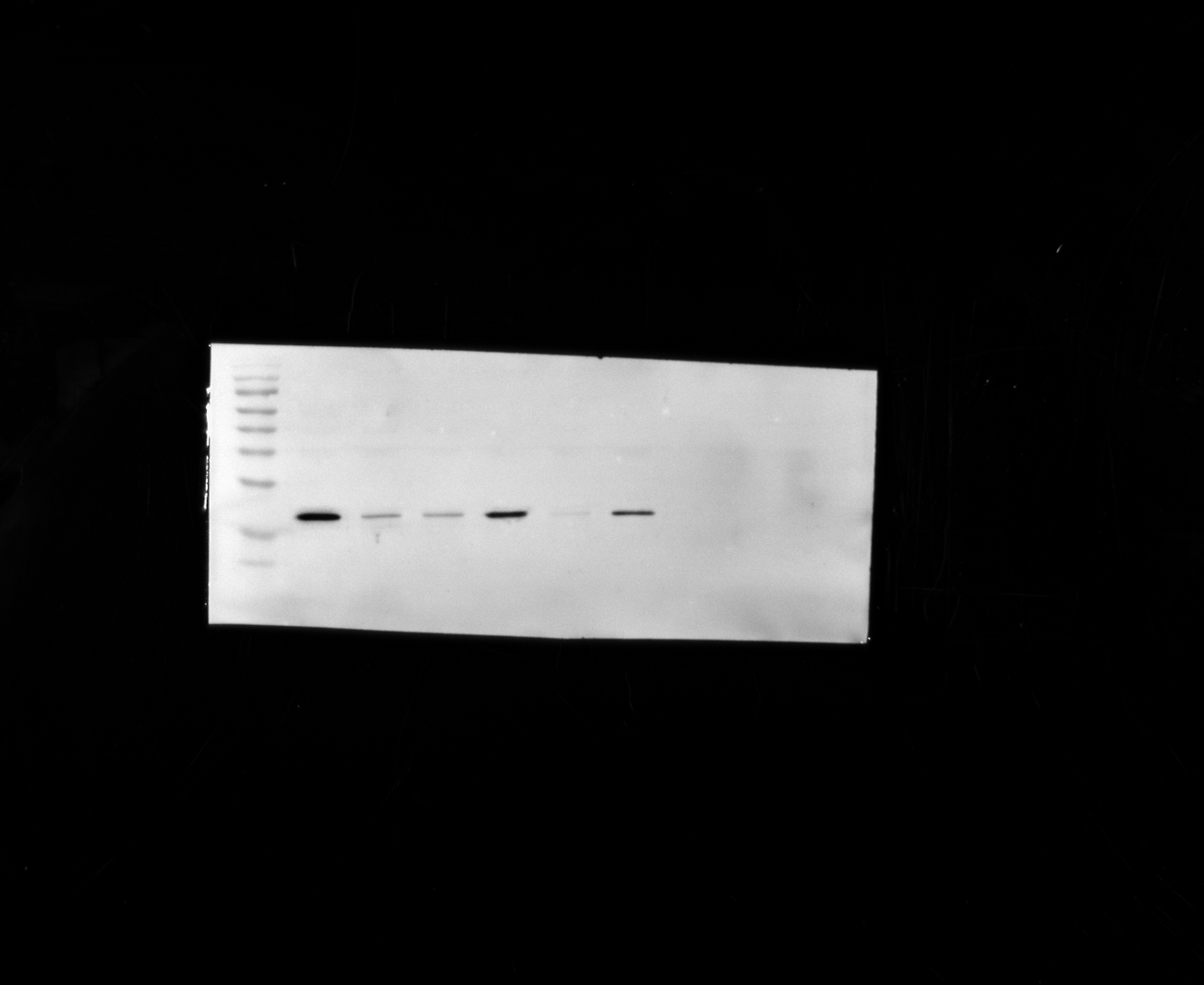

Supplement: Supplementary file 2 — Supplementary Material 2. [file 12935_2025_3665_MOESM2_ESM.zip › Supplementary Material 2/Figure 6/Figure 6A/GPX4.tif]

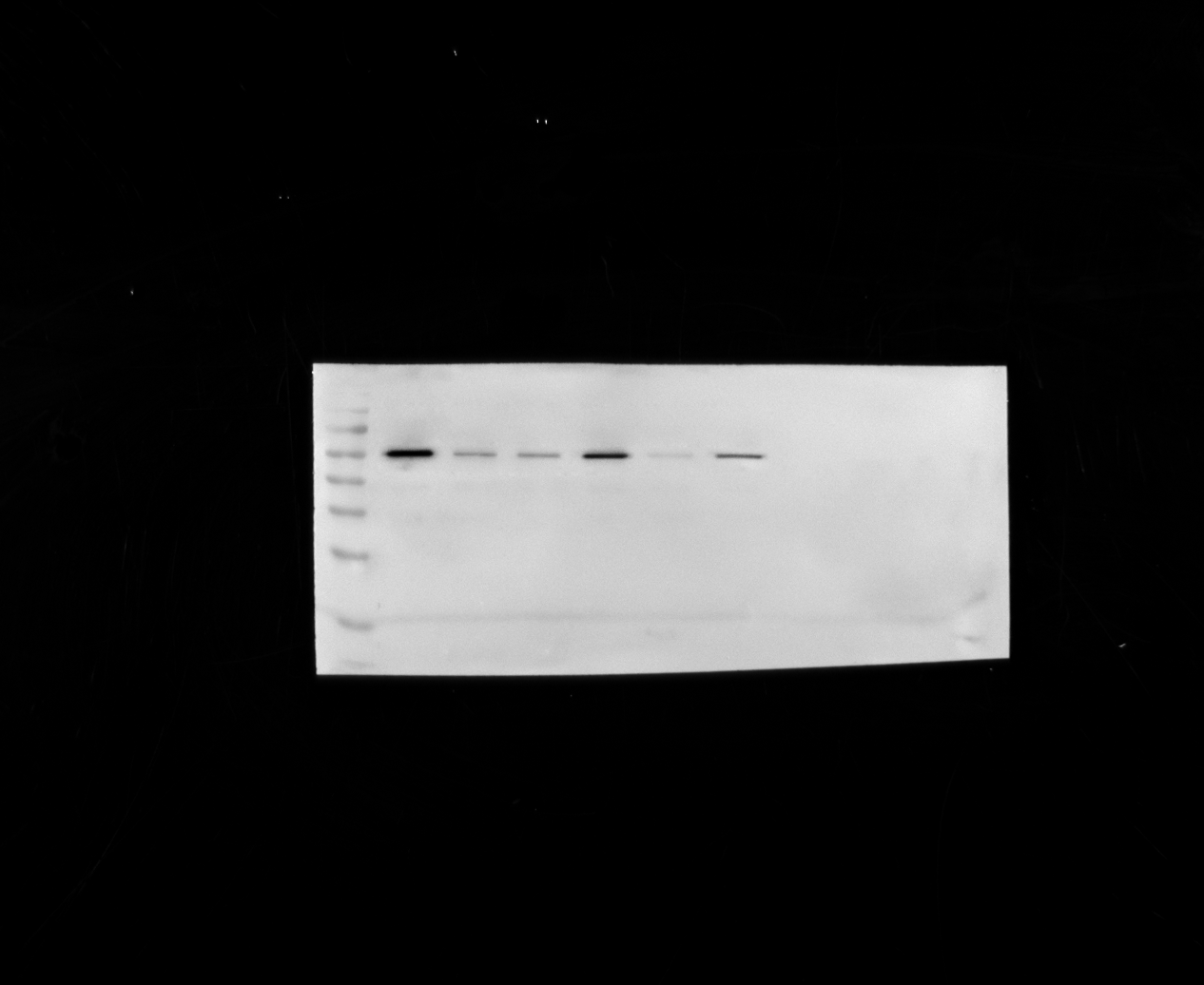

Supplement: Supplementary file 2 — Supplementary Material 2. [file 12935_2025_3665_MOESM2_ESM.zip › Supplementary Material 2/Figure 6/Figure 6A/SLC7A11.tif]

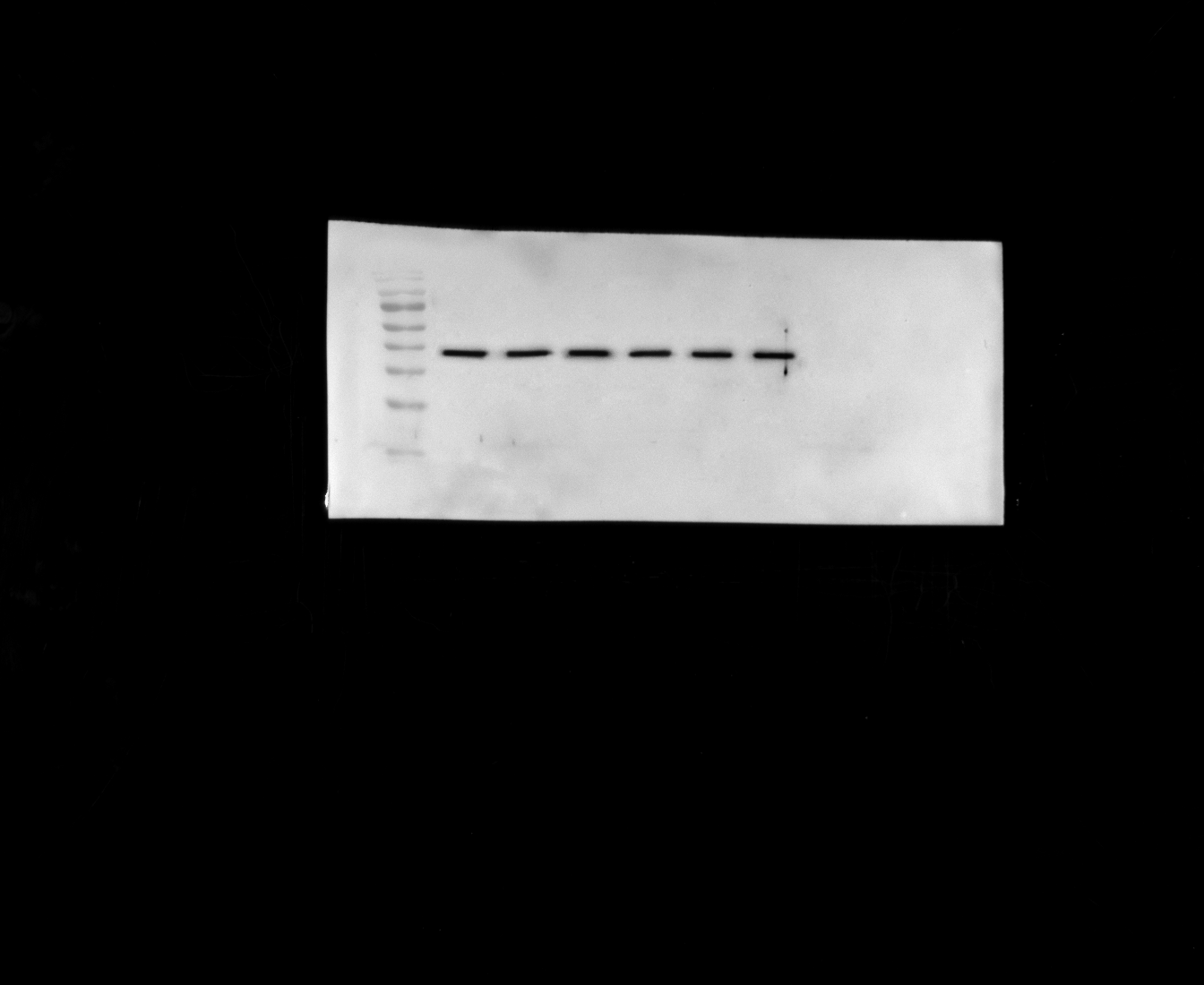

Supplement: Supplementary file 2 — Supplementary Material 2. [file 12935_2025_3665_MOESM2_ESM.zip › Supplementary Material 2/Figure 6/Figure 6A/β-actin.tif]

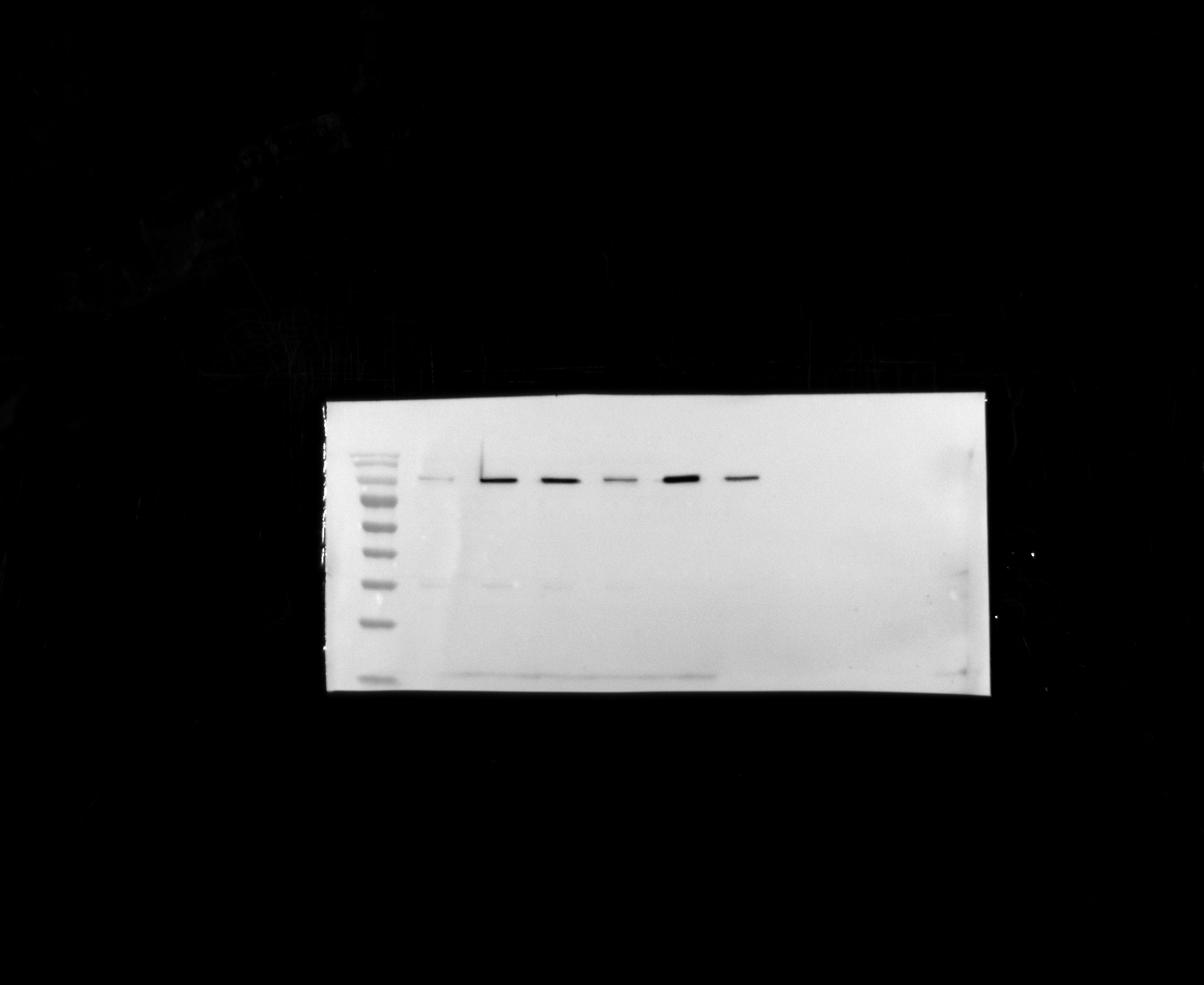

Supplement: Supplementary file 2 — Supplementary Material 2. [file 12935_2025_3665_MOESM2_ESM.zip › Supplementary Material 2/Figure 6/Figure 6K/E-cadherin.tif]

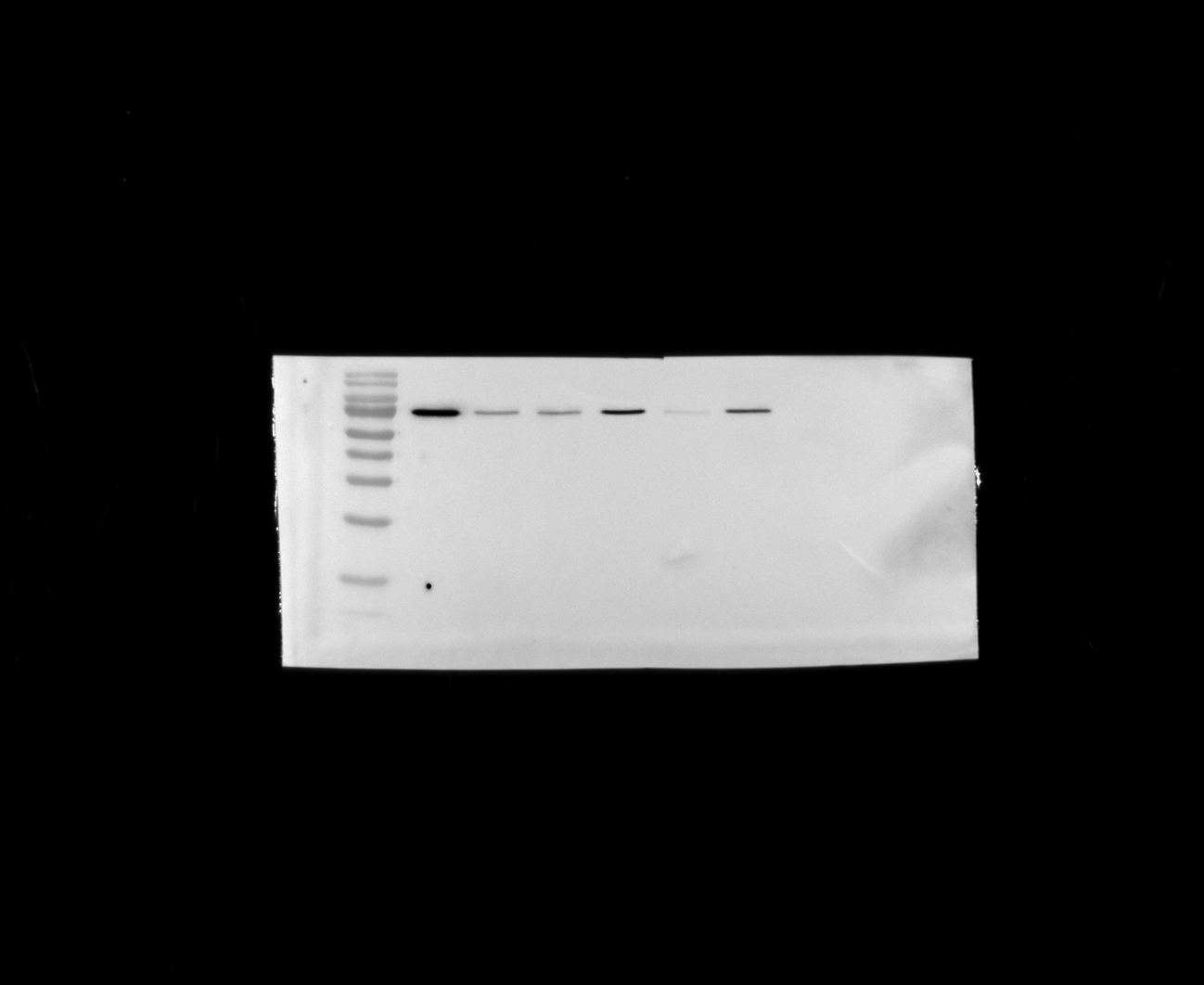

Supplement: Supplementary file 2 — Supplementary Material 2. [file 12935_2025_3665_MOESM2_ESM.zip › Supplementary Material 2/Figure 6/Figure 6K/MMP-2.tif]

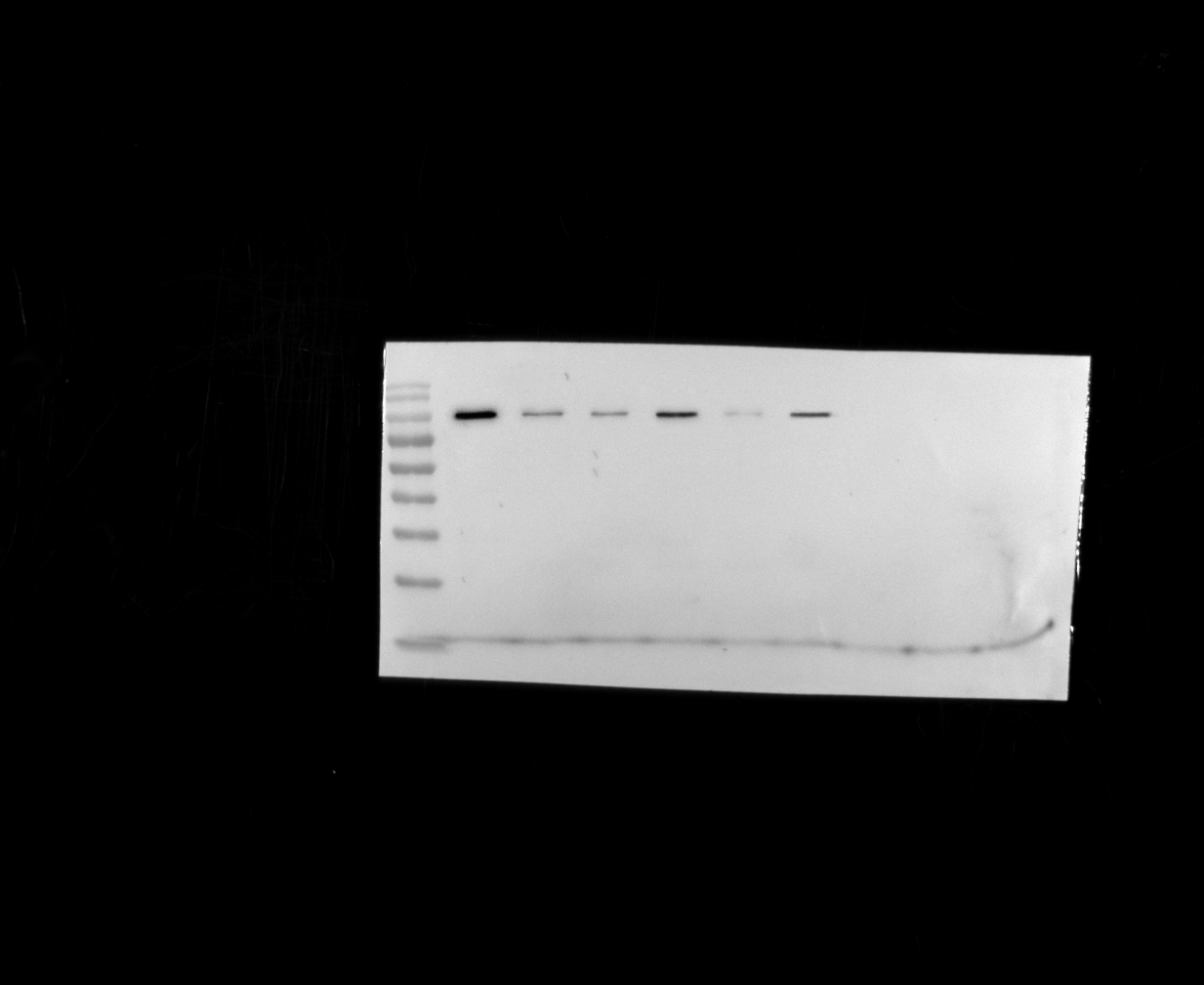

Supplement: Supplementary file 2 — Supplementary Material 2. [file 12935_2025_3665_MOESM2_ESM.zip › Supplementary Material 2/Figure 6/Figure 6K/MMP-9 .tif]

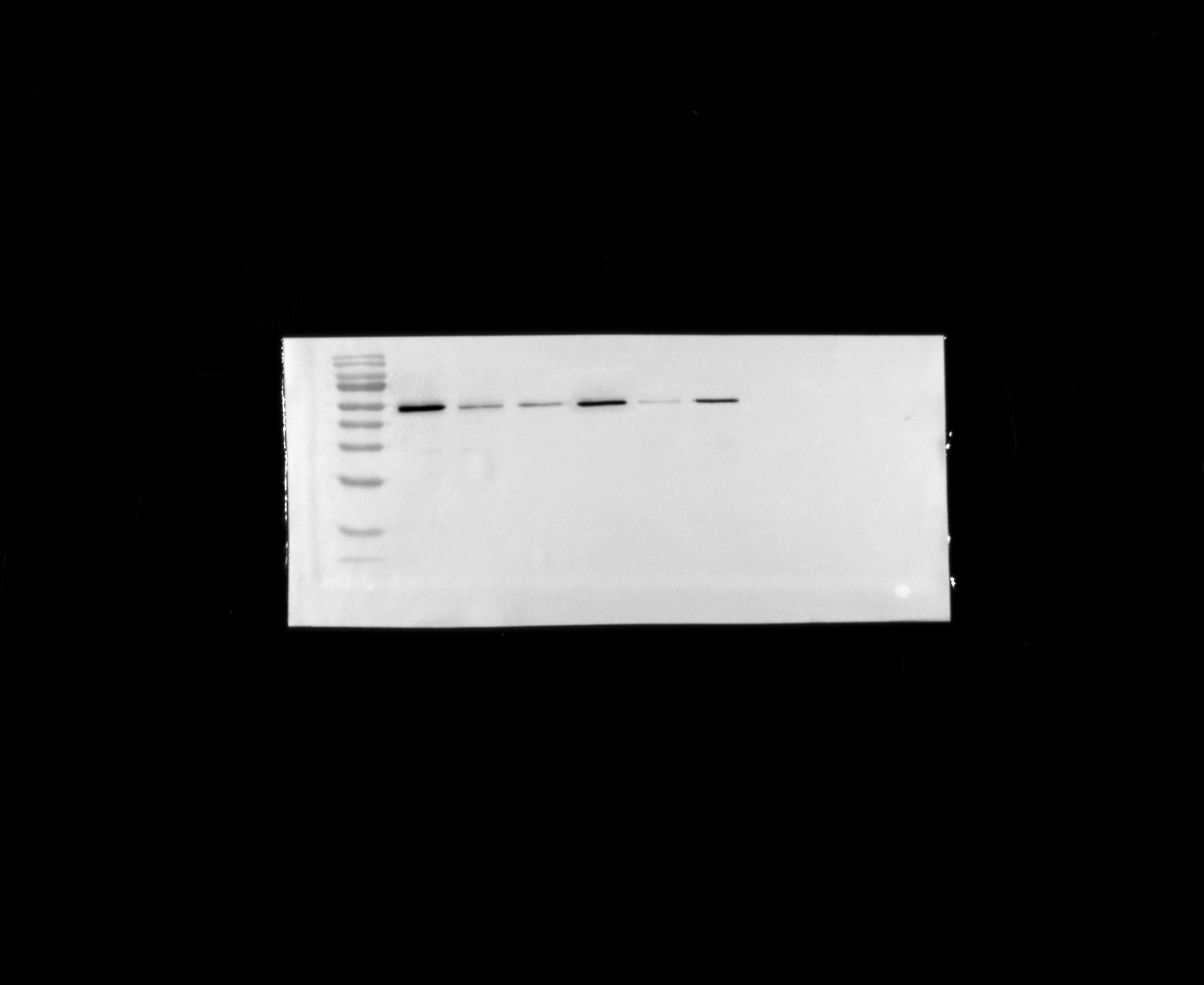

Supplement: Supplementary file 2 — Supplementary Material 2. [file 12935_2025_3665_MOESM2_ESM.zip › Supplementary Material 2/Figure 6/Figure 6K/Vimentin.tif]

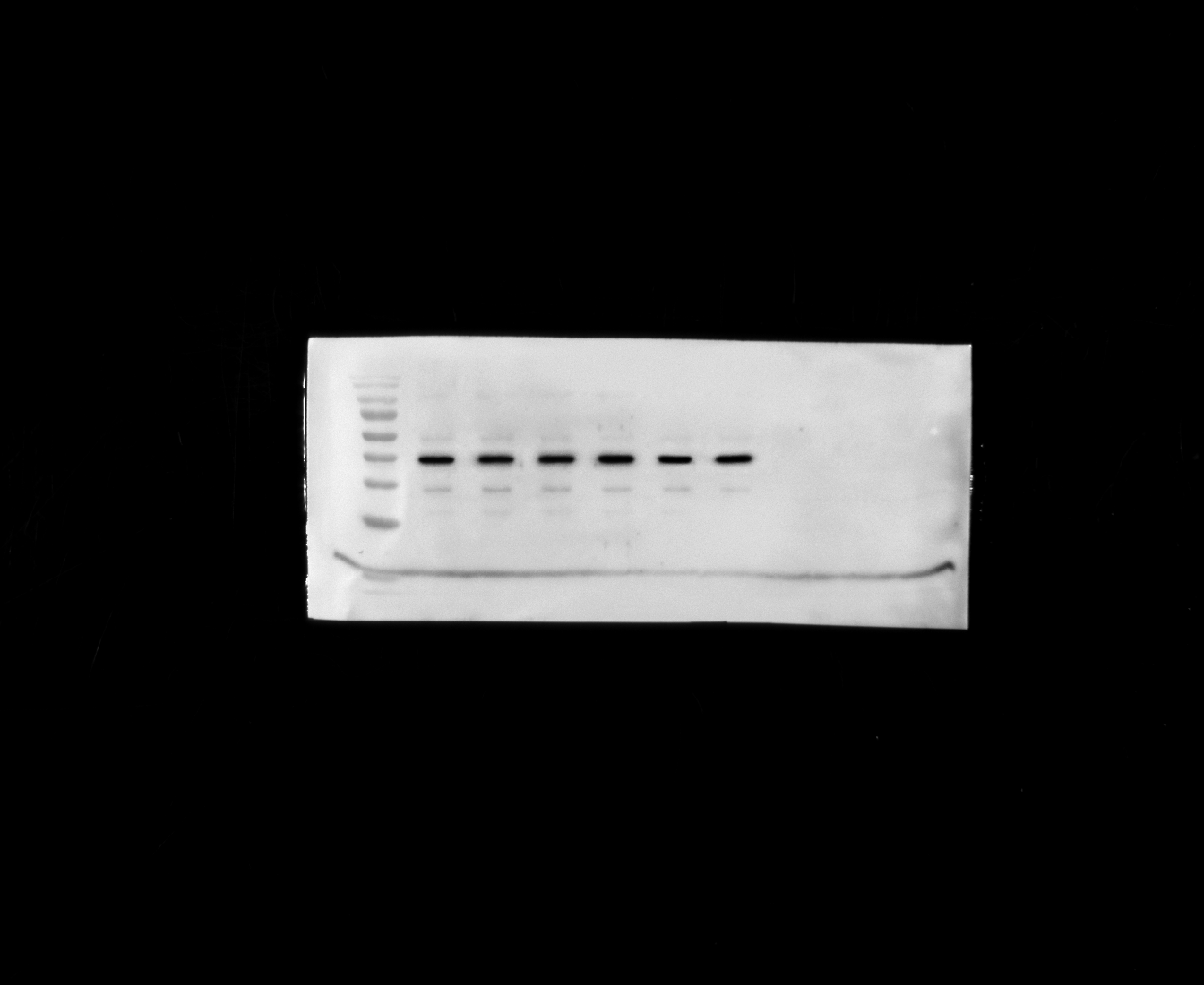

Supplement: Supplementary file 2 — Supplementary Material 2. [file 12935_2025_3665_MOESM2_ESM.zip › Supplementary Material 2/Figure 6/Figure 6K/β-actin.tif]

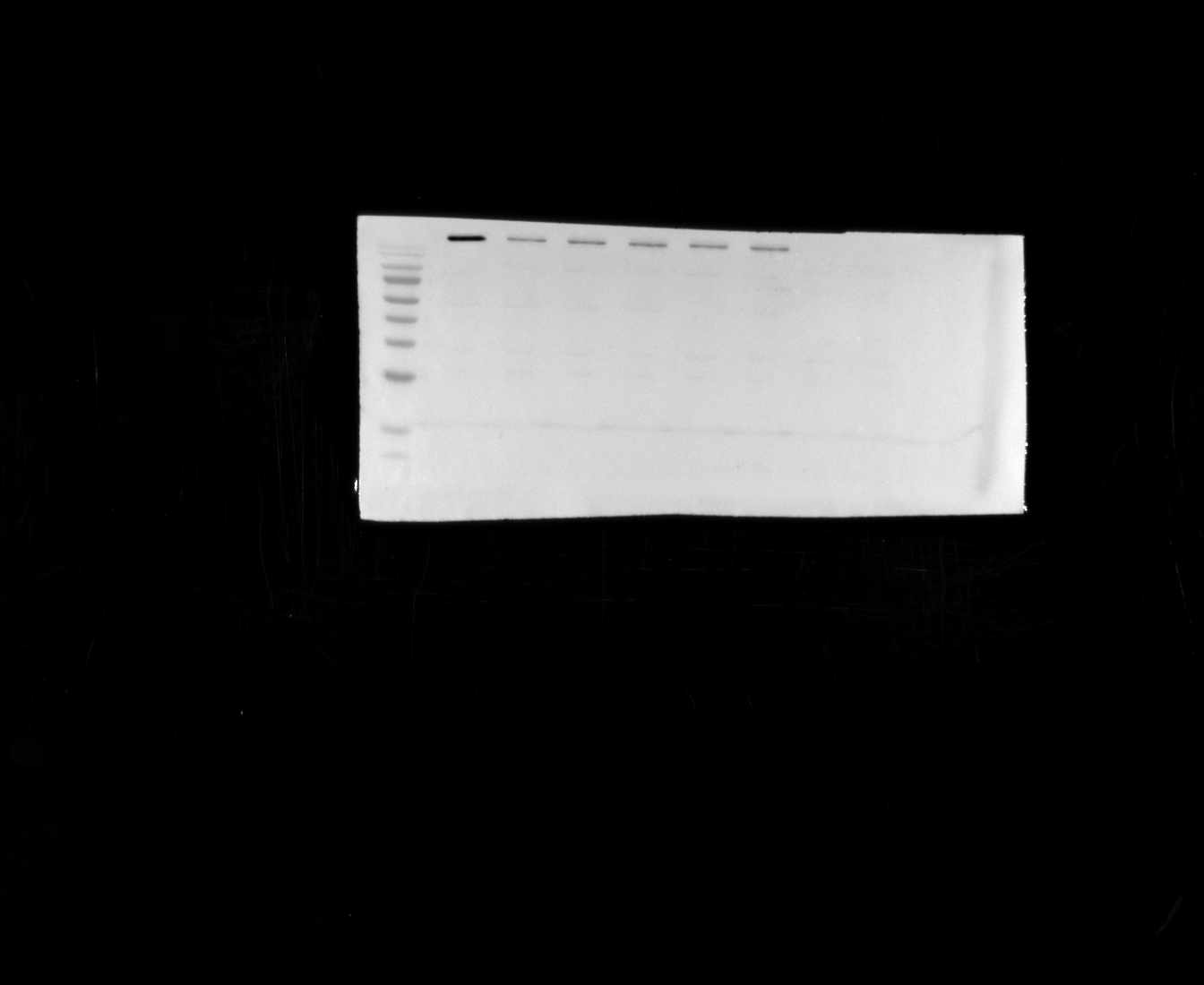

Supplement: Supplementary file 2 — Supplementary Material 2. [file 12935_2025_3665_MOESM2_ESM.zip › Supplementary Material 2/Figure 7/Figure 7C/EGFR.tif]

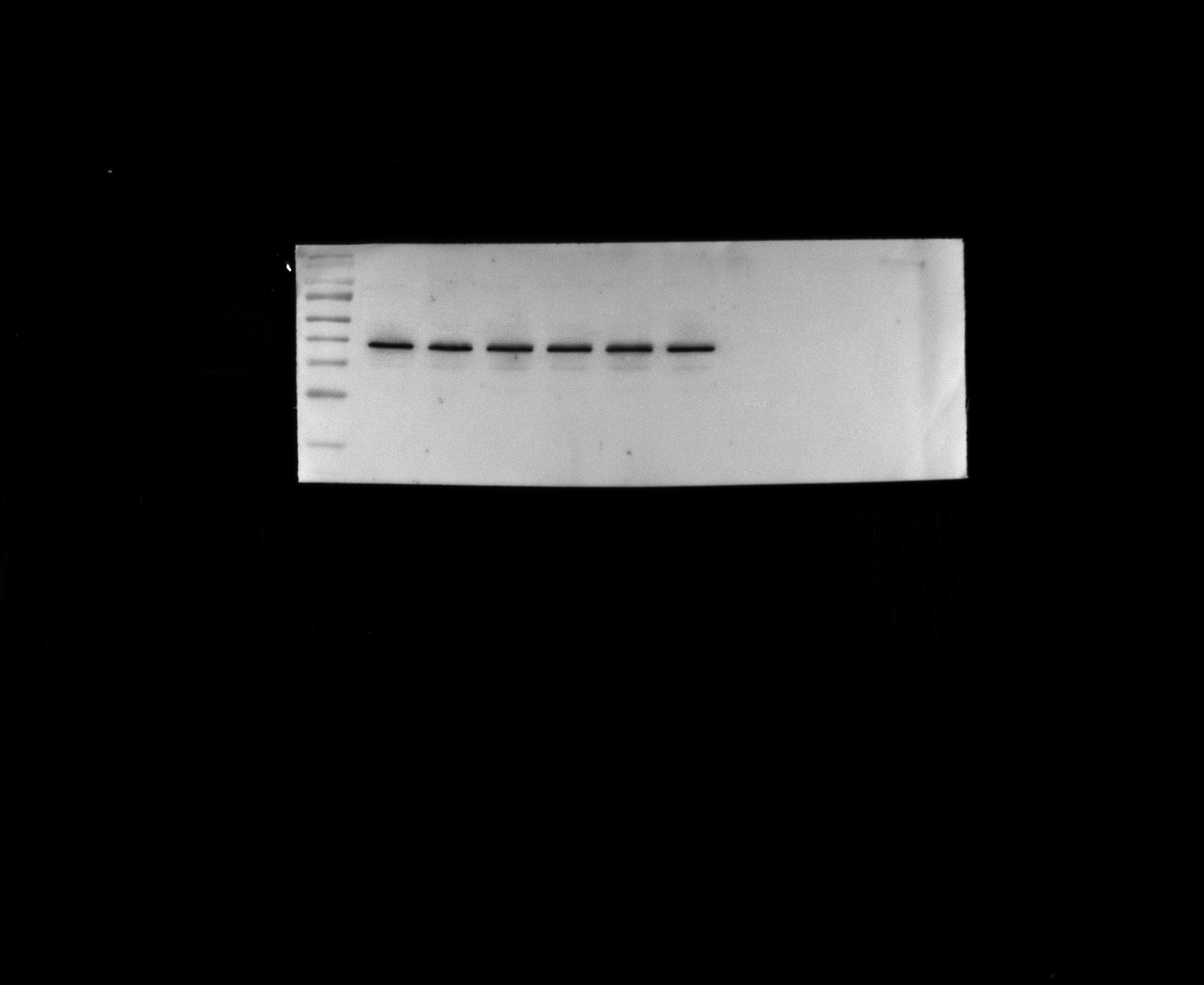

Supplement: Supplementary file 2 — Supplementary Material 2. [file 12935_2025_3665_MOESM2_ESM.zip › Supplementary Material 2/Figure 7/Figure 7C/ERK2.tif]

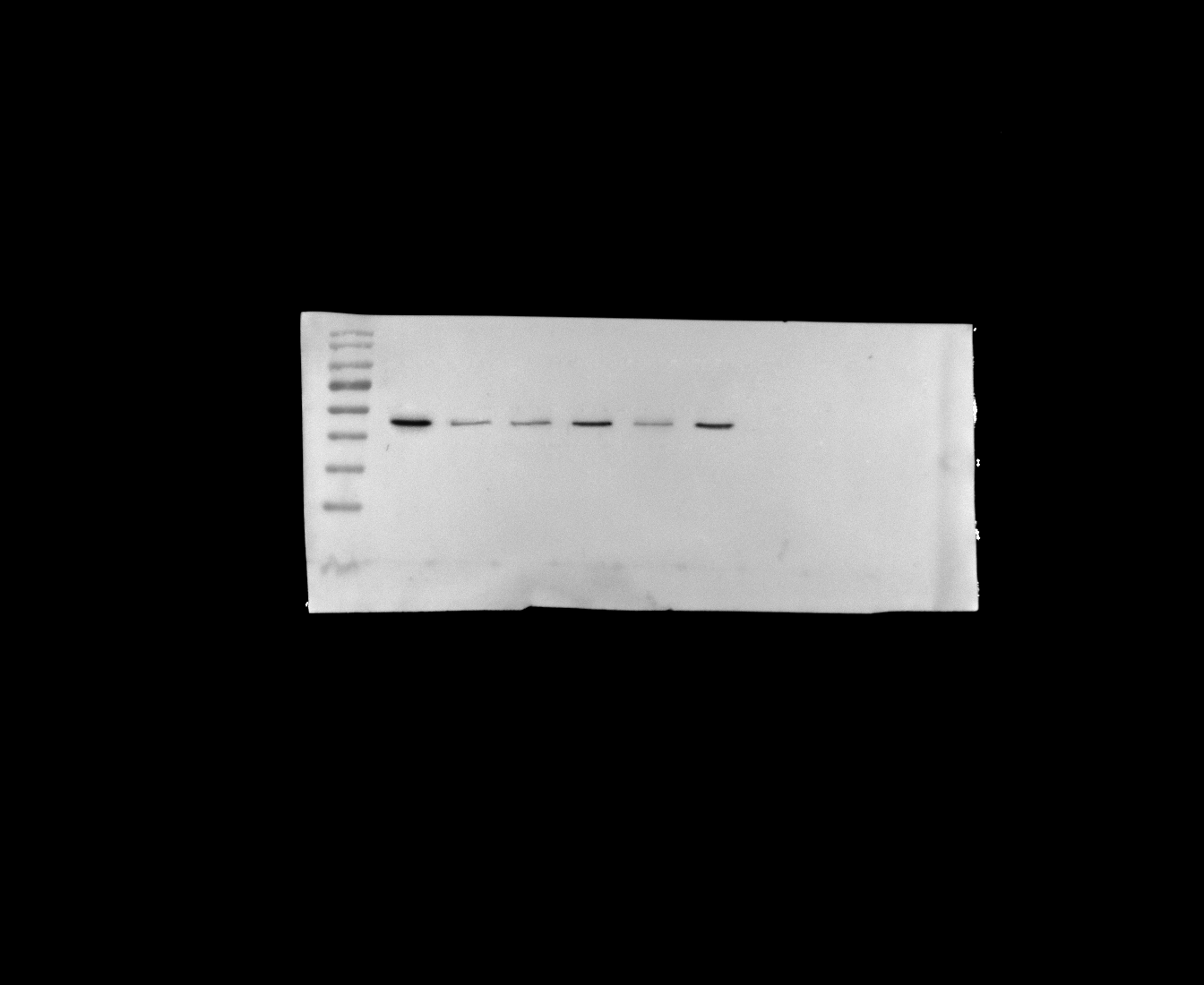

Supplement: Supplementary file 2 — Supplementary Material 2. [file 12935_2025_3665_MOESM2_ESM.zip › Supplementary Material 2/Figure 7/Figure 7C/Ets-1.tif]

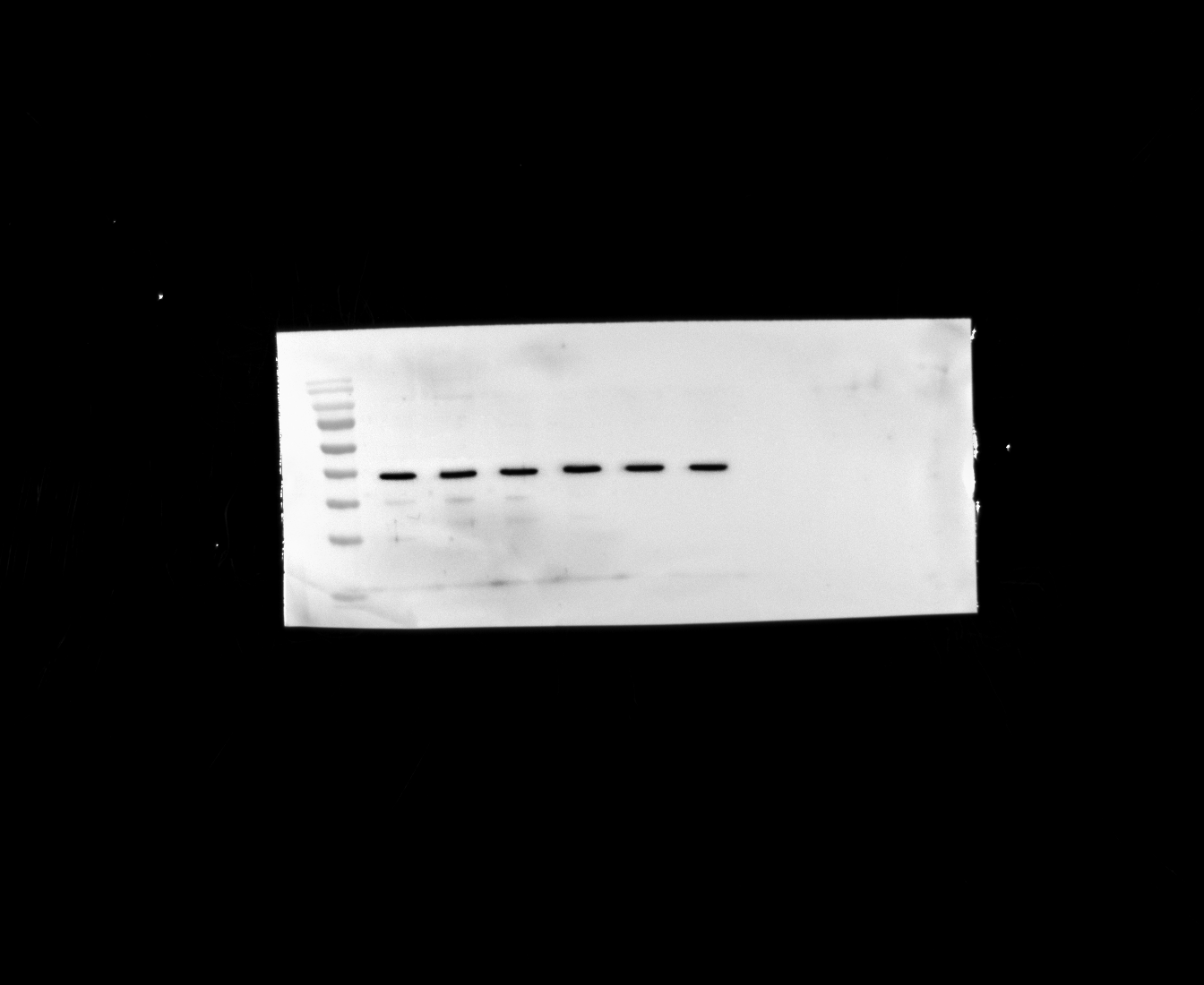

Supplement: Supplementary file 2 — Supplementary Material 2. [file 12935_2025_3665_MOESM2_ESM.zip › Supplementary Material 2/Figure 7/Figure 7C/GAPDH.tif]

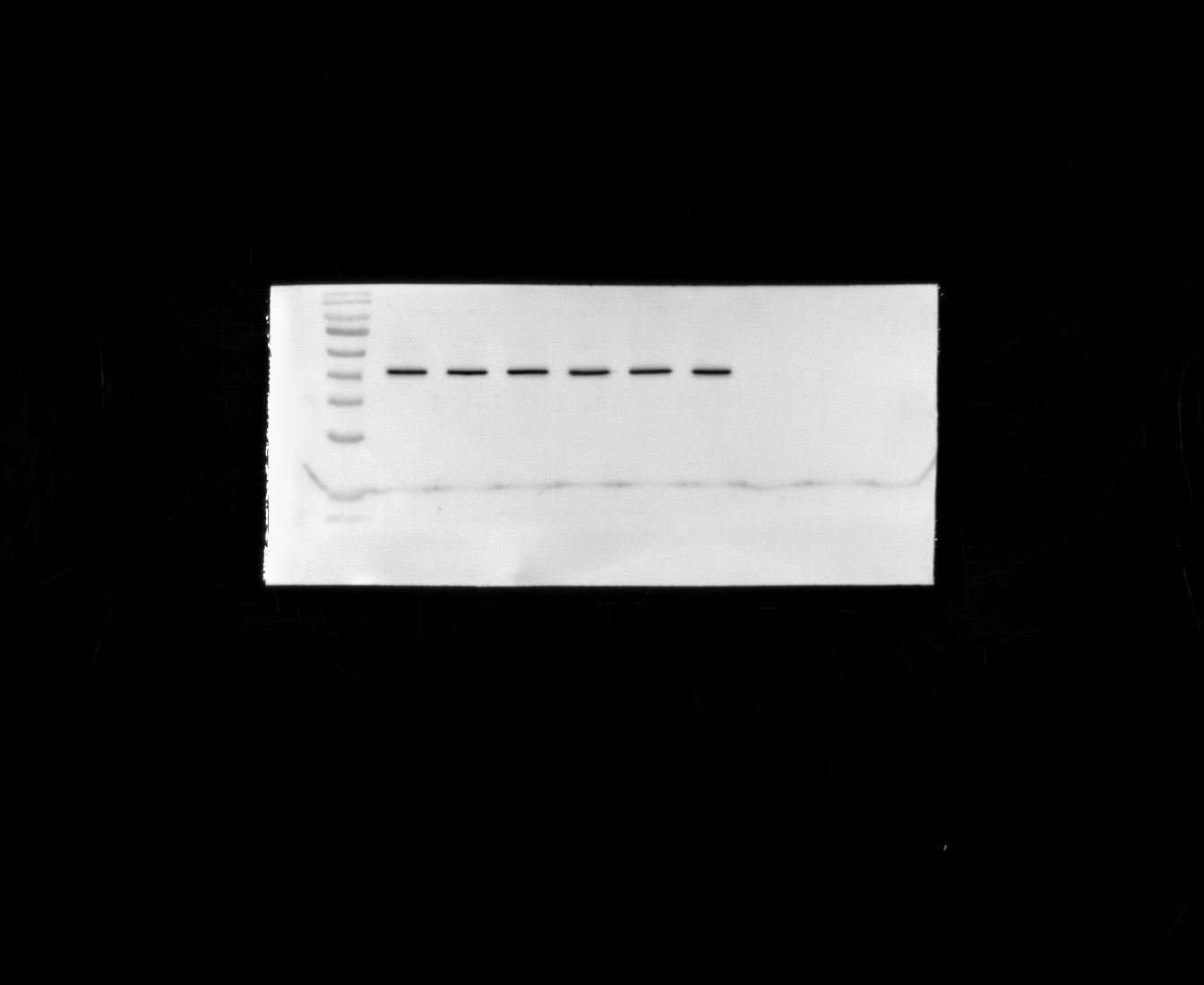

Supplement: Supplementary file 2 — Supplementary Material 2. [file 12935_2025_3665_MOESM2_ESM.zip › Supplementary Material 2/Figure 7/Figure 7C/MEK.tif]

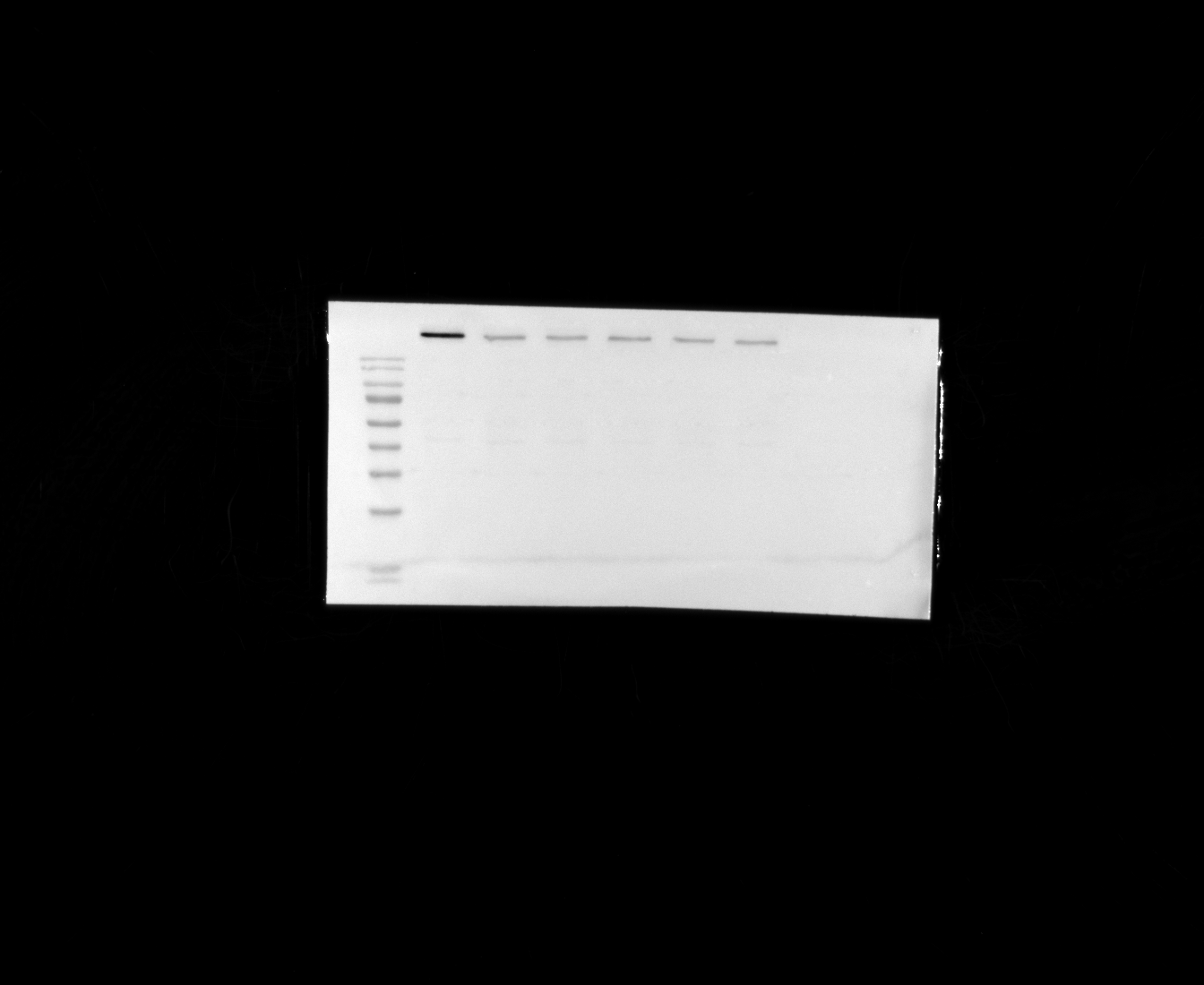

Supplement: Supplementary file 2 — Supplementary Material 2. [file 12935_2025_3665_MOESM2_ESM.zip › Supplementary Material 2/Figure 7/Figure 7C/P-EGFR(Y1069).tif]

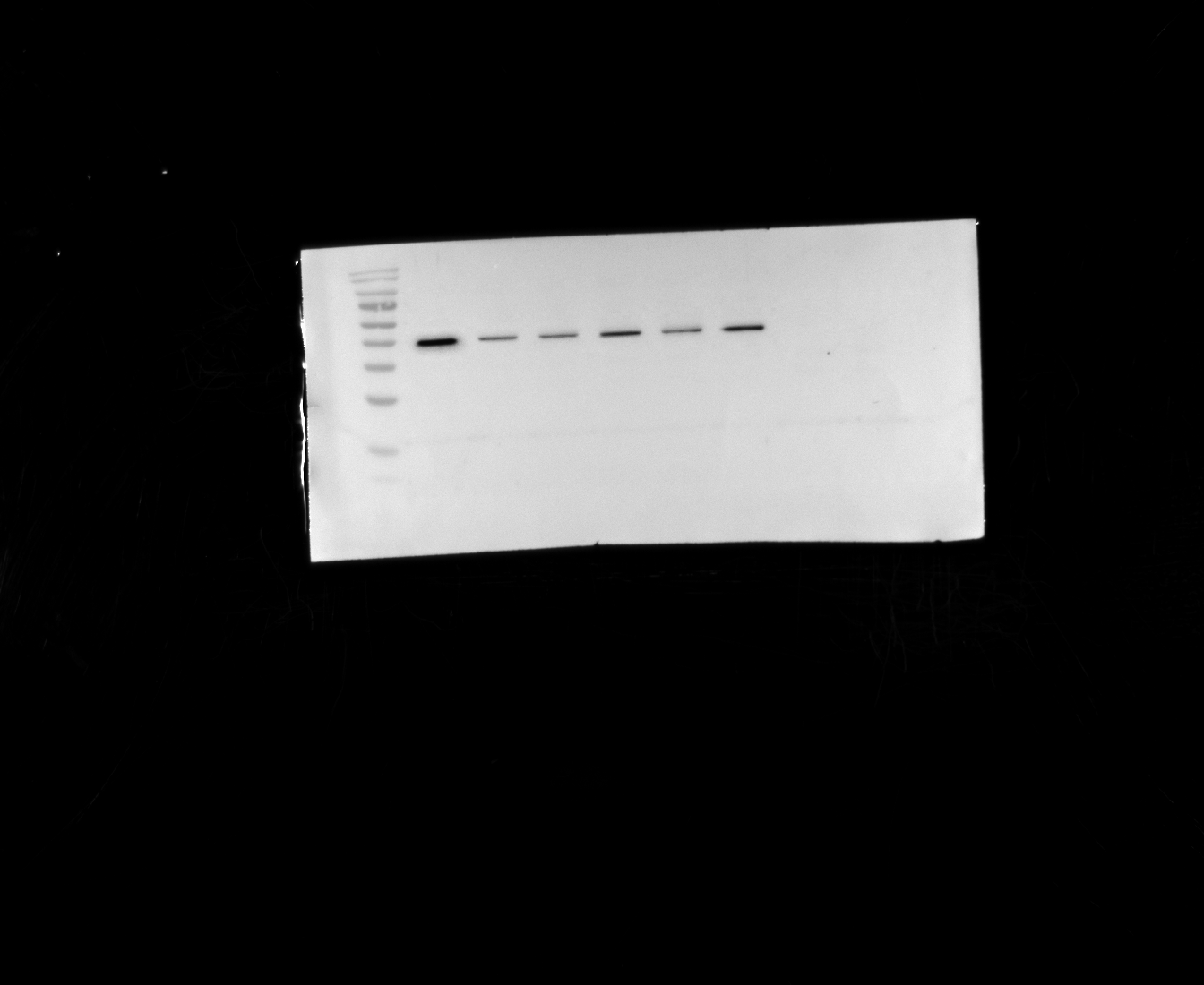

Supplement: Supplementary file 2 — Supplementary Material 2. [file 12935_2025_3665_MOESM2_ESM.zip › Supplementary Material 2/Figure 7/Figure 7C/p-ERK2(T185).tif]

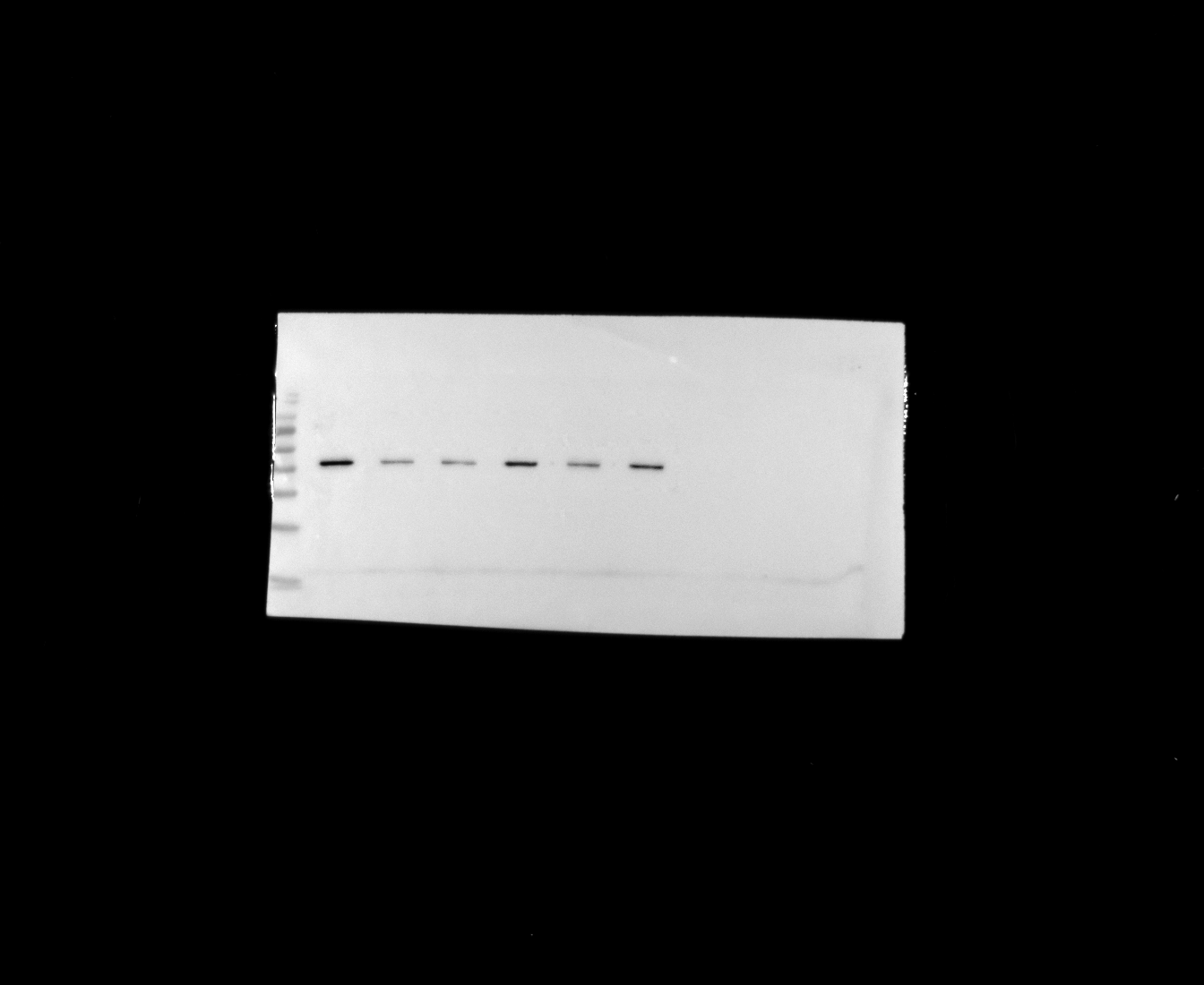

Supplement: Supplementary file 2 — Supplementary Material 2. [file 12935_2025_3665_MOESM2_ESM.zip › Supplementary Material 2/Figure 7/Figure 7C/p-MEK.tif]

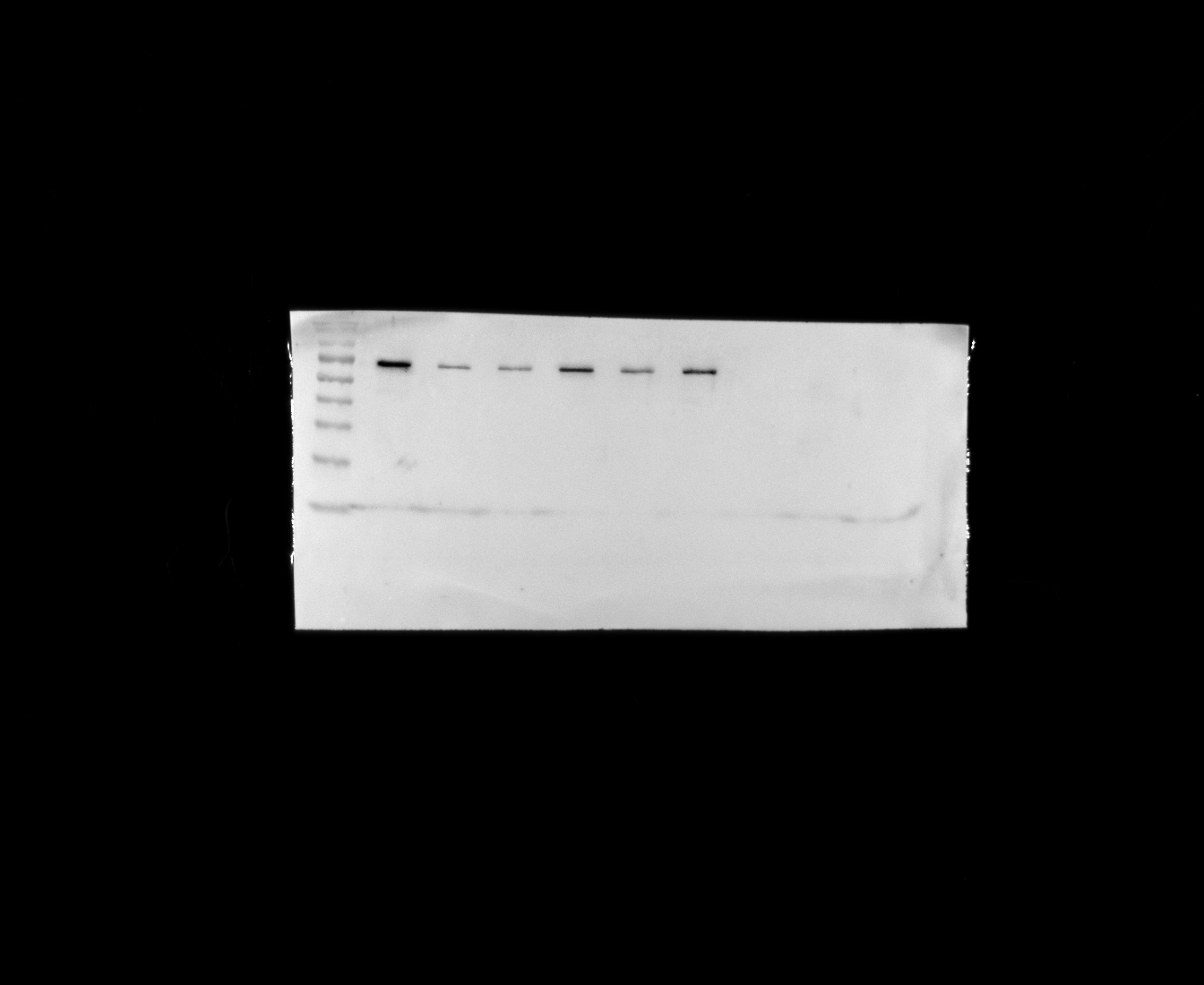

Supplement: Supplementary file 2 — Supplementary Material 2. [file 12935_2025_3665_MOESM2_ESM.zip › Supplementary Material 2/Figure 7/Figure 7C/RAF.tif]

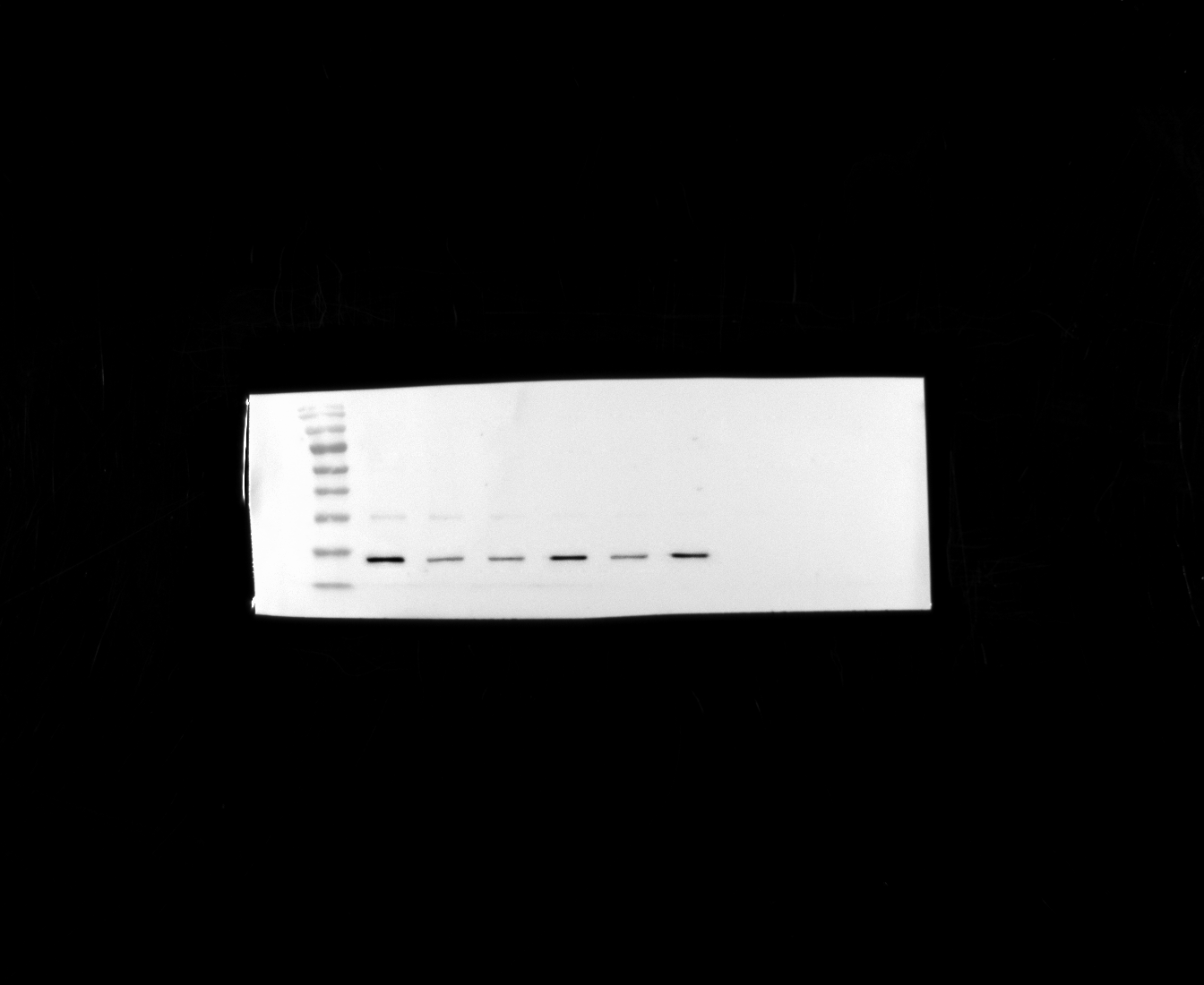

Supplement: Supplementary file 2 — Supplementary Material 2. [file 12935_2025_3665_MOESM2_ESM.zip › Supplementary Material 2/Figure 7/Figure 7C/RAS.tif]

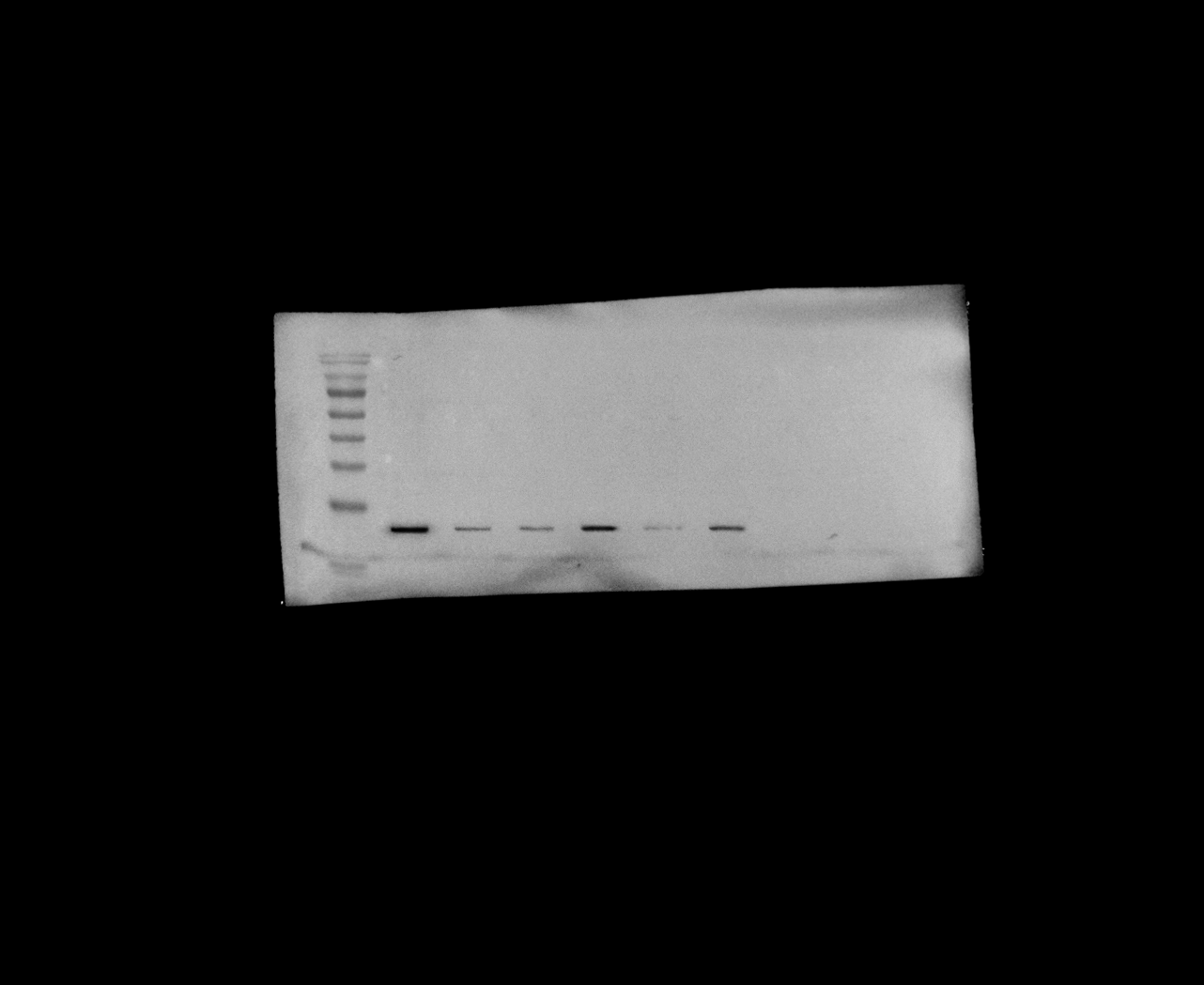

Supplement: Supplementary file 2 — Supplementary Material 2. [file 12935_2025_3665_MOESM2_ESM.zip › Supplementary Material 2/Figure 7/Figure 7D/GPX4.tif]

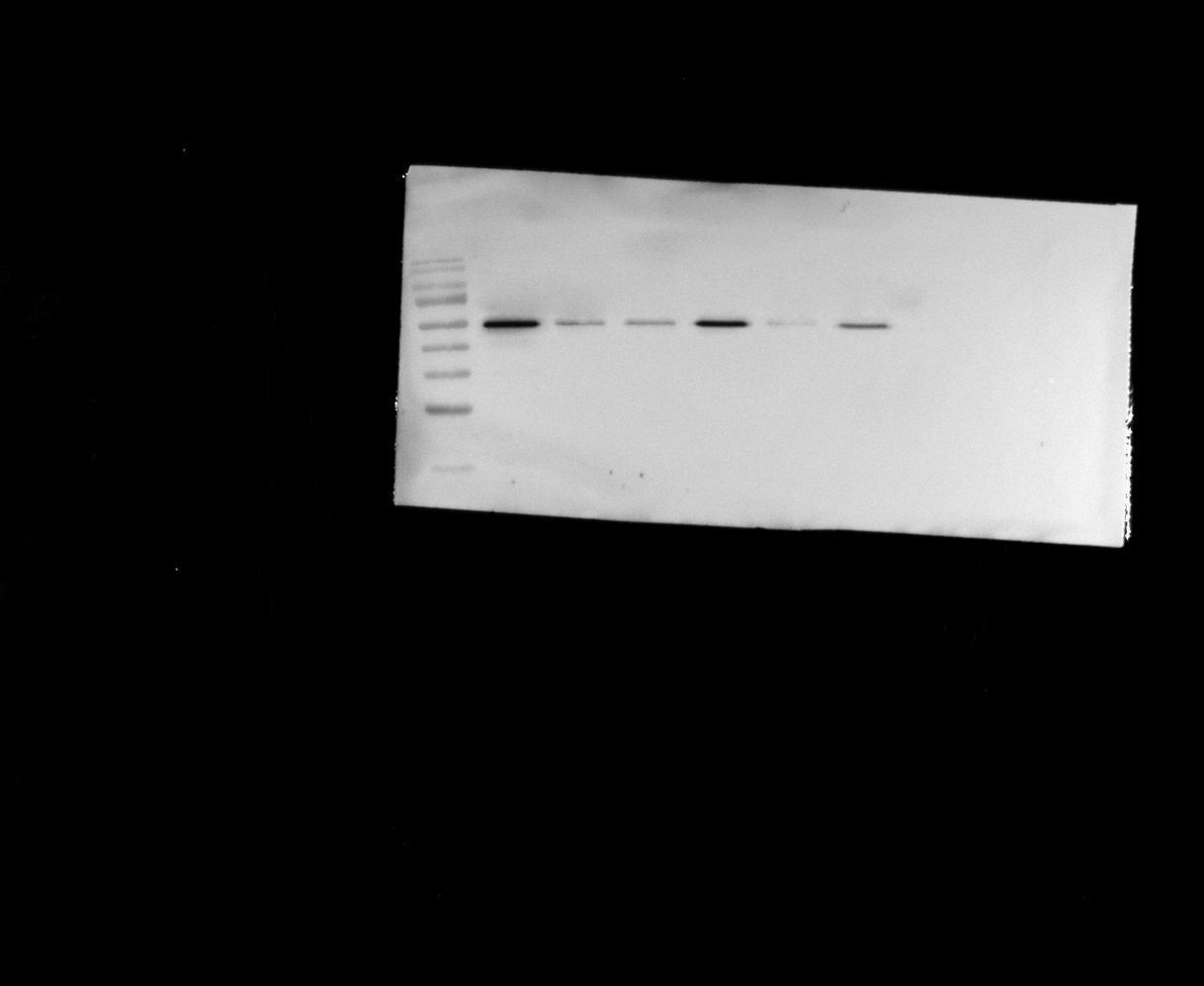

Supplement: Supplementary file 2 — Supplementary Material 2. [file 12935_2025_3665_MOESM2_ESM.zip › Supplementary Material 2/Figure 7/Figure 7D/SLC7A11.tif]

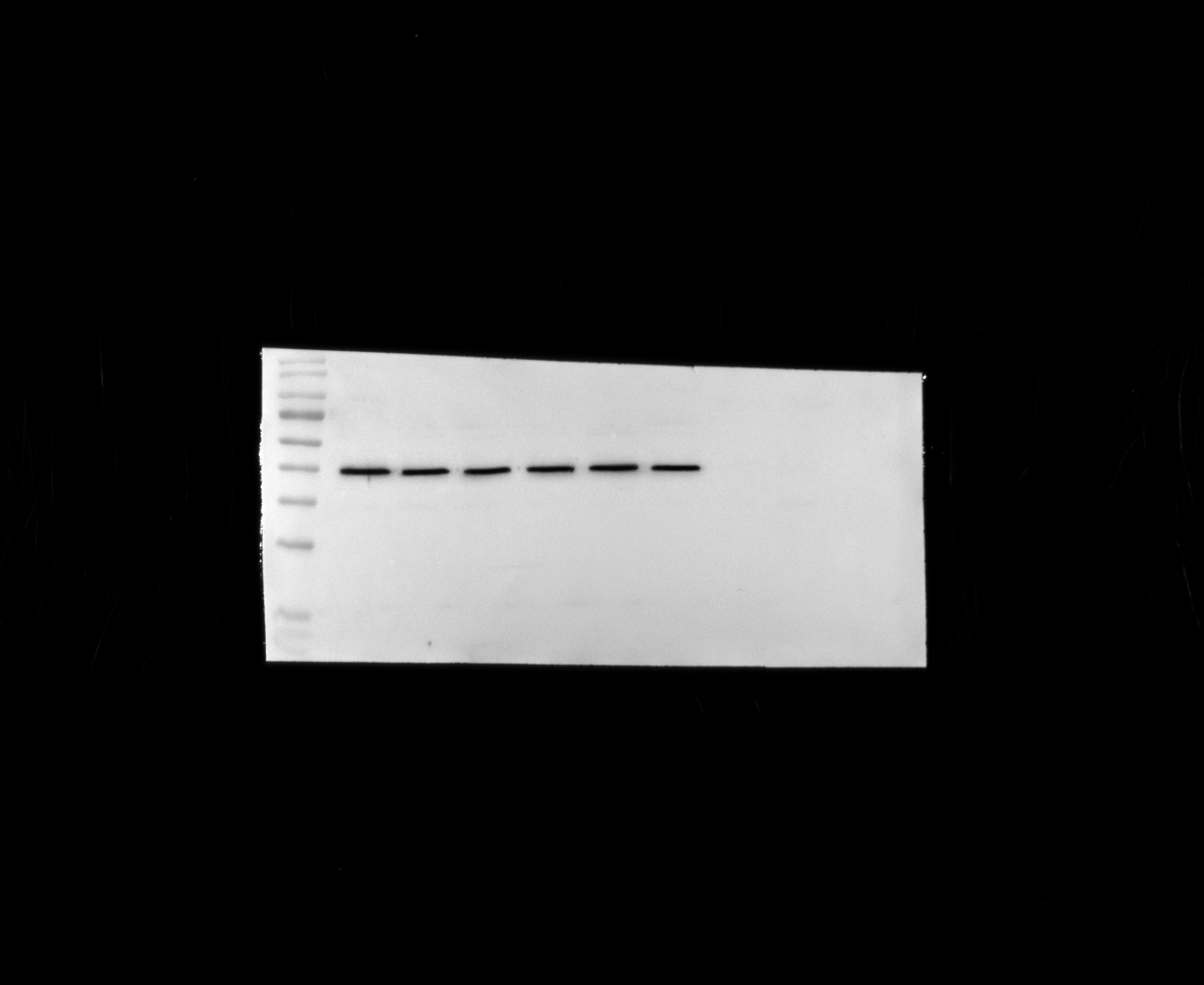

Supplement: Supplementary file 2 — Supplementary Material 2. [file 12935_2025_3665_MOESM2_ESM.zip › Supplementary Material 2/Figure 7/Figure 7D/β-actin.tif]

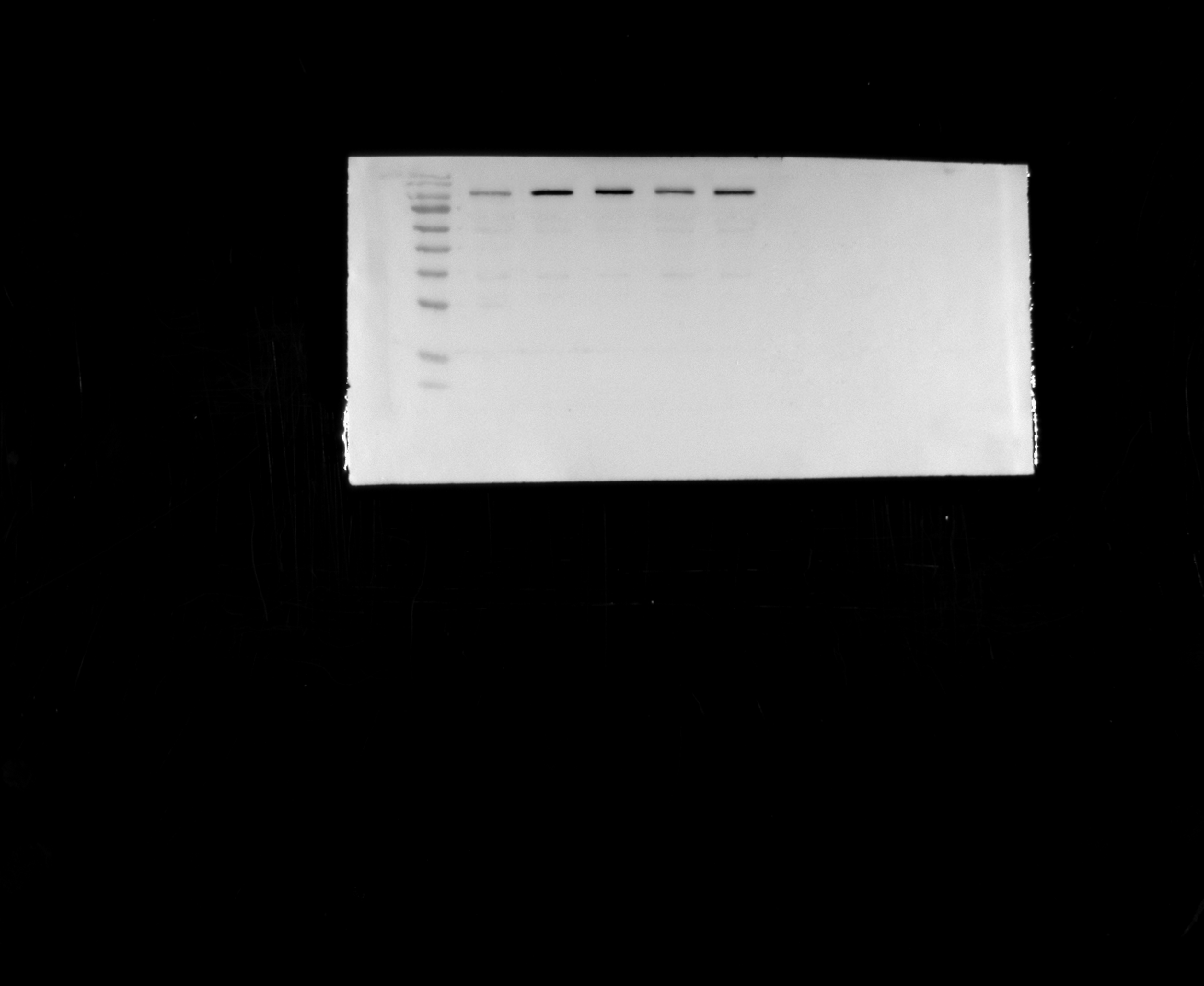

Supplement: Supplementary file 2 — Supplementary Material 2. [file 12935_2025_3665_MOESM2_ESM.zip › Supplementary Material 2/Figure S1/Figure S1I/E-cadherin.tif]

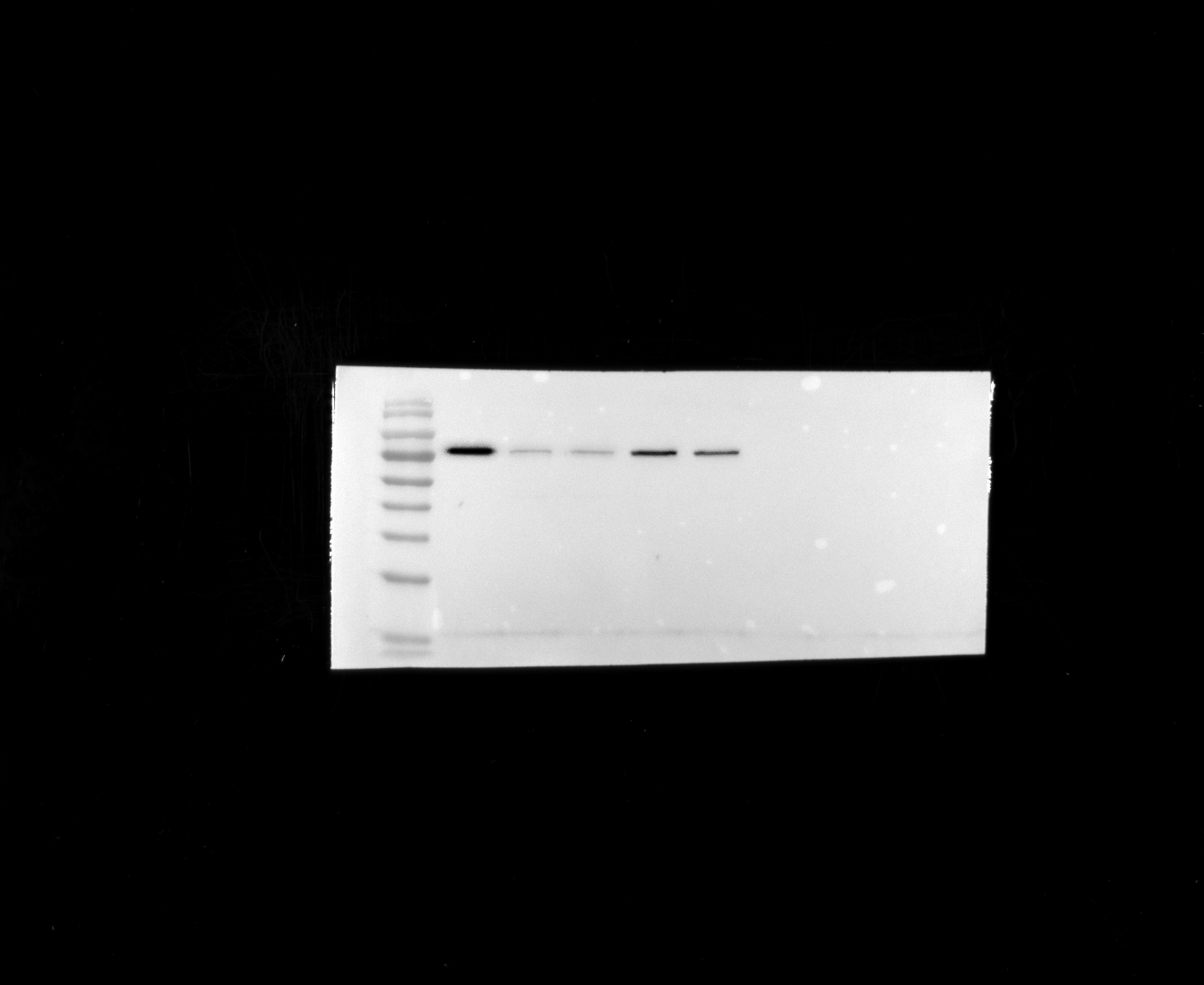

Supplement: Supplementary file 2 — Supplementary Material 2. [file 12935_2025_3665_MOESM2_ESM.zip › Supplementary Material 2/Figure S1/Figure S1I/MMP-2.tif]

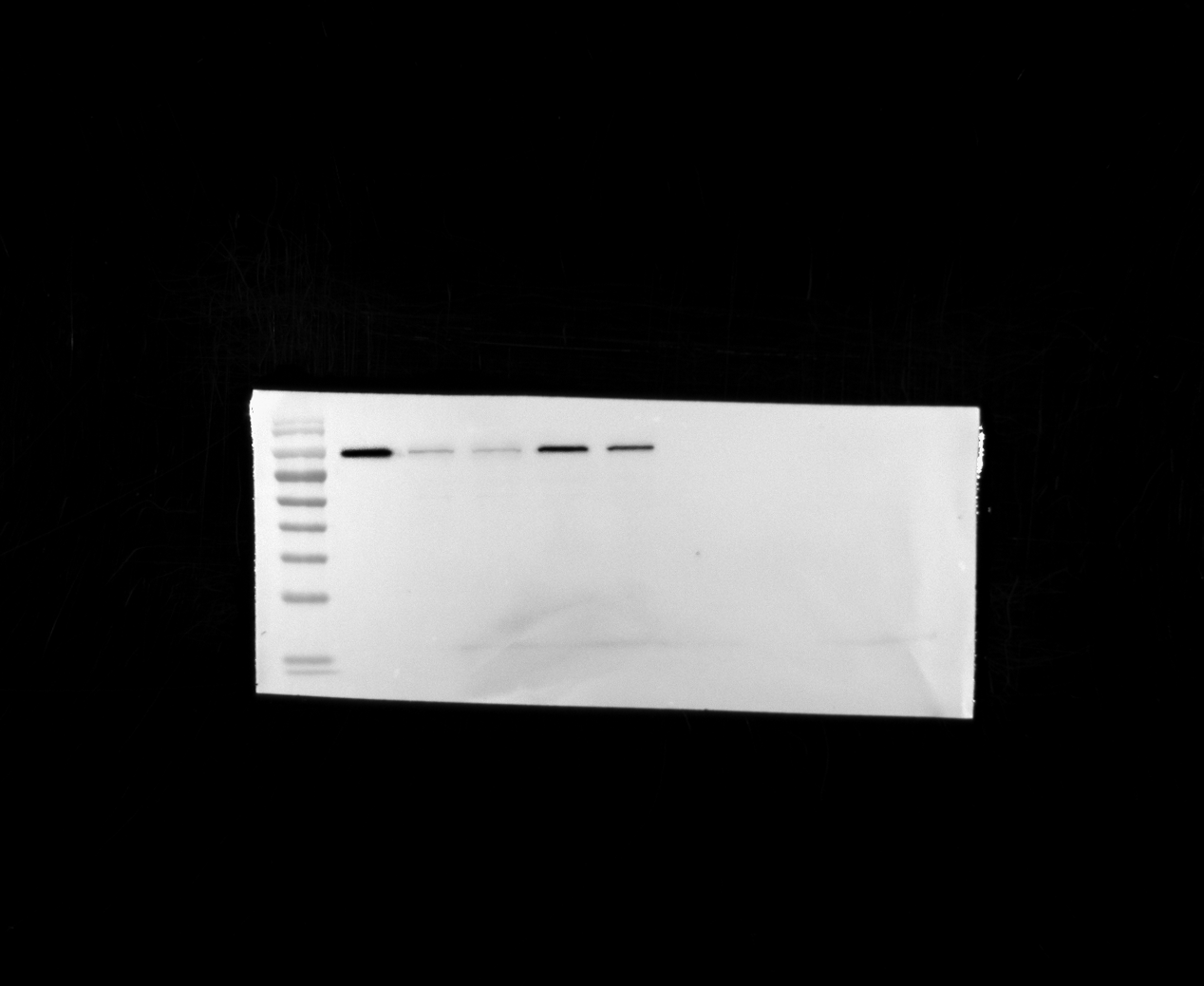

Supplement: Supplementary file 2 — Supplementary Material 2. [file 12935_2025_3665_MOESM2_ESM.zip › Supplementary Material 2/Figure S1/Figure S1I/MMP-9.tif]

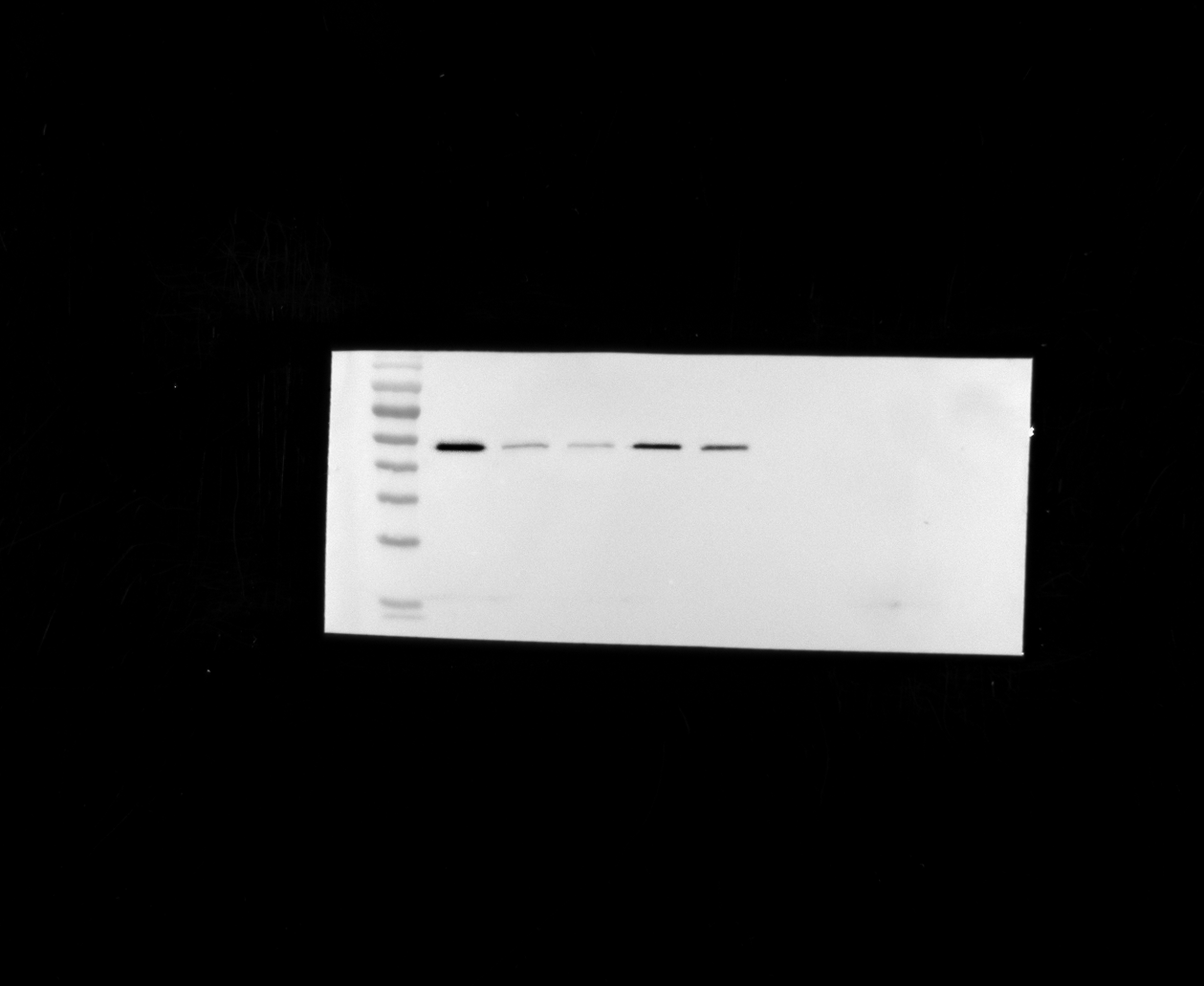

Supplement: Supplementary file 2 — Supplementary Material 2. [file 12935_2025_3665_MOESM2_ESM.zip › Supplementary Material 2/Figure S1/Figure S1I/Vimentin.tif]

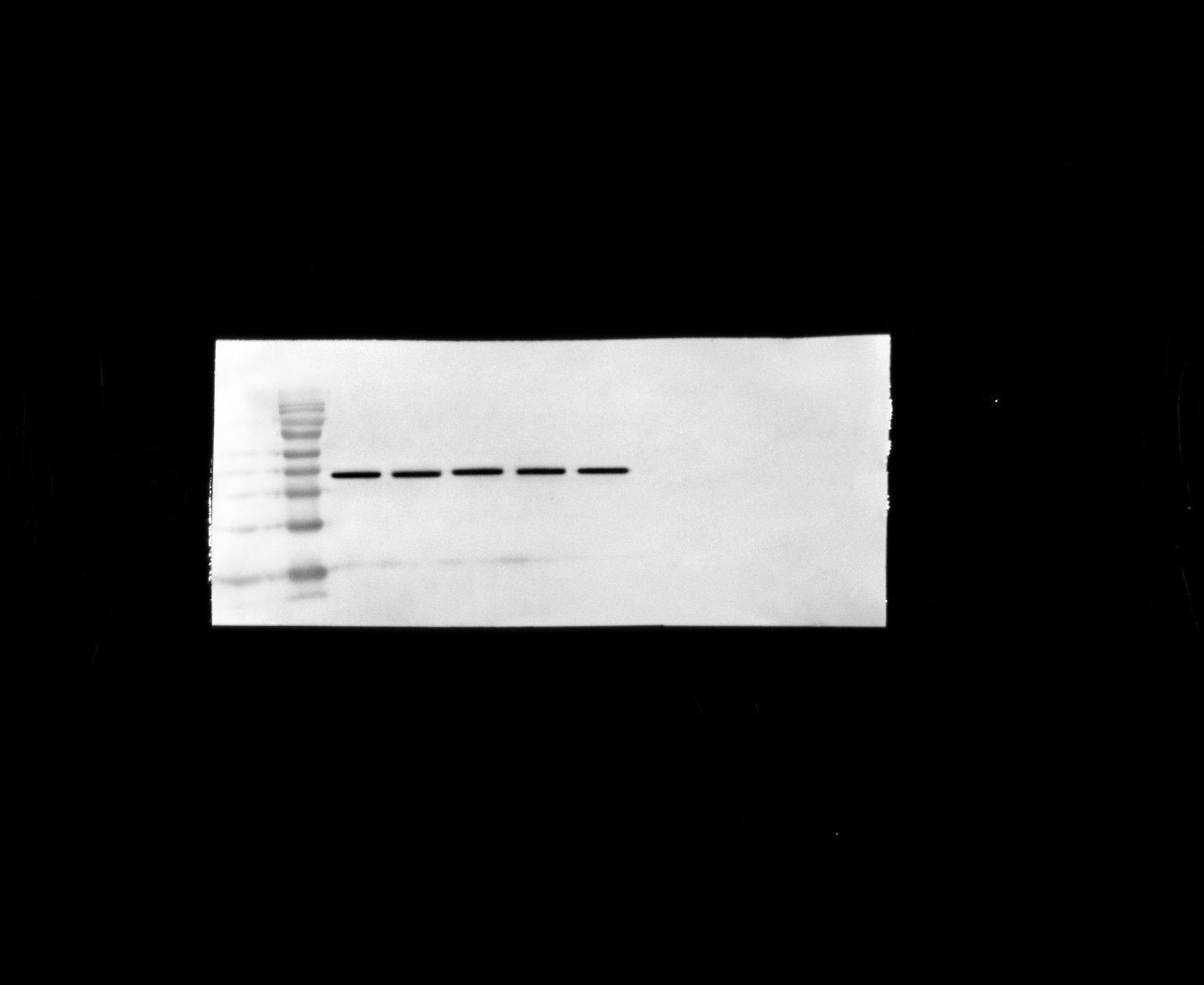

Supplement: Supplementary file 2 — Supplementary Material 2. [file 12935_2025_3665_MOESM2_ESM.zip › Supplementary Material 2/Figure S1/Figure S1I/β-actin.tif]

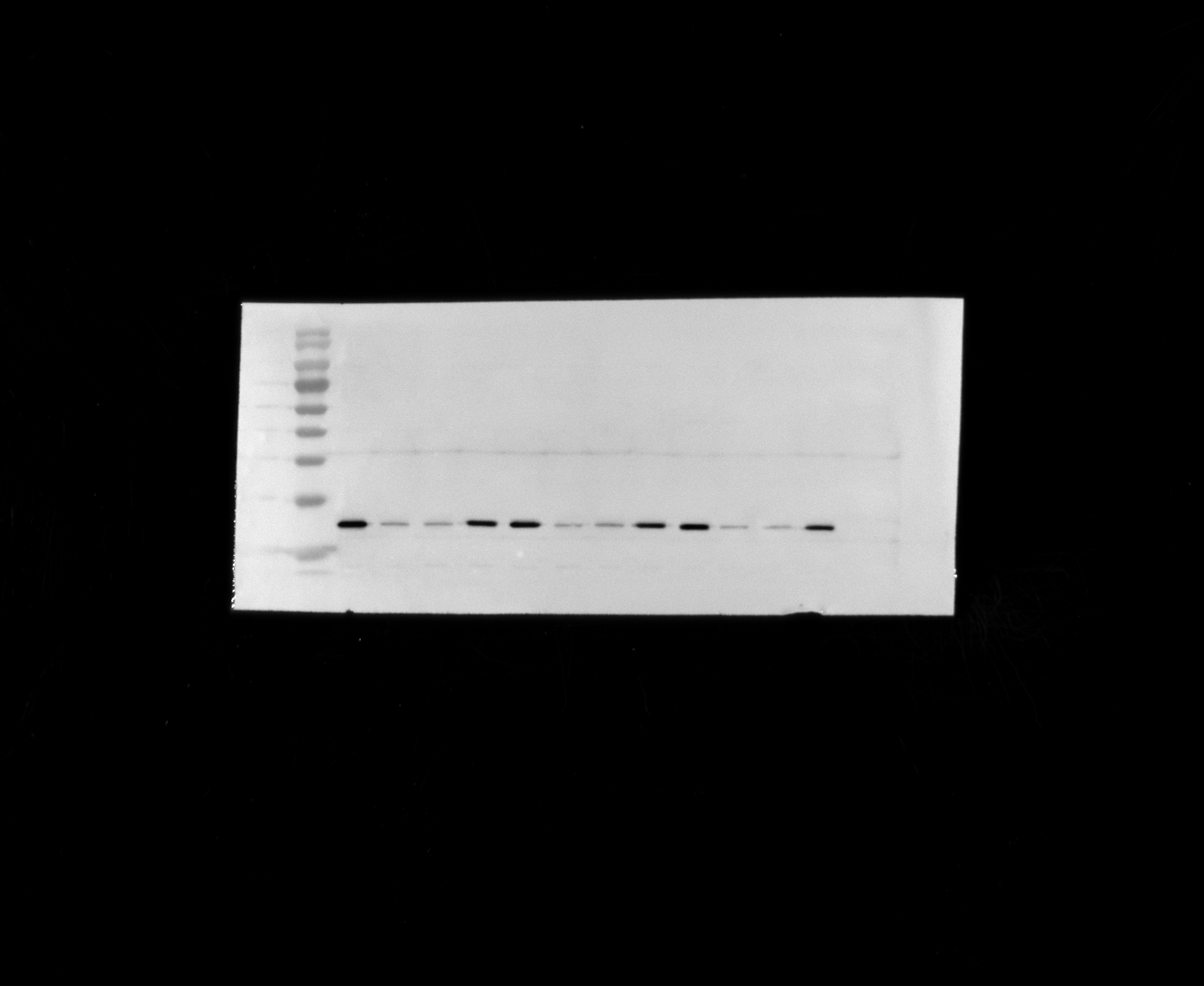

Supplement: Supplementary file 2 — Supplementary Material 2. [file 12935_2025_3665_MOESM2_ESM.zip › Supplementary Material 2/Figure S2/Figure S2A/MCF-7 GPX4.tif]

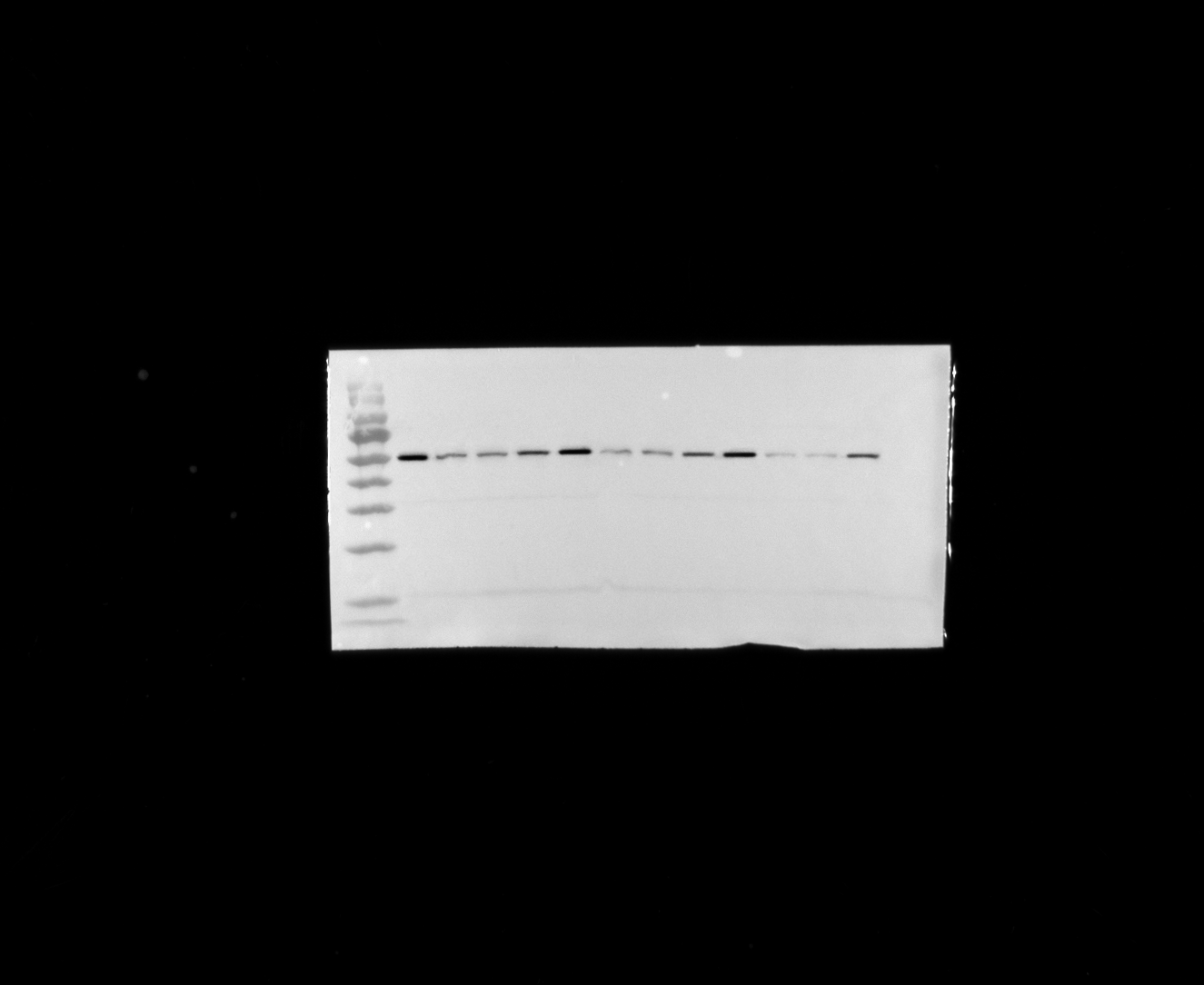

Supplement: Supplementary file 2 — Supplementary Material 2. [file 12935_2025_3665_MOESM2_ESM.zip › Supplementary Material 2/Figure S2/Figure S2A/MCF-7 SLC7A11.tif]

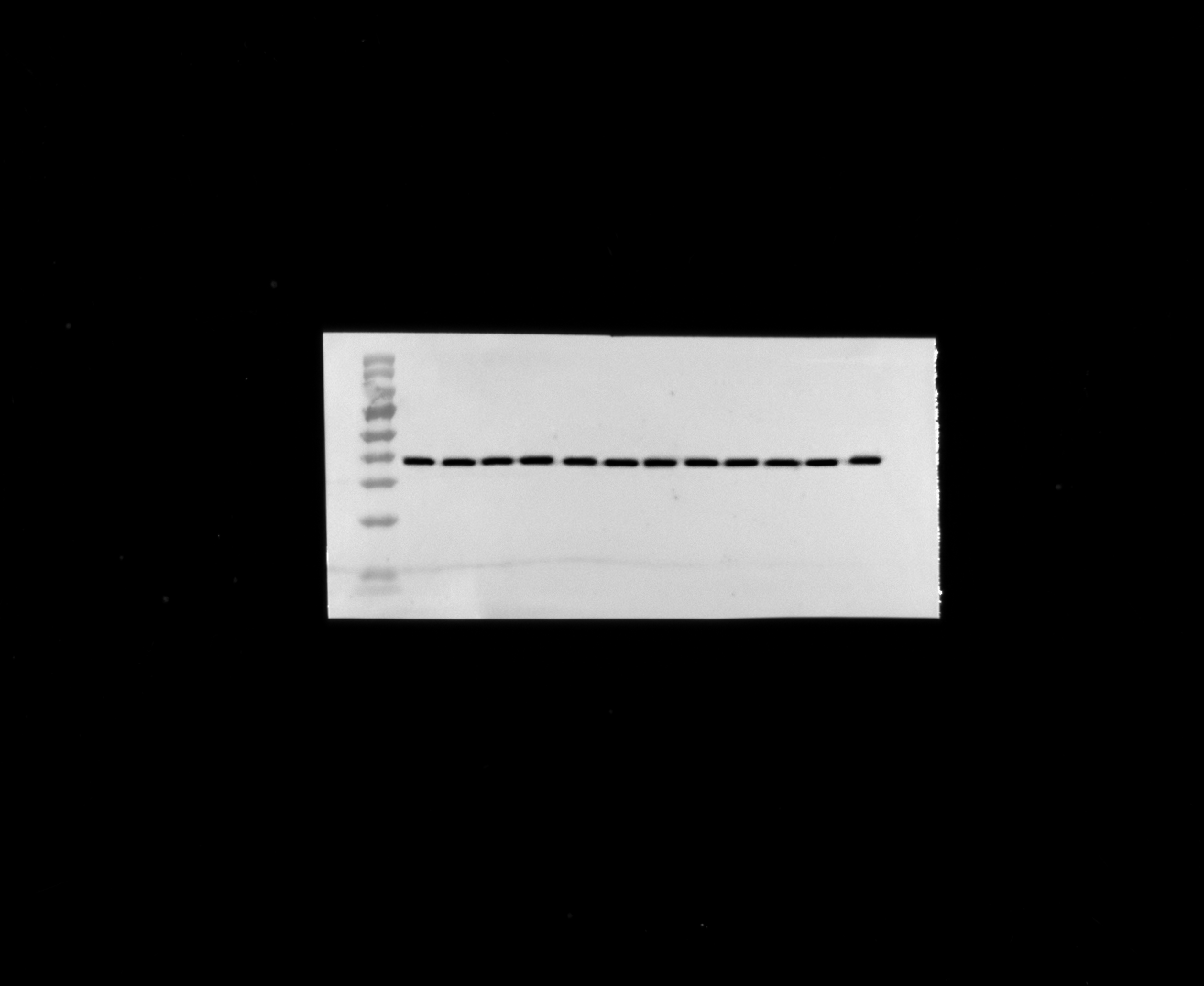

Supplement: Supplementary file 2 — Supplementary Material 2. [file 12935_2025_3665_MOESM2_ESM.zip › Supplementary Material 2/Figure S2/Figure S2A/MCF-7 β-actin.tif]

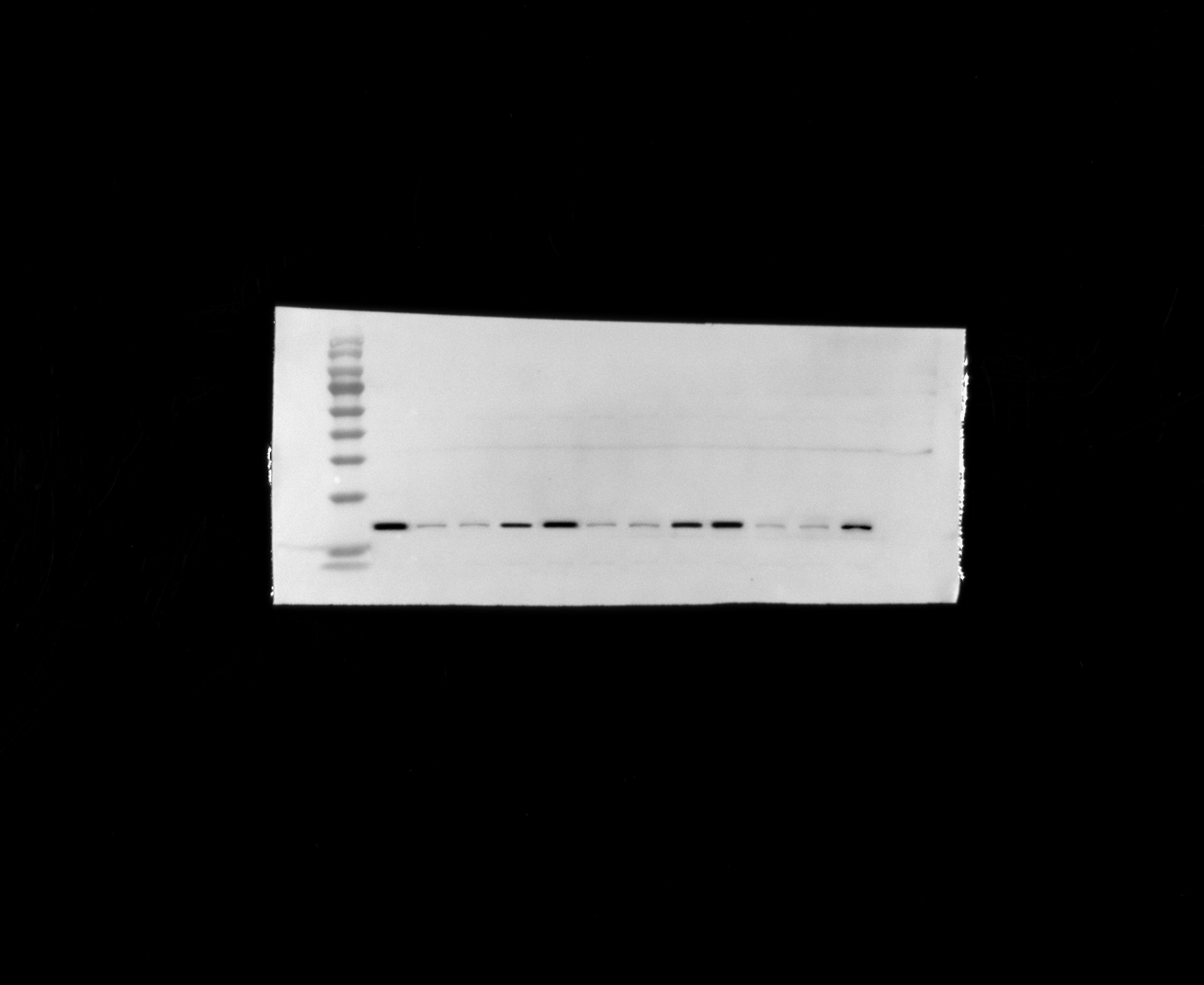

Supplement: Supplementary file 2 — Supplementary Material 2. [file 12935_2025_3665_MOESM2_ESM.zip › Supplementary Material 2/Figure S2/Figure S2A/MDA-MB-231 GPX4.tif]

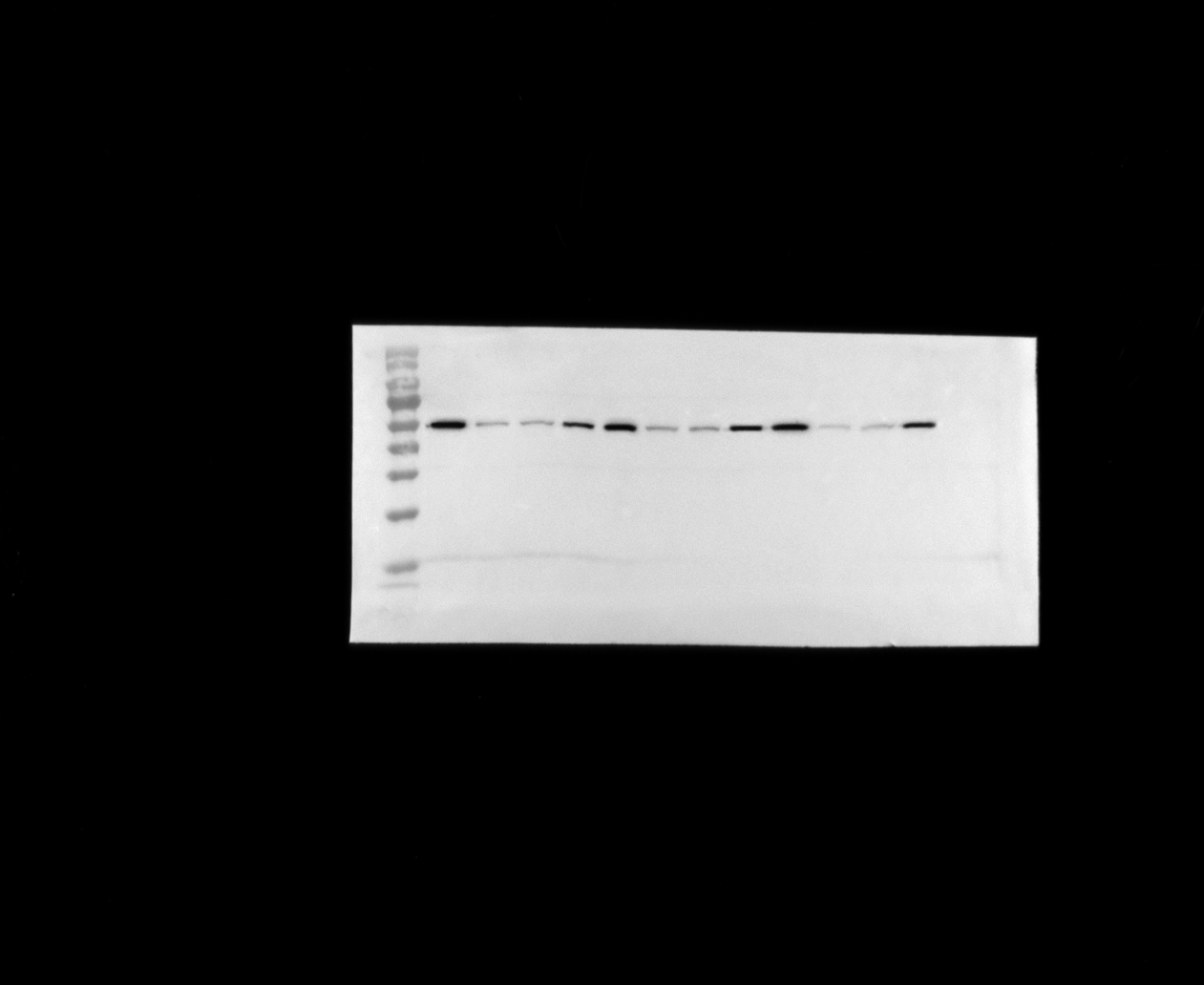

Supplement: Supplementary file 2 — Supplementary Material 2. [file 12935_2025_3665_MOESM2_ESM.zip › Supplementary Material 2/Figure S2/Figure S2A/MDA-MB-231 SLC7A11.tif]

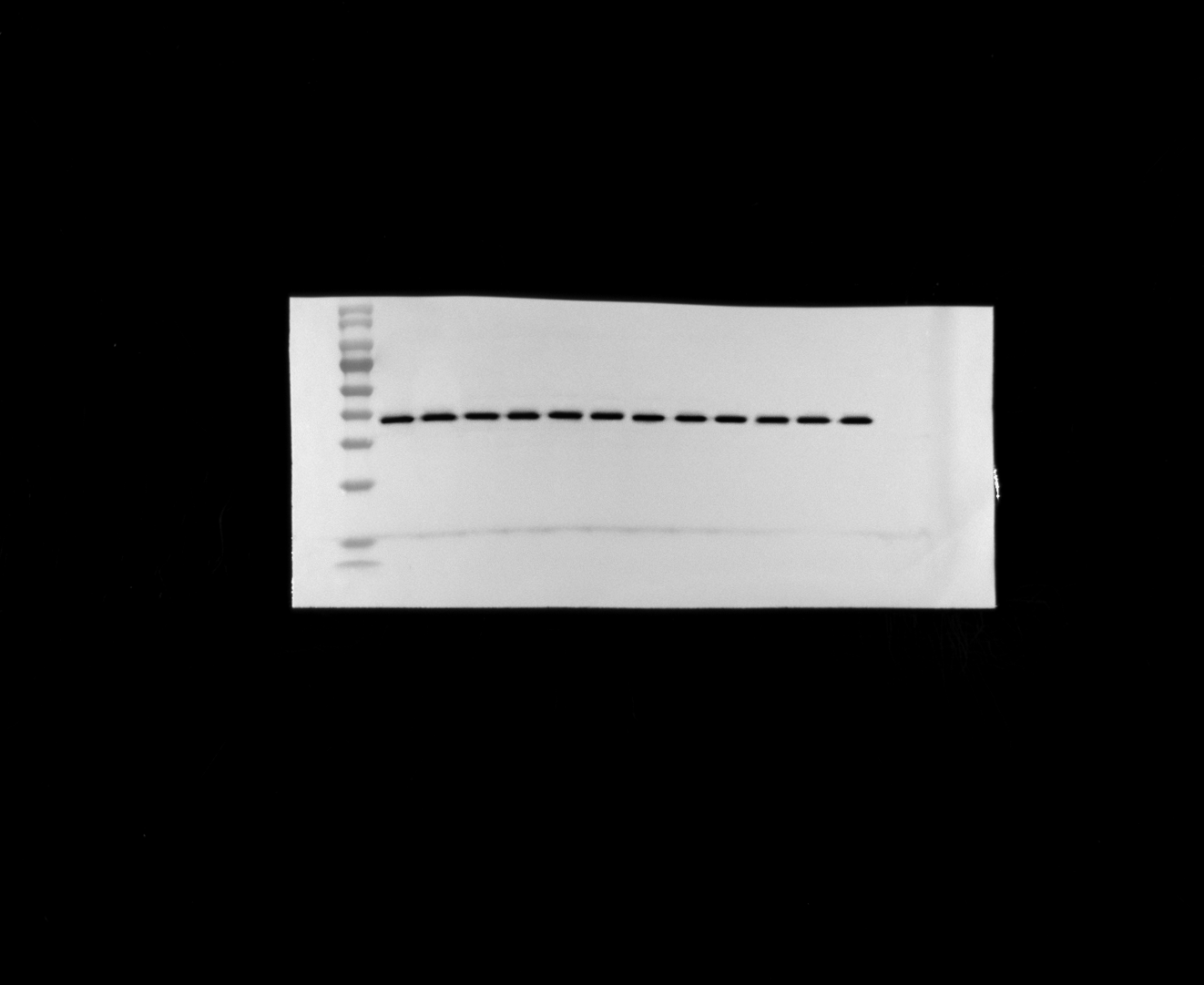

Supplement: Supplementary file 2 — Supplementary Material 2. [file 12935_2025_3665_MOESM2_ESM.zip › Supplementary Material 2/Figure S2/Figure S2A/MDA-MB-231 β-actin.tif]

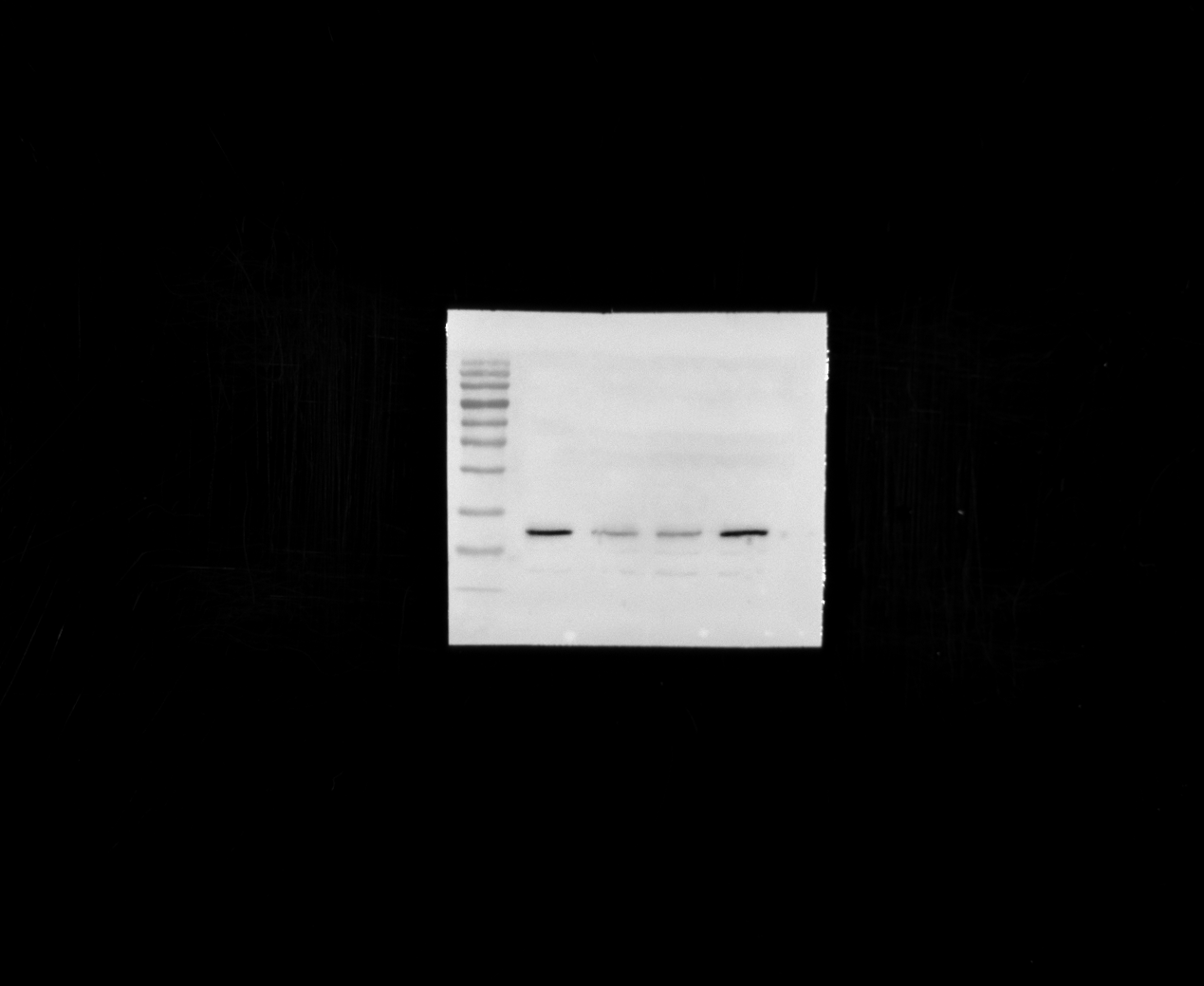

Supplement: Supplementary file 2 — Supplementary Material 2. [file 12935_2025_3665_MOESM2_ESM.zip › Supplementary Material 2/Figure S2/Figure S2E/MCF-7 Ferritin.tif]

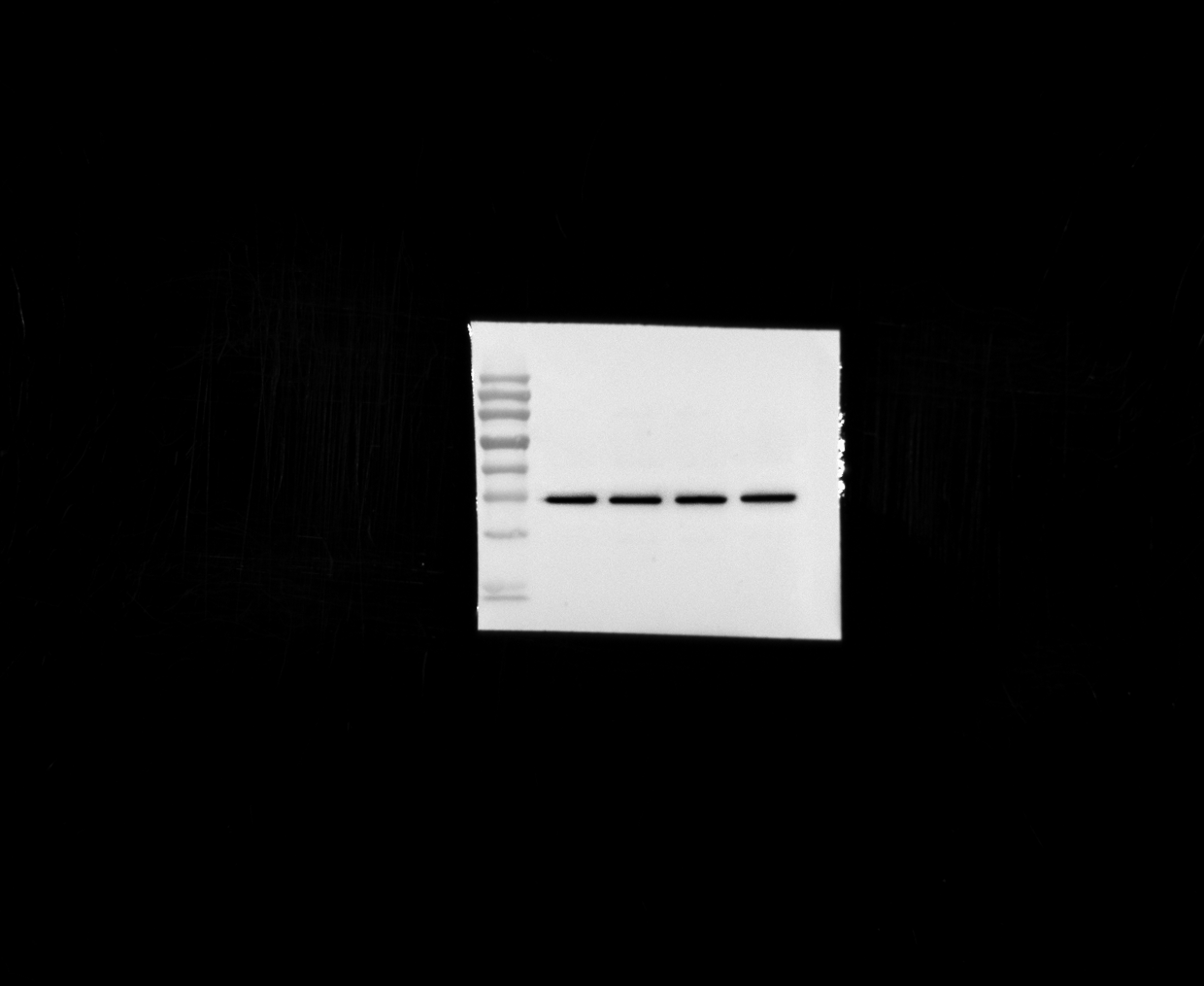

Supplement: Supplementary file 2 — Supplementary Material 2. [file 12935_2025_3665_MOESM2_ESM.zip › Supplementary Material 2/Figure S2/Figure S2E/MCF-7 β-actin.tif]

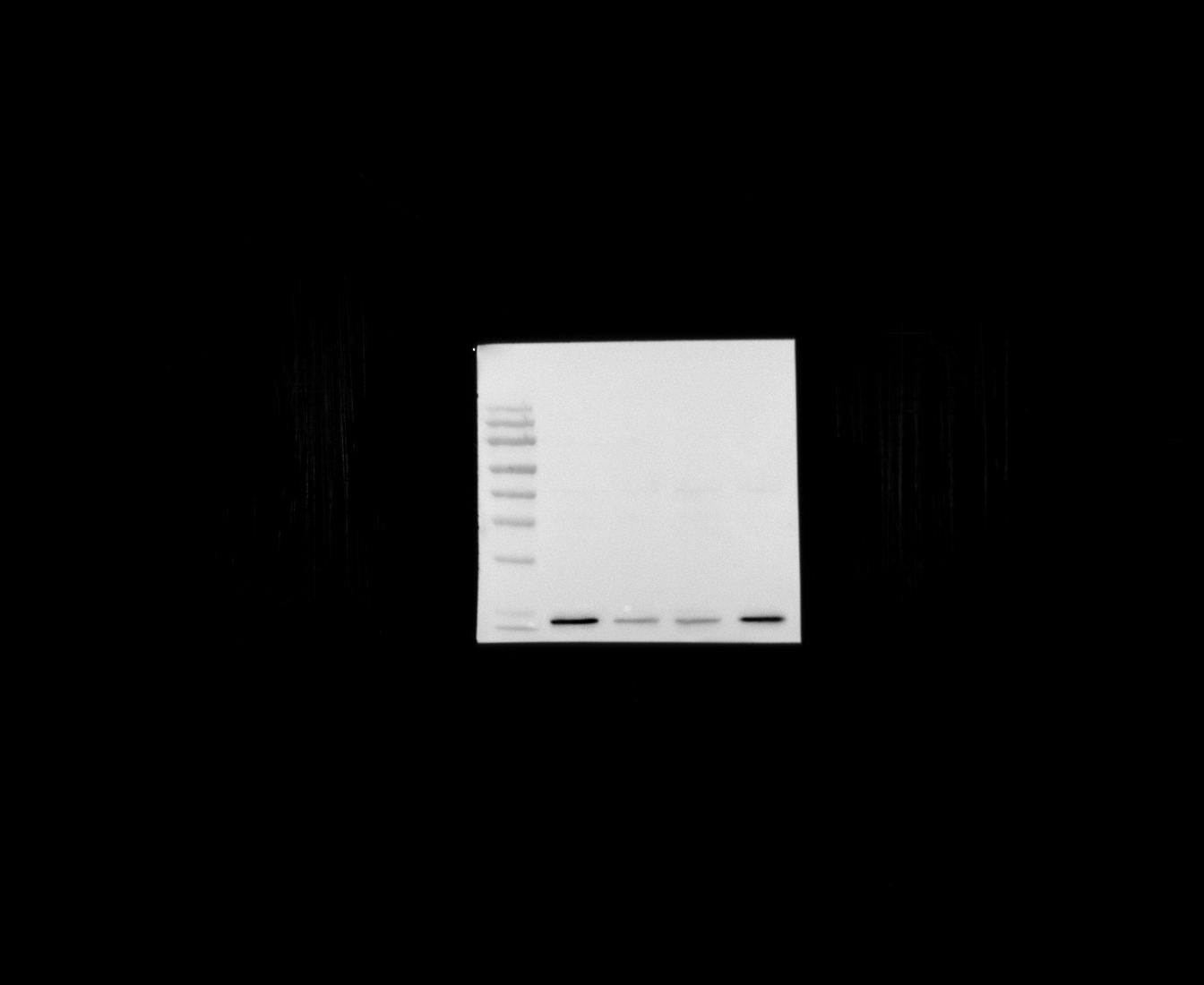

Supplement: Supplementary file 2 — Supplementary Material 2. [file 12935_2025_3665_MOESM2_ESM.zip › Supplementary Material 2/Figure S2/Figure S2E/MDA-MB-231 Ferritin.tif]

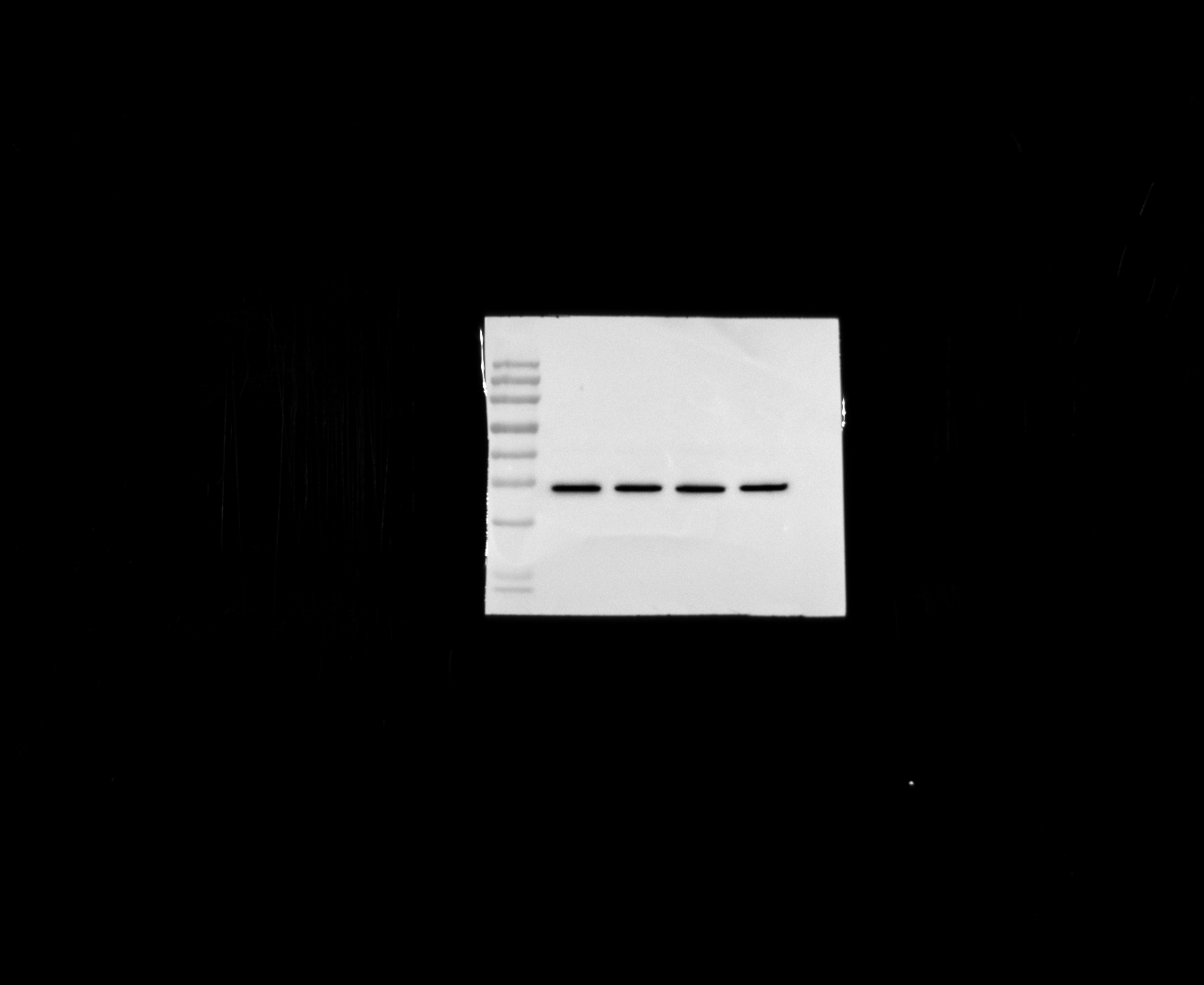

Supplement: Supplementary file 2 — Supplementary Material 2. [file 12935_2025_3665_MOESM2_ESM.zip › Supplementary Material 2/Figure S2/Figure S2E/MDA-MB-231 β-actin.tif]
